# Supplementary material for: Polygenic prediction of educational attainment within and between families from genome-wide association analyses in 3 million individuals
Source: Nat Genet. 2022 Mar 31;54(4):437–49. doi: 10.1038/s41588-022-01016-z (PMC9005349; doi:10.1038/s41588-022-01016-z)

nature portfolio

https://doi.org/10.1038/s41588-022-01016-z

Supplementary information

Polygenic prediction of educational attainment within and between families from genome-wide association analyses in 3 million individuals

In the format provided by the authors and unedited

Supplementary Information for

Polygenic prediction of educational attainment within and between families from genome-wide association analyses in 3 million individuals

Correspondence to:

Daniel Benjamin, daniel.benjamin@gmail.com

Aysu Okbay, a.okbay@vu.nl

Peter Visscher, peter.visscher@uq.edu.au

Alexander Young, alextisyoung@gmail.com

Contents

Supplementary Note **5**
1 Coding the educational attainment (EA) variable **5**
1.1 Educational attainment recoding in the UK Biobank 5
1.1.1 EA2/EA3 coding 5
1.1.2 NVQ, HND and HNC: qualification description 6
1.1.3 NVQ/HND/HNC/equivalent and EA 8
1.1.4 EA4 coding 9
1.2 Impact of including individuals aged 16-29 years old in the 23andMe GWAS 10
1.2.1 Recoding of the EA4 UKB EA measure for the simulation 10
1.2.2 Results 13
2 Additive GWAS **14**
2.1 Study overview 14
2.2 Methods 14
2.2.1 Cohorts 14
2.2.2 Phenotypes 14
2.2.3 Genotyping and imputation 14
2.2.4 Association analyses 15
2.2.5 Quality control 16
2.2.6 Meta-analysis ($\boldsymbol{N = 3,037,499}$) 17
2.2.7 Sensitivity analysis for definition of lead SNPs using COJO 19
2.2.8 Comparison of lead SNP definitions 20
2.3 Replication of EA3 lead SNPs 21
3 X chromosome analyses **26**
3.1 UK Biobank association analyses 26
3.1.1 Sex-stratified association analyses in UKB 26
3.1.2 Meta-analysis of sex-stratified association results in UKB 28
3.2 23andMe association analysis 29
3.3 Quality control of UK Biobank and 23andMe results 29
3.4 Meta-analysis of UK Biobank and 23andMe results ($\boldsymbol{N = 2,713,033}$) 29
3.5 Comparison to autosomes 30
4 Dominance GWAS **32**
4.1 Additive and dominance genetic variance: theoretical background 33
4.1.1 Genetic dominance at a single locus: background and definitions 33
4.1.2 Additive GWAS 34
4.1.3 Dominance GWAS 34
4.1.4 Partitioning the genetic variance 34

4.2 GWAS of dominance genetic variance: methods ... 35
4.2.1 Cohorts ... 35
4.2.2 Association analyses ... 35
4.2.3 Quality control ... 36
4.3 Dominance GWAS meta-analysis ($\boldsymbol{N = 2,574,253}$) ... 36
4.4 Statistical power to estimate dominance effects ... 37
4.5 Decomposition of the variation in the estimates from our dominance GWAS meta-analysis ... 39
4.6 Replications across our dominance GWAS of variance in the 23andMe and UKB data 41
4.6.1 Binomial replication tests ... 42
4.6.2 Replication results ... 42
4.6.3 Simulation framework to estimate the expected replication record ... 43
4.6.4 Estimating κ^2 ... 43
4.6.5 Simulation results ... 45
4.7 Directional dominance (inbreeding depression) ... 46
4.7.1 Background ... 46
4.7.2 Estimation of ID from dominance GWAS summary statistics ... 47
4.7.3 Analysis of summary statistics from the UKB and 23andMe ... 48
4.7.4 Reconciling evidence of directional dominance with evidence of negligible dominance variance ... 48
5 Predicting EA and cognitive performance ... 50
5.1 Constructing the polygenic indexes ... 50
5.2 Phenotypes ... 52
5.2.1 Education outcomes ... 52
5.2.2 Cognitive and academic achievement outcomes ... 53
5.3 Controls ... 54
5.4 Defining Prediction Accuracy ... 55
5.5 Expected Prediction Accuracy of the *EduYears* PGI ... 55
5.6 Results ... 56
5.7 Analysis of *European genetic ancestries to African genetic ancestries relative accuracy* in UK Biobank ... 59
5.8 Post-hoc analyses with SBayesR PGI ... 61
6 Prediction of disease risk from the EA PGI ... 62
6.1 Construction of PGIs ... 62
6.1.1 EA PGI ... 62
6.1.2 Diseases PGI ... 62
6.2 Prediction of disease from PGI ... 64

6.2.1 Analyses ... 64
6.2.2 Results ... 64
7 Comparing direct and population effects of the EA PGI ... 66
7.1 Population and Direct Effects of a PGI ... 66
7.2 Relationship to Analysis in Kong *et al.* (2018) ... 68
7.3 Datasets and phenotypes ... 68
7.4 Statistical Methods ... 69
7.4.1 Step 1: Estimating effects in the different subsamples ... 70
7.4.2 Step 2: Computing the sampling covariance matrix of the combined vector of effects 70
7.4.3 Step 3: Estimating the correlation between maternal and paternal PGI ... 71
7.4.4 Step 4. Meta-analyzing estimates ... 72
7.4.5 Step 5. Further transforming the estimates ... 72
7.4.6 Supplementary Results ... 73
8 Empirical Bayesian model and GWAS replication simulation framework ... 75
8.1 Posterior distribution of α_j ... 75
8.2 Posterior distribution of d_j ... 76
8.3 Proof that $\sigma_{d,j}^2 = (1 - 2p_j q_j)/N_j 2p_j q_j$ ... 78
8.4 GWAS replication simulation framework ... 80
8.5 Use of unadjusted summary statistics when fitting the empirical Bayesian model .. 81
9 Analysis of assortative mating ... 83
10 Proofs for sibling-based analysis ... 85
11 Additional acknowledgements ... 87
12 References ... 89
Supplementary Figures ... 95

Supplementary Note

1 Coding the educational attainment (EA) variable

Our coding of the educational attainment (EA) variable closely follows that of previous large-scale GWAS of educational attainment[1,2]. However, we have made small modifications to the coding of EA in the UKB, as we describe in **Supplementary Note** section 1.1. Also, while our previous GWAS excluded individuals aged less than 30 years old at the time EA was measured, the GWAS in the 23andMe cohort that we analyze in this paper includes individuals aged 16 to 29 years old; in **Supplementary Note** section 1.2, we report the results of a simulation using UKB data that suggests the impact of including individuals aged less than 30 in this cohort is negligible.

1.1 Educational attainment recoding in the UK Biobank

In previous large-scale GWAS of educational attainment (EA), UK Biobank participants who have a qualification in the category "NVQ or HND or HNC or equivalent" but no college or university degree were coded as having 19 years of education[1,2]. However, we have realized that this classification overstates the average EA of NVQ/NHD/HNC/equivalent holders. Furthermore, the diverse nature of the qualifications held by these individuals means that assigning one value to all might discard useful variation in the data. Accordingly, we have recoded EA for holders of an NVQ/HND/HNC/equivalent to take the value *{"Age left full-time education" - 5}*. To manage outliers, we have dropped all holders of an NVQ/HND/HNC/equivalent who report leaving full-time education before age 12. In this section, we provide details about the previous coding, which we refer to as the EA2/EA3 coding, and the revised coding, which we refer to as the EA4 coding.

1.1.1 EA2/EA3 coding

UK Biobank (UKB) participants had to answer the following question related to their EA:

1. "Which of the following qualifications do you have? (You can select more than one)"

Individuals without a college degree were further asked:

2. "At which age did you complete your continuous full-time education?"

Following Okbay et al. (2016)[1] (henceforth EA2), Lee et al. (2018)[2] (henceforth EA3) mapped individuals to an ISCED 1997 level based on their answers to Question 1. The following table summarizes the ISCED 1997 levels, which were used to harmonize the measure of educational attainment across cohorts:

ISCED 1997 levels and associated years of schooling

ISCED 1997 level	Definition	US years of schooling
0	Pre-primary education	1
1	Primary education or first stage of basic education	7
2	Lower secondary or second stage of basic education	10
3	(Upper) secondary education	13

4	Post-secondary non-tertiary education	15
5	First stage of tertiary education (not leading directly to an advanced research qualification)	19
6	Second stage of tertiary education (leading to an advanced research qualification, e.g. a PhD)	22

The following table shows the mapping from the UKB survey response categories to the ISCED 1997 level and EA coding that were used in EA2/EA3:

Mapping from UKB response to EA (EA2/EA3 coding)

UKB response		EA2/EA3 coding	
Qualification	Percentage	ISCED 1997 level	EA
College or university degree	32.4%	ISCED 5	20
A/AS Levels or equivalent	7.3%	ISCED 3	13
O Levels/GCSEs or equivalent	12.8%	ISCED 2	10
CSEs or equivalent	3.7%	ISCED 2	10
NVQ, HND, HNC or equivalent	10.7%	ISCED 5	19
Other professional qualification	14.5%	ISCED 4	15
None of the above	16.9%	ISCED 1	7
No response	1.9%	Excluded	Excluded

Note: Number of individuals in UKB sample is 502,602. Percentage is share coded with the EA value corresponding to that qualification.

Since UKB participants could report more than one qualification, the value of EA for each individual was calculated as follows. Let X_i be the set of all qualifications held by individual i and $f(X_i)$ the set of associated EA values (the image of X_i) under the above mapping. Then $EA_i = \max\{f(X_i)\}$.

In the UKB, one survey response category is “NVQ or HND or HNC or Equivalent”. EA2 and EA3 coded this group of qualifications as equivalent to 19 years of education. This is just one year less than those who hold a college degree and six years more than those who hold A Levels. We believe that the following evidence shows that this coding overstates the educational attainment of NVQ/HND/HNC/equivalent holders.

1.1.2 NVQ, HND and HNC: qualification description

NVQ and other vocational qualifications

National Vocational Qualifications (NVQs) and other vocational qualifications are a wide range of work-based qualifications that aim to certify that the holder can perform their job at a high standard. Accordingly, vocational qualifications in the UK span a broad range of difficulties, lengths and modes. For example, NVQs can range from entry-level (ISCED 1997 level 2) to doctoral level (ISCED 1997 level 6) qualifications, using official ISCED Mappings and UK qualification level classifications[3,4].

However, between 2012 and 2020, the vast majority of vocational qualifications awarded in the UK were awarded at low levels, as the following table shows:

Vocational qualifications awarded by qualification level, 2012-2020

UK level	ISCED 1997 level	Proportion of awards
Entry level	2	11.7%
Level 1	2	23.6%
Level 1/2	3	5.5%
Level 2	3	39.5%
Level 3	3	17.9%

Level 4	4	1.0%
Level 5	4	0.6%
Level 6	5	0.2%
Level 7	6	0.09%
Level 8	6	0.004%

Note: Figures calculated using Ofqual's Vocational Qualifications dataset for period Q1 2012-Q1 2020. 56,430,940 total qualifications awarded. https://www.gov.uk/government/statistical-data-sets/vocational-qualifications-dataset.

The above data are recent, whereas most participants in the UKB would have acquired their vocational qualification during the latter half of the 20th century. These individuals would have interacted with a different vocational qualifications landscape, as the framework for vocational education has changed somewhat over the years, with the 1986 introduction of the NVQ perhaps the biggest change. However, the variety and type of qualifications on offer have remained similar as official frameworks have changed, and the nature of vocational qualifications as non-academic qualifications has also remained constant[5]. We believe that the relative sparsity of high-level vocational qualifications likely carries over to earlier periods. Accordingly, the evidence provided in the above table (displayed graphically as Panel **a** of **Supplementary Figure 3**) suggests that the majority of NVQs and other vocational qualifications held by those in the UKB sample are likely to be at ISCED 1997 Levels 2 and 3.

HNC/HND

The Higher National Certificate (HNC) is a higher education qualification that is equivalent to the first year of an undergraduate degree. It usually takes one year to complete full time or two years part-time. Thus, the HNC is roughly in between ISCED 1997 Levels 4 and 5.

The Higher National Diploma (HND) is a higher education qualification in which students attain an HNC in the first year and subsequently continue their study in the second year. Accordingly, the HND is roughly equivalent to the first two years of an undergraduate degree, and also sits between ISCED 1997 Levels 4 and 5.

NVQ, HNC, and HND over time

We have not been able to find data that would allow us to be precise about the relative frequency of different types of qualifications in the category "NVQ or HNC or HND or equivalent". However, we can make some rough judgements based on the available data.

Approximately 6.5 million vocational qualifications were awarded per year from 2015 to 2019, and 7.5 million were awarded in the academic year 2015/16. Since individuals often attain more than one vocational qualification in a year, these figures overstate the number of vocational qualification holders. However, between 1985 and 2001, the percentage of 16 and 17-year-olds in vocational and work-based training in England remained almost constant at 40%[5]. This 40% projects to roughly 300,000 16/17-year-olds per cohort undertaking vocational and work-based training in the UK between 1985 and 2001, based on a cohort size of 740,000[1]. Moreover, the stability of this

[1] In the UK, live births averaged 760,000 per year from 1969 to 1985 and 880,000 per year from 1940 to 1970[84–86]. Calculations show that approximately 70% of U16 deaths occurred under the age of 1 from 1980 to 2012[87]. The U16 mortality rate was imputed from historical infant mortality rates with net U16 migration assumed to be 0. The imputed average U16 mortality rate is 4.5% for 1940-1970 and 2% for 1969-1985[88]. All estimates are rounded to two significant figures.

percentage suggests that vocational qualifications might have been similarly popular during the earlier time period (roughly 1960-1990) when most UKB participants were acquiring qualifications. Using an estimated cohort size of 840,000 for the period 1940-1970[1], approximately 340,000 16/17-year-olds per cohort are projected to have undertaken vocational and work-based training in this time period.

In contrast, approximately 15,000 students per year received either the HNC or the HND between 2015 and 2019[6]. More broadly, 216,170 learners were enrolled in ISCED 1997 level 4/5 qualifications similar to and including the HNC/HND (sub-bachelor higher qualifications) in 2015/2016[7], which sets an upper bound on the number of sub-bachelor higher qualifications awarded in 2015/16. This upper bound is over an order of magnitude smaller than the yearly number of vocational awards over the same time period (which was ~6.5 million, as mentioned above). Moreover, evidence suggests that enrolment in ISCED 1997 level 4/5 qualifications has remained largely constant over the past 60 years: in both 1962/1963 and 1979/1980, roughly 200,000 students were enrolled in sub-bachelor higher education at ISCED 1997 levels 4 and 5[8] (a number similar to the 216,170 in 2015/2016, as mentioned above). This is less than estimated enrolment in vocational qualifications among just 16/17-year-olds from 1960 to 1990.

Summary

Given these data, it seems likely to us that sub-bachelor higher qualifications were awarded less frequently than vocational qualifications across the time period in which UKB participants were acquiring the majority of the qualifications that they currently hold. Accordingly, sub-bachelor qualifications are likely not as common as vocational qualifications among UKB participants, although the exact extent of the divide is unclear.

The range of qualifications in the category “NVQ or HND or HNC or equivalent” is broad, including a variety of vocational and academic qualifications. Data on qualification attainment suggests that qualifications held by those in this category are mostly vocational qualifications at ISCED 1997 levels 2 and 3, with some sub-bachelor higher qualifications at ISCED 1997 levels 4 and 5.

1.1.3 NVQ/HND/HNC/equivalent and EA

The second table above shows that an NVQ/HND/HNC/equivalent is the highest qualification for 10.7% of the sample. This group constitutes a non-negligible portion of the UKB cohort, so it is important that the EA variable be correctly coded for NVQ/HND/HNC/equivalent holders. However, the above analysis suggests that the EA2/EA3 coding of NVQ/HND/HNC/equivalent holders as having 19 years of schooling, which corresponds to ISCED 1997 Level 5 and is just one year less than the coding for college, is potentially erroneous.

Data from the UKB support this conclusion. The distribution of “Age left full-time education (FTE)” (from Question 2) for those whose qualification is NVQ/HND/HNC/equivalent is depicted in Panel **b** of **Supplementary Figure 3**, and the mean “Age left FTE” is 17. This is much lower than would be suggested by the original coding of 19 years of education, and there is also substantial heterogeneity in “Age left FTE” among this group.

Moreover, the EA2/EA3 coding does not give an increasing relationship with other measures of education and socioeconomic status. In Panels **c** and **d** of **Supplementary**

Figure 3, “Average age left FTE” and “Prop. of hholds earning >£52k p.a.” are increasing in “EA (EA2/EA3 coding)” only up to the point of 15 or 13 years of education, respectively. NVQ/HND/HNC/equivalent holders have both a lower “Average age left FTE” and lower earnings than those with A Levels, those with professional qualifications, and those with a college education. The gap between NVQ/HND/HNC/equivalent holders and college graduates in terms of the proportion earning more than £52,000 per year is especially large.

Overall, the data suggest that NVQ/HND/HNC/equivalent holders should not be coded as having 19 years of education. To do so overstates the EA of those who hold such qualifications and is inconsistent with other correlated indicators of socioeconomic status. Accordingly, the EA variable should be recoded.

1.1.4 EA4 coding

The only change to the EA coding in EA4 vs. in EA2/EA3 is that EA for NVQ/HND/HNC/equivalent qualifications is coded as *{“Age left FTE” - 5}*, which aims to represent the number of years someone with such a qualification might have spent in FTE, given a school-beginning age of 5. Again, the value of EA for each individual was calculated as follows. Let X_i be the set of all qualifications held by individual i and $g(X_i)$ the set of associated EA values (the image of X_i) under the EA4 mapping. Then $EA_i = \max\{g(X_i)\}$.

Subsequently, all observations reporting less than seven years of schooling are dropped to exclude outliers (there are fewer than 50 such observations). The EA4 coding is as follows, with the change from the EA2/EA3 coding shaded in gray:

New EA coding

Qualification	ISCED 1997 level	EA
College or university degree	ISCED 5	20
A/AS Levels or equivalent	ISCED 3	13
O Levels/GCSEs or equivalent	ISCED 2	10
CSEs or equivalent	ISCED 2	10
NVQ or HND or HNC or equivalent	ISCED 2/3	“Age left FTE” – 5
Other prof. qual. (e.g., nursing, teaching)	ISCED 4	15
None of the above	ISCED 1	7
No response	Excluded	Excluded

This modification aims to capture the heterogeneity within the category “NVQ or HNC or HND or equivalent”, as discussed above. Individuals who attain qualifications in this category show considerable variation in “Age left FTE”. Adopting the new coding allows EA to be measured more accurately for those who have vocational or higher education qualifications. Moreover, only 3.5% of those whose highest qualification is “NVQ or HNC or HND or equivalent” have missing data for “Age left FTE”, so the updated coding does not discard a large amount of data. While some individuals provide very high responses to Question 2 (i.e. “Age left FTE” greater than 30), these are a small fraction (<1%) of the data (and were not discarded).

Panel **e** of **Supplementary Figure 3** demonstrates that the above change leads to a more consistent relationship between “Age left FTE” and “EA (EA4 coding)”. In particular, the large gap in “Age left FTE” between NVQ/HND/NHC/equivalent holders and college graduates has been eliminated.

Panel **f** of **Supplementary Figure 3** also suggests that the new coding better represents actual EA for the majority of those with an NVQ/HND/HNC/equivalent. Considering only individuals coded with values of the EA variable for which there are at least 1,500 observations, those with higher EA tend to earn more. However, the earnings pattern for the small group of NVQ/HND/HNC/equivalent holders who report spending an extreme amount of time in FTE ("Age left FTE" greater than 22) is less consistent. The proportion of individuals earning more than £52,000 per year within this group is roughly 20% and decreasing in "Age left FTE". It seems that these people do not truly have EA equivalent to more than 17 years, which is a source of measurement error. However, the total number of individuals who report extreme values for "Age left FTE" is less than 1% of the sample (see Panel **b** of **Supplementary Figure 3**). Thus, it is not likely that these individuals have much influence on estimated coefficients.

1.2 Impact of including individuals aged 16-29 years old in the 23andMe GWAS

In previous analyses of EA[1,2], the SSGAC has typically excluded anyone aged less than 30 years old to ensure that almost everyone in the sample has completed their formal schooling. However, the 23andMe GWAS of EA for EA4 includes individuals aged 16-29 years old, who constitute ~16% of the 23andMe sample. This gives rise to potential issues of truncation, where some individuals who have not completed their formal schooling report the qualifications they currently have, rather than the complete set of qualifications they will attain. For these individuals, EA represents "total years of education at survey date" rather than "total years of education", which is the intended meaning of EA.

To explore the effect of this potential truncation on GWAS results, we conducted a simulation with the UKB data. The exercise aims to simulate the GWAS of additive genetic variance that would have been run in the UKB had 16% of the UKB participants responded to the education-related questions between the ages of 16 and 29 years old. For this, as we describe in more detail below, we recoded the EA measure for a random 16% of the UKB participants to mimic what the measure would have been had the respondent been surveyed before age 30; we then ran a GWAS of this recoded EA measure in the UKB (combining the recoded 16% with the remaining 84% of participants); finally, we computed the genetic correlation between the resulting summary statistics and the summary statistics from our baseline GWAS of additive variance for EA in the UKB.

1.2.1 Recoding of the EA4 UKB EA measure for the simulation

As mentioned in **Supplementary Note** section 1.1, UK Biobank (UKB) participants had to answer the following question related to their EA:

1. Which of the following qualifications do you have? (You can select more than one)

Individuals without a college degree were further asked:

2. At which age did you complete your continuous full-time education?

Each individual was then assigned the maximum EA associated with the qualifications that they held, as summarized in the above table. Individuals with EA < 7 were excluded.

In this simulation, we aim to code a new EA measure that is consistent with how individuals would have answered the above questions at an age in the range 16-29.

Recoding

To simulate the nature of the data collection issue in the 23andMe cohort, a new variable indicating "*Educational Attainment at Response" (EAR)* is derived for each individual by the following process:

1. Select 16% of the individuals in the UKB at random and define these as the U30 Sample for the simulation. This selects 16% of the sample to be treated as 16-29 years old.
2. For the remaining 84% of the sample, let EAR = EA.
3. For each individual in the U30 Sample:
    a. Draw an "Age at Response" (AAR) from a $U[16, 29]$ distribution.[2] Let "Maximum Educational Attainment at Response" (MEAR) be defined as AAR – 5.[3]
    b. If an individual did not attain a college degree:
        i. Disallow all qualifications other than NVQ/HND/HNC for which the associated EA value from the EA4 coding (see **Supplementary Note** section 1.1.4) is greater than MEAR (i.e., disallow all the qualifications that an individual is unlikely to have attained at their AAR).
        ii. If one has an NVQ/HND/HNC qualification and AAR < "Age Left FTE", disallow the NVQ/HND/HNC qualification. Then, if AAR ≥ "Age Left FTE" – 2, assume the individual has obtained another NVQ/HND/HNC at age "Age Left FTE" – 2 (with corresponding EA coding of "Age Left FTE" – 7). If AAR ≤ "Age Left FTE" – 2, then disallow all NVQ/HND/HNC qualifications.[4]
        iii. If an individual has at least one qualification that has not been disallowed, code EAR according to the standard UKB coding for their highest remaining qualification.

[2] We do not know the distribution of ages among the 23andMe respondents who were surveyed before age 30. We believe assuming a uniform distribution is likely to be a conservative assumption.

[3] Five is the age at which a child starts education in the UK.

[4] Note that EA4 codes NVQ/HND/HNC as "Age left FTE" – 5, and thus implicitly assumes that an individual with such a degree obtained that qualification at the age they left FTE. Thus, if AAR < "Age Left FTE", the individual would not have obtained the NVQ/HND/HNC. However, to allow for the possibility that the individual could have obtained another NVQ/HND/HNC prior to the one they obtained at "Age left FTE", we assume they obtained another NVQ/HNC/HNC at "Age left FTE" – 2.

iv. Else if an individual does not have any qualifications that have not been disallowed, code EAR = $7\alpha + (1 - \alpha)$MEAR, where $\alpha = 0.7$.[5]

c. If an individual did obtain a college degree:

i. If the individual also attained an NVQ/HND/HNC, let the associated EA value for the NVQ/HND/HNC be 13.[6]

ii. If AAR >= 22, code EAR according to the standard UKB coding (i.e., EAR=EA=20).[7]

iii. If AAR < 22, disallow the college degree, and then:

- If the individual disclosed only their college degree, assume that the individual also holds A/AS Levels and O Levels/GCSEs.[8]
- Whether the individual disclosed other qualifications or not, disallow all qualifications for which the associated EA value is greater than MEAR.
- If an individual has at least one qualification that has not been disallowed, code EAR according to the standard UKB coding for their highest remaining qualification.
- Else if an individual does not have any qualifications that have not been disallowed, code EAR = $7\alpha + (1 - \alpha)$MEAR, where $\alpha = 0.7$.

4. Rerun the GWAS using *EAR* as the phenotype and compute the genetic correlation between the summary statistics from the original and those from the new GWAS using EAR.

Examples

---

[5] If an individual has no remaining qualifications, they may either truly have no other qualifications, in which case EAR should be coded as 7 (the value associated with having no formal qualifications in the original coding). Alternatively, they might have misread the first question (which asks about all qualifications) and responded only with their highest qualification. In that latter case, there is a danger of under-coding EAR. A weighted average of 7 and MEAR should correct this potential bias. Based on the fact that 30% of college degree holders (who should all have other qualifications) report no other qualifications, a reasonable value of α might be 0.7.

[6] People who did not obtain a college degree did not answer question 2 (about the age at which they completed FTE), the responses to which are used to impute the EA of NVQ/HND/HNC holders. Thus, we have to impute a suitable value of EA for college-degree holders who also obtained an NVQ/HND/HNC. We take the imputed EA value for the NVQ/HND/HNC holders who have a college degree as the average “Age Left FTE -5” of the NVQ/HND/HNC holders without college degrees in the sample. While this is an ad hoc solution, only 10% of college degree holders also report having an NVQ/HND/HNC, so the effect of any error should be small.

[7] We effectively assume that college degrees are completed at age 22. We use a cut-off of 22 rather than the implied 25 from the baseline EA coding for college-degree holders. (EA for college-degree holders is coded as 20, and education in the UK begins at age 5. The baseline EA coding assigns an EA of 20 to anyone with an undergraduate degree of more, and thus pools both undergraduate and graduate degrees. The modal student obtains an undergraduate degree and graduates before 25, which justifies the lower cut-off.)

[8] The majority of individuals who went to college will have A/AS Levels and O Levels/GCSEs. We can impute these qualifications for people who misread the question and only disclosed their highest/most recent qualification.

Individual 1

Individual 1 reports the following qualifications:

- College or university degree (*EA = 20)*
- A/AS Levels or equivalent (*EA = 13*)
- O Levels/GCSEs or equivalent (*EA = 10*)

This individual is originally coded with *EA* = 20. Suppose that *AAR* is drawn as 17. Then *MEAR* is 12. Since the individual holds a college degree and *AAR < 22*, the college degree is disallowed. In the next step, the A/AS Levels are also disallowed, but the O Levels/GCSEs are allowed. Individual 1 is left with the following qualification: O Levels/GCSEs or equivalent (*EA = 10*). So, individual 1 will be coded with *EAR = 10*.

Individual 2

Suppose that Individual 2 has the same qualifications as individual 1 but only reports the college degree. They are originally coded as *EA = 20*, and *AAR* is again drawn as 17, so *MEAR* is 12. As above, the college degree is disallowed, so now individual 2 has no remaining qualifications. Then, we impute A/AS Levels and O Levels/GCSEs. Based on *MEAR*, we eliminate A/AS Levels. So, like individual 1, individual 2 will be coded with *EAR = 10*.

Individual 3

Individual 3 reports the following qualifications:

- O Levels/GCSEs or equivalent (*EA = 10*)
- NVQ/HND/HNC or equivalent (*EA = 14*)

AAR is drawn as 18, so *MEAR* is 13. Then NVQ/HND/HNC or equivalent is reduced by two and retained, while O Levels/GCSEs are also retained. Then *EAR = 12*.

1.2.2 Results

We ran a GWAS of EAR in the UKB and computed the genetic correlation between the resulting summary statistics and the summary statistics from our baseline GWAS of additive variance for EA in the UKB. We estimated a genetic correlation that is indistinguishable from unity ($\hat{r}_g = 0.9985,\ SE = 0.0022$) between the two sets of summary statistics, thus indicating that the inclusion of individuals aged 16-29 years old at the time when EA was measure would not have substantially affected our UKB GWAS. We conclude that the inclusion of individuals in that age range in the 23andMe GWAS is unlikely to materially affect the results we report in this paper.

2 Additive GWAS

2.1 Study overview

Our primary analysis is a genome-wide association study (GWAS) on educational attainment that extends the GWAS discovery sample size from $N = 1{,}131{,}881$ in our previous GWAS to $N = 3{,}037{,}499$.

Below, we describe the methods of our additive GWA analysis and summarize the key findings.

2.2 Methods

2.2.1 Cohorts

Our primary additive GWA analysis builds on a previous GWAS on educational attainment[2] (henceforth referred to as EA3) that analyzed 1.1 million individuals from 71 studies. The EA3 study, in turn, extended another GWAS on educational attainment (EA2)[1] that combined 64 discovery cohorts and one replication cohort ($N = 405{,}072$). Here, we increase the sample size to N = 3,037,499 by meta-analyzing three sets of summary statistics: publicly available results from EA3 that exclude 23andMe and UKB (N = 324,162), new association results from 23andMe (N = 2,272,216), and new association results from a GWAS we conducted in UKB with an improved coding of the EA measure (N = 441,121; see **Supplementary Note** section 1). For summary information about the 23andMe and UKB cohorts, see Panel **A** of **Supplementary Table 15**). 23andMe research participants provided informed consent and participated in the research online, under a protocol approved by the external AAHRPP-accredited IRB, Ethical & Independent Review Services (E&I Review). Participants were included in the analysis on the basis of consent status as checked at the time data analyses were initiated. Analysis of the UK Biobank was conducted under application numbers 11425 and 12505. Informed consent was obtained from UK Biobank subjects. For the remaining cohorts included in our analysis, see Supplementary Table 16 of Lee et al.[2] and Supplementary Table 1.1 of Okbay et al.[1]).

2.2.2 Phenotypes

As in our prior work, we analyze the *EduYears* phenotype obtained by mapping the highest level of education that a respondent achieved to an International Standard Classification of Education (ISCED) category and then imputing a years-of-education equivalent for each ISCED category (see **Supplementary Note** section 1.1.1 for the ISCED to years-of-education mapping). The phenotype measurement and distribution for the 23andMe cohort and the updated UKB GWAS (see **Supplementary Note** section 1) are summarized in Panel **B** of **Supplementary Table 15**. For analogous information on the remaining cohorts, see Supplementary Tables 17 and 1.3 in Lee et al.[2] and Okbay et al.[1], respectively. Across all 71 cohorts, the sample-size-weighted mean of *EduYears* is 15.4 years of schooling with a standard deviation of 3.4.

2.2.3 Genotyping and imputation

The genotyping platform, pre-imputation quality-control filters applied to the genotype data, subject-level exclusion criteria, imputation software used, and the reference

sample used for imputation for all cohorts other than 23andMe are summarized in Supplementary Tables 18 and 1.4 of Lee et al.[2] and Okbay et al.[1] respectively. The remainder of this subsection discusses genotyping and imputation for the 23andMe cohort.

DNA extraction and genotyping were performed on saliva samples by National Genetics Institute (NGI). Samples were genotyped on five different genotyping platforms (see Panel **C** of **Supplementary Table 15**). Samples that failed to reach 98.5% call rate were re-analyzed. Individuals whose analyses failed repeatedly were re-contacted by 23andMe customer service to provide additional samples.

Imputation was conducted with a reference panel that combines the May 2015 release of the 1000 Genomes Phase 3 haplotypes[9] with the UK10K imputation reference panel[10]. Prior to imputation, each chromosome of the reference panel was split into chunks of no more than 300,000 variants, with overlaps of 10,000 variants on each side. A single batch of 10,000 individuals was used to estimate Minimac3[11] imputation model parameters for each chunk.

Phased participant data were generated using Finch, a tool internally developed by 23andMe that implements the Beagle graph-based haplotype phasing algorithm[12], and Eagle2[13]. Haplotype graphs were constructed for all participants from a representative sample of genotyped individuals, and then out-of-sample phasing of all genotyped individuals was performed against the appropriate graph. For the X chromosome, separate haplotype graphs were built for the non-pseudoautosomal region and each pseudoautosomal region, and these regions were phased separately. Phased participant data were imputed against the merged reference panel using Minimac3, treating males as homozygous pseudo-diploids for the non-pseudoautosomal region.

2.2.4 Association analyses

Cohorts were asked to estimate the following regression equation for each measured SNP

$$EduYears = \beta_0 + \beta_1\, SNP + \boldsymbol{PC}\,\boldsymbol{\gamma} + \boldsymbol{B}\,\boldsymbol{\alpha} + \boldsymbol{X} + \epsilon,$$

where *SNP* is the allele dose of the SNP; $\boldsymbol{PC}$ is a vector of the first ten principal components (PCs) of the variance-covariance matrix of the genotypic data, estimated after the removal of genetic outliers; $\boldsymbol{B}$ is a vector of standardized controls, including a third-order polynomial in year of birth, an indicator for being female, and their interactions; and $\boldsymbol{X}$ is a vector of study-specific controls. Samples were restricted to individuals whose *EduYears* was measured no earlier than age 30 and who passed the cohort's quality control, which should include the removal of genetic outliers, individuals with poor genotyping rates, and individuals that are not of European genetic ancestries. Supplementary Tables 19 and 1.5 in Lee et al.[2] and Okbay et al.[1], respectively, describe the study-specific details on the association analyses for all cohorts other than 23andMe.

For the GWAS in 23andMe, participants were first restricted to a set of individuals who are of European genetic ancestries, as determined through an analysis of local genetic ancestry[14]. The reference population data was derived from public datasets (the Human Genome Diversity Project, HapMap, and 1000 Genomes), as well as 23andMe customers who have reported having four grandparents from the same country. A maximal set of unrelated individuals was chosen using a segmental identity-by-descent (IBD) estimation algorithm[15]. Individuals were defined as related if they shared more

than 700 cM IBD, including regions where the two individuals share either one or both genomic segments IBD.

Association test results were computed for the genotyped and the imputed SNPs. For tests using imputed data, imputed dosages were used rather than best-guess genotypes. For each marker, a linear regression was performed following the specification above with age instead of birth year and additional controls for genotype batch effects.

PCs were computed using ~65,000 high quality genotyped variants present in all five genotyping platforms on a subset of 1 million participants randomly sampled across all the genotyping platforms. PCs for participants not included in the analysis were obtained by projection.

2.2.5 Quality control

To the new 23andMe and UKB results, we applied a quality control protocol close to the one described in EA2 and implemented in the EasyQC R package. We deviated from the EA2 protocol in the following steps:

- We used data from the Haplotype Reference Consortium reference panel (r1.1)[16], instead of 1000 Genomes Phase 1[17], to check for strand misalignment, position mismatch, allele concordance and allele frequency discrepancies.
- Because the 23andMe sample size was very large, applying a minor allele count threshold of 25 as in EA2 would mean preserving markers that are very rare. We wanted to err on the side of conservatism and apply a stricter filter of minor allele frequency (MAF) > 0.1%.
- We dropped genotyped markers with a call rate less than 95% or Hardy-Weinberg equilibrium exact test *P* value less than 10^{-20}. We chose a low Hardy-Weinberg *P* value cutoff because the sample sizes of the 23andMe and UKB cohorts were very large, which could result in markers with negligible deviations from Hardy-Weinberg equilibrium having very low Hardy-Weinberg *P* values and getting filtered out.
- We filtered out standard-error outliers by comparing the reported standard errors to the following approximation to the standard error of a coefficient estimated by OLS:

$$SE_{pred,j} = \frac{\hat{\sigma}_y}{\sqrt{N}} \times \frac{1}{\sqrt{2 \times MAF_j \times (1 - MAF_j)}},$$

  where $\hat{\sigma}_y$ is the standard deviation of *EduYears* in the input GWAS, MAF_j is the minor allele frequency of SNP j and N_j is the GWAS sample size for SNP j. We filtered out markers with $\frac{SE_{pred,j}}{SE_j} < \frac{1}{2}$ or $\frac{SE_{pred,j}}{SE_j} > 2$).

After the filtering step, we generated and visually inspected a set of diagnostic plots as described in the Supplementary Section 1.7 in Okbay et al.[1] to make sure there were no anomalies in the 23andMe and UKB results. **Supplementary Table 16** summarizes the number of markers dropped in each filtering step in these two GWAS.

2.2.6 Meta-analysis ($N = 3,037,499$)

We conducted a sample size weighted meta-analysis of all SNPs that passed the quality control thresholds in the new 23andMe and UKB results, and the 69 results files from EA3 (all except 23andMe and UKB) using the software METAL[18]. After applying a sample size filter of $500,000$, we were left with meta-analysis results for 10,675,380 autosomal SNPs. The upper panel of main text **Figure 1** displays the Manhattan plot for the meta-analysis.

Panel **A** of **Extended Data Figure 1** shows the quantile-quantile plot of the *P* values from the meta-analysis. The *P* values deviate strongly from a uniform distribution, consistent with a polygenic genetic architecture[19] ($\lambda_{GC} = 3.545$, calculated using the median χ^2), and the strength of this deviation depends on allele frequency. Panel B shows the distribution of *P* values stratified by allele frequency. For common variants defined as variants with MAF > 5%, the genomic inflation factor is 6.167. For low frequency variants with MAF between 1% and 5%, λ_{GC} goes down to 2.797, and for rare variants (0.1% < MAF < 1%), we have λ_{GC} = 1.840.

We did not apply genomic control to the cohort-level results prior to meta-analysis. Instead, we inflated the standard errors by the square root of the intercept $\left(\sqrt{1.663}\right)$ from an LD score regression[20] after the meta-analysis. The LD score regression was restricted to the set of HapMap3 SNPs with MAF > 1% with LD scores estimated using the 1000 Genomes Phase 1 reference sample[21]. Therefore, we are implicitly assuming that HapMap3 SNPs with MAF > 1% are representative of all SNPs included in our meta-analysis in terms of stratification or other biases. The GWAS in our meta-analysis were conducted on genotypes imputed to various reference panels (e.g. 1000 Genomes[21], Haplotype Reference Consortium (HRC)[16], etc.) that contain many SNPs with rare alleles, and our quality control pipeline allows SNPs with MAF between 0.1% and 1% to be kept. The LD score intercept adjustment could lead to an inflated Type-I error rate for these rare SNPs if the bias for rare SNPs were greater than that of common SNPs. We are not aware of any evidence on this point. We note, however, that we are less confident that the estimated LD score intercept is sufficiently adjusting for stratification biases for these rare SNPs.

In order to interpret the LD score regression intercept of 1.663, we compare it to the overall observed inflation. The average χ^2 statistic across HapMap3 SNPs in our sample is 9.906. Thus, the intercept estimate of 1.663 suggests that biases due to stratification or cryptic relatedness explain only a small share of the overall inflation in the test statistics, with roughly 93% (i.e. $1 - \frac{1.663-1}{9.906-1}$) being due instead to polygenicity. For a graphical summary of the LD score stratification analyses, see **Extended Data Figure 2**.

We selected the set of approximately independent genome-wide-significant SNPs using a clumping algorithm implemented in Plink[22]. The algorithm, with the parameters that we chose, works as follows: It first chooses the SNP with the smallest *P* value and then clumps with it all SNPs in the same chromosome that are in LD with it, as defined by $r^2 > 0.1$. The next clump is greedily formed around the SNP with the next smallest *P* value not already assigned to the first clump. This process was iterated until no SNPs remained with P value $< 5 \times 10^{-8}$. We calculated the LD between SNPs using 14,576,403 variants and 14,097 individuals from the public release of the HRC reference panel[16] that remained after the following QC filters: (i) genotyping call rate > 0.95, (ii) minor allele frequency > 0.001, (iii) Hardy-Weinberg equilibrium exact test

P value > 10^{-5}, (iv) subject missingness rate < 0.01, (v) realized genetic relatedness coefficient[23] < 0.025, and (vi) plink `--neighbor` Z score > -5. The result was 3,952 approximately independent clumps, each centered around a genome-wide-significant SNP. In what follows, we refer to these approximately independent SNPs as our lead SNPs. Of the 3,952 lead SNPs, 3,562 have minor allele frequencies greater than or equal to 5%, 357 between 1% and 5%, and 33 below 1%. The 3,952 lead SNPs are spread across the autosomes, with a randomly selected SNP having an 8% chance of being in a genome-wide-significant locus (i.e., pairwise $r^2 > 0.1$ with at least one lead SNP)[9].

To gauge the magnitude of the estimated SNP effects, we used a well-known approximation to transform the Z-statistics from the sample-size-weighted meta-analysis (the output of the software METAL) into unstandardized regression coefficients:

$$\hat{\beta}_j = Z_j \frac{\hat{\sigma}_y}{\sqrt{2\, N_j\, MAF_j\, (1 - MAF_j)}}$$

for SNP j with minor allele frequency MAF_j, sample size N_j, Z-statistic Z_j, and standard deviation of the phenotype $\hat{\sigma}_y$. For a derivation, see the SOM in EA1[24]. Without adjustment for winner's curse, the estimated effects (in absolute value) of the 3,952 lead SNPs are all in the range 0.006-0.085 SD units, corresponding to approximately 1 to 15 weeks of schooling per reference allele (assuming the standard deviation of *EduYears* is 3.4). The median, 25th percentile, and 75th percentile effect sizes are 0.009, 0.007, and 0.012 SD units (1.6, 1.2 and 2.1 weeks of schooling, respectively). An additional copy of the trait-increasing allele is on average associated with 0.011 SD units, which implies 1.9 weeks of schooling. When we consider common ($MAF \geq 5\%$), low-frequency ($1\% \leq MAF < 5\%$) and rare ($MAF < 1\%$) variants separately, the averages are instead 0.009, 0.021 and 0.041 SDs, corresponding to 1.6, 3.7, and 7.2 weeks of schooling. The minor allele frequency of the SNP with the largest effect size is 0.001.

Next, we used an empirical Bayesian approach to adjust the estimated effect sizes for the winner's curse. (See **Supplementary Note** section 8 for a description of our empirical Bayesian model.) We first estimated the parameters π and τ^2 that govern a spike-and-slab prior distribution for the additive effect sizes α_j. We used Maximum Likelihood Estimation (MLE) with the unadjusted summary statistics (see **Supplementary Note** section 8.5) for all 10,675,380 SNPs that passed our quality control filters. We obtained the estimates $(\hat{\pi}, \hat{\tau}^2) = (0.60, 3.277 \times 10^{-6})$. We then used the spike-and-slab prior distribution, treating these estimates as the true parameter values governing the prior, to calculate the posterior distribution of each SNP's true effect size. For all 3,952 lead SNPs, the estimated posterior probability π that the SNP captures signal from a true causal SNP for EA was estimated to be 1.0. We simulated the true effect sizes of the lead SNPs by drawing 1,000 times from the posterior distribution of each lead SNP, and then identified the 5th, 25th, 50th (median), 75th, and

---

[9] In EA3, we reported that the chance of a random SNP being in a genome-wide-significant locus was 17%. This number actually corresponded to the probability that a genome-wide-significant SNP randomly positioned on the genome is clumped together with one of the 1,271 lead SNPs instead of becoming a lead SNP itself. If we calculate the EA3 figure the same way we do here, we get 3%.

95th percentiles of the simulated effect sizes. The median simulated effect (in absolute value) of the 3,952 lead SNPs corresponds to 1.4 weeks of schooling per allele, and the effects at the 5th, 25th, 75th, and 95th percentiles are 0.9 weeks, 1.1 weeks, 1.9 weeks, and 3.5 weeks, respectively. These are our winner's-curse-adjusted estimated effect sizes.

In order to assess the overall homogeneity of effects across the new 23andMe and (updated-phenotype-measure) UKB results and the remaining cohorts from EA3 (i.e., the EA3 meta-analysis but excluding 23andMe and UKB), we estimated their pairwise genetic correlations using bivariate LD score regression[25]. We found genetic correlations close to 1, but statistically distinguishable from it: $r_g(23andMe, UKB) = 0.88\ (SE = 0.01)$; $r_g(23andMe, EA3) = 0.93\ (SE = 0.01)$; $r_g(UKB, EA3) = 0.96\ (SE = 0.01)$, suggesting some heterogeneity between the results.

2.2.7 Sensitivity analysis for definition of lead SNPs using COJO

We assessed the sensitivity of our conclusions about the number of lead SNPs with a conditional and joint multiple-SNP analysis (COJO)[26]. In our COJO analysis, we again used the HRC reference data that we used for clumping (described above in **Supplementary Note** section 2.2.6).

We performed COJO using the implementation found in the GCTA software[23]. Model selection was performed using the stepwise selection process outlined in the original COJO paper[26], in which SNPs from across the genome are iteratively added to the model. We set the LD window to 100 Mb, i.e., SNPs that are further than 100 Mb are assumed to have zero LD correlation.

Our COJO analysis identified 2,925 variants at genome-wide significance. 1,147 of these are also identified by the clumping algorithm. Another 1,055 are in LD ($r^2 \geq 0.1$) with a lead SNP identified by the clumping algorithm. The remaining 723 are independent ($r^2 < 0.1$) from the clumping lead SNPs. The mean χ^2 across these 723 SNPs is 19.4, much lower than the mean χ^2 across lead SNPs that are identified by both COJO and clumping (mean χ^2 = 70.1) or the COJO lead SNPs that are in LD with clumping lead SNPs (mean χ^2 = 52.0). This suggests that COJO is pushing the *P* values of some SNPs that are well above the genome-wide significance cutoff to below the cutoff by conditioning on other SNPs, thereby identifying additional associations. Conversely, by conditioning on other SNPs, COJO is pushing the *P* values of some SNPs that otherwise would be genome-wide significant to above the *P* value cutoff. These 2,805 SNPs are identified by the clumping algorithm but not by COJO, and 1,599 of them (mean $\chi^2 = 40.4$) are not in LD with any of the COJO lead SNPs. On average, the lead SNPs identified by both the clumping algorithm and COJO (or identified by only one of the methods but in LD with the lead SNPs from the other method) have higher minor allele frequencies (mean MAF = 0.28) compared to SNPs identified by only one of the methods (mean MAF = 0.22), suggesting that the two methods tend to disagree more on lower frequency SNPs.

In order to classify each of the COJO lead SNPs as either "primary" or "secondary", we applied our clumping algorithm to the list of 2,925 COJO variants (**Supplementary Table 2**). The clumping algorithm eliminated 41 SNPs from the list of COJO lead SNPs (pairwise $r^2 > 0.1$ with at least one COJO lead SNP with lower *P* value). We call these 41 SNPs secondary associations and the remaining 2,884 SNPs primary associations.

2.2.8 *Comparison of lead SNP definitions*

In GWAS with such large sample sizes as our current study, the χ^2 statistic can attain very large values for some SNPs, which can generate false positives when our clumping algorithm is used to identify lead SNPs. To see why, consider a SNP that is truly null but neighbors a lead SNP that has $\chi^2 > 300$. If the null SNP's LD with the lead SNP is just below the algorithm's cutoff of $r^2 = 0.1$ (and the null SNP is not in LD with any other SNPs), then purely due to its correlation with the lead SNP, it will have a χ^2 statistic just above the genome-wide significance threshold of 29.7, and hence will be incorrectly identified as another lead SNP. In practice, we believe most such false positives are generated due to a null SNP being in LD with two or more associated SNPs (rather than being in LD with just one truly associated SNP). We suspect it is because of this that we find so many more lead SNPs with our clumping than our COJO definition.

In some additional analyses, we assessed which of the two lead SNP definitions, clumping or COJO, is more suitable for GWAS conducted in very large samples. First, we compared the two methods in terms of the predictive power of polygenic indexes (PGIs) made using only lead SNPs ($P < 5 \times 10^{-8}$) in two of our prediction cohorts, the Health and Retirement Study (HRS) and the National Longitudinal Study of Adolescent to Adult Health (Add Health). For this analysis, we used a version of our meta-analysis that excludes HRS, Add Health, and the Wisconsin Longitudinal Study, which we obtained for the prediction analyses in **Supplementary Note** section 5. In order to maximize comparability, we restricted the meta-analysis to SNPs available in both HRS and Add Health. Next, we applied our clumping and COJO algorithms (described in **Supplementary Note** sections 2.2.6 and 2.2.7, respectively) to the summary statistics, which resulted in 3,878 clumping and 2,942 COJO lead SNPs. For each set of lead SNPs, we obtained PGIs using Plink2[27] as weighted sums of genotype probabilities at each SNP in the set, where the weights for the clumping PGI were set equal to the coefficient estimates from the meta-analysis and, for the COJO PGI, to the posterior effect sizes estimated by COJO. In each of the two prediction cohorts, we estimated four models with *EduYears* as the dependent variable: (i) a model with only controls (a full set of dummy variables for year of birth, an indicator variable for sex, a full set of interactions between sex and year of birth, and the first 10 principal components (PCs) of the variance-covariance matrix of the genetic data), (ii) a model with the clumping PGI and controls, (iii) a model with the COJO PGI and controls, and (iv) a model with both PGIs and controls. Using the differences in R^2 values from these models, we looked at how much predictive power (incremental-R^2) each PGI had, as well as how much additional variation they explain when fitted into a model with the other PGI and controls (**Supplementary Table 25**). When added into a model with only controls, the clumping PGI had more predictive power than the COJO PGI in HRS (COJO incremental-R^2 = 6.64%, clumping incremental-R^2 = 6.98%), and vice-versa in Add Health (COJO incremental-R^2 = 9.99%, clumping incremental-R^2 = 9.18%). Similarly, in HRS, the additional variance explained by the COJO PGI when added into a model with the clumping PGI and controls (incremental-R^2 = 0.74%) was lower than the additional variance explained by the clumping PGI when added into a model with the COJO PGI controls (incremental-R^2 = 1.08%). In Add Health, the outcome was reversed (COJO incremental-R^2 = 1.66%, clumping incremental-R^2 = 0.85%).

These results suggest there could be two forces at play that partly offset each other:

1. Within a given locus, COJO can identify secondary associations (whereas clumping drops all SNPs that are in LD with the top SNP). For this reason, the COJO-based predictor may have greater out-of-sample predictive power.
2. Genome-wide, in our data, the clumping algorithm identifies a greater number of lead SNPs than COJO at a fixed threshold of genome-wide significance (probably because, in our clumping definition of a lead SNP, LD with other SNPs could cause relatively weakly associated SNPs to cross the threshold of genome-wide significance, as mentioned above). Because EA is a highly polygenic phenotype, including more SNPs in the PGI tends to make it more predictive.

This reasoning implies that if we held the total number of SNPs constant in the comparison, COJO should do better. To test this hypothesis, we conducted a second analysis using a clumping PGI made with a stricter P value threshold ($P < 4.61 \times 10^{-9}$) so that the number of SNPs in the PGI equals the number of SNPs in the COJO PGI. We estimated the same quantities using the COJO PGI and the new clumping PGI. As predicted, we found that the COJO PGI had higher predictive power than the new clumping PGI in both cohorts when added into a model with only controls (HRS incremental-R^2 = 6.31%, Add Health incremental-R^2 = 8.35%). Moreover, in both cohorts, the additional variance explained by the COJO PGI when added into a model with the new clumping PGI and controls (HRS incremental$-R^2$ = 1.07%, Add Health incremental-R^2 = 2.20%) was higher than the additional variance explained by the new clumping PGI when added into a model with the COJO PGI and controls (HRS incremental-R^2 = 0.74%, Add Health incremental-R^2 = 0.55%).

When the LD reference sample used in the COJO analysis is small or noisy, the posterior effect sizes estimated by COJO can be less predictive than the original GWAS effect sizes. To assess whether this affects our results, we reran all analyses using a COJO PGI made using the same set of COJO lead SNPs, but using GWAS effect sizes. The conclusions qualitatively stayed the same, with the incremental-R^2's for the new COJO PGI being slightly lower in all models (except for the incremental-R^2 of adding the COJO PGI to a model with only controls in HRS).

Our findings support the conclusion that in our results, there is more information per SNP in the COJO lead SNPs compared to the clumping lead SNPs. For this reason, and because COJO is more likely to identify causal SNPs by virtue of identifying conditional associations, COJO appears to be a more suitable methodology for defining lead SNPs, at least when GWAS sample sizes are very large. However, it is important to note that both COJO and clumping lead SNPs capture information that is not captured by the other methodology: the regression coefficients for both PGIs remain statistically distinguishable from 0 when they are simultaneously fitted into a model with controls, whether or not we include the same number of SNPs in the PGIs (**Supplementary Table 26**). In the current study, we adopt clumping as our main lead SNP definition in order to be report a number of lead SNPs that is comparable with the previously published EA GWASs, but we also report the COJO lead SNPs.

2.3 Replication of EA3 lead SNPs

Lee et al.[2] reported a replication analysis, using the new data in EA3 beyond that in EA2, of the 162 genome-wide-significant lead SNPs from EA2. Here, using the new data in EA4 beyond that in EA3, we repeat this analysis for the lead SNPs from EA3,

but with a few differences. Instead of attempting to replicate the 1,271 genome-wide significant lead SNPs from EA3, we re-run the EA3 meta-analysis by replacing the UKB GWAS in that analysis with our updated UKB GWAS that uses the new phenotype coding explained in **Supplementary Note** section 1. This way, we ensure that the only difference between the (updated) EA3 meta-analysis and the current EA4 meta-analysis is the additional individuals from the 23andMe cohort, which we will use for this replication exercise.

We start by getting the list of genome-wide significant lead SNPs from the updated EA3 meta-analysis using our main clumping algorithm described in **Supplementary Note** section 2.2.6. The algorithm results in 1,572 SNPs, 1,571 of which are available in our current meta-analysis with matching alleles[10]. We focus on 1,451 of these SNPs, for which we have additional individuals from 23andMe in the current meta-analysis. To examine their out-of-sample replicability, we calculated Z-statistics from the subsample of our data that was not included in the updated EA3 meta-analysis (N_{max} = 2,272,216[11]). Let the Z-statistics of association from, respectively, (updated) EA3, the new data, and our current EA4 meta-analysis, be denoted by Z_{EA3}, Z_{new} and Z_{EA4}. Note that we cannot calculate Z_{new} directly because the 23andMe sample included in the EA3 meta-analysis ($N = 365{,}538$) is a subset of the 23andMe sample in EA4, and we do not have summary statistics from association analyses conducted only in subjects that contributed to the second but not the first meta-analysis. However, because our current EA4 meta-analysis uses sample-size weighting, we can calculate Z_{new} indirectly from the following approximation

$$Z_{EA4} \approx Z_{EA3}\sqrt{\frac{N_{EA3}}{N_{EA4}}} + Z_{new}\sqrt{\frac{N_{new}}{N_{EA4}}}$$

where SNP subscripts have been dropped for notational convenience and N's are sample sizes. We used the unadjusted $EA3$ and $EA4$ summary statistics to compute Z_{new} with this approximation (since the approximation does not hold with adjusted summary statistics), and subsequently adjusted the resulting new summary statistics by inflating their standard errors by the square root of the LD score regression (estimated in the new summary statistics). In the replication below, we use these adjusted new summary statistics. We note that the Z_{new} defined here is not necessarily equal to the Z statistic of association calculated directly in the new data (e.g., if the new 23andMe data contains individuals related to individuals in the earlier 23andMe data), but it captures the signal from the independent component of that sample and therefore is the correct value to use in this replication analysis.[2]

Of the 1,451 SNPs that we focus on, 1,447 have matching signs in the new data. For the remaining 4 SNPs, the estimated effect is statistically significant at $P < 0.1$ only for one. Of the 1,447 SNPs with matching signs, 1,343 are significant at $P < 0.01$, 1,005 are significant at $P < 10^{-5}$, and 687 are significant at $P < 5 \times 10^{-8}$. The replication results are shown graphically in **Extended Data Figure 3**.

---

[10] One SNP, rs12748397, is available in the 23andMe GWAS included in EA3 but not in the current 23andMe GWAS. Therefore, in the current EA4 meta-analysis, its sample size is below the minimum sample-size threshold of 500,000, and it gets filtered out.

[11] The maximum sample size for the new data is larger than $N_{max,EA4} - N_{max,EA3}$ = 1,905,618 because some SNPs are included in the current 23andMe summary statistics but not in the EA3 23andMe summary statistics. For those SNPs, the maximum sample size is equal to the sample size of the current 23andMe summary statistics, which is 2,272,216.

These numbers understate the replication record because there is substantial variation in the sample size of the new data, N_{new}, across the 1,451 SNPs $\left(N_{new,min} = 345{,}532\,, N_{new,max} = 2{,}272{,}216\right)$, so some SNPs replicate less strongly due to low power in the replication sample. To enforce more similar sample sizes across SNPs, we supplement the above analysis with one where we applied the following sample-size filters to the EA3 GWAS prior to clumping: $N_{EA3} > 0.8 \times N_{EA3,\max}$ and $N_{new} > 0.8 \times N_{new,\max}$. Applying our clumping algorithm to the filtered EA3 GWAS leaves us with a set of 1,504 genome-wide-significant SNPs (compared with the 1,572 above), all of which are available in the new data with matching alleles[12]. Of the 1,504 SNPs, 1,502 have matching signs in the new data. None of the 2 SNPs with non-matching signs are significant at $P < 0.1$. Of the 1,502 SNPs that have matching signs, 1,409 are significant at $P < 0.01$, 1,071 are significant at $P < 10^{-5}$, and 746 are significant at $P < 5 \times 10^{-8}$.

This replication record is strong. Under the null hypothesis that all the variation in the EA3 estimates of the additive effect sizes α_j is due to noise (or confounding due to population stratification and other sources of bias that are not shared between the EA3 data and the new data), only 50% of the SNPs would have concordant signs, and 0.5%, 0.0005%, and 0.00000025% would have concordant signs and be significant at the 0.01, 10^{-5}, and 5×10^{-8} levels, respectively. That null hypothesis is strongly rejected.

To benchmark the replication results, we conducted simulations under the assumption that the true additive effect sizes α_j are identical across the EA3 data and the new data. The replication simulation framework is described in detail in **Supplementary Note** section 8 and is similar to that used in Supplementary Note section 4 of the Supplementary Information of Karlsson Linnér *et al.* (2019)[28]. Following the simulation framework, we first estimated the parameters π and τ^2 of the prior distribution of the additive effect sizes α_j. (See **Supplementary Note** section 8 for a description of our empirical Bayesian model of that posterior distribution.) As in **Supplementary Note** section 2.2.6, we used Maximum Likelihood Estimation (MLE) to fit the distribution to the unadjusted summary statistics (see **Supplementary Note** section 8.5) for all 10,675,380 SNPs that passed our quality control filters. Our estimates are $(\hat{\pi}, \hat{\tau}^2) = (0.60, 3.277 \times 10^{-6})$. We then used the spike-and-slab prior distribution, treating these estimates as the true parameter values governing the prior, to calculate the posterior distribution of each SNP's true effect size. Finally, we simulated the replication by drawing from the SNPs' posterior distributions (**Supplementary Note** section 8 provides additional details). The following table shows the simulated expected replication record and its standard deviation across simulations, together with the results of the actual replications:

Expected and Actual Replication Results

	Without sample size filters ($M = 1{,}451$ matched SNPs with new data)	With sample size filters ($M = 1{,}504$ matched SNPs with new data)

[12] The 1,504 SNPs are not a subset of the 1,572 genome-wide-significant SNPs above because we clumped the summary statistics (to identify the new set of genome-wide-significant SNPs) after applying the new sample-size filters. All of the new genome-wide-significant SNPs are available in the new data because both sample-size filters (in particular, the filter $N_{new} > 0.8 \times N_{new,\max}$, which implies availability in the new data) were applied to the EA3 SNPs prior to clumping.

Test	Simulated replication	Actual replication	Simulated replication	Actual replication
Sign concordance	1,450.48 (0.71)	1,447	1,503.90 (0.32)	1,502
Sign conc. + $P < 0.01$	1,403.80 (6.38)	1,343	1,468.48 (6.02)	1,409
Sign conc. + $P < 10^{-5}$	1,091.84 (15.20)	1,005	1,155.92 (15.75)	1,071
Sign conc. + $P < 5 \times 10^{-8}$	743.58 (16.39)	687	794.59 (17.70)	746

Note: the actual and simulated replication records for each test are expressed as the numbers of SNPs that passed (or that are predicted to pass) the test; the standard deviation of the simulated replication record is shown in parentheses.

Overall, the actual replication results are similar to the predictions from our simulations but not quite as good. The lower-than-expected replication record could point to a positive (but likely low) false-discovery rate in EA3, but there are a number of other explanations that we think are likely. Most simply, the true additive effect sizes α_j are likely not identical across the EA3 data and the new data. Indeed, the genetic correlation between our estimates in the EA3 data and in the new data is $\hat{\rho}_g = 0.94$ ($SE = 0.01$), which is statistically distinguishable from unity. In addition, we think it is likely that our assumed spike-and-slab distribution does not yield a sufficiently good approximation to the SNPs' true effect-size distribution, now that the GWAS sample size is so large.

Another possibility, which we examine here, is that our simulation is sensitive to input model parameters that are not separately identified very precisely. To evaluate this, we reran the simulations for various assumed values of the parameter π (which captures the fraction of SNPs that are nonnull SNPs), while keeping the overall amount of variation in the SNPs' true effect sizes constant. (Specifically, we kept the product of π and τ^2 – which is equal to the overall amount of variation – constant at the value implied by the estimates we used for the above simulations: $\pi \cdot \tau^2 = \hat{\pi} \cdot \hat{\tau}^2 = 0.60 \cdot 3.277 \times 10^{-6} = 1.966 \times 10^{-6}$.) We note that the estimate of π tends to vary across different sets of summary statistics: for instance, in the summary statistics from the updated EA3 meta-analysis, $\hat{\pi} = 0.49$, while $\hat{\pi} = 0.69$ in the summary statistics from the current paper restricted to the set of SNPs with MAF no less than 0.01 (and, as mentioned above, $\hat{\pi} = 0.60$ in the summary statistics from the current paper for all SNPs).

The following table shows the results for $\pi \in \{0.25, 0.50, 0.75\}$. As can be seen, the simulated replication results vary somewhat as a function of the assumed values of π. If $\pi = 0.25$, the actual replication fares less well than what the simulation predicts. If $\pi = 0.75$, however, the replication fares about just as well as the simulation's prediction on the test of whether the lead SNPs have matching signs and P values less than 10^{-5} in the new data, and better than the simulation's prediction on the test of whether the lead SNPs have matching signs and are genome-wide significant in the new data. (On the other hand, on the sign concordance test, the actual replication consistently fares less well than the simulated prediction.) Overall, our assessment is that the replication record is strong regardless of which particular parameter values are

assumed, but the benchmarking of just how strong is sensitive to assumptions about genetic architecture that cannot be assessed with the current data.

Sensitivity of Expected Replication Results to Assumed Fraction of Nonnull SNPs

	Without sample size filters ($M = 1{,}451$ matched SNPs with new data)			
Test	Actual replication	Simulated replication With $\pi = 0.25$	Simulated replication With $\pi = 0.50$	Simulated replication With $\pi = 0.75$
Sign concordance	1,447	1,450.78 (0.47)	1,450.59 (0.64)	1,450.33 (0.80)
Sign conc. + $P < 0.01$	1,343	1,429.63 (3.95)	1,412.86 (5.47)	1,387.74 (6.94)
Sign conc. + $P < 10^{-5}$	1,005	1,259.78 (11.47)	1,143.90 (14.74)	1,012.78 (16.45)
Sign conc. + $P < 5 \times 10^{-8}$	687	999.27 (15.81)	814.37 (17.20)	645.00 (16.98)

Note: the actual and simulated replication records for each test are expressed as the numbers of SNPs that passed (or that are predicted to pass) the test; the standard deviation of the simulated replication record is shown in parentheses.

3 X chromosome analyses

Our primary GWAS meta-analysis of additive variation in *EduYears* was restricted to autosomal SNPs. Following the same analysis plan as in EA3[2], we conducted a separate association analysis of the X chromosome SNPs in our largest two cohorts: UKB and 23andMe, increasing the sample size from $N = 694{,}894$ to $N = 2{,}713{,}033$. Here, we report results from this association analysis as well as a number of supplemental analyses that analyze the contribution of common SNPs in the X chromosome to additive variation in *EduYears*.

In this section, we follow the notation and framework established in the EA3 Supplementary Section 4.1. We start by describing the association analyses conducted in UKB. Next, we report the amount of dosage compensation and male-female genetic correlation that we estimate using the sex-stratified UKB results. Finally, we report the results from a meta-analysis of mixed-sex association analyses conducted in UKB and 23andMe using identical allele coding.

3.1 UK Biobank association analyses

3.1.1 Sex-stratified association analyses in UKB

We conducted new sex-stratified association analyses of the X chromosome in UKB that used the new *EduYears* phenotype coding described in **Supplementary Note** section 1 and Panel **B** of **Supplementary Table 15**. Our new X chromosome analyses in UKB also differ from the analyses in EA3[2] in the following steps:

1. We changed some of the control variables to match what we did in our autosomal UKB association analysis. Specifically, as in our autosomal UKB association analysis, we residualized *EduYears* on the first 40 PCs provided by UKB (instead of the first 10 PCs that we constructed ourselves as in EA3[2]) and a third-degree polynomial in birth year (instead of indicator variables for birth year). As in both our autosomal UKB association analysis and EA3, we also residualized *EduYears* on indicator variables for genotype-measurement batch.
2. Instead of imputing the X chromosome genotypes ourselves, we used the imputed genotype dosages officially released by UKB that were not available at the time EA3[2] was conducted.
3. Instead of restricting the sample to unrelated individuals, we included all individuals of European genetic ancestries after filtering out individuals who have withdrawn their consent for their data to be used, heterozygosity missingness outliers and individuals with sex/gender mismatch or putative sex chromosome aneuploidy. We identified individuals of European genetic ancestries as individuals who self-report to be White, British, Irish, or of any other white background and whose loading on the first PC of the genotype data (in the official UKB release) was greater than 0.
4. To account for relatedness, we ran the association analysis in BOLT-LMM v2.3.4[29] (instead of Plink1.9[22]) using genotype dosages (instead of hard calls). As model SNPs (an input in BOLT-LMM), we used 548,860 hard-called HapMap3 SNPs that remained after filtering for MAF > 1% and pruning with an r^2 threshold of 0.9.

In our sex-stratified association analyses, ($N_{males} = 201{,}456, N_{females} = 239{,}361$; larger samples than in EA3 because, as noted above, we did not restrict the sample to unrelated individuals), we obtained association results for 1,165,370 biallelic SNPs with imputation accuracy > 0.3 and MAF > 0.0001 in the non-pseudoautosomal region of the X chromosome.

Let β_i denote the coefficient from the population regression of the phenotype on x_i, where $i \in \{m, f\}$ indicates males or females, and x_i is the allele count with $x_m \in \{0,1\}$ and $x_f \in \{0,1,2\}$. Dosage compensation can be parameterized as $\beta_m = d\beta_f$, with $1 \leq d \leq 2$, where $d = 1$ in the absence of dosage compensation and $d = 2$ under full dosage compensation. Under Hardy-Weinberg equilibrium, the variance contributed by a SNP is $d^2 p(1-p)\beta_f^2$ in males, where p is the minor allele frequency of the SNP, and $2p(1-p)\beta_f^2$ in females.

Under the assumption that $\beta_m = d\beta_f$ across all X-chromosome SNPs with a common value of d, we can estimate d using sex-stratified association results. Let $\gamma \equiv \frac{h_m^2}{h_f^2} = \frac{d^2}{2}$ be the dosage compensation (DC) ratio, where h_i^2 is the SNP heritability for the X chromosome. Then

$$\hat{\gamma} = \frac{(\hat{\chi}_m^2 - 1)N_f}{(\hat{\chi}_f^2 - 1)N_m}$$

where $i \in \{m, f\}$ indicates males or females, $\hat{\chi}_i^2$ is the mean χ^2 statistic across SNPs in the association analysis, and N_i is the sample size (see EA3 Supplementary Section 4.1[2] for a full derivation)[30]. The ratio takes on a value between 0.5 (zero DC) and 2 (full DC). For SNPs with MAF > 1%, we estimated a ratio of 0.78 (S.E. = 0.10)[13], which implies $d = 1.25$. This DC ratio estimate is somewhat smaller than what we found in EA3[2] (DC ratio = 1.05), but is contained in the 95% confidence interval (0.66 to 1.44).

Next, we used results from the sex-stratified association analyses to test the hypothesis that the male-female genetic correlation on the X chromosome is unity. The male-female genetic correlation on the X chromosome can be calculated as

$$\hat{r}_g = \frac{\widehat{Z_m Z_f}}{\sqrt{(\hat{\chi}_f^2 - 1)(\hat{\chi}_m^2 - 1)}} \quad (3.1)$$

where $\widehat{Z_m Z_f}$ is the mean of the product of the Z-statistics from the female and male analyses (see EA3 Supplementary Section 4.1[2] for the derivation). The standard errors

[13] Assuming that the individual association test statistics are distributed as a non-central χ^2 with expected value given by $1 + \frac{N_i h_i^2}{M_{eff}}$ where $i \in \{m, f\}$ and M_{eff} is the effective number of loci (which is assumed to be the same in males and females) and variance $2\left(1 + \frac{h_i^2 N_i}{M_{eff}}\right)$, the variance of the mean test statistic across the chromosome is approximately $\frac{2}{M_{eff}}[1 + 2(\hat{\chi}^2 - 1)]$, and the variance of the dosage compensation ratio is approximately $\gamma^2 \left[\frac{Var(\hat{\chi}_m^2)}{(\hat{\chi}_m^2 - 1)^2} + \frac{Var(\hat{\chi}_f^2)}{(\hat{\chi}_f^2 - 1)^2}\right]$. The M_{eff} value used here was 1,300. See EA3 Supplementary Section 4.3 for how this value was estimated.

were calculated using a block jackknife procedure with $B = 1000$ blocks of contiguous SNPs across the X chromosome using the formula

$$SE(\hat{r}_g) = \sqrt{\frac{B-1}{B}\sum_{k=1}^{B}\left(\hat{r}_g - r_g^{(k)}\right)^2},$$

where for each block k, $r_g^{(k)}$ is calculated as an estimate of the genetic correlation as in equation (3.1) using all SNPs except for those included in the k-th block. For SNPs with MAF > 1%, we estimated a male-female genetic correlation close to, but statistically distinguishable from, unity ($r_g = 0.94$; $SE = 0.03$), and smaller than what we found in EA3[2] ($r_g^{EA3} = 1.01$; $SE = 0.05$).

3.1.2 Meta-analysis of sex-stratified association results in UKB

In a joint analysis of males and females, the phenotype is regressed on a genotype variable equal to $x_f \in \{0,1,2\}$ for females and cx_m for males, with $x_m \in \{0,1\}$ and $c \in [1,2]$, where $c = 1$ corresponds to the zero-DC analysis, $c = 2$ to the full-DC analysis, and c between 1 and 2 to a partial-DC analysis. (Note that d, defined above, denotes the true amount of DC, whereas c, defined here, denotes the amount of DC assumed in the empirical analysis.) Under the simplifying assumption that the residual variance is exactly one in both males and females, the coefficient from a joint analysis can be written as

$$b_{joint} = \frac{\frac{cb_m}{Var(b_m)} + \frac{b_f}{Var(b_f)}}{\frac{c^2}{Var(b_m)} + \frac{1}{Var(b_f)}},$$

where b_m and b_f are regression coefficients that would be estimated in separate male and female association analyses, respectively, if these analyses were conducted separately. In EA3 Supplementary Section 4.1[2], we show that (i) in a joint analysis, this estimator is unbiased when c equals the true dosage compensation parameter d and (ii) an optimally weighted meta-analysis of association results from sex-stratified analyses will weight the sex-specific estimates as in this equation, setting $c = d$. That is, the joint analysis with $c = d$ is optimal in the sense that the resulting estimator has the lowest variance among the class of unbiased estimators.

We conducted two inverse-variance-weighted meta-analyses ($N = 440{,}817$) for 1,165,370 SNPs on the X chromosome, one assuming zero DC ($c = 1$) and the other assuming full DC ($c = 2$). The mean χ^2 statistic under full DC was smaller under all MAF cutoffs (see **Supplementary Table 17**) but the differences were small. Note that the estimator defined above is unbiased only when $c = d$. Therefore, this result is to be expected as the value of d estimated in our data is 1.25, closer to a model of zero DC than full DC.

Supplementary Table 17 provides a summary overview of the results from the sex-specific and joint (no DC and full DC) analyses for all SNPs with allele frequency above 1%, 0.1% or 0.01%.

3.2 23andMe association analysis

We obtained summary statistics from joint male-female association analyses of 346,055 SNPs on the X chromosome conducted in a sample of 23andMe customers ($N = 2{,}272{,}216$). These analyses were conducted using a 0/2 genotype coding for males, corresponding to a full-DC analysis. All other aspects of the analysis were identical to those of the autosomal analyses (see **Supplementary Note** section 2 and **Supplementary Table 15** for details on genotyping, imputation, phenotype coding and the association model), except for some quality control steps as described in the next section.

3.3 Quality control of UK Biobank and 23andMe results

In all the analyses that follow in the remainder of this section, we use the UKB male-female meta-analysis assuming full DC, since this was the genotype coding used by 23andMe.

We applied the quality control pipeline described in **Supplementary Note** section 2.2.5 to the UKB and 23andMe association results, with the following differences:

- We were unable to plot the reported standard errors against a correct analytical approximation for the standard errors because we did not have access to sex-specific per-SNP sample sizes in 23andMe. (Unlike in the autosomal analyses, here sex-specific sample sizes are necessary for the calculation of a correct analytical approximation to the standard error of a SNP's coefficient because the predicted standard error for males differs from that for females (and autosomal SNPs) and is a function of the assumed amount of dosage compensation.) Instead, we plotted the reported standard errors against values obtained using the analytical approximation that would be correct for autosomes (see **Supplementary Note** section 2.2.5). We identified no outliers upon visual inspection in either set of results.
- For the same reason, we did not apply the explained variance (R^2) filter (see Supplementary Section 1.7 in Okbay et al.[1]).
- We applied a stricter filter for the Hardy-Weinberg equilibrium exact test P value ($P > 10^{-10}$) (i.e., we drop all SNPs with P-values less than 10^{-10}, rather than 10^{-20} as used for the autosomes) because the P-values were calculated in the sample of females (with about half the sample size) rather than the whole sample.
- We dropped SNPs with male-female allele frequency differences above 0.005 in UKB.

After applying all filters, association results for 228,999 markers in 23andMe and 333,063 markers in UKB remained. **Supplementary Table 16** summarizes the number of markers dropped in each filtering step.

3.4 Meta-analysis of UK Biobank and 23andMe results ($N = 2{,}713{,}033$)

We meta-analyzed the summary statistics from UKB and 23andMe using sample-size weighting in METAL[18]. After applying a sample size filter of $500{,}000$ and restricting

the set of SNPs to those that were available in both UKB and 23andMe, we were left with meta-analysis results for 211,581 SNPs. To adjust test statistics for bias due to uncontrolled-for population stratification, we inflated the standard errors by the square-root of the LD score intercept from an autosomal meta-analysis of UKB and 23andMe $(\sqrt{1.666})$. The mean χ^2 test statistic, calculated prior to the LD score intercept adjustment, is 7.38. As in autosomes, the inflation of test-statistics varies by allele frequency: For common variants (defined as variants with MAF > 5%), the mean χ^2 statistic is 10.36, for low frequency variants (1% < MAF < 5%) it is 3.92, and for rare variants (MAF < 1%), we have mean χ^2 = 2.06. **Extended Data Figure 4** shows Manhattan and quantile-quantile plots from the meta-analysis.

We selected the set of approximately independent genome-wide-significant SNPs using the clumping algorithm described in **Supplementary Note** section 2.2.6. We calculated the LD between SNPs using 440,217 variants and 13,182 individuals from the public release of the HRC reference panel[16] that remained after restricting the sample to the set of individuals in the clumping reference data that we used for the autosomes[14] (see **Supplementary Note** section 2.2.6) and applying the same SNP-level filters, the only difference being that we estimated the Hardy-Weinberg equilibrium exact test *P* values for SNPs on the X chromosome in the sample of females. The algorithm identified 57 approximately independent SNPs at genome-wide significance. **Supplementary Table 27** lists the association results for these SNPs.

To gauge the magnitude of per-allele SNP effects, we used an approximation to transform the *Z*-statistics from the sample-size-weighted meta-analysis (the output of the software METAL) into unstandardized regression coefficients:

$$\widehat{\beta_j} = Z_j \frac{\hat{\sigma}_y}{\sqrt{2MAF_j\,(1 - MAF_j)(2\,N_{j,m} + N_{j,f})}}$$

for SNP j with minor allele frequency MAF_j, sample size $N_{j,m}$ in males and $N_{j,f}$ in females, Z-statistic Z_j, and standard deviation of the phenotype $\hat{\sigma}_y$. Note that this is the same approximation that we used in our additive autosomal meta-analysis except for the denominator, which now reflects the standard deviation of the genotype in an X-chromosome analysis with males coded as 0/2.

The estimated effects (in absolute value) of the 57 lead SNPs are all in the range 0.005-0.016 SD units per reference allele, corresponding to approximately 0.9 to 2.8 weeks of schooling (assuming the standard deviation of *EduYears* is 3.3, i.e. equal to the sample-size-weighted standard deviation of *EduYears* in 23andMe and UKB). Across the 57 lead SNPs, an additional copy of the trait-increasing allele is on average associated with 0.008 SD units. The minor allele frequency of the SNP with the largest effect size is 0.06.

3.5 Comparison to autosomes

Following EA3[2], we compared our autosomal and X-chromosomal meta-analyses in terms of number of lead SNPs and heritability due to common SNPs. To make results

[14] The resulting sample size for the X chromosome ($N = 13{,}182$) is lower than that of the HRC reference data that we used for the autosomes ($N = 14{,}097$), even though we restricted the sample to the same set of individuals, because the sample size for the X chromosome was smaller than that for autosomes prior to all filters.

from the two meta-analyses comparable in terms of sample size, we started by running a new autosomal meta-analysis restricted to 23andMe and UKB ($N = 2{,}713{,}337$). We subsequently applied our clumping algorithm (see **Supplementary Note** section 2.2.6) to these results to obtain the number of lead SNPs on each chromosome. For the purposes of this comparison, we used X-chromosomal and autosomal test statistics that are not adjusted for stratification biases.

The results are shown in **Supplementary Table 28** and **Supplementary Figure 4**. In terms of chromosome length, our X chromosome analysis, spanning a total distance of 152 Mb, is most similar to chromosomes 6 through 10 (mean 150, range 135 to 171). However, consistent with EA3[2], we identified less than half as many lead SNPs on the X chromosome (173 lead SNPs) as on those autosomes of similar length (average 480 lead SNPs, range 419 to 596).

One possible factor contributing to the discrepancy is that our meta-analysis of SNPs on the X chromosome likely used suboptimal weights since we estimated a dosage compensation parameter of $d = 1.25$ in UKB, whereas we conducted our meta-analysis assuming full dosage compensation (in order to match what was assumed in the 23andMe association analysis). However, as we show in EA3, the suboptimal weighting has minimal impact on the power of the meta-analysis. The power of the meta-analysis we conducted in our sample of $N = 2{,}713{,}033$ is equal to an optimally weighted meta-analysis conducted in a sample of $N = 2{,}570{,}242$ individuals. The fact that this difference is small implies that the sub-optimality of our weighting scheme contributes minimally to the observed discrepancy.

Another factor could be that the effective number of independent markers on the X chromosome could be less than on autosomes of similar length. If we compare the number of lead SNPs on the X chromosome to autosomes with a similar effective number of independent markers (see EA3 Supplementary Section 4.3[2] for a description of how the effective number of independent markers was calculated), we get a different picture. The effective number of independent markers on the X-chromosome (1,309) is similar to the numbers on chromosomes 19-22 (mean = 1,401, range 1,148 to 1,453). The average number of lead SNPs on these chromosomes is 139 (range 95 to 161) compared to 173 on the X chromosome, indicating that the X chromosome results are similar to those from autosomes with a comparable effective number of loci.

To compare the heritability due to common SNPs on the X chromosome to the per-chromosome SNP heritabilities of the autosomes, we used the following equation (see EA3 Supplementary Section 4.1 for the derivation) to estimate SNP heritabilities:

$$E[\chi^2] = 1 + \frac{Nh^2}{M_{eff}},$$

where h^2 is the SNP heritability, N is the GWAS sample size, and M_{eff} is the effective number of loci. We found that the X chromosome has a lower SNP heritability compared to autosomes of similar length, but again, the SNP heritability of the X chromosome is not an outlier if we compare it to autosomes with a similar effective number of independent markers.

Overall, our results are consistent with those of Lee et al[2].

4 Dominance GWAS

This section discusses our dominance GWAS meta-analysis. It begins by providing a brief overview of the concepts of additive and dominance genetic variance. Second, this section describes how we estimated the relevant dominance parameters in our GWAS and the methods we followed to conduct that GWAS. Third, it reports the main results of our dominance GWAS meta-analysis. Fourth, it evaluates the statistical power to estimate dominance effects in our dominance GWAS. Fifth, it reports the results of an exercise in which we decompose the variation in our estimates from our dominance GWAS. Sixth, this section concludes by describing a cross-cohort replication exercise we conducted to compare the dominance effect-size estimates from the two cohorts in our dominance GWAS (the UKB and 23andMe cohorts). Finally, the seventh and last subsection reports the results of an analysis of directional dominance (inbreeding depression) using the summary statistics from our dominance GWAS.

The main takeaway of the first six subsections, which focus on dominance rather than directional dominance, is that our results imply that there is very little dominance genetic variance for EA. Our GWAS of dominance variance identified no genome-wide-significant SNPs; the decomposition exercise shows that the bulk of the variation in our estimates of the dominance effect "d" is attributable to sampling variation rather than to true dominance signal; and the cross-cohort replication exercise suggests that the dominance GWAS estimates from one cohort do not tend to have particularly consistent signs and effects in another cohort, although that exercise does suggest some low degree of consistency compatible with a small amount of dominance variance.

This finding – that genetic dominance accounts for no more than a small share of the variance – is consistent with theoretical predictions as well as results from heritability research in laboratory animal and livestock and from twin and pedigree studies in humans (for a review, see Hill et al.[31]). It is also consistent with theoretical results that predict that a highly polygenic phenotype such as EA for which there is known inbreeding depression[32] should have little or no dominance variance[33]. That finding is also consistent with the findings of two recent papers that separately estimated that dominance SNP heritability averages only ~0.1% across dozens of phenotypes in the UKB (although neither paper studied educational attainment)[34,35].

However, two caveats should be kept in mind when interpreting these results. First, our analyses of dominance variance for EA are based principally on common SNPs. As others have pointed out (e.g., ref. [34]), for dominance the loss of information due to imperfect linkage disequilibrium between two SNPs is proportional to the fourth power of their correlation (r^4), vs. the second power of their correlation (r^2) for additive variance. Our results thus do not rule out the possibility that there are numerous rare SNPs with large dominance effect sizes but that are poorly tagged by the common SNPs we analyze.

The second caveat is that our results may in theory be sensitive to the scale of our EA variable. For example, and as is well-known, if the phenotype is an indicator (i.e., 0-1) variable, a locus may be purely additive on the underlying liability scale but still have dominance variance on the indicator scale (see, e.g., Figure 1b in ref. [36]). Thus, if our measure of educational attainment were a variable indicating whether one completed college instead of years of education, it is conceivable that we would have detected dominance variance.

As mentioned above, the seventh and last subsection reports the results of an analysis of directional dominance (inbreeding depression) for EA. We find suggestive evidence of ID for EA. Our estimate of ID implies the offspring of first cousins have on average ~1.0 fewer months of EA (P = 0.04) than the offspring of unrelated individuals.

4.1 Additive and dominance genetic variance: theoretical background

This section provides a brief overview of the concepts of additive and dominance genetic variance in the context of a single-locus model. It also discusses the implications of genetic dominance for the interpretation of SNP-effect estimates from traditional GWAS with no dominance term and shows how to partition genetic variance into additive and dominance variance components. The concepts and theoretical results as well as many of the derivations below are not novel, and readers can consult refs. [37–39] for more in-depth treatments; we follow the notation of ref. [37].

4.1.1 Genetic dominance at a single locus: background and definitions

Let AA, AT (interchangeable with TA), and TT be the three genotypes at the locus of interest. Alleles A and T have frequencies q and $p = 1 - q$ in the population. We assume Hardy-Weinberg equilibrium, so the frequencies of genotypes AA, AT, and TT are q^2, $2pq$, and p^2, respectively. We denote the allele dosage (i.e., the number of "T" alleles) by $x \in \{0,1,2\}$, and we define h as a heterozygosity indicator that is equal to 1 if $x = 1$ (and to 0 otherwise). The relationship between the phenotype y and the alleles at the locus is:

$$y = \mu + a(x - 1) + dh + \varepsilon \qquad (4.1)$$

where μ is a constant and ε is a disturbance term that is orthogonal to x and h. The parameter d is the dominance effect.

Figure 1 illustrates this relationship between the phenotype and the genotype. The additive genetic model is represented by the upward-sloping line: the population regression of y on dosage x only (i.e., without including h as a regressor). (This is the model we estimated and reported in **Supplementary Note** section 2 and what the vast majority of GWAS so far have estimated.) The regression slope α is known as the "average effect" of allele substitution[40], and the variance explained by this model is called the "additive variance." As is well-known and as shown below, $\alpha = a + (1 - 2p)d$. Thus, additive variance depends on both the a and the d parameters (and on the allele

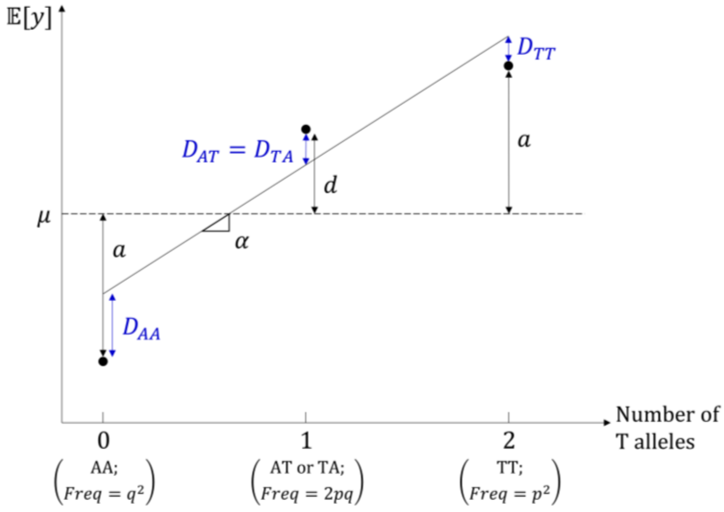

Figure 1. Genetic dominance in a single-locus model.

frequency p). By contrast, as is also shown below, dominance variance only depends on the d parameter (and on the allele frequency p). Thus, when $d = 0$, there is no dominance variance, but when $a = 0$ and $d \neq 0$ (and $p \neq 0.5$), there will be additive variance. Importantly, though these quantities are intricately linked, the a and d parameters are distinct from the additive and dominance variance.

D_{AA}, D_{AT}, and D_{TT} in Figure 1 depict the "dominance deviations" for the three genotypes, defined as the differences between the phenotypic values predicted by the additive genetic model and the actual average values at each genotype.

4.1.2 Additive GWAS

Here, we present derivations to show that the SNP effect at a given locus in an additive GWAS—what is also known as the "average effect" of allele substitution—is given by $\alpha = a + (1 - 2p)d$.

As noted above, an additive GWAS estimates the regression $y = \beta_0 + \alpha x + u$. (We ignore control variables, for simplicity.) The OLS estimator $\hat{\alpha}$ is given by

$$\begin{aligned} \hat{\alpha} &= \mathrm{Cov}(x, y)/\mathrm{Var}(x) \\ &= \mathrm{Cov}(x, \mu + a(x - 1) + dh + u)/\mathrm{Var}(x) \\ &= a + d\ \mathrm{Cov}(x, h)/\mathrm{Var}(x). \end{aligned}$$

Observe that $\mathrm{Var}(x) = 2pq$, $\mathrm{E}[x] = 2p$, $\mathrm{E}[h] = 2pq$, and $\mathrm{E}[xh] = q^2 \cdot 0 \cdot 0 + 2pq \cdot 1 \cdot 1 + p^2 \cdot 2 \cdot 0 = 2pq$. It follows that

$$\begin{aligned} \mathrm{Cov}(x, h) &= \mathrm{E}[xh] - \mathrm{E}[x]E[h] \\ &= 2pq - [2p][2pq] \\ &= 2pq(1 - 2p). \end{aligned}$$

Therefore,

$$\begin{aligned} \hat{\alpha} &= a + d \cdot 2pq(1 - 2p)/2pq \\ &= a + (1 - 2p)d. \end{aligned}$$

4.1.3 Dominance GWAS

In a dominance GWAS, the main parameter of interest is d in equation (4.1), which can be estimated directly by regressing y on $(x - 1)$ and h. (As we discuss below, in some cohorts we estimated the dominance GWAS by regressing y on x and h_{std}, where $h_{\mathrm{std}} = \frac{h - E[h]}{\sqrt{Var(h)}}$ denotes h standardized to have mean 0 and variance 1, but it is easy to recover the parameter d from such a regression.)

4.1.4 Partitioning the genetic variance

The genetic variance implied by Model (4.1) can partitioned into additive and dominance variance components. Under Model (4.1),

$$\begin{aligned} \mathrm{Var}(y) &= \mathrm{Var}(\mu + a(x - 1) + dh + \varepsilon) \\ &= \mathrm{Var}(ax + dh + \varepsilon) \\ &= a^2\ \mathrm{Var}(x) + 2ad\ \mathrm{Cov}(x, h) + d^2\ \mathrm{Var}(h) + \sigma_\varepsilon^2 \\ &= a^2 \cdot 2pq + 2ad \cdot 2pq(1 - 2p) + d^2 \cdot 2pq(1 - 2pq) + \sigma_\varepsilon^2 \\ &= 2pq[a^2 + 4(1 - 2p)ad + (1 - 2pq)d^2] + \sigma_\varepsilon^2 \\ &= 2pq[a^2 + 4(1 - 2p)ad + (1 - 2pq + q^2 + p^2 - q^2 - p^2)d^2] + \sigma_\varepsilon^2 \end{aligned}$$

$$
\begin{aligned}
&= 2pq[a^2 + 4(1-2p)ad + (q^2 - 2pq + p^2)d^2] \\
&\qquad + 2pq[1 - q^2 - p^2]d^2 + \sigma_\varepsilon^2 \\
&= 2pq[a + (1-2p)d]^2 + [2pqd]^2 + \sigma_\varepsilon^2 \\
&= 2pq\alpha^2 + [2pqd]^2 + \sigma_\varepsilon^2 \\
&= \sigma_A^2 + \sigma_D^2 + \sigma_\varepsilon^2,
\end{aligned}
$$

where $\sigma_A^2 \equiv 2pq\alpha^2$ is the additive variance and $\sigma_D^2 \equiv [2pqd]^2$ is the dominance variance. The 7th equality follows from the facts that $q^2 - 2pq + p^2 = (q-p)^2 = (1-2p)^2$ and that $1 - q^2 - p^2 = 2pq$.

Thus, as mentioned above, we see that there is no dominance variance when $d = 0$, but that there is additive variance when $d \neq 0$, even when $a = 0$ (unless $p = 0.5$).

4.2 GWAS of dominance genetic variance: methods

4.2.1 Cohorts

We meta-analyze summary statistics from two cohorts, UKB and 23andMe, with a total sample size of $N = 2{,}574{,}253$. For summary information about the cohorts, phenotypes, genotyping and imputation, see **Supplementary Note** section 2.2.

4.2.2 Association analyses

For the dominance GWAS in the 23andMe cohort, we used OLS to estimate the following regression for each SNP:

$$y = \theta_0 + ax + d_{\text{std}} h_{\text{std}} + v,$$

where $h_{\text{std}} = \frac{h - \mathrm{E}[h]}{\sqrt{\mathrm{Var}(h)}}$ denotes h standardized to have mean 0 and variance 1.

Due to computational constraints, we took a slightly different approach for the dominance GWAS in the UKB. We first used Plink 2.0[22,27] to fit the linear model

$$y = \theta_{0,UKB} + a_{UKB}x + d_{UKB}h + v,$$

where $x \in \{0, 1, 2\}$ is still allele dosage but h has not been standardized. To transform the coefficients to match those in the 23andMe GWAS of genetic dominance, we note that $h = h_{std}\sqrt{Var(h)} + E[h]$. Substituting this definition of h into the UKB regression yields

$$y = \theta_{0,UKB} + d_{UKB}\mathrm{E}[h] + a_{UKB}x + d_{UKB}\sqrt{\mathrm{Var}(h)} \cdot h_{std} + v.$$

By observation, we find that

$a = a_{UKB}$, and

$d_{\text{std}} = d_{UKB}\sqrt{\mathrm{Var}(h)}$.

Using the definitions of expectation and variance, we express $\mathrm{Var}(h)$ as a function of $\Pr(h = 1)$, which can be calculated from allele counts[15]:

$$
\begin{aligned}
\mathrm{Var}(h) &= \Pr(h=0)\cdot(0 - E[h])^2 + \Pr(h=1)\cdot(1 - E[h])^2 \\
&= [1 - \Pr(h=1)] \times \Pr(h=1)^2 + \Pr(h=1) \times [1 - \Pr(h=1)]^2.
\end{aligned}
$$

---

[15] Under Hardy-Weinberg equilibrium (HWE), $\Pr(h = 1) = 2pq$. However, we observed deviations from HWE in our data and so used the above expression for $\mathrm{Var}(h)$.

Thus, we have demonstrated equivalence between the 23andMe GWAS coefficients and the transformed UKB GWAS coefficients.

4.2.3 Quality control

We applied the same quality control pipeline described in **Supplementary Note** section 2.2.5 to the dominance GWAS results from the UKB and 23andMe (for d_{std} estimated in the 23andMe data and for the rescaled estimate $d_{UKB}\sqrt{\text{Var}(h)}$ from the UKB GWAS), but with stricter minor allele frequency (>1% instead of 0.1%) and imputation accuracy (>0.9 instead of 0.7) thresholds. We chose to be more conservative in the dominance GWAS because of the lower statistical power to detect dominance effects, especially for rare and badly imputed SNPs.

In order to apply the standard error and explained variance (R^2) filters (described in **Supplementary Note** section 2.2.5 of this paper and in Supplementary Section 1.7 of Okbay et al.[1], respectively) to the dominance GWAS results, we calculated the predicted standard errors and R^2 for the dominance effect d for each SNP j as follows:

$$SE_{d,j} \approx \frac{\hat{\sigma}_y}{\sqrt{N_j}} \times \frac{\sqrt{1-2p_jq_j}}{\sqrt{2p_jq_j}},$$

$$R^2_{d,j} \approx \frac{\hat{d}_j^2}{\hat{\sigma}_y^2} \times \frac{2p_jq_j}{(1-2p_jq_j)}$$

where $p_j = MAF_j$ is the minor allele frequency and $q_j = 1 - p_j$, $\hat{\sigma}_y^2$ is the variance of *EduYears* in the input GWAS, N_j is the GWAS sample size, and $\hat{d}_j$ is the estimated dominance effect for SNP j. (**Supplementary Note** section 8 presents derivations for the expression for $SE_{d,j}$.)

4.3 Dominance GWAS meta-analysis ($N = 2,574,253$)

We conducted a sample-size-weighted meta-analysis for 5,870,596 autosomal SNPs that passed the quality control thresholds and were available in both the 23andMe ($N =$ 2,272,216) and UKB ($N = 302{,}037$) results using the following formula:

$$Z_j = Z_{j,23andMe} \times \sqrt{\frac{N_{j,23andMe}}{N_{j,23andMe} + N_{j,UKB}}} + Z_{j,UKB} \times \sqrt{\frac{N_{j,UKB}}{N_{j,23andMe} + N_{j,UKB}}}$$

for SNP j with sample size $N_{j,i}$ and Z-statistic $Z_{j,i}$ in $i = \{UKB,\ 23andMe\}$. To gauge the magnitude of the estimated SNP effects (in units of $\hat{\sigma}_y$, the standard deviation of the phenotype), we used the approximation given above in **Supplementary Note** section 4.2.3 for the standard errors, substituted $\hat{\sigma}_y = 1$, and calculated the effect sizes as $\hat{d}_j = Z_j \cdot SE_{d,j}$.

The quantile-quantile (Q-Q) plot of the P values for the estimates of the dominance effect d from the meta-analysis (Panel **a** of **Supplementary Figure 5**) shows very little inflation ($\lambda_{GC} = 1.01$). Separately examining SNPs by MAF, there is little inflation among both common SNPs (MAF > 0.05; $\lambda_{GC} = 1.01$) and among low-frequency SNPs (0.01 ≤ MAF ≤ 0.05; $\lambda_{GC} = 1.01$) (Panel **b** of **Supplementary Figure 5**).

We did not apply genomic control to the cohort-level results prior to meta-analysis. Instead, similar to what we did in the additive GWAS, after the meta-analysis we inflated the standard errors by the square root of the intercept from an LD score regression. However, LD scores are calculated differently for the dominance effect d because of the faster decay of information from tagged SNPs as a function of LD for dominance effects (see, e.g., ref. [34]). The LD score regression was restricted to the set of HapMap3 SNPs with dominance LD scores estimated using the 1000 Genomes Phase 1 reference sample[21]. The estimates from the LD score regression suggest there is no or very little confounding and dominance genetic signal: the intercept of the LD Score regression is 1.006 (S.E. = 0.004), and the dominance heritability estimate is not statistically distinguishable from 0.

Figure 1 in the main text shows a Manhattan plot for the estimates of d. There are no genome-wide-significant SNPs, though the SNP with the smallest *P* value, rs8057808 on chromosome 16, is almost genome-wide significant, with *P* value = 6.07×10^{-8}.

These results suggest there is no or very little true dominance genetic variance for educational attainment. Consistent with this, **Supplementary Note** sections 4.5 and 4.6 below will show that the bulk of the variation in our dominance GWAS estimates is due to sampling variation rather than to true genetic dominance signal, and that the estimates from the two largest cohorts in our meta-analysis (23andMe and the UKB) do not appear to be consistent.

4.4 Statistical power to estimate dominance effects

We now evaluate our statistical power to estimate dominance effects in our dominance GWAS.

Consider the regression

$$y = \theta_0 + a_j x_j + d_j h_j + v_j,$$

where, as above, h_j is the (unstandardized) heterozygosity indicator and $x_j \in \{0, 1, 2\}$ is allele dosage. By the Frisch–Waugh–Lovell theorem, the estimate of d_j and its standard error in the above regression will be identical to those in the following regression:

$$M_x y = \theta_0' + d_j M_x h_j + v_j,$$

where $M_x = I - x_j\left(x_j' x_j\right)^{-1} x_j'$ is the orthogonal projection matrix that projects off the space spanned by x_j, and $M_x h_j$ is the residual from a regression of $h_{j,\text{std}}$ on x_j – i.e., it is the part of $h_{j,\text{std}}$ that is orthogonal to x_j. Since x_j explains no more than a minute fraction of the variation in y, we note that $M_x y \approx y$.

As we show at the end of this subsection, the R^2 of the previous regression is $\left(2p_j q_j d_j\right)^2/\text{Var}(y) = \sigma_{D,j}^2/\text{Var}(y)$, where as defined in **Supplementary Note** section 4.1 $\sigma_{D,j}^2$ is the dominance variance for SNP j. In other words, the R^2 is the share of the variance in y that is accounted for by dominance variance for SNP j.

Without loss of generality, let us work with the standarized versions of $M_x y \approx y$ and of $M_x h_j$, which we shall denote $\ddot{y}$ and $\ddot{M_x h_j}$. Let us rewrite the previous equation with the standarized variables:

$$\ddot{y} \approx \ddot{\theta}_0' + \ddot{d}_J \ddot{M_x h_J} + \ddot{v}_j.$$

The power to obtain a significant estimate of $\ddot{d}_J$ is given by

$$\begin{aligned}
\text{Power} = \text{Prob}\left(\left|\frac{\hat{\ddot{d}}_J}{\hat{\sigma}_{\hat{\ddot{d}}_J}}\right| > z_{\alpha/2}\right) &\approx \text{Prob}\left(\left|\frac{\ddot{d}_J + z\sigma_{\hat{\ddot{d}}_J}}{\hat{\sigma}_{\hat{\ddot{d}}_J}}\right| > z_{\alpha/2}\right)y \\
&\approx \text{Prob}\left(\left|\frac{\ddot{d}_J}{\hat{\sigma}_{\hat{\ddot{d}}_J}} + z\right| > z_{\alpha/2}\right) \\
&= \text{Prob}\left(\left|\sqrt{N}\ddot{d}_J + z\right| > z_{\alpha/2}\right)
\end{aligned}$$

where $z_{\alpha/2}$ is the critical value at the α level of significance; the sampling variation in $\hat{\ddot{d}}_J$ is approximately equal to $z\sigma_{\hat{\ddot{d}}_J}$, with $z \sim N(0,1)$, in sufficiently large samples by the Central Limit Theorem; and where $\sigma_{\hat{\ddot{d}}_J} = \sqrt{\text{Var}(\ddot{M_x h_J})/N} = \sqrt{1/N}$ is the sampling variance of the estimator of $\ddot{d}_J$. Because the previous regression is a univariate regression with standardized variables, $\ddot{d}_J = \sqrt{R_j^2}$, where R_j^2 is the regression's R^2. (As mentioned above, $R_j^2 = \sigma_{D,j}^2/\text{Var}(y)$ is the share of the variance in y that is accounted for by dominance variance for SNP j.) Thus, we can write

$$\begin{aligned}
\text{Power} &= \text{Prob}\left(\left|\sqrt{NR_j^2} + z\right| > z_{\alpha/2}\right) \\
&= \text{Prob}\left(\sqrt{NR_j^2} + z > z_{\alpha/2}\right) + \text{Prob}\left(\sqrt{NR_j^2} + z < -z_{\alpha/2}\right) \\
&= \left\{\Phi\left(\sqrt{NR_j^2} - z_{\alpha/2}\right)\right\} + \left\{1 - \Phi\left(\sqrt{NR_j^2} + z_{\alpha/2}\right)\right\}
\end{aligned}$$

where $\Phi(\cdot)$ is the cumulative distribution function of a standard normal variable.

Using this formula, we find that for a sample size $N = 2{,}574{,}253$ (the size of the dominance GWAS meta-analysis), we have 80% power to obtain a significant estimate at the genome-wide level of significance ($\alpha = 5 \times 10^{-8}$) of a dominance effect for a SNP with a R^2 of 1.54×10^{-5} (i.e., for a SNP for which dominance variance explains 0.00154% of the variation in y).

Proof that the R^2 of the regression $M_x y = \theta_0' + d_j M_x h_j + v_j$ is $\sigma_{D,j}^2/\mathrm{Var}(y) = (2p_j q_j d_j)^2/\mathrm{Var}(y)$.

First, observe that $\mathrm{Var}(M_x h_j) = \mathrm{Var}(h_j - \hat{\gamma} x_j) = \mathrm{Var}(h_j - (1-2p_j)x_j)$, where $\hat{\gamma} = \frac{\mathrm{Cov}(h_j, x_j)}{\mathrm{Var}(x_j)} = \frac{2p_j q_j (1-2p_j)}{2p_j q_j} = (1-2p_j)$ is the coefficient on x_j from a regression of h_j on a constant and x_j. Therefore,

$$\begin{aligned}\mathrm{Var}(M_x h_j) &= \mathrm{Var}(h_j) - 2(1-2p_j)\mathrm{Cov}(h_j, x_j) + (1-2p_j)^2 \mathrm{Var}(x_j)\\ &= 2p_j q_j (1-2p_j q_j) - 2(1-2p_j) 2p_j q_j (1-2p_j) + (1-2p_j)^2 2p_j q_j\\ &= 2p_j q_j \left[(1-2p_j q_j) - 2(1-2p_j)^2 + (1-2p_j)^2\right]\\ &= 2p_j q_j \left[1-2p_j q_j - (1-2p_j)^2\right] = 2p_j q_j \left[1-2p_j q_j - (q_j - p_j)^2\right]\\ &= 2p_j q_j [1-2p_j q_j - {q_j}^2 + 2p_j q_j - {p_j}^2] = (2p_j q_j)^2.\end{aligned}$$

Thus, the R^2 of the regression is ${d_j}^2 \mathrm{Var}(M_x h_j)/\mathrm{Var}(y) = (2p_j q_j d_j)^2/\mathrm{Var}(y)$. And per the derivations in Section 4.1, this is equal to $\sigma_{D,j}^2/\mathrm{Var}(y)$ – i.e., to the share of the variance in y that is accounted for by dominance variance for SNP j.

4.5 Decomposition of the variation in the estimates from our dominance GWAS meta-analysis

In this section, we decompose the variation in the estimated dominance effect sizes into shares due to true signal due to dominance genetic variance and to sampling variation. Specifically, we decompose the variance in $\hat{d}_{j,\mathrm{std}}$, which are standardized dominance effect-size estimates $\hat{d}_j$. $\hat{d}_{j,\mathrm{std}}$ is the estimate of the coefficient on $h_{j,\mathrm{std}}$ from a regression of y_{std} on x_j and $h_{j,\mathrm{std}}$ (and controls), where y_{std} is the standardized phenotype, $x_j \in \{0,1,2\}$ is allele dosage, $h_j \in \{0,1\}$ is the heterozygosity indicator that is equal to 1 if $x_j = 1$ (and to 0 otherwise), and $h_{j,\mathrm{std}}$ is h_j standardized to have mean 0 and variance 1. Because the reference alleles are arbitrary, $\mathrm{E}[d_{j,\mathrm{std}} | p_j, N_j] = \mathrm{E}[\hat{d}_{j,\mathrm{std}} | p_j, N_j] = 0$.

The estimated dominance effect sizes $\hat{d}_{j,\mathrm{std}}$ are equal to the true dominance effect sizes $d_{j,\mathrm{std}}$ plus an error due to sampling variation: $\hat{d}_{j,\mathrm{std}} = d_{j,\mathrm{std}} + \varepsilon\sigma_{d,j}$, where ε has mean 0 and unit variance (and is approximately normally distributed due to the Central Limit Theorem), and $\sigma_{d,j}^2$ is the variance of the estimation error of the estimate of $d_{j,\mathrm{std}}$. As we show in **Supplementary Note** section 8, $\sigma_{d,j}^2$ is equal to $\frac{1-2p_j q_j}{N_j 2p_j q_j}$, where N_j is the sample size for SNP j, p_j is the minor allele frequency of SNP j, and $q_j = 1 - p_j$.

By the Law of Total Variance, $\mathrm{Var}(\hat{d}_{j,\mathrm{std}}) = \mathrm{E}[\mathrm{Var}(\hat{d}_{j,\mathrm{std}} | p_j, N_j)] + \mathrm{Var}(\mathrm{E}[\hat{d}_{j,\mathrm{std}} | p_j, N_j])$, where the variances and expectations are taken across the SNPs.

Here $\mathrm{E}\left[\hat{d}_{j,\mathrm{std}} \middle| p_j, N_j\right] = 0$ for all p_j and N_j and $\mathrm{E}[\mathrm{Var}(\hat{d}_{j,\mathrm{std}} | p_j, N_j)] = \mathrm{E}[\mathrm{Var}(d_{j,\mathrm{std}} + \varepsilon\sigma_{d,j} | p_j, N_j)] = \mathrm{E}[\mathrm{Var}(d_{j,\mathrm{std}} | p_j, N_j)] + \mathrm{E}[\sigma_{d,j}^2] = \mathrm{Var}(d_{j,\mathrm{std}}) + \mathrm{E}[\sigma_{d,j}^2]$, where the last equality follows from applying the Law of Total Variance to $\mathrm{Var}(d_{j,\mathrm{std}})$ and from the fact that $\mathrm{E}\left[d_{j,\mathrm{std}} \middle| p_j, N_j\right] = 0$ for all p_j and N_j. Thus, $\mathrm{Var}(\hat{d}_{j,\mathrm{std}}) = \mathrm{Var}(d_{j,\mathrm{std}}) + \mathrm{E}[\sigma_{d,j}^2]$.

We do not observe $\mathrm{Var}(d_{j,\mathrm{std}})$, but we can estimate $\mathrm{Var}(\hat{d}_{j,\mathrm{std}})$ and $\mathrm{E}[\sigma_{d,j}^2]$ from the summary statistics. Thus, an estimator of the variance of the true dominance effect sizes $d_{j,\mathrm{std}}$ is given by $\widehat{\mathrm{Var}}(d_{j,\mathrm{std}}) = \widehat{\mathrm{Var}}(\hat{d}_{j,\mathrm{std}}) - \widehat{\mathrm{E}}[\sigma_{d,j}^2]$, where $\widehat{\mathrm{Var}}$ and $\widehat{\mathrm{E}}$ denote the sample variance and mean taken across the SNPs' summary statistics.

Supplementary Table 18 shows, for SNPs in various MAF bins, the quantities $\widehat{\mathrm{Var}}(\hat{d}_{j,\mathrm{std}})$, $\widehat{\mathrm{E}}[\sigma_{d,j}^2]$, as well as $\widehat{\mathrm{Var}}(d_{j,\mathrm{std}})$ expressed as a percentage of $\widehat{\mathrm{Var}}(\hat{d}_{j,\mathrm{std}})$. For this exercise, we used the unadjusted summary statistics from our overall meta-analysis of dominance variance, for all 5,870,596 SNPs that passed our quality control filters and with MAF > 0.01.

As can be seen in **Supplementary Table 18**, our calculations imply that the variance of the true dominance effect sizes ($\widehat{\mathrm{Var}}(d_{j,\mathrm{std}})$) accounts for only 0.68% of the variation in the estimated effect sizes ($\widehat{\mathrm{Var}}(\hat{d}_{j,\mathrm{std}})$) across all SNPs. In other words, the bulk of the variation in our GWAS estimates of the dominance effect size is attributable to sampling variation rather than to variation in the true dominance effect sizes across the SNPs.

We note that the estimates of $\widehat{\mathrm{Var}}(d_{j,\mathrm{std}})/\widehat{\mathrm{Var}}(\hat{d}_{j,\mathrm{std}})$ for some of the MAF bins are negative, because sampling variation can cause $\widehat{\mathrm{Var}}(\hat{d}_{j,\mathrm{std}})$ to be smaller than $\widehat{\mathrm{E}}[\sigma_{d,j}^2]$. As a (rough) gauge of the precision of our estimate of $\widehat{\mathrm{Var}}(d_{j,\mathrm{std}})/\widehat{\mathrm{Var}}(\hat{d}_{j,\mathrm{std}})$, we estimated that quantity separately for each autosome. The estimates were noisy, ranging from -7.85% on chromosome 16 to 6.22% on chromosome 8, with a mean of 0.09% and a standard deviation of 3.47%.

To benchmark these quantities related to dominance variance in educational attainment, **Supplementary Table 18** also shows analogous quantities related to additive variance: $\widehat{\mathrm{Var}}(\hat{\alpha}_{j,\mathrm{std}})$, $\widehat{\mathrm{E}}[\sigma_j^2]$, as well as the estimated variance of the true additive effect sizes $\widehat{\mathrm{Var}}(\alpha_{j,\mathrm{std}}) = \widehat{\mathrm{Var}}(\hat{\alpha}_{j,\mathrm{std}}) - \widehat{\mathrm{E}}[\sigma_j^2]$, expressed as a percentage of $\widehat{\mathrm{Var}}(\hat{\alpha}_{j,\mathrm{std}})$. (Here, σ_j^2 is the variance of the estimation error of the estimates of $\alpha_{j,\mathrm{std}}$, and as **Supplementary Note** section 8 shows, $\sigma_j^2 \approx \frac{1}{N_j}$.) For this, we used the unadjusted summary statistics from our GWAS of additive variance in the 23andMe cohort, for all 10,675,380 SNPs that passed our quality control filters and with MAF > 0.01.

In contrast to our estimates from the dominance GWAS, our estimate from the additive GWAS implies that the bulk of the variation in the estimated additive effect sizes ($\widehat{\mathrm{Var}}(\hat{\alpha}_{j,\mathrm{std}})$) is due to variation in the true additive genetic signal ($\widehat{\mathrm{Var}}(\alpha_{j,\mathrm{std}})$): we estimate that 79.77% of the variation in the estimated additive effect sizes is due to variation in the true additive effect size.

These results suggest that there is very little dominance variance for educational attainment.

4.6 Replications across our dominance GWAS of variance in the 23andMe and UKB data

Despite the relatively large size of the two cohorts in our dominance GWAS, UKB and 23andMe, we do not have sufficient statistical power to individually replicate the estimates of the SNPs with the lowest P values from either cohort in the other cohort. Instead, we conducted a series of replication exercises to assess whether the estimates of the dominance effect d for various subsets of SNPs appear to be broadly consistent across the two cohorts.

We had preregistered these replication exercises (see https://osf.io/uegqv/) in the hope that they would help demonstrate that the estimates of d are consistent across the two cohorts. In the end, however, these replication exercises mainly serve to demonstrate that the estimates are *not* particularly consistent, although there is evidence of some low degree of consistency compatible with a small amount of dominance variance. Thus, these exercises further support the conclusion that there is very little dominance variance for educational attainment.

We closely followed the methods described in the preregistration document (https://osf.io/uegqv/). We highlight below the small modifications we made to the preregistered methods, but the key result—namely that neither the signs nor the significance of the estimates of d appear to be consistent across the 23andMe and UKB cohorts—also holds if we strictly follow the methods in the preregistration document.

To assess whether the estimates of d are consistent across the 23andMe and UKB cohorts, we defined the set of "X-threshold lead SNPs" from our 23andMe GWAS as the set of approximately independent ($r^2 < 0.1$) SNPs whose estimates of d have (adjusted) P values less than X in our dominance GWAS in the 23andMe data, for a given P value threshold X.[16] In our "23andMe → UKB" replication, we assessed whether the corresponding estimates of d in the UKB data for the X-threshold lead SNPs for various thresholds X tend to have the same signs (test (i)) and tend to have the same signs and P values smaller than 0.1 (test (ii)). In our "UKB → 23andMe" replication, we repeated this exercise, this time comparing the estimates of d for the X-threshold lead SNPs from our UKB GWAS to their estimates in the 23andMe data.

We begin by describing the replication tests (i) and (ii) in more detail. We then report the results of the actual replication exercises. Finally, we describe a set of simulations we conducted to benchmark the results of replications. **Supplementary Note** section 8 describes the empirical Bayesian model we used to estimate the posterior distributions of d and which we used in our simulations.

[16] We used our main clumping algorithm, which we also used to identify the additive GWAS lead SNPs (see **Supplementary Note** section 2.2.6), to generate the X-threshold lead SNPs. The only difference is that the clumping process was iterated until no SNP with a P value less than X (instead of 5×10^{-8}) remained. Also, to maximize the number of SNPs used for these replication exercises, we applied our clumping algorithm to the set of SNPs that are present in both the UKB and the 23andMe data. We applied that clumping algorithm to the adjusted summary statistics, in which the standard errors have been inflated by the square root of the LD score regression intercept (and where modified LD scores computed for the dominance effect d were used in the LD score regression).

4.6.1 Binomial replication tests

For each of our two cross-cohort replications, we followed the framework of Karlsson Linnér *et al.* (2019)[28] and conducted two series of binomial tests of the null hypothesis that all the X-threshold lead SNPs are null dominance SNPs (i.e., $d_j = 0$ for all SNPs j). In the first series of binomial tests, we assessed for the X-threshold lead SNPs for various thresholds X whether the estimates of d_j have concordant signs in the replication GWAS (condition (i), which corresponds to test (i)). In the second series of binomial tests, we assessed for the X-threshold lead SNPs for various thresholds X whether the estimates of d_j have concordant signs and (adjusted) P values smaller than 0.1 in the replication GWAS (condition (ii), which corresponds to test (ii)). Under the null hypothesis of no dominance variance, we would expect 50% of the X-threshold lead SNPs to satisfy condition (i) and 50% × 0.1 = 5% to satisfy condition (ii).

Because the X-threshold lead SNPs are approximately independent (pairwise $r^2 < 0.1$), the number of X-threshold lead SNPs that satisfy condition (i) or (ii) can be modeled as a series of coin flips, where the probability of a "success" is 0.5 for condition (i) and 0.05 for condition (ii). Let k denote the total number of X-threshold lead SNPs satisfying conditions (i) or (ii). It follows that under the null hypothesis $k \sim \text{Binomial}(M, p)$, where M is the total number of X-threshold lead SNPs and p is the probability of condition (i) or (ii) occurring for a SNP (i.e., 0.5 or 0.05). For both binomial tests, we used one-sided tests of the null hypothesis because we are specifically interested in testing for a larger share of SNPs satisfying condition (i) or (ii) relative to what is expected under the null.

As we discuss below, our simulations show that the P-value threshold X that maximizes statistical power for either test depends on the assumed value of ω, which corresponds to the fraction of SNPs that are nonnull dominance SNPs in our empirical Bayesian model of the posterior distribution of d_j (which we describe in **Supplementary Note** section 8). Because we do not know the true value of ω, for each of our two planned cross-cohort replications we conducted tests (i) and (ii) for the X-threshold lead SNPs for the P value thresholds 1, 0.01, 10^{-4}, and 10^{-6}.

4.6.2 Replication results

Panels **A.1.i**, **A.2.i**, **B.1.i**, and **B.2.i** of **Supplementary Table 19** show the results of the replication exercises. We fail to reject the null hypothesis that there is no dominance genetic signal at the 5% level of significance in 11 of the 16 replication tests. (For both the 23andMe → UKB and the UKB → 23andMe replication, we conducted tests (i) and (ii) for four different P value thresholds, so there is a total of 16 tests.) 3 of the 5 significant replication tests were with the P-value threshold 10^{-6} and involved only a single SNP (for the UKB → 23andMe replication) or only 3 SNPs (for the 23andMe → UKB); this suggests that those SNPs may be have non-null dominance effects, although those successful results may also be the result of chance given the small number of SNPs involved. Only 2 of the 12 tests involving the P-value thresholds 1, 0.01, and 10^{-4} (and thus involving more than 100 SNPs) were significant, which is a little more than one would expect if the data only captured noise and is thus also suggestive that there some true, but very weak, dominance signal.

4.6.3 Simulation framework to estimate the expected replication record

While most of the 16 replication tests were not significant, this could in principle be due to lack of statistical power. To evaluate this possibility, we conducted simulations of the replications. For each replication, we estimated the statistical power of tests (i) and (ii) for the *P* value thresholds 1, 0.01, 10^{-4}, and 10^{-6}. Our approach and presentation are similar to those in Supplementary Note section 4 of the Supplementary Information of Karlsson Linnér *et al.* (2019)[28], with adjustments to the empirical Bayesian model for a dominance (rather than additive) GWAS.

Our simulations of the UKB → 23andMe replication assume that 1% of the variation in the estimates $\hat{d}_{j,\text{std}}$ from our UKB dominance GWAS is due to true dominance signal, and our simulations of the 23andMe → UKB replication assume that 1% of the variation in the estimates $\hat{d}_{j,\text{std}}$ from our 23andMe dominance GWAS is due to true dominance signal (and that the rest is due to sampling variation). We find that, even if true dominance signal explains that little of the variation in the estimates $\hat{d}_{j,\text{std}}$, most of our binomial replication tests should be reasonably well powered for many of the *P* value thresholds. This, together with the non-significance of many of our well-powered replication tests, suggests that less than 1% of the variation in the estimates $\hat{d}_{j,\text{std}}$ from our UKB and 23andMe dominance GWAS is due to true dominance signal. This is consistent with our above result that only 0.68% of the variation in the estimates $\hat{d}_{j,\text{std}}$ from our overall dominance GWAS meta-analysis is due to true signal.

4.6.4 Estimating κ^2

The simulations are based on an empirical Bayesian model of the distribution of d_j (see **Supplementary Note** section 8). A key parameter in our empirical Bayesian model is κ^2, which captures the variance of "nonnull dominance SNPs" (i.e., the SNPs for which $d_j \neq 0$). We began by estimating the parameter κ^2 for various assumed values of ω. ω is the fraction of SNPs that are nonnull dominance SNPs, and the estimate of κ^2 depends on ω.

As we show in **Supplementary Note** section 8, our estimator of κ^2 for a given assumed value of ω is

$$\hat{\kappa}^2 = (\widehat{\text{Var}}(\hat{d}_{j,\text{std}}) - \hat{\text{E}}[\sigma^2_{d,j}])/\omega = \widehat{\text{Var}}(d_{j,\text{std}})/\omega,$$

where the second equality follows from the derivations in **Supplementary Note** section 4.5 above.

In **Supplementary Note** section 4.5, we decomposed the variation in the estimates $\hat{d}_{j,\text{std}}$ from our dominance GWAS meta-analysis and found that variation in the true dominance effect sizes $d_{j,\text{std}}$ accounts for only 0.68% of the variation in $\hat{d}_{j,\text{std}}$, thus implying small estimates of κ^2.

For the UKB → 23andMe replication, we estimated κ^2 for various assumed values of ω using the (unadjusted; see **Supplementary Note** section 8.5) summary statistics from our GWAS of dominance variance in the UKB data for all 5,870,596 SNPs that have data in both the 23andMe and the UKB summary statistics and that passed our quality control filters. Our estimate is $\omega\hat{\kappa}^2 = \widehat{\text{Var}}(d_{j,\text{std}}) = 2.86 \times 10^{-7}$, which implies that 1.33% of the variation in $\hat{d}_{j,\text{std}}$ is due to true dominance genetic signal. For the

23andMe → UKB replication, we estimated κ^2 using the (unadjusted) summary statistics from our GWAS of dominance variance in the 23andMe data, again using the 5,870,596 SNPs that have data in both the 23andMe and the UKB summary statistics and that passed our quality control filters. Our estimate is $\omega\hat{\kappa}^2 = \widehat{\text{Var}}(d_{j,\text{std}}) = 1.42 \times 10^{-8}$, which implies that 0.48% of the variation in $\hat{d}_{j,\text{std}}$ is due to true dominance genetic signal.

We note that the estimates of κ^2 appear to be quite imprecise. To illustrate this, we estimated κ^2 separately for each autosome for the case when $\omega = 1$. In the 23andMe data the mean κ^2 across the autosomes is 5.15×10^{-9}, with a standard deviation of 1.09×10^{-7} and minimum and maximum values of -2.30×10^{-7} and 2.19×10^{-7}. In the UKB the mean κ^2 across the autosomes is 1.56×10^{-7}, with a standard deviation of 6.98×10^{-7} and minimum and maximum values of -1.50×10^{-6} and 1.63×10^{-6}.

Since our estimates of κ^2 are imprecise, we simply assumed in our simulation that 1% of the variation in the estimates $\hat{d}_{j,\text{std}}$ from our UKB dominance GWAS, as well as in the estimates $\hat{d}_{j,\text{std}}$ from our 23andMe dominance GWAS, is due to variation in the true dominance effect sizes $d_{j,\text{std}}$ (and that the rest is due to sampling variation). For our simulation of the UKB → 23andMe replication, this implies that $\omega\kappa^2 = 2.15 \times 10^{-7}$ in the UKB data; for our simulation of the 23andMe → UKB replication, this implies that $\omega\kappa^2 = 2.98 \times 10^{-8}$ in the 23andMe data.

Simulation for the UKB → 23andMe replication

We first describe our simulation of the UKB → 23andMe replication.

For each of the four P value thresholds and each assumed value of ω, we used simulations to estimate the statistical power of tests (i) and (ii), the expected replication record $\hat{E}[C_{(\text{i})}]$ and $\hat{E}[C_{(\text{ii})}]$ for tests (i) and (ii), and the standard deviations of $C_{(\text{i})}$ and $C_{(\text{ii})}$, where $C_{(\text{i})}$ and $C_{(\text{ii})}$ are the number of X-threshold lead SNPs satisfying conditions (i) and (ii) for a given threshold. To do so, we used the replication simulation framework as well as the empirical Bayesian model of the posterior distribution of d_j, both of which are described in detail in **Supplementary Note** section 8. Our estimate of the statistical power of tests (i) and (ii) is the fraction of the simulations in which the corresponding null is rejected at the 5% level. As mentioned above we assume that $\omega\kappa^2 = 2.15 \times 10^{-7}$ in the UKB data for these simulations.

Simulation for the 23andMe → UKB replication

For the 23andMe → UKB replication, we had conducted and reported the results of simulations in the preregistration document (https://osf.io/uegqv/) using an earlier estimate of $\omega\kappa^2$ in the 23andMe data. We reran those simulations following using the methodology described above for the UKB → 23andMe replication and assuming that $\omega\kappa^2 = 2.98 \times 10^{-8}$ in the 23andMe data, as mentioned above[17].

[17] Our methodology for the simulations in the preregistered document was analogous to the one we followed here, except that we had applied the clumping algorithm to all 23andMe SNPs instead of to the set of SNPs that are present in both the UKB and the 23andMe data. Furthermore, we had used an earlier estimate of $\omega\kappa^2$ in the 23andMe data, and we had not inflated the simulated standard errors in the replication data by the square root of the LD score regression intercept (see **Supplementary Note** section 8).

4.6.5 Simulation results

Panels **A.1.ii**, **A.2.ii**, **B.1.ii** and **B.2.ii** of **Supplementary Table 19** show how the statistical power of tests (i) and (ii) varies as a function of the assumed value of ω and of the P value threshold for the UKB → 23andMe and the 23andMe → UKB replication. As can be seen, power for both tests ranges from close to 0 to 100% depending on ω and the P value threshold[18].

Importantly, as can be seen in Panels A.1.ii, for replication test (i) (the sign test) for the UKB → 23andMe, for every value of ω except $\omega = 10^{-4}$, there is a P value threshold for which estimated power equals 100%; and even for $\omega = 10^{-4}$, estimated power equals 76.9% for the P value threshold $P < 10^{-6}$. In other words, under the assumption that 1% of the variation in the UKB dominance GWAS summary statistics is due to true dominance signal and as long as $\omega \geq 10^{-3}$, our simulations suggest that the null hypothesis should have been rejected with near certainty on test (i) for at least one of the P value thresholds; and if $\omega = 10^{-4}$, the null hypothesis was likely to be rejected for one of the thresholds. Consistent with this, the null hypothesis was rejected at the P value thresholds 1 and 10^{-6} (although the test with the latter threshold involved only one SNP), thus suggesting our dominance GWAS estimate captures at least some true dominance signal.

As can be seen in Panels A.2.ii, test (ii) is also well-powered for the UKB → 23andMe replication, with estimated power equal to 100% for at least one P value threshold if $\omega \geq 10^{-3}$. In fact, estimated power exceeds 80% for all values of ω for the P value threshold 10^{-4}, so that test was well-powered regardless of ω; despite this, that test was not significant ($P = 0.33$), thus suggesting that true signal accounts for less than 1% of the variation in the UKB dominance GWAS summary statistics.

As can be seen from Panels B.2.i and Panels B.2.ii, the 23andMe → UKB replication is also well-powered, albeit a bit less so than the UKB → 23andMe replication.

Panels **A.1.iii**, **A.2.iii**, **B.1.iii** and **B.2.iii** of **Supplementary Table 19** show the expected replication record for tests (i) and (ii), as a function of the assumed value of ω and of the P value threshold. For ease of interpretation, the expected replication record is expressed as the expected fraction of X-threshold lead SNPs that will pass tests (i) and (ii) (i.e, $\hat{E}[C_{(\mathrm{i})}/M]$ and $\hat{E}[C_{(\mathrm{ii})}/M]$), and the standard deviations of $C_{(\mathrm{i})}/M$ and $C_{(\mathrm{ii})}/M$ are shown in parentheses. The null hypothesis for test (i) is that $\frac{C_{(\mathrm{i})}}{M} = 0.5$, and the null hypothesis for test (ii) is that $\frac{C_{(\mathrm{ii})}}{M} = 0.05$, where M is the number of X-threshold lead SNP for the P value threshold used for the test.

In sum, our simulations of the UKB → 23andMe and 23andMe → UKB replications suggest that the replication tests were reasonably well powered, if at least 1% of the variation in the UKB and 23andMe dominance GWAS summary statistics were due to true dominance signal. Although some replication tests were significant at the 5% level of significance, other well-powered tests were not, thereby suggesting that less, or no more than, 1% of the variation in the UKB and 23andMe dominance GWAS summary statistics is due to true dominance signal.

---

[18] Also note that the power-maximizing P value threshold depends on the assumed value of ω. We had reached the same conclusion with our simulations of the 23andMe → UKB replication in the preregistration document available at https://osf.io/uegqv/, and this is the reasons why we did not select a single threshold but rather conducted tests (i) and (ii) for the P value thresholds 1, 0.01, 10^{-4}, and 10^{-6}.

4.7 Directional dominance (inbreeding depression)

This section summarizes the theory and estimation strategy devised to quantify directional dominance from summary statistics of dominance GWAS. The method described here was developed in Yengo et al. (2021)[41] and is extensively detailed in Supplementary Note 3 of their manuscript.

4.7.1 Background

The expected number of offspring an individual will contribute to the next generation is classically called Darwinian fitness, or just fitness. Fitness depends on many phenotypes, referred to as fitness components, such as infertility, stature, lung function and intelligence.

Offspring of genetically related individuals often exhibit reduced fitness[32,42,43] as a consequence of a phenomenon known as inbreeding depression (ID). ID affects most fitness components (including cognitive performance) and has been shown to be largely caused by partially recessive alleles, whose deleterious effects on fitness occur as a result of increased homozygosity due to inbreeding[39,44].

For a phenotype subject to ID whose causal alleles do not interact between different loci, theory predicts that the phenotypic value of an individual declines *linearly* with respect to the individual's inbreeding coefficient[39]. The inbreeding coefficient of an individual, classically denoted F, denotes the kinship coefficient, or one half the coefficient of relationship, between that individual's parents. For example, the offspring of two unrelated individuals has $F = 0$, while the offspring of two first-cousins has an expected inbreeding coefficient $F = 0.0625$.

Conditional on inbreeding coefficient F_i, the expected heterozygosity of an individual i at SNP j is $E[h_{ij}|F_i] = 2p_jq_j(1 - F_i)$, while the expected allele dosage $E[x_{ij}|F_i] = 2p_j$ does not depend on F_i. Therefore, for a phenotype subject to ID, y_i, controlled by M causal variants and defined as

$$y_i = \mu + \sum_{j=1}^{M} \left(a_j x_{ij} + d_j h_{ij} \right) + e_i,$$

it follows that

$$\begin{aligned} E[y_i|F_i] &= \left(\mu + \sum_{j=1}^{M} \left(a_j(2p_j) + d_j(2p_jq_j) \right) \right) - \left(\sum_{j=1}^{M} d_j(2p_jq_j) \right) F_i \\ &\equiv \mu' + bF_i. \end{aligned}$$

Consequently, a more formal definition of ID (in the population) is given by

$$b = -\left(\sum_{j=1}^{M} 2p_jq_jd_j \right).$$

This last equation predicts the phenotypic reduction expected in fully inbred individuals (F = 1) and highlights that dominance effects (d_j) — that is, the effects of heterozygosity — need to be on average positive for ID to occur. This is the reason why ID is sometimes referred to as "directional dominance".

4.7.2 *Estimation of ID from dominance GWAS summary statistics*

ID is classically estimated from individual-level data as the slope (b) of the linear regression of the phenotype of interest on F. In practice, because pedigree information is scarce, F is often estimated from SNP data using methods like runs of homozygosity[45] and the genome-wide average of the per-SNP estimate of F[23]. Among the latter, one popular SNP-based measure of F, denoted in Yengo et al. (2017)[46] as F_{UNI}, is defined for a set of M_{SNP} SNPs as:

$$F_{UNI} = \frac{1}{M_{SNP}} \sum_{k=1}^{M_{SNP}} \frac{x_k^2 - (1 + 2p_k)x_k + 2p_k^2}{2p_k(1 - p_k)}.$$

F_{UNI} has useful statistical properties such as (1) minimal variance over the class of per-SNP measures of F and (2) orthogonality to alleles counts (under Hardy-Weinberg equilibrium), which makes it more robust to population stratification[23,46] (population stratification is classically captured by genetic principal components, which are linear combinations of allele counts).

We define b_{UNI} as the regression slope obtained from regressing the phenotype of interest (y) on F_{UNI} across individuals. When the allele frequency and LD distributions of causal variants do not differ from those of SNPs used to calculate F_{UNI}, Yengo et al. (2017)[46] showed that b_{UNI} is an unbiased estimator of ID, i.e., $E[b_{UNI}|F_{UNI}] = b$.

Under Hardy-Weinberg equilibrium, Yengo et al. (2021)[41] further established a connection between estimation of ID using F_{UNI} and the summary statistics from a dominance GWAS. They did so by showing that the expectation of the Z-statistic $Z_{d,k}$ (i.e., estimated effect divided by its standard error) of the OLS estimator of the dominance effect at SNP k is given by

$$E[Z_{d,k}] = -\sqrt{N}\left(\frac{b_{UNI}}{M_{LD}}\right)\ell_k,$$

where N is the dominance GWAS sample size and ℓ_k is the standard LD score of SNP k as defined in Bulik-Sullivan et al. (2014). This equation is akin to the LD score regression model used to estimate SNP-based heritability[20]. Assuming an infinitesimal model where all SNPs are causal, M_{LD} can be chosen to be the number of SNPs for which LD scores are available. Analogous to LD score regression, b_{UNI} can estimated by regressing $Z_{d,k}$ on $-\sqrt{N}\ell_k/M$ across SNPs. These estimates of ID are expressed in phenotypic standard deviations.

We used the LDSCdom software (https://github.com/loic-yengo/LDSCdom), which implements a weighted least squares estimator of b_{UNI}. The weights are proportional to $1/\ell_k$. Standard errors of the estimated slope (b_{UNI}) and intercept are obtained using a block-jackknife procedure based on 289 ~10 Mb-long pre-defined chromosomal segments. (This number of blocks in slightly larger than the 200 blocks used in LDSC, yet still minimises the effect of long-range LD over using shorter blocks (e.g., ~2 Mb-long segments).)

4.7.3 *Analysis of summary statistics from the UKB and 23andMe*

We analyzed the unadjusted[19] dominance GWAS summary statistics from the UKB and 23andMe using two different sets of LD scores for those analyses. The first set contains LD scores of 1,290,028 SNPs with MAF > 1% calculated from the genotypes of 378 European-genetic-ancestry participants of the 1,000 Genomes Project (1KG). 1KG LD scores were downloaded from https://data.broadinstitute.org/alkesgroup/LDSCORE/. The second set contains LD scores, calculated using GCTA, for 9,326,609 SNPs with MAF > 1% imputed in 348,501 unrelated European-genetic-ancestry participants of the UKB.

We report in **Supplementary Table 20** the estimates of ID for each cohort separately as well as the inverse-variance-weighted meta-analysis of these two estimates. Overall, we found a good consistency between ID estimates obtained using either the 1KG or the UKB LD scores. Therefore, we hereafter only comment on ID estimates based on the UKB LD scores, which have smaller standard errors. Results are in units of standard deviations of EA.

We found significant evidence of ID in EA using the UKB GWAS summary statistics ($\hat{b}_{\text{UKB}} \sim$ -1.71; S.E. = 0.6; P = 0.004). However, we did not replicate that result in the 23andMe summary statistics despite a sign-consistent estimate of ID ($\hat{b}_{\text{23andMe}} \sim$ -0.24; S.E. = 0.2; P = 0.26). The smaller magnitude in the 23andMe cohort may be due to stronger ascertainment on high EA in 23andMe, which would be expected to reduce the magnitude of ID. Next, we meta-analyzed these two estimates and obtained a marginally significant combined estimate of ID, $\hat{b}_{\text{META}} \sim$ -0.41 (S.E. = 0.6; P = 0.04). Note that the Cochran's heterogeneity statistic is I^2 ~ 81.5%, which is consistent with a difference in ID estimates between UKB and 23andMe.

Our meta-analyzed estimate of ID implies a 0.41 standard-deviation reduction of EA in fully inbred individuals, relative to the population mean. Equivalently, in a population where the variance of EA is ~10 years (the sample-size weighted average variance across our two samples), the EA of offspring of first cousins (F = 0.0625) would be expected to be ~1.0 month lower ($-0.41 \times 0.0625 \times \sqrt{10} \times$ (12 months)) than that of the offspring of unrelated individuals.

4.7.4 *Reconciling evidence of directional dominance with evidence of negligible dominance variance*

Consistent with previous studies based on individual-level regression of EA on the inbreeding coefficient[32,43], we have reported evidence of directional dominance effects on EA. We note that our meta-analyzed estimate of ID is considerably smaller than those reported in those previous studies. For instance, the main estimate from Clark et al.[32] is $\hat{b} = -1.54$, which implies that a 1.54 standard-deviation reduction in EA in fully inbred individuals (vs. our meta-analyzed estimate of $\hat{b} = -0.41$). However, that difference is driven by our estimate in the 23andMe data: our UKB estimate is $\hat{b}_{\text{UKB}} \sim -1.71$, which is similar to Clark et al.'s estimate.

This evidence of directional dominance for EA supports the presence of non-zero dominance effects and thus seem to contradict our finding of negligible dominance

[19] I.e., the summary statistics were *not* adjusted by inflating the standard errors by the square root of the LD score regression intercept.

variance for EA. We reconcile these two observations by considering a simplified scenario where EA is controlled by M causal SNPs with constant dominance effects and constant allele frequencies, i.e., $d_j \equiv d$ and $p_j \equiv p$.

Under these assumptions, ID is defined as $b = 2Mpqd$, which is equivalent to $2pqd = (b/M)$. Therefore, the dominance variance σ_d^2, defined as the sum over all M causal variants of the $\left(2p_j q_j d_j\right)^2$ terms, can be expressed under our simplified assumption as $\sigma_d^2 = M(b/M)^2 = b^2/M$. Thus, σ_d^2 is expected to be non-zero yet vanishingly small (on the order of $1/M$) for highly polygenic phenotypes such as EA. This result was first shown by Robertson and Hill[33].

In conclusion, our observations are not contradictory and are likely explained by the high degree of polygenicity of EA.

5 Predicting EA and cognitive performance

In this section, we examine polygenic indexes (PGIs) derived from the autosomal GWAS of additive variation described in **Supplementary Note** section 2. We assess empirically how well they predict a host of phenotypes related to educational attainment, academic achievement, and cognition in European-genetic-ancestry holdout cohorts. We begin by describing the methodology used to generate the PGIs analyzed in this section. We continue by describing the phenotypes that we analyze in our three holdout cohorts: the Health and Retirement Study (*HRS*), the National Longitudinal Study of Adolescent to Adult Health (*Add Health*), and the Wisconsin Longitudinal Study (*WLS*). Next, we describe the control variables that we include in our analyses and define our measure of prediction accuracy. In the final subsection, we present the results.

5.1 Constructing the polygenic indexes

LDpred PGIs. The analyses in this section are based on summary statistics from a meta-analysis that excludes *HRS*, *Add Health* and *WLS*. To make fair comparisons across our holdout cohorts, we restrict the set of markers included in the PGIs to HapMap3 SNPs. We use HapMap3 SNPs because these SNPs are generally well imputed and provide good coverage in European-genetic-ancestry individuals.

Our main PGIs are derived using a Bayesian approach implemented in the software LDpred (v. 1.0.11)[47], which explicitly models genetic architecture and sets the weight for each variant equal to the mean of its posterior effect-size distribution after accounting for LD. Following EA3[2], we assume a Gaussian prior for the distribution of effect sizes. Our pipeline deviates from EA3 in the following settings:

- Rather than using cohort-specific genotype data, we estimate LD patterns in a sample of 14,028 individuals and 1,214,408 HapMap3 SNPs from the public release of the Haplotype Reference Consortium (HRC) reference panel[16] that remained after the following QC filters: (i) SNP call rate > 0.98, (ii) sample call rate > 0.98, (iii) genetic relatedness coefficient from plink1.9[22] < 0.025, and (vi) plink1.9 `--neighbor` *Z* score > -5. We opt for using the HRC data as our reference sample because using sequenced (as opposed to imputed) data would likely allow us to estimate LD patterns more accurately.
- We use the `--z-from-se` option in LDpred, which allows the program to obtain *Z* statistics from GWAS coefficients and their standard errors, as opposed to the default option of obtaining them using *P* values. We use this option because we have encountered issues with extremely low *P* values being rounded down to 0 by LDpred, making it impossible to obtain the *Z* statistics. As a result, SNPs with the strongest signal of association with the phenotype would be dropped from the analysis.
- In EA3 we applied a call rate filter of > 0.98 to the genotype data of prediction cohorts, but in this paper we do not apply any call rate filter. The reason for applying the call rate filter in EA3 was that we were using cohort-specific genotype data for the estimation of LD patterns; estimating LD using SNPs with low call rate could lead to errors. Since we use the HRC reference panel described above for LD estimation in the current study, no filters on the cohort genotype data were necessary in addition to those that are applied by default by

LDpred (MAF > 0.01 and the allele frequency discrepancy between the meta-analysis and the prediction cohort does not exceed 0.15).

In the *Add Health* data imputed to the HRC reference panel[16], there are a total of 1,211,662 HapMap3 SNPs. Of these, 1,201,142 are available in both the GWAS summary statistics and the HRC reference panel that we use to estimate LD patterns. 6,231 SNPs are dropped by LDpred due to mismatching nucleotides, 24,126 due to low MAF, and 110 due to allele frequency discrepancy with the GWAS summary statistics, leaving 1,194,911 SNPs that are used to construct the *Add Health* PGI.

The *HRS* and *WLS* data are imputed to the 1000 Genomes Phase 3 reference panel[21]. In *HRS*, there are 1,216,798 HapMap3 SNPs. Of these, 1,202,197 are available in the GWAS summary statistics and the HRC reference panel. 40,780 SNPs are dropped by LDpred due to mismatching nucleotides, 23,843 due to the low MAF, and 94 due to allele frequency discrepancy with the GWAS summary statistics, leaving 1,161,417 SNPs that are used to construct the *HRS* PGI. In WLS, there are 1,209,437 HapMap3 SNPs, 1,197,043 of which are common across all three data sets. 7,283 SNPs get filtered out due to mismatching nucleotides, 23,946 due to low MAF, and 84 due to MAF discrepancy. The remaining 1,189,760 SNPs are used to construct the *WLS* PGI.

We obtain the PGIs for European-genetic-ancestry individuals in Plink2[27] by multiplying the genotype probabilities at each SNP by the corresponding estimated posterior mean calculated by LDpred, and then summing over all included SNPs. We identify European-genetic-ancestry individuals based on the first four principal components of the genetic data of each cohort. In order to obtain the principal components, for each cohort, we first convert the imputed genotype probabilities for HapMap3 SNPs into hard calls. Then, we merge the data with all samples from the third phase of the 1000 Genomes Project[21], restricting to SNPs that had a call rate greater than 99% and MAF greater than 1% in the merged sample. We calculate the PCs for the 1000 Genomes subsample and project the remaining individuals onto these PC weights. Finally, we plot the first four PCs against each other and visually identify individuals that cluster together with the 1000 Genomes EUR sample.

LDpred PGI for African-genetic-ancestry individuals in the HRS and Add Health. In order to examine how well a PGI for years of education predicts *EduYears* among African-genetic-ancestry individuals, we construct an LDpred PGI for African-genetic-ancestry individuals in *HRS and Add Health* using the same LDpred weights described above. We identify the African-genetic-ancestry individuals following the identical procedure that we use for European-genetic-ancestry individuals (as described in the previous paragraph) but using the 1000 Genomes AFR sample.

Clumping and Thresholding PGIs. In addition to our main LDpred PGIs, we analyze several PGIs made using the "clumping and thresholding" (C+T)[48] method. In order to make these PGIs, we first clump our meta-analysis excluding *Add Health*, *HRS* and *WLS* using the algorithm for obtaining lead SNPs described in **Supplementary Note** section 2.2.6 but without a *P* value cutoff. Then, from this set of "approximately independent" SNPs, we select SNPs that have association *P* values less than 5×10^{-8}, 5×10^{-5}, 5×10^{-3}, and 1 (i.e., all SNPs). For each set of SNPs, we obtain PGIs using Plink2[27] as weighted sums of genotype probabilities at each SNP in the set, where the weights are set equal to the coefficient estimates from the meta-analysis.

X chromosome PGI. We also examine, in HRS, a PGI made using only the SNPs on the X chromosome. We conducted this analysis only in *HRS* because we did not have

imputed genotypes for the X chromosome in *Add Health*. We made the PGI by applying the C+T methodology described above (see **Supplementary Note** section 3.4 for details on the clumping procedure for the X chromosome) with a *P* value cutoff of 1 to our X chromosome meta-analysis. Prior to clumping, we restricted the set of SNPs in the summary statistics to those available in *HRS* genotype data.

5.2 Phenotypes

We supplement the education and cognition outcomes that we analyzed in EA3 with an academic achievement outcome in *WLS* that we have not examined before: high school grades percentile rank. We also incorporate new waves of data from *HRS* that have been made available since EA3 was published. Below, we describe in detail how each outcome was constructed.

5.2.1 Education outcomes

We analyze education outcomes in *HRS* and *Add Health*, both of which provide comparable measures of completed education. Our main education phenotype is years of education (*EduYears).* In additional analyses of both the *Add Health* and *HRS* samples, we consider three binary variables related to educational attainment: (i) High School Completion, (ii) College Completion, and (iii) Grade Retention (i.e., retaking a grade). We omit *WLS* from the analysis of education outcomes because *WLS* has a truncated distribution of *EduYears.*

Add Health: We obtain the *EduYears* variable using the following survey item from the Wave IV In Home Interview:

- *"What is the highest level of education that you have achieved to date? (1. 8th grade or less / 2. Some high school / 3. High school graduate / 4. Some vocational/technical training / 5. Completed vocational/technical training (after high school) / 6. Some college / 7. Completed college (bachelor's degree) / 8. Some graduate school / 9. Completed a master's degree / 10. Some graduate training beyond a master's degree / 11. Completed a doctoral degree / 12. Some post baccalaureate professional education (e.g., law school, med school, nurse) / 13. Completed post baccalaureate professional education (e.g., law school, med school, nurse) / 96. Refused / 98. Don't know)"*

We use the following mapping to ISCED[49] categories and from ISCED to *EduYears* for individuals older than 25 years old:

- 1 → ISCED = 1 → *EduYears* = 7
- 2 → ISCED = 2 → *EduYears* = 10
- 3, 4, 6 → ISCED = 3 → *EduYears* = 13
- 5 → ISCED = 4 → *EduYears* = 15
- 7, 8, 9, 10, 12 → ISCED = 5 → *EduYears* = 19
- 11, 13 → ISCED = 6 → *EduYears* = 22

The *Completed High School* variable is an indicator variable equal to 1 if *EduYears* ≥ 13. *Completed College* is an indicator equal to 1 if *EduYears* ≥ 19.

The *Grade Retention* variable is an indicator variable set to 1 for "Yes" responses to the following item from the Wave I In Home Interview, and to 0 for "No" responses:

"Have you ever repeated a grade or been held back a grade? (0. No / 1. Yes / 6. Refused / 8. Don't know)"

HRS: In HRS, we use two survey items from the HRS 2018 cross-wave tracker file (early release - v1.0):

- Q1. *"What is the highest grade of school or year of college you completed? (0-17)"*
- Q2. *"Highest degree of education (0. No degree / 1. GED / 2. High school diploma / 3. Two year college degree / 4. Four year college degree / 5. Master degree / 6. Professional degree (Ph.D., M.D., J.D). / 9. Degree unknown/Some College)"*

We map the responses to ISCED categories and ISCED categories to *EduYears* as following:

- Q1 = 0 and Q2 = 0 → ISCED = 0 → *EduYears* = 1
- Q1 = 1-6 and Q2 = 0, → ISCED = 1 → *EduYears* = 7
- Q1 = 7-12 and Q2 = 0 → ISCED = 2 → *EduYears* = 10
- Q2 = 1 or 2 → ISCED = 3 → *EduYears* = 13
- Q2 = 3 → ISCED = 4 → *EduYears* = 15
- Q2 = 4 or 5 → ISCED = 5 → *EduYears* = 19
- Q2 = 6 → ISCED = 6 → *EduYears* = 22

The *Completed High School* variable is set equal to 1 for subjects who report having a GED or high school diploma (9 > Q2 ≥ 1), and 0 otherwise (Q2 < 1). Similarly, the *Completed College* variable is equal to 1 for subjects who report having a four-year college degree (9 > Q2 ≥ 4), and 0 otherwise (Q2 < 4).

For *Grade Retention*, we use the following question from the 2006 - 2012 Core waves: "*For each of the following events, please indicate whether the event occurred AT ANY POINT IN YOUR LIFE. If the event did happen, please indicate the year in which it happened most recently. (Mark (X) one box for each line. If "Yes", indicate which year.) Before you were 18 years old, did you have to do a year of school over again? (Yes/No)*". The variable was set to 1 if the response was "Yes" in any of the waves, 0 otherwise.

5.2.2 Cognitive and academic achievement outcomes

In addition to the education outcomes, we analyze some detailed measures of scholastic achievement in adolescence available in *Add Health* and *WLS*, cognitive performance measures in *Add Health* and *WLS*, and measures of several dimensions of cognitive functioning in older individuals in *HRS*.

Add Health: In *Add Health*, we analyze *Verbal Cognition* and several grade point average (GPA) measures. *Verbal Cognition* was measured using a modified version of the Peabody Picture Vocabulary Test[50] in the first wave of *Add Health*, when participants were 12–20 years old.[50] The test contains eighty-seven items where the interviewer reads a word aloud, and a respondent selects the illustration that best fits the word's meaning. The scores were age-standardized.

The GPA measures were calculated using the common United States 0.0 to 4.0 range from respondents' transcripts that were collected from their high schools as part of Wave III Education Data. We analyze cumulative *Overall GPA*, as well as subject-specific cumulative GPAs: *Math GPA*, *Science GPA* and *Verbal GPA* (calculated as the mean of English GPA, Foreign Language GPA, and History and Social Sciences GPA).

***HRS*:** In *HRS*, we analyze *Total Cognition*, *Verbal Cognition*, changes over time in *Total* and *Verbal Cognition*, and *Alzheimer's disease*. *Total Cognition* is the sum of correct responses to cognitive tests from three domains common across waves 2 through 13 (1993-2016) measuring memory (immediate word recall task, delayed word recall), working memory (serial 7's), and mental status (backwards count, date naming, object naming, president/vice president naming), with a total score ranging from 0 to 35. The *Verbal Cognition* measure was adapted from WAIS-R[51]. In waves 3 through 13 (1995-2016), respondents were asked to define 5 words. Responses were rated as incorrect (0), partially correct (1) or completely correct (2), resulting in a total score ranging from 0 to 10. We include only those total and verbal cognition scores with no missing/imputed item values. To evaluate changes over time, we study wave-to-wave changes in *Total Cognition* and *Verbal Cognition*, $(x_t - x_{t-1})$. Because the data are longitudinal, the unit of analysis for these four cognitive outcomes is a person-year. Our final cognitive outcome, *Alzheimer's disease*, is an indicator variable equal to one for subjects who report having been diagnosed with Alzheimer's disease in one of the 2010 – 2018 Core waves, and 0 otherwise.

WLS: In *WLS*, we analyze *Cognitive Performance* and *High School Grades Percentile Rank*. *Cognitive Performance* is measured with the Henmon-Nelson test of mental ability[52], a 30-minute multiple-choice test consisting of 90 individual verbal or quantitative items, with a maximum of 90 points. The Henmon-Nelson test is a psychometrically validated test whose scores are known to correlate highly with *g*. We use the test scores converted to the metric of junior-year since participants took the test at various grades, hampering the comparability of raw test scores. *High School Grades Percentile Rank* was calculated as $100 - 100 \times$ (rank in class / # of students in class).

5.3 Controls

All regressions of education outcomes include the following set of controls: a full set of dummy variables for year of birth, an indicator variable for sex, a full set of interactions between sex and year of birth, and the first 10 principal components (PCs) of the variance-covariance matrix of the genetic data. In regressions of cognitive and academic achievement outcomes in *HRS* and *Add Health*, we replace year of birth with age at assessment. In the *WLS* Henmon-Nelson analysis, instead of replacing year of birth with age at assessment, we control for both (and their interactions with sex) because the analysis included both primary respondents and their siblings, whose year of assessment differed substantially from the year of assessment for primary respondents in some cases. In our analyses of GPA outcomes in *Add Health*, we also control for high school fixed effects.

PCs are constructed after restricting the samples to individuals from European genetic ancestries. We start by converting the imputed genotype probabilities to hard calls and removing markers with imputation accuracy less than 70% or minor allele frequency less than 1%, as well as markers in long-range LD blocks (chr5:44mb-51.5mb, chr6:25mb-33.5mb, chr8:8mb-12mb, chr11:45mb-57mb). Next, we prune the markers to obtain a set of approximately independent markers, using a rolling window of 1000

base pairs (incremented in steps of 5) and an R^2 threshold of 0.1. We use this set of markers to estimate a genetic relatedness matrix. We identify all pairs of individuals with a relatedness coefficient greater than 0.05. We exclude one individual from each pair, calculate the first 10 PCs for the resulting sample of unrelated individuals using Plink2[22,27], and project the PCs onto the sample of unrelated individuals.

For the African-genetic-ancestry individuals in *HRS*, we construct the PCs in a similar fashion, the only difference being the exclusion of long-range LD blocks. Since the long-range LD blocks listed above are observed in European-genetic-ancestry individuals, whereas African-genetic-ancestry individuals are known to have much less LD in general than European-genetic-ancestry individuals, we do not exclude those regions when calculating the PCs for the African-genetic-ancestry subsample of *HRS*.

5.4 Defining Prediction Accuracy

To evaluate prediction accuracy, we use the same two-step process that we used in EA3. First, we regress the phenotype on the set of controls described above without the PGI. Next, we rerun the same regression but with the PGI included. For quantitative phenotypes, our measure of predictive power is the incremental R^2: the difference in R^2 between the regressions with and without the PGI. For binary outcomes, we proceed similarly but calculate the incremental pseudo-R^2 from a Probit regression. We obtain 95% confidence intervals (CIs) around the incremental (pseudo-)R^2's by performing a bootstrap with 1000 repetitions.

5.5 Expected Prediction Accuracy of the *EduYears* PGI

Here, we describe a framework to assess how the observed change in predictive power of the *EduYears* PGI is consistent with theoretical projections of the change in predictive power. This calculation is based on a generalization of de Vlaming et al.[53], which gives the expected predictive power of a PGI for a given discovery sample size when the heritability of the discovery and prediction sample may be different and the genetic correlation of the phenotype between the discovery and prediction sample may be less than one. The generalization we derive here is novel, as far as we are aware.

First, we define some terms. Let y_i denote the phenotype and $\hat{g}_i$ denote the PGI for individual i in the prediction sample. We can decompose $\hat{g}_i$ as $\hat{g}_i = g_i + e_i$, where g_i is the PGI that would be estimated if the discovery sample size were infinite and e_i is the portion of the PGI that is due to sampling variation from estimating the PGI weights. Because the discovery and prediction sample are non-overlapping, e_i is uncorrelated with both g_i and y_i (this would be true exactly if the PGI weights were estimated using multivariable regression in the discovery sample, but it is approximately true if the PGI weights are estimated using LDpred from GWAS summary statistics, as they are here[54]). We assume that the error in the PGI is decreasing inverse-proportionately to the discovery sample size, N, such that $\mathrm{Var}(e_i) = M/N$ for some constant M. The constant factor M will be affected by factors related to the effective number of SNPs included in the PGI, including linkage disequilibrium patterns and assortative mating.

We now derive a general expression for the predictive power of the PGI for different discovery sample sizes. We calculate

$$E(R^2) = \frac{\mathrm{Cov}(y_i, \hat{g}_i)^2}{\mathrm{Var}(y_i)\mathrm{Var}(\hat{g}_i)}$$

$$= \frac{[\text{Cov}(y_i, g_i) + \text{Cov}(y_i, e_i)]^2}{\text{Var}(y_i)[\text{Var}(g_i) + \text{Var}(e_i)]}$$

$$= \frac{\text{Cov}(y_i, g_i)^2}{\text{Var}(y_i)[\text{Var}(g_i) + M/N]}$$

$$= \frac{\text{Cov}(y_i, g_i)^2/M}{\text{Var}(y_i)[\text{Var}(g_i)/M + 1/N]}$$

$$= \frac{A}{B + 1/N},$$

where $A \equiv \text{Cov}(y_i, g_i)^2/[M\text{Var}(y_i)]$ and $B \equiv Var(g_i)/M$. Notice that through this derivation, we only assume that the sampling variance decreases at a rate $1/N$ and that the discovery and prediction samples are non-overlapping; no other assumptions are needed for predictive power of a PGI to have this general form of dependence on the GWAS discovery sample size for a fixed prediction sample. We highlight that B is only related to the variance of the infinite-discovery-sample PGI and the effective number of SNPs, both things that are not dependent on the prediction or discovery samples. The parameter A will be related to a number of factors, including the heritability of the phenotype in each cohort and genetic correlation of the phenotype between the discovery and prediction cohorts. Thus, it is only the parameter A that may differ by prediction sample.

Finally, we describe how we calibrate the parameters A and B to assess how well our observed predictive power aligns with theoretical expectations. We will do so using non-linear least squares. Specifically, using multiple estimates of predictive power across several prediction cohorts and PGIs based on various discovery sample sizes, we find

$$\min_{A_j, B} \left\{ \sum_{j,k} \left(R_{j,k}^2 - \frac{A_j}{B + \frac{1}{N_k}} \right)^2 \right\}$$

where $R_{j,k}^2$ is the estimated incremental R-squared of the PGI in prediction sample j using PGI k and N_k is the discovery sample size of PGI k. Notice that we find a different value of A_j for each prediction sample, but we hold B constant across all prediction cohorts. These parameters are estimated using four PGIs from three studies published prior to this one: Rietveld et al.[24], Okbay et al.[1] (one PGI from the discovery sample and one PGI from the combined discovery and replication sample), and Lee et al.[2] Using this procedure, we estimate $B = 2.242 \times 10^{-6}$. For the *Add Health* prediction cohort, we estimate $A_{\text{AddHealth}} = 3.455 \times 10^{-7}$. For the *HRS*, we have $A_{\text{HRS}} = 3.091 \times 10^{-7}$.

Using the estimated values of A_j and B and using the sample size from this study (N = 3,014,057), we calculate the projected theoretical predictive power of the PGI from this study in our prediction cohorts.

5.6 Results

Supplementary Table 4 and **Extended Data Figure 6** provide a summary overview of the results from our prediction analyses as well as descriptive statistics for the

phenotypes considered. In addition to the measure of prediction accuracy described above, for each outcome, we report the coefficient of the *EduYears* PGI and its standard error from an ordinary least squares regression of the outcome on the PGI and controls. Since the unit of analysis is a person-year for all cognitive outcomes in *HRS* except for Alzheimer's disease, we cluster standard errors at the person level in these regressions.

Education Outcomes. We begin with our primary phenotype, *EduYears*. In *Add Health*, one standard deviation increase in the *EduYears* PGI is associated with 1.33 years of schooling ($SE = 0.04$), with an incremental-R^2 of 15.8% (95% CI: 14.0% to 17.3%). In *HRS*, the PGI has an incremental-R^2 of 12.0% (95% CI: 10.8% to 12.9%). One standard deviation increase in the *EduYears* PGI is associated with 1.22 extra years of schooling ($SE = 0.03$) in *HRS*.

Figure 2a in the main text depicts how the predictive power of the *EduYears* PGI changes with increasing *EduYears* GWAS meta-analysis sample sizes over time. In this figure, the actual predictive power of each PGI is plotted against the projected predictive power as a function of the discovery sample size (calculated as described in **Supplementary Note** section 5.5). To maximize comparability, all numbers reported in the figure are based on PGIs constructed using the methods described above and a common set of SNPs (namely, all HapMap3 SNPs present in each of the five meta-analyses). Our current meta-analysis increases the sample size from $N = 1{,}131{,}881$ to $N = 3{,}037{,}499$. This increase in sample size is paired with an increase in the prediction accuracy of the *EduYears* PGI from 11.0% to 15.9% in *Add Health*. In *HRS*, the incremental-R^2 increases from 9.6% to 12.1% (**Supplementary Table 29**). The predictive power of the PGI in the *HRS* aligns almost perfectly with expectation, but the predictive power of the PGI in *Add Health* exceeds expectations.

In **Extended Data Figure 5** and **Supplementary Table 3**, we show the predictive power of polygenic indexes made using the "clumping and thresholding" (C+T)[48] method with different *P* value cutoffs (5×10^{-8}, 5×10^{-5}, 5×10^{-3}, and 1). As a point of comparison, we also include our main LDpred PGI constructed using HapMap3 SNPs. The C+T PGI made using only genome-wide-significant SNPs explains 9.1% of the variance in *EduYears* in *Add Health* and 7.0% in *HRS*. Compared to the equivalent PGIs from EA3 (*Add Health* incremental R^2 = 3.8%, *HRS* incremental R^2 = 2.5%), this is a substantial increase. The predictive power of the PGI goes up as the *P* value threshold increases until the cutoff of 5×10^{-3}, where the PGI explains 13.5% of the variation in *Add Health* and 10.9% in *HRS*. The PGI with the *P* value cutoff of 1 (i.e., including all approximately independent SNPs) explains slightly less (12.7% in *Add Health*, 10.4% in *HRS*). Finally, the LDpred PGI generates further gains in predictive power for both *Add Health* and *HRS*, to 15.8% and 12.0%, respectively.

Next, we examine the results from analyses of our three binary education outcomes: (i) *High School Completion*, (ii) *College Completion*, and (iii) *Grade Retention*. In *Add Health*, a one-standard-deviation increase in the *EduYears* PGI is associated with a 5.5 percentage-point increase in the probability of completing high school (incremental pseudo-R^2 = 7.8%), a 18.6 percentage-point increase in the probability of completing college (incremental pseudo-R^2 = 12.6%), and a 8.9 percentage-point reduction in the probability of having retaken a grade (incremental pseudo-R^2 = 5.4%). The corresponding figures in the *HRS* are a 10.8 percentage-point increase in the probability of completing high school (incremental pseudo-R^2 = 8.0%), a 14.6 percentage-point increase in the probability of completing college (incremental pseudo-R^2 = 9.3%), and a 5.9 percentage-point decrease in the probability of having retaken a grade

(incremental pseudo-$R^2 = 2.6\%$). All effects are statistically distinguishable from zero at $P < 0.001$ and substantial with respect to baseline prevalences of 93%, 32% and 19%, in *Add Health,* and of 82%, 25% and 16% in *HRS,* respectively.

Figure 2b in the main text and **Extended Data Figure 7** visualize the results by showing the prevalences of each of our 3 binary outcomes across deciles of our *EduYears* PGI, where the 1st decile reflects the lowest PGIs and the 10th decile reflects the highest PGIs. Each quintile contains roughly 565 individuals in *Add Health* and roughly 1,085 individuals in *HRS*. We observe substantial differences between prevalences in lowest and highest PGI deciles: 63.4 and 46.2 percentage-point difference in the prevalence for *College Completion*, 20.0 and 36.5 percentage-point difference for *High School Completion*, and 31.0 and 21.6 percentage-point difference for *Grade Retention* in *Add Health* and *HRS*, respectively.

Finally, in *HRS*, we examine a PGI made using our X chromosome meta-analysis results. The PGI has an incremental R^2 of 0.38% (95% CI: 0.12% to 0.80%) in the sample of men, 0.13% (95% CI: 0.02% to 0.38%) in the sample of women, and 0.25% (95% CI: 0.10% to 0.49%) in the pooled-sex sample. The predictive power of the PGI is larger in the sample of men compared to women (although the confidence intervals overlap), which seems counterintuitive given that we estimate higher X chromosome heritability of *EduYear*s in the sample of women in UKB (see **Supplementary Table 17**). However, using GCTA[23], we estimated a larger autosomal heritability for *EduYears* in *HRS* males ($h^2 = 0.29,\ S.E. = 0.06$) compared to females ($h^2 = 0.19,$ $S.E. = 0.08$), suggesting that contrary to what we observe in UKB, X chromosome heritability of *EduYears* in *HRS* may be larger in males.

In order to assess how much the predictive power of the X chromosome PGI increased with the increase in sample size, we compared two PGIs based on the EA3 and EA4 X chromosome meta-analyses, made using the same methodology and SNPs available in both studies. These results are shown in Table 1.

Cognitive and Academic Achievement Outcomes. In *Add Health*, we examine five academic achievement outcomes: (i) *Verbal Cognition* (Peabody verbal score), (ii) *Overall GPA*, (iii) *Math GPA*, (iv) *Science GPA,* and (v) *Verbal GPA*. The *EduYears* PGI positively predicts all outcomes (P value < 0.001). A one-standard-deviation increase in the PGI is associated with a 3.7-point increase in the Peabody test scores (incremental-R^2 = 8.7%), a 0.3-point increase in *Overall GPA* (incremental-R^2 = 12.3%) and *Math GPA* (incremental-R^2 = 8.4%), and 0.4-point increases in *Science GPA* (incremental-R^2 = 10.0%) and *Verbal GPA* (incremental-R^2 = 12.4%).

In *HRS,* we analyze cognitive functioning in older individuals using measures of *Total* and *Verbal Cognition*, changes in total and verbal cognition over time, and *Alzheimer's disease*. The *EduYears* PGI positively predicts the *Total Cognition* and *Verbal Cognition* scores (P value < 0.001) with incremental-R^2 = 3.1% and 4.7%, respectively. However, the PGI's association with wave-to-wave changes in *Total Cognition* is only suggestive (P value = 0.04) and the PGI does not predict wave-to-wave changes in *Verbal Cognition* (P value = 0.46) or *Alzheimer's disease* (P value = 0.15).

Finally, we examine *Henmon-Nelson test scores* and *High School Grades Percentile Rank* in WLS. The *EduYears* PGI is positively predictive of both outcomes. A one standard deviation increase in the PGI is associated with a 2.9-point increase in the

Henmon-Nelson test scores (incremental-R^2 = 6.1%)[20] and an 8.0-point increase in *High School Grades Percentile Rank* (incremental-R^2 = 7.7%).

EduYears in African-genetic-ancestry individuals in the HRS and Add Health. We examine how well the *EduYears* PGI predicts *EduYears* among African-genetic-ancestry individuals in *HRS* (*N* = 2,507) and *Add Health* (*N* = 1,716). We find that the PGI has an incremental R^2 of 1.3% (95% CI: 0.6% to 2.2%) in *HRS* and 2.3% (95% CI: 1.1% to 3.7%) in *Add Health*. This represents 89% and 85% attenuation in the predictive power of the PGI compared to our European-genetic-ancestry samples from *HRS* and *Add Health*, respectively (**Supplementary Table 21**). We note that the *HRS* incremental R^2 is smaller than we report in EA3 (incremental-R^2 = 1.6%). In order to understand the reason for the discrepancy, we estimated the incremental-R^2 in the same *HRS* African-genetic-ancestry prediction sample that we analyzed in EA3, and found that in that sample, the incremental R^2 rises to 2.2%. Therefore, we conclude that the decrease in incremental R^2 stems from the sample definition: in EA3, our African-genetic-ancestry prediction sample was restricted to the list of African-American individuals provided by the *HRS*, whereas here, we identify African-genetic-ancestry individuals as described in **Supplementary Note** section 5.1.

5.7 Analysis of *European genetic ancestries to African genetic ancestries relative accuracy* in UK Biobank

We define the *European genetic ancestries to African genetic ancestries relative accuracy* (RA) as

$$RA_{E\to A} = \frac{R^2_{AFR}}{R^2_{EUR}},$$

where R^2_{AFR} and R^2_{EUR} are prediction accuracies (fractions of phenotypic variance explained) of PGIs derived from a GWAS conducted in European-genetic-ancestry populations. The previous literature has established that the RA is usually substantial. For example, Duncan et al.[55] found that the average $RA_{E\to A}$ across multiple phenotypes is 36%, and Martin et al.[56], who examined mostly different phenotypes but with some overlap, found an average $RA_{E\to A}$ of 22%. In our analyses of our EA PGI in the *HRS* and *Add Health* samples described above, our estimate of $RA_{E\to A}$ is 11% and 15%, respectively (both estimates are similar to the 15% we found for HRS for the EA PGI from a previous GWAS of EA[2]).

To investigate the factors contributing to the substantial loss of prediction accuracy, and to understand if the reduction observed for EA is anomalously large relative to what has been found for other phenotypes, we used a method[57] that was recently developed by Wang et al.[57]. Wang et al. applied their method to five quantitative phenotypes (albeit not EA) and three common diseases in the *UKB* (average $RA_{E\to A}$ of 24% across the eight phenotypes). Wang et al. consider a model in which the loss in prediction accuracy depends on population differences in linkage disequilibrium (LD), allele frequencies, and SNP heritability, as well as on the cross-population correlation of

[20] The incremental R^2 of the PGI for Henmon-Nelson test scores is lower than what we report in EA3 (incremental-R^2 = 7.73%). We note that these results are not comparable for two reasons: (i) In EA3, the phenotype is erroneously reported to be the "raw Henmon-Nelson test scores", while it actually is an IQ score mapped from raw Henmon-Nelson test scores, (ii) the sample of individuals in the current study is larger.

causal SNP effects. They then derive an analytic formula that can be used to infer the contribution of LD and allele frequencies to the relative accuracy between any two populations. While their formula is derived for PGIs based on independent genome-wide-significant SNPs, they show through simulations that it also gives a good approximation for genome-wide PGIs, such as PGIs (like ours) based on LDpred[47]. For EA, we also observed similar RA between our LDpred-based PGI and that based on independent genome-wide-significant SNPs (**Supplementary Table 21**).

To facilitate comparability with Wang et al.'s[57] results for *European genetic ancestries to African genetic ancestries* prediction accuracy loss, we extended their original analyses to also include EA. We thus performed a GWAS of HapMap 3 SNPs (1,365,446 SNPs) in a sample of European-genetic-ancestry individuals in *UKB* (N = 425,231 individuals). We identified 507 approximately independent genome-wide-significant SNPs (using the LD clumping algorithm implemented in Plink1.9[22], setting the window size equal to 1 Mb and the LD r^2 threshold of 0.1). We then used these 507 SNPs to generate PGIs and evaluate their accuracy in *UKB* hold-out samples of African-genetic-ancestry individuals (N = 6,514) and European-genetic-ancestry individuals (N = 10,000). We found a RA of 0.33% (S.E. = 0.94%).

Next, we used the methods developed in Wang et al.[57] to compare our empirical estimate of RA to the RA predicted by the model. To generate the prediction, we used genotypes from 503 European-genetic-ancestry and 504 African-genetic-ancestry participants in the 1000 Genomes Project to estimate genetic-ancestry-specific MAF and LD correlations between all candidate causal variants (defined as any SNP within a 100 kb window of a genome-wide-significant SNP whose squared correlation with the genome-wide-significant SNP is above 0.45). Following Wang et al.[57], we then substituted these estimates into their Equation (2) and evaluated the expression. For EA, the predicted RA is 35%.

Extended Data Figure 8 and **Supplementary Table 5** show these results for EA together with the results obtained by Wang et al. in an analogous way for the eight phenotypes they study. Compared to the other phenotypes, not only is the observed RA substantially lower for EA, but the proportion of loss attributable to MAF and LD is somewhat smaller for EA. The smaller proportion of loss attributable to MAF and LD implies that the remaining loss is due to environmental factors, which would cause differences in phenotypic heritability across populations or an imperfect cross-population correlation of causal effects (for example, due to gene-environment interactions).

We highlight three caveats to our analysis. First, differential ascertainment of African-genetic-ancestry versus European-genetic-ancestry participants of the *UKB* could have biased the observed RA. In fact, the mean EA in the African-genetic-ancestry sample is slightly larger than the mean EA in the European-genetic-ancestry sample—15.9 years versus 15.3 years (t-test $P = 2.5 \times 10^{-21}$)—suggesting a stronger ascertainment bias. This differential ascertainment bias could reduce the heritability in the African-genetic-ancestry sample relative to the European-genetic-ancestry sample, thereby contributing to the reduced predictive power in the African-genetic-ancestry sample. (The sample size of African-genetic-ancestry individuals is too small to reliably estimate the SNP heritability.). Consequently, the contribution of MAF and LD differences between European and African genetic ancestries to the loss of accuracy is likely to be larger in the general UK population compared to the *UKB* sample.

Second, the African genetic-ancestry-sample likely includes a higher fraction of immigrants to the UK than the European-genetic-ancestry sample. Individuals who completed some or all of their schooling outside the UK education system are less comparable.

Third, we have followed Wang et al.[57] in studying African-genetic-ancestry and European-genetic-ancestry samples in the *UKB*, so our findings may not generalize to our sample of African-Americans in the *HRS* and *Add Health*. In fact, for PGIs based on independent genome-wide significant SNPs, we find RAs of 10.27% (S.E. = 4.52%) and 13.52% (S.E. = 5.96%) in *HRS* and *Add Health*, respectively, substantially larger than the RA in the *UKB*.

5.8 Post-hoc analyses with SBayesR PGI

We ran all of our main analyses in the paper using a PGI constructed with LDpred, partly for comparability with Lee et al.[2] and partly because at the time we undertook our main analyses, we had convergence problems with SBayesR[58] that were subsequently fixed. In post-hoc analyses, we assessed the predictive power of a PGI made using the SBayesR methodology implemented in the GCTB software[59]. SBayesR is a Bayesian method that differs from LDpred in that it imposes a flexible finite mixture of normal distributions as the prior on the SNP effects instead of a point-normal mixture distribution. Like LDpred, SBayesR requires an estimate of LD between SNPs. We used the 2,865,810 pruned common variants from the full UKB European-genetic-ancestry ($N \approx 450{,}000$) data set from Lloyd-Jones et al.[58] as our LD reference data. We excluded 3,638 SNPs in the MHC region (Chr6 : 28-34Mb) from the analysis as recommended by Lloyd-Jones et al.[58], as this was observed to improve model convergence. The 2,548,339 remaining SNPs that were available in the meta-analysis excluding *HRS*, *Add Health* and *WLS* were included in the analysis. We ran SBayesR assuming 4 components in the finite mixture model, with initial mixture probabilities $\boldsymbol{\pi} = (0.95, 0.02, 0.02, 0.01)$ and fixed $\boldsymbol{\gamma} = (0.0, 0.01, 0.1, 1)$, where $\boldsymbol{\gamma}$ is a parameter that constrains how the common SNP effect variance scales in each of the four distributions. The MCMC was run for 10,000 iterations with 2,000 taken as burn-in.

We obtained the PGIs for European-genetic-ancestry individuals in Plink2[27] by multiplying the genotype probabilities at each SNP by the corresponding estimated posterior mean calculated by SBayesR, and then summing over all included SNPs (2,548,339 in *Add Health*, 2,540,570 in *HRS*). The PGI explained 17.0% (95% CI: 15.2% to 18.5%) of the variance in *EduYears* in *Add Health* and 12.9% (95% CI: 11.7% to 13.9%) in *HRS*, approximately 1 percentage-point higher than the LDpred PGI in each of the two cohorts.

6 Prediction of disease risk from the EA PGI

To evaluate the potential relevance of the EA PGI in clinical setting, we estimated its predictive power for 10 common diseases among individuals of European genetic ancestry in the UK Biobank. To benchmark those results, we also estimated the predictive power of disease-specific PGI for each disease.

Our results imply that the predictive power of the EA PGI for the diseases is non-trivial compared to the disease-specific PGI: the predictive power of the EA PGI is on average about half that of the disease-specific PGI across the 10 diseases.

6.1 Construction of PGIs

6.1.1 EA PGI

The EA PGI was constructed using LDpred (v.1.0.11)[47], following the pipeline described in **Supplementary Note** section 5.1 and using the summary statistics of an EA meta-analysis that excludes the UK Biobank. A total of 1,134,788 SNPs that overlapped across the summary statistics, UK Biobank genotype data, and the 1,214,408 HapMap3 SNPs in the HRC reference panel that we used to estimate LD, and passed the LDpred quality control filters, were used to construct the PGI.

6.1.2 Diseases PGI

The PGIs for diseases were derived and analyzed at a separate location (the University of Queensland) than the EA PGI. To minimize the differences in the way the PGIs were constructed across the two locations, we used the same reference panel to estimate LD in the two locations (i.e., 14,028 individuals and 1,214,408 HapMap3 SNPs from the public release of the aforementioned HRC reference panel). We also restricted our hard-call genotypes and discovery GWAS summary statistics to the same set of HapMap3 SNPs. The main difference between the way the EA and diseases PGIs were constructed is that dosage data were used for the EA PGI, whereas hard calls were used for the disease-specific PGIs. Because we only used HapMap3 SNPs for all PGI and because HapMap3 SNPs tend to be well-imputed, this difference is unlikely to matter much in practice.

UK Biobank genotype data and diseases phenotypes

Genotype data from UK Biobank (UKB, Project: 12505) individuals were imputed using the HRC and UK10K as the reference sample. Genotype probabilities were converted to hard-call genotypes using Plink2 (`--hard-call-threshold 0.1`), excluding SNPs with Hardy-Weinberg equilibrium test P value < 10^{-5}, missing genotype rate > 0.05, or imputation accuracy (INFO) score < 0.3. Individuals with European genetic ancestry were identified by projecting the UKB sample onto the first two principal components (PCs) of the 1000 Genome Project (1KGP), using HapMap3 SNPs with MAF > 0.01 in both data sets. An individual was assigned European genetic ancestry if they had > 0.9 posterior probability of belonging the 1KGP European reference cluster. A total of 8,546,065 SNPs with MAF ≥ 0.01 among the UKB European individuals were identified, of which 1,133,746 SNPs overlapped with the 1,214,408 HapMap3 SNPs used in EA PGI derivation step.

442,091 UKB European individuals with non-missing EA phenotype and covariates (sex, a third degree polynomial in birth year and interactions with sex, the first 40 PCs,

and batch dummies) were retained after sample quality control (QC). (We used the same set of QC'd individuals as we used for an earlier version of our EA GWAS in the UKB; our final EA GWAS in the UKB used slightly fewer individuals since, subsequent to the analyses discussed here, some individuals withdrew their consent for their data to be analyzed.) Diseases phenotypes were generated based on UKB Category 1712 and Data-Field 41270 among these individuals.

Briefly, we only selected cases with data from primary care, hospital admission, or death registry records (those with self-reported data only were removed). After the case status was defined, among the remaining individuals we removed individuals with similar diseases (control screening, e.g., we removed individuals with type 1 diabetes or gestational diabetes from type 2 diabetes controls). In total, we selected 10 common diseases. **Supplementary Table 23** provides additional details on the definitions and coding of these diseases and of the cases and controls.

Discovery GWAS summary statistics

For PGI derivation, we used summary statistics from GWAS studies conducted among participants of European genetic ancestry for nine traits: type 2 diabetes (T2D)[60], low-density lipoprotein cholesterol (LDLC)[61], systolic blood pressure (SBP)[62], rheumatoid arthritis (RA)[63], femoral neck bone mineral density (BMD)[64], major depression (MD)[65], asthma[66], coronary artery disease (CAD)[67] and migraine[68]. For migraine GWAS summary statistics, only SNPs with association P value < 10^{-5} were available.

A PGI was constructed for each of the nine traits, and these nine PGI were used to predict a set of ten phenotypes (the PGI for CAD was used to predict two diseases: ischaemic heart disease and myocardial infarction). **Supplementary Table 22** lists the GWAS used to construct the nine PGIs, provides additional details on how the summary statistics were obtained, and shows the phenotypes that were predicted with each PGI.

UKB samples were, to the best of our knowledge, not included in any of the nine GWAS studies, consistent with the results from bivariate LD score regression analyses (**Supplementary Table 24**). These GWAS summary statistics were formatted and restricted to the aforementioned 1,133,746 SNPs.

PGI derivation

For the first eight traits listed above, we first estimated the weights using LDpred (v.1.0.11) and obtained the PGI using Plink1.9. For migraine, we used a clumping and thresholding derivation strategy (since only SNPs with P value < 10^{-5} were available).

LDpred was run using the same settings and HRC reference data used in the EA PGI derivation step. The HRC reference data (EGAD00001002729) were downloaded from https://www.ebi.ac.uk/ega/ after ethics approval, and were converted from vcf format to Plink binary format using Plink1.9. The LD matrix was calculated using Plink1.9 and was used as input for `--ldf` in the `gibbs` step (instead of using the genotype data as input and letting LDpred calculate the LD matrix). In order to calculate the LD matrix, we first restricted the data to the 1,214,408 HapMap3 SNPs and 14,028 individuals in the HRC reference data that were also used in the EA PGI derivation step. We then used Plink1.9 with the options `--r2 --ld-window 99999 --ld-window-kb 500 --ld-window-r2 0`. PGIs were obtained using the LD-adjusted weights in Plink1.9 and hard-call genotypes for the 1,133,746 SNPs with MAF ≥ 0.01 among the UKB European individuals that overlapped with the 1,214,408 HapMap3 SNPs.

The SNPs from the formatted migraine GWAS summary statistics (only SNPs with association *P* value < 10^{-5}) were clumped, discarding SNPs within 1,000 kb of, and in r^2 > 0.1 with, another (more significant) SNP using the aforementioned LD reference data. After clumping, the weights from the GWAS summary statistics of these remaining SNPs, together with the aforementioned UKB hard-call genotypes, were used to calculate migraine PGI in Plink (v1.90b).

6.2 Prediction of disease from PGI

6.2.1 Analyses

As mentioned in the Online Methods, we computed the predictive power of the EA and disease-specific PGI for the various diseases (**Supplementary Table 6**). Our measure of a PGI's predictive power for binary phenotypes is the incremental *Nagelkerke's* R^2 after adding the PGI to a logistic regression of the disease phenotype on sex, a third-degree polynomial in birth year and interactions with sex, the first 40 PCs, and batch dummies. For each disease, we then compared the incremental *Nagelkerke's* R^2 of the EA and disease-specific PGI. 95% confidence intervals (CIs) around the incremental *Nagelkerke's* R^2 was obtained by performing a bootstrap with 1,000 repetitions. We also estimated the incremental *Nagelkerke's* R^2 after adding the EA PGI, the disease PGI and their interaction to a logistic regression of the disease phenotype on the same covariates as above.

We also computed the odds ratio for selected diseases by deciles of the EA PGI in the UK Biobank (**Supplementary Table 7**). We converted the EA PGI into deciles (1= lowest, 10 = highest), and nine dummy variables were created to contrast each of deciles 2-10 to decile 1 as the reference. Odds ratio and 95% confidence intervals were estimated using logistic regression while controlling for covariates (sex, a third degree polynomial in birth year and interactions with sex, the top 40 PCs, and batch dummies).

In another analysis, we converted the EA PGI into deciles (1 = lowest, 10 = highest PGI), and created nine dummy variables to contrast each of deciles 2-10 to decile 1 as the reference (**Supplementary Table 8**). Odds ratio (OR) and 95% CIs were estimated using logistic regression while controlling for the covariates. We also computed the OR and 95% CIs of the EA PGI top decile relative to the bottom 9 deciles pooled togther, again adjusting for the covariates. We repeated this analysis using deciles based on the disease-specific PGI instead of the EA PGI, as well as using deciles of risk as predicted based on the combination of the EA PGI, disease-specific PGI, and their interaction. Finally, we repeated these analyses but using the lowest decile of the EA PGI, disease-specific PGI and risk as predicted based on the EA PGI, disease-specific PGI and their interaction relative to the top 9 deciles combined.

6.2.2 Results

The results are shown in the main text **Figure 3** and **Supplementary Table 6**. The EA PGI is a predictor of all 10 diseases (*P* < 2.98×10^{-8} for osteoporosis and much smaller for the other phenotypes), and the predictive power of the EA PGI (as measured by the *Nagelkerke's* R^2) is on average about half as large as that of the disease-specific PGI. Importantly, for all 10 diseases, the incremental *Nagelkerke's* R^2 from the model with the combined PGI (with the EA PGI, the disease-specific PGI, and their interactions) is larger than the incremental *Nagelkerke's* R^2 from the model with the disease-specific PGI only, typically by ~40-50%. Interestingly, the predictive power of the combination

of the PGIs is approximately equal to the sum of that of the EA PGI and disease-specific PGI considered separately, suggesting that EA PGI is help for predicting these common diseases independent of the disease-specific PGI[21].

Through the stratification of the EA PGI of the UKB participants into deciles, as shown in **Extended Data Figure 9** and **Supplementary Table 7**, it can be seen that the top deciles have substantially lower ORs than the lower deciles for all 10 diseases, consistent with a protective role of the EA PGI for these diseases.

We also computed the OR of the EA PGI top decile relative to the remaining 9 deciles combined. As can be seen in **Supplementary Table 8**, for 9 of the 10 diseases the ORs of getting the disease are sizeably smaller for individuals in the top decile, again pointing to a protective role of the EA PGI for these diseases (for 9 of the 10 phenotypes, the P value for the null hypothesis that the OR in the top decile is equal to the OR in the other deciles is $\leq 6.97 \times 10^{-19}$; the exception is osteoporosis, for which $P = 0.08$). We then repeated the exercise, but using the bottom EA PGI decile relative to the remaining 9 deciles combined. The OR were all larger than 1.0 ($P \leq 1.79 \times 10^{-5}$ for all 10 phenotypes, including osteoporosis). We repeated this exercise by comparing deciles of the disease PGI and of risk as predicted based on the EA PGI, disease-specific PGI and their interaction[22]. As expected, the results are stronger than for the EA PGI alone and are shown in **Supplementary Table 8**.

---

[21] This "near-additivity" of the predictive power of the EA PGI and disease-specific PGI is likely due to two factors. First, the PGIs are noisy estimators of the additive genetic factors. The noise in the two PGIs attenuates their correlation, thus reducing the amount of disease variance they commonly explain. Second, the genetic correlations between EA and the diseases is probably low to moderate only.
To see both of these points, let $Y = \beta_1 S_1 + \beta_2 S_2 + \varepsilon$, where Y is the phenotype and S_1 and S_2 are the EA and disease-specific PGIs. If we regress Y on S_1 only, the resulting regression coefficient on S_1 will be asymptotically equal to $plim\ \hat{\gamma}_1 \equiv \gamma_1 = \frac{\text{Cov}(Y,S_1)}{\text{Var}(S_1)} = \beta_1 + \frac{\text{Cov}(S_1,S_2)}{\text{Var}(S_1)}\beta_2$, and the regression R^2 will be asymptotically equal to $plim\ R^2_{S_1} = \frac{{\gamma_1}^2\text{Var}(S_1)}{\text{Var}(Y)} = \frac{\left(\beta_1+\frac{\text{Cov}(S_1,S_2)}{\text{Var}(S_1)}\beta_2\right)^2\text{Var}(S_1)}{\text{Var}(Y)}$. Similarly, the R^2 of a regression of Y on S_2 will be asymptotically equal to $plim\ R^2_{S_2} = \frac{\left(\beta_2+\frac{\text{Cov}(S_1,S_2)}{\text{Var}(S_2)}\beta_1\right)^2\text{Var}(S_2)}{\text{Var}(Y)}$. Finally, the R^2 of the regression of Y on S_1 and S_2 will be asymptotically equal to $plim\ R^2_{S_1,S_2} = \frac{(\beta_1)^2\text{Var}(S_1)+(\beta_2)^2\text{Var}(S_2)}{\text{Var}(Y)}$. For the first point above, observe that when the PGIs S_1 and S_2 are very noisy estimators of the underlying additive genetic factors for EA and for the disease, $\text{Var}(S_1)$ and $\text{Var}(S_2)$ will be large relative to $\text{Cov}(S_1, S_2)$. For the second point above, observe that when the genetic correlation is small, $\text{Cov}(S_1, S_2)$ will be small relative to $\text{Var}(S_1)$ and $\text{Var}(S_2)$. Both of these imply that $\frac{\text{Cov}(S_1,S_2)}{\text{Var}(S_1)}$ and $\frac{\text{Cov}(S_1,S_2)}{\text{Var}(S_2)}$ will be small, in which case $plim\ R^2_{S_1,S_2} \approx plim\ R^2_{S_1} + plim\ R^2_{S_2}$. Thus there will be "near-additivity" of the predictive power of the EA PGI and disease-specific PGI.

[22] Note that increased femoral neck bone mineral density is associated with a lower risk of osteoporosis. Therefore, we took the top decile for the femoral neck bone mineral density PGI for the odds ratio calculations that demonstrate reduced risk (OR < 1).

7 Comparing direct and population effects of the EA PGI

Direct genetic effects are the causal effects of an individual's genetic material on that individual; indirect genetic effects are the causal effects of an individual's genetic material on another individual's phenotypes (through the environment)[69,70]. Associations between genetic variants, or PGIs, and phenotypes capture both direct genetic effects and indirect genetic effects, in addition to confounding due to gene-environment correlation (including bias from population stratification) and assortative mating[71]. We aim to decompose the population-level association between the EA PGI and various phenotypes into direct and remaining components by using genetic data on siblings and parents.

7.1 Population and Direct Effects of a PGI

Without loss of generality, we assume that the PGIs are standardized in the population to have variance one. Let PGI_{ij} be the phenotype of sibling j in family i, where "family" refers to a set of full biological siblings and both of their biological parents.

The "population effect", ψ, of a PGI is the coefficient from regression of an individual's phenotype, Y_{ij}, on that individual's PGI, PGI_{ij}:

$$\psi = \mathrm{Cov}(PGI_{ij}, Y_{ij}). \tag{7.1}$$

We refer to ψ as the "population effect," as it reflects the overall association between phenotype and PGI in the population. The population effect does not have a causal interpretation since it may include the effects of gene-environment correlation, including parental indirect effects and uncorrected-for population stratification bias, and assortative mating. The primary goal of the analysis is to compare the direct effect of the PGI, defined next, to this population effect.

The "direct effect" of a PGI comes from estimating the regression:

$$Y_{ij} = \mu + \delta PGI_{ij} + \alpha\left(PGI_{p(i)} + PGI_{m(i)}\right) + \epsilon_{ij} \tag{7.2}$$

where $PGI_{p(i)}$ is the PGI of the father in family i; $PGI_{m(i)}$ is the PGI of the mother in family i; μ is a constant; δ is what we call the "direct effect" of the PGI and reflects the association of the individual's PGI with the phenotype through direct genetic effects (and only through direct genetic effects); α captures indirect genetic effects, gene-environment correlation, and the inflation of the correlation between phenotype and PGI due to assortative mating[71] (as discussed below); and ϵ_{ij} is the residual from this regression, which is uncorrelated with PGI_{ij} and $(PGI_{p(i)} + PGI_{m(i)})$ by construction.

The direct effect of the PGI is a function of the direct effects of genetic variants. It is not a function of indirect genetic effects, other gene-environment correlation, or assortative mating. To see why, note that the variation in an individual's PGI after conditioning on parental genotypes is due to random Mendelian segregations which are independent of environment; thus, PGI_{ij} is conditionally independent of all environmental factors given parental genotypes. Furthermore, letting $G_{p(i)}$ and $G_{m(i)}$ represent the genotype vectors of the father and mother in family i, note that

$E[PGI_{ij}|G_{p(i)}, G_{m(i)}] = (PGI_{p(i)} + PGI_{m(i)})/2$. The Conditional Independence Lemma from Young et al.[73] then implies that including $(PGI_{p(i)} + PGI_{m(i)})$ as a regressor is sufficient to control for environmental confounding, implying that the δ coefficient reflects only direct genetic effects.

Importantly, although direct effects of individual genetic variants are causal, the direct effect of the PGI does not have a straightforward causal interpretation because it includes the direct effects of genetic variants on the same chromosome that are in local linkage disequilibrium (due to physical linkage) with SNPs included in the PGI (which may not themselves have direct effects).

The coefficients in equation (7.2) can be interpreted as average effects, specifically, weighted averages over any heterogeneity of effects across individuals that may exist. In particular, δ is a weighted average over the direct effects of the PGI for the individuals in the population, unconfounded by indirect genetic effects, other gene-environment correlation, and assortative mating. However, the weights that define the weighted average can be complicated and do not necessarily correspond to a uniform weighting across individuals[72].

To derive the expected regression coefficient ψ from equation (7.1) in terms of the coefficients in equation (7.2), it is necessary to account for the increased correlation between parent and offspring PGIs due to non-random mating, due to both population structure and assortative mating. If the parental PGIs have correlation r_{am}, then the correlation between parent and offspring PGIs is $\frac{(1+r_{am})}{2}$ (as derived by Fisher[40]), and therefore $\psi = \delta + (1 + r_{am})\alpha$.

As we note in the main text, assortative mating increases the variance explained by the PGI because it induces a correlation between the PGI and the genetic component of the phenotype that would be orthogonal to the PGI in a random mating population. The degree to which α reflects assortative mating depends upon both the degree of assortative mating and the fraction of the variation in the genetic component of the phenotype captured by the PGI[69]. A useful way to understand this is to consider a PGI that is based on a certain set of chromosomes, and the genetic component that would be orthogonal to the PGI in a random-mating population is based on the remaining, complementary set of chromosomes. (This works since alleles on different chromosomes are independent in a random-mating population.) Assortative mating causes linkage disequilibrium between causal SNPs on all chromosomes. If you estimate the direct effect of the PGI, then this will only capture the inflation of the variance of the PGI based on correlations between alleles within the set of chromosomes the PGI is based on, but it will not capture the effect of the remaining genetic component on the other chromosomes, since chromosomes segregate independently within a family. The population effect of the PGI, however, captures both the inflation of variance in the PGI due to the correlations between the alleles on the chromosomes the PGI is based upon, as well as (partially) the effect of the remaining chromosomes (due to the correlations between the PGI chromosomes and the remaining chromosomes induced by assortative mating). In most real-data applications, the PGI is based on noisy estimates of the effects of genome-wide common SNPs, but the same principle applies: assortative mating implies that the population effect partially captures the effect of the genetic component of the phenotype that would be orthogonal to the PGI under random mating, whereas the direct effect does not.

7.2 Relationship to Analysis in Kong *et al.* (2018)

The regression equation (7.2) is equivalent to the regression used to estimate the direct effect of a PGI in Kong et al.[69], which regressed proband phenotype onto proband PGI and a PGI constructed from non-transmitted parental alleles. Since the transmitted and non-transmitted alleles together comprise the maternal and paternal alleles, $PGI_{p(i)} + PGI_{m(i)} = PGI_{ij} + PGI_{ij}^{NT}$, where PGI_{ij}^{NT} is the PGI constructed from the non-transmitted parental alleles. Therefore, we can express equation (7.2) as

$$Y_{ij} = \mu + (\delta + \alpha)PGI_{ij} + \alpha PGI_{ij}^{NT} + \epsilon_{ij}. \qquad (7.3)$$

The estimate of the direct effect of a PGI in Kong et al. subtracts the coefficient on the non-transmitted parental PGI, $\alpha,$ from the coefficient on the proband's PGI, $(\delta + \alpha)$. This is equivalent to the estimate of δ from performing the regression as in equation (7.2).

While not necessary for the purposes of obtaining an unbiased estimate of the direct effect of the PGI, we can consider a model where the coefficients on the paternal and maternal PGIs can differ:

$$Y_{ij} = \mu + \delta PGI_{ij} + \alpha_p PGI_{p(i)} + \alpha_m PGI_{m(i)} + \epsilon_{ij}. \qquad (7.4)$$

The coefficients α_p and α_m could differ due to differences in indirect genetic effects from mothers and fathers, for example.

In our analysis of trios, we use regression equation (7.4) rather than the Kong et al.[69] approach because the latter requires an additional step of determining parent-of-origin of alleles, which is both computationally costly and could introduce error.

7.3 Datasets and phenotypes

We used data from UK Biobank (UKB)[74], Generation Scotland (GS)[75], and the Swedish Twin Registry (STR)[76]. In both UKB and GS, first-degree relatives were identified using KING with the "--related --degree 1" option[77]. For parent-offspring relations, the parent was identified as the older individual in the pair. We removed 621 individuals from Generation Scotland that had been previously identified by Generation Scotland as being also present in UK Biobank. In UKB, we identified 34,955 individuals with genotyped siblings without both parents genotyped and 873 individuals with both parents genotyped; in GS, we identified 9,699 individuals with genotyped siblings but without both parents genotyped, and 2,674 individuals with both parents genotyped. In STR, 5,324 DZ twin pairs were available.

In these three samples, we analyzed PGIs for our main EA phenotype, as well as cognitive performance, height and BMI. In order to make the PGIs for first-degree relatives in UKB, GS and STR, we first ran GWASs for EA, cognitive performance, height and BMI in UKB that excludes all pairs of individuals who are first-degree relatives, as well as the relatives of these individuals up to third degree. Then, we meta-analyzed the EA GWAS with GWASs from all other cohorts in our current meta-analysis excluding GS, STR and UKB, and the cognitive performance, height, and BMI GWAS with the largest previously published GWAS for these phenotypes that were publicly available and did not include UKB[78–80] after applying the quality control

pipeline described in **Supplementary Note** section 2.2.5 to each file (see **Supplementary Table 16**). We made EA and cognitive performance PGIs for all three samples, and height and BMI PGIs for UKB and GS, using these summary statistics and following the LDpred PGI pipeline described in **Supplementary Note** section 5.1. We did not analyze height and BMI in STR because STR was included in the published GWAS that we used for the height and BMI meta-analyses[79,80].

We selected 24 phenotypes related to education, cognition, income, and health (**Supplementary Tables 10-13**) available in at least one of the datasets. For all phenotypes in all datasets, we first regressed the phenotype onto sex and age, age^2, and age^3, and their interactions with sex. We also used additional covariates depending on the dataset. Specifically, for UKB, we included as covariates the top 40 genetic principal components provided by UK Biobank and the genotyping array dummies[74]. For GS and STR, we included the top 20 genetic principal components (see **Supplementary Note** section 5.3 for a description of how the principal components were created). For each phenotype in all datasets, we then took the residuals from the regression of the phenotype on the covariates and normalized the residuals' variance within each sex separately, so that the phenotypic residual variance was 1 in each sex in the combined sample of siblings and individuals with both parents genotyped. The PGIs of the phenotyped individuals were also normalized to have variance 1 in the same sample. This way, effect estimates correspond to (partial) correlations, and their squares to proportions of phenotypic variance explained.

7.4 Statistical Methods

Unbiased estimates of direct genetic effects can be derived from samples of siblings and from samples with genotypes of both parents available[2,69,71]. In both cases, the unbiasedness of direct-effect estimates derives from the fact that genetic variation within a family is generated by random segregation in the parents during meiosis, which is uncorrelated with environment. We therefore analyzed individuals with at least one genotyped sibling and/or both parents genotyped. For individuals with at least one sibling and both parents genotyped, we analyzed them using the method for individuals with both parents genotyped, since this produces more precise estimates of effects than using sibling differences[81].

We estimated effects in each dataset, splitting each dataset into non-overlapping subsamples: those with at least one genotyped sibling but not both parents genotyped, and those with both parents genotyped.

For UKB and GS, the steps of the statistical estimation were:

Step 1: Separately estimate effects in the subsample of siblings and the subsample of individuals with both parents genotyped.

Step 2: Compute the sampling covariance matrix of the combined vector of effects from siblings and trios.

Step 3: Estimate the correlation between maternal and paternal PGI in each dataset.

For STR, we did not have trios, so we simply computed the estimated coefficient vector and its sampling covariance matrix from the sample of DZ twins.

Then, given estimated coefficient vectors and their sampling covariance matrices from UKB, GS, and STR, we:

Step 4. Transform the vector of estimates and combine them across the three samples into a meta-analysis estimate of $[\delta, \alpha_p, \alpha_m]$ (and its sampling covariance matrix).

Step 5: Use these to calculate meta-analysis estimates of δ, $\delta + (1 + r_{am})(\alpha_p + \alpha_m)/2$, and $\alpha_m - \alpha_p$ (and their sampling covariance matrix).

We discuss these steps in more detail below.

7.4.1 Step 1: Estimating effects in the different subsamples

Estimating effects when both parents are genotyped

For individuals with both parents genotyped, as in (7.4) we regress the phenotype of each individual jointly onto the individual's PGI and the individual's father's and mother's PGI:

$$Y_{ij} \sim PGS_{ij} + PGS_{p(i)} + PGS_{m(i)}.$$

In this case, the expected coefficient vector is $[\delta, \alpha_p, \alpha_m]$.

Estimating effects from siblings

Let n_i be the number of siblings in family i. For each family, we estimate the mean PGI among the siblings: $\overline{PGS_i} = \frac{1}{n_i}\sum PGS_{ij}$. We regress the phenotype of each individual onto the deviation of that individual's PGI from the family mean, and the family mean PGI:

$$Y_{ij} \sim (PGS_{ij} - \overline{PGS_i}) + \overline{PGS_i}.$$

In this case, the expected coefficient vector is $[\delta, \delta + c(\alpha_p + \alpha_m)]$, where

$$c = (1 + \frac{1 - r_{am}}{1 + r_{am}} \mathrm{E}[n_i^{-1}])^{-1},$$

which can be calculated from the distribution of the number of siblings in each family. This coefficient depends upon the distribution of the number of genotyped siblings in each family in the sample because, while the covariance of the mean PGI in the family with individual siblings' phenotypes is constant and captures both direct and parental effects, the variance of the mean PGI decreases with the number of siblings in each family. See **Supplementary Note** section 9 for a derivation.

7.4.2 Step 2: Computing the sampling covariance matrix of the combined vector of effects

Here we detail the regression method used for the UKB and GS datasets, where both siblings and trios are present and the relatedness structure is complex. For the STR, where we only had DZ twins available, we used a simpler approach that models phenotypic correlations between siblings, as detailed elsewhere[81,82].

In order to account for relatedness, we used a linear mixed model that captures phenotypic covariance due to genetic effects and shared environment. In order to do this, we followed previous work[83] by partitioning the relatedness matrix estimated from genome-wide SNPs into two relatedness matrices: one with off-diagonal elements less than 0.025 set to zero, R_u; and one with off-diagonal elements greater than 0.025 set to zero, R_l. We further include a matrix that models shared environmental effects between

siblings, C_{sib}, where off-diagonal elements are 1 for pairs that are siblings, and are zero otherwise. Let Y be the column vector of phenotype observations in a dataset, partitioned as $Y = [Y_s^T, Y_t^T]^T$, where Y_s^T is the vector of observations for individuals with genotyped siblings but not both parents genotyped, and Y_t^T is the vector of observations for individuals with both parents genotyped. We first infer the variance components in a linear mixed model with the PGI as a fixed effect:

$$Y|\text{PGI} \sim N\left(\psi\text{PGI}, h_{\text{l}}^2 R_l + h_{\text{u}}^2 R_u + c_{\text{sib}}^2 C_{sib} + \sigma_\epsilon^2 \text{I}\right),$$

where PGI is the vector of individuals' PGI values; ψ is the population effect of the PGI; h_{l}^2 is the SNP heritability remaining after accounting for the variance captured by the PGI; h_{u}^2 is the remaining phenotypic variance that is shared in proportion to relatedness; and c_{sib}^2 captures any additional phenotypic variance shared between siblings. We infer the variance components by REML in *GCTA*[23]. Let Σ be the covariance matrix inferred by REML. We can partition Σ as

$$\Sigma = \begin{bmatrix} \Sigma_s & \Sigma_{st} \\ \Sigma_{ts} & \Sigma_t \end{bmatrix}.$$

Let X_s be the matrix of covariates for the sibling sample, including the deviation of the individuals' PGI from the family mean and the family mean PGI; and let X_t be the matrix of covariates for the sample with both parents genotyped. We then estimate the effects in each subsample as the generalized-least-squares estimates using Σ as the covariance matrix of the regression residuals:

$$\hat{\theta}_s = (X_s^T \Sigma_s^{-1} X_s)^{-1} X_s^T \Sigma_s^{-1} Y_s \text{ with } \text{Var}\left(\hat{\theta}_s\right) = (X_s^T \Sigma_s^{-1} X_s)^{-1};$$

$$\hat{\theta}_t = (X_t^T \Sigma_t^{-1} X_t)^{-1} X_t^T \Sigma_t^{-1} Y_t \text{ with } \text{Var}\left(\hat{\theta}_t\right) = (X_t^T \Sigma_t^{-1} X_t)^{-1}.$$

Further, we have that

$$\text{Cov}(\hat{\theta}_s, \hat{\theta}_t) = (X_s^T \Sigma_s^{-1} X_s)^{-1} X_s^T \Sigma_s^{-1} \Sigma_{st} \Sigma_t^{-1} X_t (X_t^T \Sigma_t^{-1} X_t)^{-1}.$$

7.4.3 Step 3: Estimating the correlation between maternal and paternal PGI

This is trivial when both maternal and paternal PGI are observed. For the sibling data, we estimate r_{am} from the variance in mean PGI between families. From **Supplementary Note** section 9, we have that

$$\text{Var}(\overline{PGI_\iota}) = \text{E}[n_\iota^{-1}] + (1 - \text{E}[n_\iota^{-1}])(1 + r_{am})/2.$$

Solving this for r_{am}, we obtain our estimator:

$$\hat{r}_{am} = \frac{2\widehat{Var(\overline{PGI})} - (1 + \widehat{E[n_\iota^{-1}]})}{1 - \widehat{E[n_\iota^{-1}]}}.$$

As our estimate of r_{am} in each dataset, we took the weighted average of the estimates of r_{am} from the siblings and parents, weighted by the number of families. For the EA PGI, we estimated r_{am} to be 0.190, 0.182, and 0.175 in UKB, GS, and STR respectively. We took the average of these estimates, 0.182, to use for the meta-analysis estimates. For the height PGI, we estimated r_{am} to be 0.100 and 0.099 in UKB and GS respectively. For the BMI PGI, we estimated r_{am} 0.035 and 0.026 in UKB and GS respectively. For the cognition PGI, we estimated r_{am} to be 0.053, 0.024, and 0.019 in UKB, GS, and STR respectively.

7.4.4 *Step 4. Meta-analyzing estimates*

In each subsample in each dataset, the expected regression coefficients are linear transformations of the underlying parameter vector, $\theta = [\delta, \alpha_p, \alpha_m]$. We therefore have that, for some constant matrix A_k, $\hat{\theta}_k$ has distribution

$$\hat{\theta}_k \sim N\left(A_k\theta, \mathrm{Var}(\hat{\theta}_k)\right),$$

where $\mathrm{Var}(\hat{\theta}_k)$ is the known sampling covariance matrix of $\hat{\theta}_k$.

Here, we have parameter estimates from two subsamples from UKB and GS, and the siblings from STR, which we label dataset 3. Let $\hat{\theta}_{ij}$ be the estimate from subsample j in dataset i, and let A_{ij} be such that $E[\hat{\theta}_{ij}] = A_{ij}\theta$. We assume that estimates from different datasets are independent, so we have

$$\hat{\theta} \sim N(A\theta, S),$$

where $A = \begin{bmatrix} A_s \\ A_t \\ A_s \\ A_t \\ A_s \end{bmatrix}$; $\hat{\theta} = \begin{bmatrix} \hat{\theta}_{1s} \\ \hat{\theta}_{1t} \\ \hat{\theta}_{2s} \\ \hat{\theta}_{2t} \\ \hat{\theta}_{3s} \end{bmatrix}$; $A_s = \begin{bmatrix} 1 & 0 & 0 \\ 1 & c & c \end{bmatrix}$; $A_t = \begin{bmatrix} 1 & 0 & 0 \\ 0 & 1 & 0 \\ 0 & 0 & 1 \end{bmatrix}$; and

$$S = \begin{bmatrix} \mathrm{Var}(\hat{\theta}_{1s}) & \mathrm{Cov}(\hat{\theta}_{1s}, \hat{\theta}_{1t}) & 0 & 0 & 0 \\ \mathrm{Cov}(\hat{\theta}_{1t}, \hat{\theta}_{1s}) & \mathrm{Var}(\hat{\theta}_{1t}) & 0 & 0 & 0 \\ 0 & 0 & \mathrm{Var}(\hat{\theta}_{2s}) & \mathrm{Cov}(\hat{\theta}_{2s}, \hat{\theta}_{2t}) & 0 \\ 0 & 0 & \mathrm{Cov}(\hat{\theta}_{2t}, \hat{\theta}_{2s}) & \mathrm{Var}(\hat{\theta}_{2t}) & 0 \\ 0 & 0 & 0 & 0 & \mathrm{Var}(\hat{\theta}_{3s}) \end{bmatrix}.$$

We therefore have that the MLE for θ is $(A^T S^{-1} A)^{-1}(A^T S^{-1}\hat{\theta})$ with sampling covariance matrix $(A^T S^{-1} A)^{-1}$.

7.4.5 *Step 5. Further transforming the estimates*

Finally, we transform the MLE for θ by a matrix

$$B = \begin{bmatrix} 1 & 0 & 0 \\ 1 & (1 + r_{am})/2 & (1 + r_{am})/2 \\ 0 & (1 + r_{am})/2 & (1 + r_{am})/2 \\ 0 & -1 & 1 \end{bmatrix}.$$

The resulting matrix $B\theta$ gives, respectively: meta-analysis estimates of the direct effect, population effect, the difference between the population and the direct effects, and the difference between the maternal and paternal effects, along with their sampling variances. This allows us to perform Z-tests for differences between population and direct-effect estimates, and for differences between maternal and paternal effects.

7.4.6 Supplementary Results

For the EA PGI, we detected differences between direct and population effects for EA, AAFB (women), vocabulary, cognitive performance, hourly income, household income, BMI, self-rated health, height, HDL, and FEV1 at a Bonferroni-adjusted significance threshold of $\frac{0.005}{23} = 2.2 \times 10^{-4}$. The EA PGI explains 5.95% of the variance in cognitive performance. For predicting cognitive performance, the ratio of direct to population effects is 0.656 (S.E. = 0.041)—larger than for predicting EA—implying that direct effects account for 43.1% of the overall variance explained by the EA PGI. Vocabulary, hourly income and household income show a similar ratio of direct to population effects to both EA and cognitive performance. Across metabolic-related phenotypes (BMI, HDL, SBP, DBP, non-HDL cholesterol, blood glucose) the EA PGI explains on average 0.56% of the variance in the phenotypes. The inverse-variance-weighted average ratio between direct and population effects for the metabolic-related phenotypes is 0.645 (S.E. = 0.013). For other health-related phenotypes, the pattern is less clear: direct and population effects are not statistically distinguishable from each other for ever-smoked, drinks per week, and cigarettes per day. For FEV1, the ratio of direct to population effect estimates is only 0.184, implying that only around 3.4% of the overall variance in FEV1 explained by the EA PGI is due to direct genetic effects.

For the height PGI (**Supplementary Figure 2a**), we detected differences between direct and population effects for height, FEV1, and EA at a Bonferroni-adjusted significance threshold of $\frac{0.005}{23} = 2.2 \times 10^{-4}$.

For the BMI PGI (**Supplementary Figure 2b**), we cannot statistically detect a difference between direct and population effects on BMI, consistent with a negligible influence of assortative mating and indirect genetic effects for BMI. At a Bonferroni-adjusted significance threshold of $\frac{0.005}{23} = 2.2 \times 10^{-4}$, we detect differences between the direct and population effects of the BMI PGI for 9 of the phenotypes: systolic blood pressure (SBP), height, cognitive performance, AAFB (women), household income, EA, hourly income, vocabulary, and FEV1. While it is possible that cross-phenotype assortative mating could explain these differences, this seems unlikely given the limited strength of assortative mating with respect to BMI itself. The difference between direct and population effects of the BMI PGI on SES-related phenotypes could reflect SES-related stratification in the BMI PGI. However, this would have to be a type of stratification that does not have much of an impact on the correlation of the BMI PGI with BMI. Indirect genetic effects could explain this gap if the BMI PGI captures aspects of parental behavior that influence offspring SES but not offspring BMI.

For the cognitive performance PGI (**Supplementary Figure 2c**), we detected differences between direct and population effects for cognitive performance, vocabulary, EA, hourly income, household income, self-rated health, height, and BMI at a Bonferroni-adjusted significance threshold of $\frac{0.005}{23} = 2.2 \times 10^{-4}$. While we find a difference between the population and direct effects of the cognitive performance PGI on cognitive performance, the ratio of direct to population effect estimates is 0.824 (S.E. = 0.033), much larger than for the ratio for the effect of the EA PGI on EA (0.556). This implies that the large differences we observe between the EA PGI direct and population effects cannot be explained by the effects of cognitive performance on educational attainment. Further, the ratio of direct to population effects of the cognitive

performance PGI on EA is 0.526 (S.E. = 0.030), similar to the ratio for the effect of the EA PGI on EA. This suggests that the genetic variants correlated with cognitive performance are correlated with EA through pathways other than through cognitive performance, including through the family environment and/or through population stratification and assortative mating.

We now make a general comparison of between the PGIs in terms of the contribution of their direct effects to cross-phenotype prediction. The inverse-variance-weighted average ratio of direct to population effects for the height PGI on the non-height phenotypes is 0.721 (S.E. = 0.030); for the BMI PGI on the non-BMI phenotypes, 0.592 (S.E. = 0.024); and for the cognitive performance PGI on the non-cognitive-performance phenotypes, 0.554 (S.E. = 0.021). Thus, unlike for the EA PGI, for these PGIs the ratio of direct to population effects—and thus also the fraction of the PGI's predictive power that is due to direct effects—drops substantially when predicting phenotypes other than the phenotype from which the PGI was derived.

8 Empirical Bayesian model and GWAS replication simulation framework

Our study involved two main GWAS of educational attainment: an additive GWAS, which yields estimates of the "average effect of allele substitution" α for each SNP by regressing educational attainment on allele dosage x, and a dominance GWAS, which yields estimates of the parameter d (as well as a, which we do not analyze). Although our estimators of the parameters α_j and d_j for SNP j are unbiased (assuming there is no confounding), the actual estimates $\hat{\alpha}_j$ and $\hat{d}_j$ will tend to be too large in magnitude, due to the Winner's Curse (as explained in Okbay *et al.* (2016)[1] in the context of additive GWAS). Intuitively, conditional on an estimate being positive (for example), the expectation of the sampling error in that estimate is also positive, and so the expected true value of the estimated parameter is smaller than the estimate.

This Appendix describes the empirical Bayesian model we use to compute the posterior distribution of the SNPs' true α and d, which allows us to account for the Winner's Curse when computing the SNPs' expected replication record. This Appendix also describes the simulation framework we use to benchmark some internal replications we conduct of our GWAS results.

We continue to use following notation in this section. We let $x_j \in \{0,1,2\}$ denote allele dosage at SNP j; h_j be a dummy variable that is equal to 1 if $x = 1$ (and to 0 otherwise); and $x_{j,\text{std}}$ and $h_{j,\text{std}}$ denote x_j and h_j standardized to have mean 0 and variance 1, respectively.

8.1 Posterior distribution of α_j

Our presentation of the empirical Bayesian model closely follows Supplementary Note section 4 of the Supplementary Information of Karlsson Linnér *et al.* (2019)[28], which in turn follows section 8 of the Supplementary Note of Okbay *et al.* (2016)[1].

Let $\alpha_{j,\text{std}}$ be the coefficient from a regression of y_{std} on $x_{j,std}$ (and controls, which we will ignore here for simplicity), where y_{std} denotes the standardized phenotype and $x_{j,std}$ denotes the standardized dosage for SNP j. Throughout this Appendix, we assume that y has been standardized and work with y_{std}, unless otherwise noted. We assume that $\alpha_{j,\text{std}}$ is drawn from a mixture distribution of a Gaussian and a point mass at zero:

$$\alpha_{j,\text{std}} \sim \begin{cases} N(0,\tau^2) & \text{with probability } \pi \\ 0 & \text{otherwise,} \end{cases} \tag{8.1}$$

where τ^2 is the variance of SNPs with a nonzero additive effect size (i.e., $\alpha_j \neq 0$) and π is the fraction of SNPs with a nonzero additive effect size. Henceforth, we will call such SNPs with a nonzero additive effect size "nonnull additive SNPs."

Observe that if we let α_j be the coefficient from a regression of y_{std} on (unstandardized) dosage x_j, then $\alpha_j = \alpha_{j,\text{std}} / \sqrt{\text{Var}(x_j)}$. Our distributional assumption thus implies that $\text{Var}(\alpha_j | \alpha_j \neq 0) = \tau^2 / \text{Var}(x_j)$, i.e, that the variance of the additive effect size α_j is inversely proportional to the variance of the dosage x_j.

Let σ_j^2 denote the variance of the estimation error of the estimate of $\alpha_{j,\text{std}}$. By the Central Limit Theorem, that estimation error is approximately normally distributed. It follows that the distribution of $\hat{\alpha}_{j,\text{std}}$ is given by:

$$\hat{\alpha}_{j,\text{std}} \sim \begin{cases} N\left(0, \tau^2 + \sigma_j^2\right) & \text{with probability } \pi \\ N(0, \sigma_j^2\ ) & \text{otherwise.} \end{cases} \quad (8.2)$$

Okbay *et al.* (2016)[1] show that, given an estimate $\hat{\alpha}_{j,\text{std}}$ of a SNP j's additive effect size, the implied posterior probability that the SNP is a nonnull additive SNP is:

$$p_{\hat{\alpha}_j} = \frac{\frac{\pi}{\sqrt{\tau^2 + \sigma_j^2}} \phi\left(\frac{\hat{\alpha}_{j,\text{std}}}{\sqrt{\tau^2 + \sigma_j^2}}\right)}{\frac{1-\pi}{\sigma_j} \phi\left(\frac{\hat{\alpha}_{j,\text{std}}}{\sigma_j}\right) + \frac{\pi}{\sqrt{\tau^2 + \sigma_j^2}} \phi\left(\frac{\hat{\alpha}_{j,\text{std}}}{\sqrt{\tau^2 + \sigma_j^2}}\right)}. \quad (8.3)$$

Also, the posterior distribution for a nonnull additive SNP is:

$$\left(\alpha_{j,\text{std}} | \hat{\alpha}_{j,\text{std}}, \alpha_j \neq 0\right) \sim N\left(\frac{\tau^2}{\tau^2 + \sigma_j^2} \hat{\alpha}_{j,\text{std}}, \frac{\tau^2 \sigma_j^2}{\tau^2 + \sigma_j^2}\right). \quad (8.4)$$

To estimate the posterior distribution $\left(p_{\hat{\alpha}_j}, \left(\alpha_{j,\text{std}} | \hat{\alpha}_{j,\text{std}}, \alpha_j \neq 0\right)\right)$, we first need to obtain estimates of π and τ^2. To do so, we observe that $\sigma_j^2 \approx \text{Var}\left(y_j\right) / (N_j \text{ Var}(x_{j,std})) = 1/N_j$, and use Maximum Likelihood Estimation (MLE) to fit equation (8.2) to the unadjusted (see **Supplementary Note** section 8.5) summary statistics from the relevant GWAS of additive variance.

8.2 Posterior distribution of d_j

We also estimate the posterior distribution of d_j. To do so, we need to make adjustments to the above empirical Bayes framework for the posterior distribution of α_j. The main adjustment concerns the counterpart for d_j of the distributional assumption we made for α_j, which is summarized in (8.4). As mentioned above, that assumption implies that the variance of the additive effect size α_j is inversely proportional to the variance of the dosage x_j. Such an assumption may be biologically reasonable and is commonly made in the literature (see, e.g., ref. [20]), but it is not obvious what the appropriate, biologically relevant counterpart is for d_j.

Here, we assume that

$$d_{j,\text{std}} \sim \begin{cases} N(0, \kappa^2) & \text{with probability } \omega \\ 0 & \text{otherwise,} \end{cases} \quad (8.5)$$

where $d_{j,\text{std}}$ is the coefficient on $h_{j,\text{std}}$ from a regression of y_{std} on x_j and $h_{j,\text{std}}$ (and controls, which we will again ignore here for simplicity), where, as above, $h_{j,\text{std}}$ is the heterozygosity indicator h_j standardized to have mean 0 and variance 1. κ^2 is the variance of "nonnull dominance SNPs" (i.e., the SNPs for which $d_j \neq 0$) and ω is the fraction of SNPs that are nonnull dominance SNPs. We maintain the convention that y has been standardized and work with y_{std}.

The distributional assumption (8.5) implies that $\text{Var}(d_j | d_j \neq 0) = \kappa^2/\text{Var}(h_j)$, i.e., that the variance of the dominance parameter d_j is inversely proportional to the variance of the heterozygosity indicator h_j, and is thus similar to assumption (8.1). Because $\text{Var}(x_j) = 2p_j q_j$, assumption (8.1) implies that $\text{Var}(\alpha_j | \, \alpha_j \neq 0) = \tau^2/2p_j q_j$ (where p_j denotes the minor allele frequency of SNP j and $q_j = 1 - p_j$.) And because $\text{Var}(h_j) = 2p_j q_j(1 - 2p_j q_j)$ (since h_j is an indicator that equals one with probability $2p_j q_j$), assumption (8.5) implies that $\text{Var}(d_j | d_j \neq 0) = \kappa^2/(2p_j q_j(1 - 2p_j q_j))$.

The Central Limit Theorem implies that estimation error of the estimate of $d_{j,\text{std}}$ is approximately normally distributed, so it follows that the distribution of $\hat{d}_{j,\text{std}}$ is given by:

$$\hat{d}_{j,\text{std}} \sim \begin{cases} N(0, \kappa^2 + \sigma_{d,j}^2) & \text{with probability } \omega \\ N(0, \sigma_{d,j}^2) & \text{otherwise.} \end{cases} \tag{8.6}$$

Having specified a distribution for d_j, we obtain direct counterparts to equations (8.3) and (8.4) above. The implied posterior probability that the SNP is a nonnull dominance SNP is:

$$p_{\hat{d}_j} = \frac{\frac{\omega}{\sqrt{\kappa^2 + \sigma_{d,j}^2}} \phi\left(\frac{\hat{d}_{j,\text{std}}}{\sqrt{\kappa^2 + \sigma_{d,j}^2}}\right)}{\frac{1-\omega}{\sigma_{d,j}} \phi\left(\frac{\hat{d}_{j,\text{std}}}{\sigma_{d,j}}\right) + \frac{\omega}{\sqrt{\kappa^2 + \sigma_{d,j}^2}} \phi\left(\frac{\hat{d}_{j,\text{std}}}{\sqrt{\kappa^2 + \sigma_{d,j}^2}}\right)},$$

and the posterior distribution for a nonnull dominance SNP is:

$$(d_{j,\text{std}} | \hat{d}_{j,\text{std}}, d_j \neq 0) \sim N\left(\frac{\kappa^2}{\kappa^2 + \sigma_{d,j}^2} \hat{d}_{j,\text{std}}, \frac{\kappa^2 \sigma_{d,j}^2}{\kappa^2 + \sigma_{d,j}^2}\right),$$

where $\sigma_{d,j}^2$ is the variance of the estimation error of the estimate of $d_{j,\text{std}}$. As we show below, $\sigma_{d,j}^2 = \frac{1 - 2p_j q_j}{N_j 2p_j q_j}$.

We would like to estimate ω and κ^2 in equation (8.6) using MLE applied to the unadjusted summary statistics from the relevant dominance GWAS, analogously to what we do to estimate π and τ^2 for the posterior distribution of α_j. However, the MLE algorithm fails to converge for equation (8.6), likely because κ^2 is much smaller than $\sigma_{d,j}^2$, as we show **Supplementary Note** section 2.5.

For that reason, we do not estimate ω and κ^2 jointly, but rather obtain estimates of κ^2 implied various assumed values of ω and then compute the expected records of the planned replication exercises for these ω's and the implied κ^2's.

To obtain κ^2 for a given ω, we note that the Law of Total Variance implies that $\mathrm{Var}(\hat{d}_{j,\mathrm{std}}) = \mathrm{E}[\mathrm{Var}(\hat{d}_{j,\mathrm{std}}|p_j, N_j)] + \mathrm{Var}(\mathrm{E}[\hat{d}_{j,\mathrm{std}}|p_j, N_j])$, where the variances and expectations are taken across the SNPs. Equation (8.6) implies that $\mathrm{E}[\hat{d}_{j,\mathrm{std}}|p_j, N_j] = 0$ for all p_j and N_j and that $\mathrm{Var}(\hat{d}_{j,\mathrm{std}}|p_j, N_j) = \omega\kappa^2 + \sigma^2_{d,j}$. It follows that $\mathrm{Var}(\hat{d}_{j,\mathrm{std}}) = \omega\kappa^2 + \mathrm{E}[\sigma^2_{d,j}]$, and thus that $\kappa^2 = (\mathrm{Var}(\hat{d}_{j,\mathrm{std}}) - \mathrm{E}[\sigma^2_{d,j}])/\omega$. Our estimator of κ^2 for a given assumed value of ω is

$$\hat{\kappa}^2 = (\widehat{\mathrm{Var}}(\hat{d}_{j,\mathrm{std}}) - \hat{\mathrm{E}}[\sigma^2_{d,j}])/\omega,$$

where $\widehat{\mathrm{Var}}$ and $\hat{\mathrm{E}}$ denote the sample variance and mean taken across the SNPs' summary statistics[23].

8.3 Proof that $\sigma^2_{d,j} = (1 - 2p_j q_j)/N_j 2p_j q_j$

We now show that $\sigma^2_{d,j} = \frac{1-2p_jq_j}{N_j 2p_j q_j}$, where $\sigma^2_{d,j}$ is the variance of the estimation error of the estimate of $d_{j,\mathrm{std}}$, where $d_{j,\mathrm{std}}$ is the coefficient on $h_{j,std}$ from a regression of y_{std} on x_j and $h_{j,std}$. For this derivation, we assume that every genotype is imputed perfectly and that there is no assortative mating.

Step 1: regression of unstandardized phenotype on unstandardized dosage and unstandardized heterozygosity dummy

We first derive the variance of d_j. Recall that d_j is the coefficient on h_j in a regression of (unstandardized) y on (unstandardized) x_j and (unstandardized) h_j (and controls, which we ignore here for simplicity). The variance of d_j will depend on the variances of x_j and h_j and their covariance, so we first compute these. We know that $\mathrm{Var}(x_j) = 2p_jq_j$, and since h_{ij} is a binary variable with probability $2p_jq_j$ of being equal to 1, $\mathrm{Var}(h_{ij}) = 2p_jq_j(1 - 2p_jq_j)$. Finally,

$$\mathrm{Cov}(x_{ij}, h_{ij}) = \mathrm{E}[x_jh_j] - \mathrm{E}[x_j]\mathrm{E}[h_j] = 2p_jq_j - (2p_j)(2p_jq_j) = 2p_jq_j(1 - 2p_j).$$

Next, observe that

$$\mathrm{Var}\left(\begin{bmatrix}\hat{\alpha}_j \\ \hat{d}_j\end{bmatrix}\right) \approx \frac{\sigma^2_y}{N_j}\begin{bmatrix}\mathrm{Var}(x_j) & \mathrm{Cov}(x_j, h_j) \\ & \mathrm{Var}(h_j)\end{bmatrix}^{-1},$$

where we use the approximation $\sigma^2_{\varepsilon_j} \approx \sigma^2_y$ since the R^2 of the regression is very small. With some algebra, we see that

$$\begin{bmatrix}\mathrm{Var}(x_j) & \mathrm{Cov}(x_j, h_j) \\ & \mathrm{Var}(h_j)\end{bmatrix}^{-1} = \begin{bmatrix}2p_jq_j & 2p_jq_j(1 - 2p_j) \\ & 2p_jq_j(1 - 2p_jq_j)\end{bmatrix}^{-1}$$

[23] As above for the estimation of π and τ^2 and as we further discuss below in **Supplementary Note** section 8.5, we use the unadjusted summary statistics to estimate $\hat{\kappa}^2$.

$$= \frac{1}{2p_jq_j}\begin{bmatrix}1 & 1-2p_j \\ & 1-2p_jq_j\end{bmatrix}^{-1}$$

$$= \frac{1}{2p_jq_j}\left(\frac{1}{1-2p_jq_j-\left(1-2p_j\right)^2}\right)\begin{bmatrix}1-2p_jq_j & 2p_j-1 \\ & 1\end{bmatrix}$$

$$= \frac{1}{2p_jq_j}\left(\frac{1}{1-2p_jq_j-1+4p_j-4{p_j}^2}\right)\begin{bmatrix}1-2p_jq_j & 2p_j-1 \\ & 1\end{bmatrix}$$

$$= \frac{1}{2p_jq_j}\left(\frac{1}{2p_jq_j}\right)\begin{bmatrix}1-2p_jq_j & 2p_j-1 \\ & 1\end{bmatrix}$$

$$= \frac{1}{\left(2p_jq_j\right)^2}\begin{bmatrix}1-2p_jq_j & 2p_j-1 \\ & 1\end{bmatrix}.$$

It follows that

$$\mathrm{Var}\left(\begin{bmatrix}\hat{\alpha}_j \\ \hat{d}_j\end{bmatrix}\right) \approx \frac{\sigma_y^2}{N_j\left(2p_jq_j\right)^2}\begin{bmatrix}1-2p_jq_j & 2p_j-1 \\ & 1\end{bmatrix},$$

and in particular that $\mathrm{Var}\left(\hat{d}_j\right) \approx \frac{\sigma_y^2}{N_j\left(2p_jq_j\right)^2}$.

Step 2: regression of standardized phenotype on unstandardized dosage and standardized heterozygosity indicator

Next, we derive the variance of $d_{j,\mathrm{std}}$, the coefficient on $h_{j,\mathrm{std}}$ in a regression of (standardized) y_{std} on (unstandardized) x_j and (standardized) $h_{j,\mathrm{std}}$ (and controls, which we will ignore here for simplicity). We have $\sigma_{\varepsilon_j}^2 \approx \sigma_y^2 = 1$, and

$$h_{j,\mathrm{std}} = \frac{h_j-\widehat{\mathrm{E}}\left[h_j\right]}{\sqrt{\widehat{\mathrm{Var}}\left(h_j\right)}} = \frac{h_j-2p_jq_j}{\sqrt{2p_jq_j\left(1-2p_jq_j\right)}} \quad \text{and} \quad \mathrm{Cov}\left(x_j, h_{j,\mathrm{std}}\right)$$

$$= \frac{\mathrm{Cov}\left(x_j, h_j\right)}{\sqrt{2p_jq_j\left(1-2p_jq_j\right)}}.$$

It follows that

$$\mathrm{Var}\left(\begin{bmatrix}\hat{\alpha}_j \\ \hat{d}_{j,\mathrm{std}}\end{bmatrix}\right) \approx \frac{1}{N_j}\begin{bmatrix}\mathrm{Var}\left(x_j\right) & \mathrm{Cov}\left(x_j, h_{j,\mathrm{std}}\right) \\ & \mathrm{Var}\left(h_{j,\mathrm{std}}\right)\end{bmatrix}^{-1}$$

$$= \frac{1}{N_j}\left(\begin{bmatrix}1 & 0 \\ & \frac{1}{\sqrt{2p_jq_j\left(1-2p_jq_j\right)}}\end{bmatrix}\begin{bmatrix}\mathrm{Var}\left(x_j\right) & \mathrm{Cov}\left(x_j, h_j\right) \\ & \mathrm{Var}\left(h_j\right)\end{bmatrix}\begin{bmatrix}1 & 0 \\ & \frac{1}{\sqrt{2p_jq_j\left(1-2p_jq_j\right)}}\end{bmatrix}\right)^{-1}$$

$$= \frac{1}{N_j}\begin{bmatrix}1 & 0 \\ & \sqrt{2p_jq_j\left(1-2p_jq_j\right)}\end{bmatrix}$$

$$\begin{bmatrix}\mathrm{Var}\left(x_j\right) & \mathrm{Cov}\left(x_j, h_j\right) \\ & \mathrm{Var}\left(h_j\right)\end{bmatrix}^{-1}\begin{bmatrix}1 & 0 \\ & \sqrt{2p_jq_j\left(1-2p_jq_j\right)}\end{bmatrix}$$

$$= \frac{1}{N_j}\begin{bmatrix}1 & 0 \\ & \sqrt{2p_jq_j\left(1-2p_jq_j\right)}\end{bmatrix}$$

$$\left[\frac{1}{\left(2p_jq_j\right)^2}\begin{bmatrix}1-2p_jq_j & 2p_j-1\\ & 1\end{bmatrix}\right]\begin{bmatrix}1 & 0\\ & \sqrt{2p_jq_j\left(1-2p_jq_j\right)}\end{bmatrix}$$

$$=\frac{1}{N_j\left(2p_jq_j\right)^2}\begin{bmatrix}1-2p_jq_j & \left(2p_j-1\right)\sqrt{2p_jq_j\left(1-2p_jq_j\right)}\\ & 2p_jq_j\left(1-2p_jq_j\right)\end{bmatrix}.$$

This implies that $\sigma_{d,j}^2 = \mathrm{Var}\left(\hat{d}_{j,\text{std}}\right) \approx \frac{2p_jq_j(1-2p_jq_j)}{N_j\left(2p_jq_j\right)^2} = \frac{2p_jq_j-\left(2p_jq_j\right)^2}{N_j\left(2p_jq_j\right)^2} = \frac{1-2p_jq_j}{N_j2p_jq_j}$.

8.4 GWAS replication simulation framework

We now describe the framework we used to simulate and benchmark internal replications of our additive and dominance GWAS results. This framework was also used in Supplementary Note section 4 of the Supplementary Information of Karlsson Linnér *et al.* (2019)[28]. In the current paper, we used this framework in **Supplementary Note** section 2.3 to benchmark a replication of the results from our earlier EA3 GWAS of additive variance (Lee *et al.* 2018[2]) in the new data from the current paper, and in **Supplementary Note** section 2.6 to benchmark a cross-cohort replication of the results of the GWAS of dominance variance across the 23andMe and UKB cohorts.

Suppose we wish to assess how well the effect-size estimates obtained for a set of "lead SNPs" in a discovery dataset replicate in a replication dataset. Let θ_j denote the true effect size for SNP j, and define a test T of whether the rescaled estimate $\hat{\theta}_{j,\text{std[rep]}}$ from the replication data is consistent with the rescaled estimate $\hat{\theta}_{j,\text{std[disc]}}$ from the discovery data. (In practice, θ_j is either the additive effect α_j or the dominance effect d_j, depending on the replication; the test T may be, for example, whether $\hat{\theta}_{j,\text{std[repln]}}$ has the same sign as $\hat{\theta}_{j,\text{std[disc]}}$, or the same sign plus a P value less than a certain threshold.) The lead SNPs are obtained using our clumping algorithm (**Supplementary Note** section 2.2.6) and are thus approximately independent from one another. For each lead SNP from the discovery data, we assess whether the estimate in the replication data passes test T.

We note that, consistent with the way we define the genome-wide-significant lead SNPs in this paper, the lead SNPs in our replications are determined using their adjusted P values in the discovery data (i.e., P values calculated using standard errors that have been inflated by the square root of the LD score regression). This only impacts the set of SNPs included in our replication exercises, and thus in the simulations. Our replication tests also use the SNPs' adjusted P values (in the replication data), and as described below, we adjust the simulated standard errors accordingly. However, in our simulations, the relevant posterior distribution of $\theta_{j,\text{std}}$ is estimated using the unadjusted summary statistics, as we further discuss in **Supplementary Note** section 8.5 below.

To compute $\hat{E}[C_T]$, where C_T is the number of lead SNPs that pass test T, we proceed as follows.

We simulate 1,000 estimates of $\theta_{j,\text{std}}$ in the replication data for each lead SNP j. We generate the k-th simulation estimate of $\theta_{j,\text{std}}$ by adding Gaussian noise to a "true" $\theta_{j,\text{std}}$ drawn from the relevant posterior distribution:

$$\hat{\theta}_{j,\mathrm{std},k[\mathrm{rep}]} = \theta_{j,\mathrm{std},k} + \delta_{j,k}\hat{\sigma}_{\theta,j[\mathrm{rep}]}\sqrt{\mathrm{Intercept}_{\mathrm{LD\ score}}},$$

where $\delta_{j,\mathrm{k}}$ is an independent draw from a standard normal distribution, $\hat{\sigma}_{\theta,j[\mathrm{rep}]}$ is the standard error of the estimates of $\theta_{j,\mathrm{std}}$ in the replication data, and $\mathrm{Intercept}_{\mathrm{LD\ score}}$ is the intercept from the LD score regression estimated in the replication data[24]. (As shown above in this Appendix, $\hat{\sigma}_{\theta,j[\mathrm{rep}]} = \frac{1}{N_j}$ if $\theta = \alpha$ and $\hat{\sigma}_{\theta,j[\mathrm{rep}]} = \frac{1-2p_j q_j}{N_j 2p_j q_j}$ if $\theta = d$, where N_j is the sample size for SNP j in the replication GWAS, p_j is the minor allele frequency, and $q_j = 1 - p_j$.)

We let $\hat{C}_{T,k}$ denote the number lead SNPs in the replication's k-th simulation that pass test T. We obtain our estimate of $E[C_T]$ for the replication by averaging $\hat{C}_{T,k}$ across the 1,000 simulations:

$$\hat{E}[C_T] = \frac{1}{1000}\sum_{k=1}^{1000}\hat{C}_{T,k}.$$

In addition, we estimate the standard deviation of C_T by using the formula for the sample standard deviation:

$$\widehat{\mathrm{SD}}(C_T) = \sqrt{\frac{1}{999}\sum_{k=1}^{1000}\left(\hat{C}_{T,k} - \hat{E}[C_T]\right)^2}.$$

We note that the $\hat{C}_{T,k}$'s generated by our simulation procedure capture the uncertainty in C_T stemming from both sampling variation and the posterior distribution of θ_j. Thus, estimating $\widehat{\mathrm{Var}}(C_T) = \left(\widehat{\mathrm{SD}}(C_T)\right)^2$ is the empirical counterpart to calculating the expectation of $(C_T - E[C_T])^2$—i.e., calculating the variance of C_T—with respect to both the distribution of the sampling variation and the posterior distribution of θ_j.

8.5 Use of unadjusted summary statistics when fitting the empirical Bayesian model

As mentioned above (**Supplementary Note** sections 2.2.6, 2.3, 4.6, 8.1, and 8.4), for both our additive and dominance GWAS, we used the unadjusted summary statistics to compute the SNPs' posterior distribution based on the empirical Bayesian model. (Adjusted summary statistics are summary statistics in which the standard errors have been inflated by the square root of the intercept from a LD score regression, whereas standard errors have not been inflated in unadjusted summary statistics. The inflation of the standard errors in adjusted summary statistics is a standard step employed in GWAS analysis to adjust for inflation in SNPs' χ^2 statistics due to population stratification[20].)

For the cross-cohort replications in **Supplementary Note** sections 2.3 and 4.6, the simulations based on the empirical Bayesian model aim to predict the results of our replications under the assumption that the discovery GWAS estimates are not biased, including by population stratification. It is therefore appropriate to use the unadjusted

[24] As mentioned above, in our actual replications, the replication tests use adjusted *P* values in the replication data. Our replication simulations thus reproduce that feature.

summary statistics and to simulate a scenario in which there is no population stratification. More generally and perhaps more plausibly, one can think of the simulations of the cross-cohort replications as assuming that the discovery and replication cohorts come from the same underlying population and that any residual stratification bias (after controlling for PCs) in the estimates in one cohort is also present in the estimates of the other.

In **Supplementary Note** section 2.2.6, we use the unadjusted summary statistics to correct the lead SNPs' estimated effect sizes for the winner's curse. That is because the goal is to correct for the inflation in the magnitude of the estimates due to sampling variation (i.e., for the winner's curse) rather than for bias due to residual population stratification (i.e., the population stratification remaining after the GWAS has already controlled for PCs). While the LD score regression framework could potentially be adapted to correct estimated effect sizes for residual population stratification (as we discuss in the following paragraph), that framework has not to our knowledge previously been used for that precise purpose.

We note that our empirical Bayesian model could in principle be modified to be used with adjusted summary statistics. In the case of our model of additive variance (**Supplementary Note** section 8.1), this could be done by modifying equation (8.2) by adding a parameter σ_s^2 to the variance of $\hat{\alpha}_{j,\text{std}}$ to account for bias due to population stratification:

$$\hat{\alpha}_{j,\text{std}} \sim \begin{cases} N\left(0, \tau^2 + \sigma_j^2 + \sigma_s^2\right) & \text{with probability } \pi \\ N\left(0, \sigma_j^2 + \sigma_s^2\right) & \text{otherwise.} \end{cases}$$

(As mentioned in **Supplementary Note** section 8.1, $\alpha_{j,\text{std}}$ is the coefficient from a regression of the standardized phenotype on the standardized dosage for SNP j, and controls). If we let $\tilde{\sigma}_j^2 \equiv \sigma_j^2 + \sigma_s^2$, we can replace equation (8.2) by

$$\hat{\alpha}_{j,\text{std}} \sim \begin{cases} N\left(0, \tau^2 + \tilde{\sigma}_j^2\right) & \text{with probability } \pi \\ N\left(0, \tilde{\sigma}_j^2\right) & \text{otherwise.} \end{cases} \qquad (8.7)$$

The difficulty is then to estimate $\tilde{\sigma}_j^2$. Under the assumptions that justify adjusting GWAS summary statistics by the LD score intercept, $\tilde{\sigma}_j^2 = \sigma_j^2 \cdot Intercept = \frac{Intercept}{N_j}$, where $Intercept$ is the intercept from the LD score regression and $\sigma_j^2 = \frac{1}{N_j}$ as mentioned in **Supplementary Note** section 8.1. One could thus in principle use our modified empirical Bayesian model (with equation (8.7)) with adjusted summary statistics to estimate the posterior distribution of the true effect sizes $\alpha_{j,\text{std}}$ corrected for both the winner's curse and for population stratification. This approach could be particularly useful if we wanted to simulate a replication of the estimates of a standard (i.e., cross-sectional) GWAS in a within-family GWAS, since the estimates from the latter are not biased by population stratification.

9 Analysis of assortative mating

To investigate whether the estimated correlation between mate pairs' PGIs could be explained by phenotypic assortative mating, we conducted further analyses in samples with both parents genotyped in the UK Biobank (862 mate pairs) and Generation Scotland (1603 mate pairs). Although the UK Biobank is not a representative sample, the correlation between mate pairs' educational attainments in our sample (0.430, S.E. = 0.017) is not very different from those in representative UK samples: very close to the correlation of 0.45 estimated by Hugh-Jones et al.[42] and only somewhat smaller than we estimate (0.513, S.E. = 0.018) in the English Longitudinal Study of Ageing[43] (we used the 3470 mate pairs identified in the harmonized ELSA data from the Gateway to Global Aging (g2aging.org) and our updated UK Biobank coding of *EduYears*).

We identified mate pairs by identifying genotyped parents of genotyped individuals within each sample. Let r_y denote the phenotypic correlation between mate pairs, and let r_p and r_m denote the correlations between the phenotype and PGI for the father and mother, respectively. The correlation between the mate pairs' PGIs should be equal to $r_y r_p r_m$ if the correlation is explained by assortative mating on the phenotype alone, and the relationship between the PGI and the phenotype is linear. Furthermore, the PGI of the mother conditional on the mother's phenotype, should be independent of the PGI of the father, conditional on the father's phenotype. We note that we use the correlation in phenotypes measured in mate pairs after they paired up, which could be inflated if mate pairs influence each other, leading to greater phenotypic similarity than at the time of pairing. This would have the effect of predicting a higher mate-pair PGI correlation than if we had used phenotypes from the time of pairing.

To test the model of phenotypic assortment, we estimated the expected correlation between mate pairs' PGIs by estimating r_y, r_p, and r_m. We estimated the standard error of the product of r_y, r_p, and r_m using 1000 bootstrap samples where we sampled over the mate pairs. We also estimated the correlation between the residual of the father's PGI after regression onto the father's phenotype and the residual of the mother's PGI after regression onto the mother's phenotype. This correlation between residuals should be zero if the correlation between mate pairs' PGIs is explained entirely by assortative mating on the phenotype, and the relationship between phenotype and PGI is linear. To test robustness to the linearity assumption, we also computed the correlation between the residuals from regression of (father or mother)'s PGI onto a cubic function of the (father or mother)'s trait. This did not make a noticeable difference to the results (**Supplementary Table 14**), so we used only a linear model for the relationship between trait and PGI for the following analyses.

We investigated whether, beyond assortative mating on the phenotype alone, assortment based on genetic ancestry captured by genetic principal components explained the observed correlation between mate pairs' PGIs. To do this, we took the residuals of the father's PGI after regression on the father's phenotype and the father's values of the top 40 genetic principal components; and we computed the correlation of this with the residuals of the mother's PGI after regression on the mother's phenotype and the mother's values of the top 40 genetic principal components.

In UKB, north and east birth coordinates in the UK (Data Fields 129-130) were recorded, in addition to the center where individuals were assessed (Data Field 54). To further assess the impact of geographic factors on the correlation between mate pairs' EA PGIs, we added north and east birth coordinates and the product of north and east

birth coordinate, along with assessment center coded as a categorical variable, as regressors to the regression of the EA PGI onto EA and principal components in the UKB.

For EA, we hypothesized that assortment may occur through phenotypes correlated with EA, such as cognitive performance and vocabulary. To assess this, we added the cognitive performance and vocabulary phenotypes as regressors to the regressions of the EA PGI onto EA and principal components, and we computed the correlation between the mate pairs' residuals (**Supplementary Table 14**). We did this in Generation Scotland alone, due to the lack of appropriate cognitive measures in UKB.

10 Proofs for sibling-based analysis

Consider joint regression of Y_{ij} onto $PGI_{ij} - \overline{PGI}_i$ and $\overline{PGI}_i$, where $\overline{PGI}_i = \frac{1}{n_i}\sum_{j=1}^{n_i} PGI_{ij}$, and n_i is the number of genotyped siblings (including the focal individual j) in family i. We have that $Cov(PGI_{ij}, PGI_{ik}) = (1+r)/2$ for $j \neq k$; and

$$Cov(PGI_{ij}, PGI_{p(i)}) = Cov(PGI_{ij}, PGI_{m(i)}) = (1+r)/2. \qquad (9.1)$$

First, we show that $Cov(\overline{PGI}_i, PGI_{ij} - \overline{PGI}_i) = 0$. By the definition of covariance, we have that

$$Cov(\overline{PGI}_i, PGI_{ij} - \overline{PGI}_i) = Cov(\overline{PGI}_i, PGI_{ij}) - Var(\overline{PGI}_i). \qquad (9.2)$$

We now compute the terms in equation (9.2), that is

$$Cov(\overline{PGI}_i, PGI_{ij}) = \frac{1}{n_i}\left(1 + (n_i - 1)\frac{1+r}{2}\right) \qquad (9.3)$$

$$= \frac{1}{n_i} + \left(1 - \frac{1}{n_i}\right)\frac{1+r}{2}; \qquad (9.4)$$

and

$$Var(\overline{PGI}_i) = \frac{1}{n_i^2}\left(n_i + n_i(n_i - 1)\frac{1+r}{2}\right) \qquad (9.5)$$

$$= \frac{1}{n_i} + \left(1 - \frac{1}{n_i}\right)\frac{1+r}{2}. \qquad (9.6)$$

Therefore, $Cov(\overline{PGI}_i, PGI_{ij} - \overline{PGI}_i) = 0$. This means, for the purpose of deriving the expected regression coefficients, the joint regression can be treated as two independent univariate regressions.

To compute the expected estimates, we first compute $Cov(Y_{ij}, PGI_{ij})$ and $Cov(Y_{ij}, \overline{PGI}_i)$:

$$Cov(Y_{ij}, PGI_{ij}) = \delta + \frac{1+r}{2}(\alpha_p + \alpha_m); \qquad (9.7)$$

and

$$Cov(Y_{ij}, \overline{PGI}_i) = \delta Var(\overline{PGI}_i) + Cov(\overline{PGI}_i, PGI_{p(i)})(\alpha_p + \alpha_m), \qquad (9.8)$$

using the fact that $Cov(PGI_{ij}, \overline{PGI}_i) = Var(\overline{PGI}_i)$ and the symmetry of parent-offspring PGI covariances. We now compute

$$Cov(\overline{PGI}_i, PGI_{p(i)}) = \frac{1}{n_i} n_i \frac{1+r}{2} = \frac{1+r}{2}. \qquad (9.9)$$

Therefore,

$$Cov\left(Y_{ij}, \overline{PGI}_i\right) = \delta Var(\overline{PGI}_i) + \frac{1+r}{2}\left(\alpha_p + \alpha_m\right). \quad (9.10)$$

We therefore have that

$$Cov\left(Y_{ij}, PGI_{ij} - \overline{PGI}_i\right) = \left(1 - Var(\overline{PGI}_i)\right)\delta. \quad (9.11)$$

Furthermore, we have that

$$\begin{aligned} Var\left(PGI_{ij} - \overline{PGI}_i\right) &= 1 + Var(\overline{PGI}_i) - 2Cov\left(PGI_{ij}, \overline{PGI}_i\right) \\ &= 1 - Var(\overline{PGI}_i). \end{aligned} \quad (9.12)$$

Therefore, the regression of Y_{ij} onto $PGI_{ij} - \overline{PGI}_i$ gives an unbiased estimate of δ.

The expected regression coefficient from the regression of Y_{ij} onto $\overline{PGI}_i$ depends upon the distribution of the sizes of sibships in the sample. Let Y be a vector comprised of observations from multiple sibships each of size n_i, with n_i potentially varying between sibships, and let $\overline{PGI}_i$ be a corresponding vector of mean PGI in each family. To compute the expected regression coefficient in this case, we need to compute the total variance of $\overline{PGI}_i$ across sibships of different size:

$$Var(\overline{PGI}_i) = \mathbb{E}_i[Var(\overline{PGI}_i)] = \frac{1+r}{2}\left(1 + \frac{1-r}{1+r}\mathbb{E}\left[\frac{1}{n_i}\right]\right). \quad (9.13)$$

Similarly,

$$\begin{aligned} Cov(\overline{PGI}_i, Y) &= \mathbb{E}_i\left[Cov\left(\overline{PGI}_i, Y_{ij}\right)\right] \\ &= \delta \mathbb{E}_i[Var(\overline{PGI}_i)] + \frac{1+r}{2}\left(\alpha_p + \alpha_m\right). \end{aligned} \quad (9.14)$$

Therefore, we have that the expected regression coefficient from the regression of Y onto $\overline{PGI}_i$ is

$$\frac{Cov(Y, \overline{PGI}_i)}{Var(\overline{PGI}_i)} = \delta + \left(1 + \frac{1-r}{1+r}\mathbb{E}\left[\frac{1}{n_i}\right]\right)^{-1}\left(\alpha_p + \alpha_m\right). \quad (9.15)$$

11 Additional acknowledgements

23andMe, Inc. — 23andMe research participants provided informed consent to take part in this research under a protocol approved by the AAHRPP-accredited institutional review board, Ethical and Independent Review Services. We would like to thank the research participants and employees of 23andMe for making this work possible.

Add Health (National Longitudinal Study of Adolescent to Adult Health) — dbGaP accession: phs001367.v1.p1. This research uses data from Add Health, a program project directed by Kathleen Mullan Harris and designed by J. Richard Udry, Peter S. Bearman, and Kathleen Mullan Harris at the University of North Carolina at Chapel Hill, and funded by grant P01 HD31921 from Eunice Kennedy Shriver National Institute of Child Health and Human Development (NICHD), with cooperative funding from 23 other federal agencies and foundations. Add Health GWAS data were funded by NICHD Grants R01 HD073342 (Harris) and R01 HD060726 (Harris, Boardman, and McQueen). Investigators thank the staff and participants of the Add Health Study for their important contributions.

GSII (Generation Scotland) — We would like to acknowledge the contributions of the families who took part in the Generation Scotland: Scottish Family Health Study, the general practitioners and Scottish School of Primary Care for their help in recruiting them, and the whole Generation Scotland team, which includes academic researchers, IT staff, laboratory technicians, statisticians and research managers. Generation Scotland received core support from the Chief Scientist Office of the Scottish Government Health Directorates [CZD/16/6] and the Scottish Funding Council [HR03006] and is currently supported by the Wellcome Trust [216767/Z/19/Z]. Genotyping of the GS:SFHS samples was carried out by the Genetics Core Laboratory at the Edinburgh Clinical Research Facility, University of Edinburgh, Scotland and was funded by the Medical Research Council UK and the Wellcome Trust (Wellcome Trust Strategic Award "STratifying Resilience and Depression Longitudinally" (STRADL) Reference 104036/Z/14/Z). We are grateful to all the families who took part, the general practitioners and the Scottish School of Primary Care for their help in recruiting them, and the whole Generation Scotland team, which includes interviewers, computer and laboratory technicians, clerical workers, research scientists, volunteers, managers, receptionists, healthcare assistants and nurses. Information on applications for access to Generation Scotland data can be found at http://www.generationscotland.org/.

HRS (Health and Retirement Study) — HRS is supported by the National Institute on Aging (NIA U01AG009740). HRS genotyping received additional support from the National Institute on Aging (RC2 AG036495, RC4 AG039029). Genotype data on HRS participants was obtained using the llumina HumanOmni2.5 BeadChips (HumanOmni2.5-4v1, HumanOmni2.5-8v1, HumanOmni2.5-8v1.1). Genotyping was conducted by the NIH Center for Inherited Disease Research (CIDR) at Johns Hopkins University. Genotyping quality control and final preparation of the data were performed by the Genetics Coordinating Center at the University of Washington and the University of Michigan. The validation dataset includes respondents who provided DNA samples and signed consent forms in 2006, 2008, and 2010 (dbGaP accession phs000428.v2.p2).

STR (Swedish Twin Registry) — The Swedish Twin Registry is managed by Karolinska Institutet and receives funding through the Swedish Research Council under the grant no 2017-00641. Genotyping was performed by the SNP&SEQ Technology Platform in Uppsala (www.genotyping.se). The facility is part of the National Genomics Infrastructure supported by the Swedish Research Council for Infrastructures and Science for Life Laboratory, Sweden. The SNP&SEQ Technology Platform is also supported by the Knut and Alice Wallenberg Foundation.

WLS (Wisconsin Longitudinal Study) — dbGaP accession: phs001157.v1.p1. The Wisconsin Longitudinal Study genetic data is sponsored by the National Institute on Aging (grant numbers R01AG009775, R01AG033285, and R01AG041868) and was conducted by the University of Wisconsin.

UK Biobank — This research has also been conducted using the UK Biobank Resource under Application Numbers 11425 and 12505. Informed consent was obtained from UK Biobank subjects.

12 References

1. Okbay, A. *et al.* Genome-wide association study identifies 74 loci associated with educational attainment. *Nature* **533**, 539–542 (2016).

2. Lee, J. J. *et al.* Gene discovery and polygenic prediction from a genome-wide association study of educational attainment in 1.1 million individuals. *Nat. Genet.* **50**, 1112–1121 (2018).

3. UNESCO Institute for Statistics. United Kingdom ISCED Mappings. *ISCED Mappings* http://uis.unesco.org/sites/default/files/documents/isced_2011_mapping_en_united_kingdom_0.xlsx (2011).

4. Department for Education. Qualification Levels. *What Qualification Levels Mean* https://www.gov.uk/what-different-qualification-levels-mean/list-of-qualification-levels (2020).

5. West, J. & Steedman, H. Finding our way: vocational education in England. *LSE Res. Online Doc. Econ.* (2003).

6. HESA. Higher Education Student Statistics: UK, 2018/19. *Statistical Bulletin SB255* https://www.hesa.ac.uk/news/16-01-2020/sb255-higher-education-student-statistics (2020).

7. Boniface, R., Whalley, G. & Goodwin, D. *Mapping the higher technical landscape*. http://www.gatsby.org.uk/uploads/education/reports/pdf/mapping-the-higher-technical-landscape-final-version.pdf (2018).

8. National Committee of Inquiry into Higher Education. *Higher Education in the Learning Society (The Dearing Report)*. (1997).

9. Auton, A. *et al.* A global reference for human genetic variation. *Nature* **526**, 68–74 (2015).

10. Walter, K. *et al.* The UK10K project identifies rare variants in health and disease. *Nature* **526**, 82–90 (2015).

11. Das, S. *et al.* Next-generation genotype imputation service and methods. *Nat. Genet.* **48**, 1284–1287 (2016).

12. Browning, S. R. & Browning, B. L. Rapid and accurate haplotype phasing and missing-data inference for whole-genome association studies by use of localized haplotype clustering. *Am. J. Hum. Genet.* **81**, 1084–1097 (2007).

13. Loh, P. R., Palamara, P. F. & Price, A. L. Fast and accurate long-range phasing in a UK Biobank cohort. *Nat. Genet.* **48**, 811–816 (2016).

14. Durand, E. Y., Do, C. B., Mountain, J. L. & Macpherson, J. M. Ancestry Composition: A Novel, Efficient Pipeline for Ancestry Deconvolution. *bioRxiv* 010512 (2014) doi:10.1101/010512.

15. Henn, B. M. *et al.* Cryptic Distant Relatives Are Common in Both Isolated and Cosmopolitan Genetic Samples. *PLoS One* **7**, e34267 (2012).

16. McCarthy, S. *et al.* A reference panel of 64,976 haplotypes for genotype

imputation. *Nat. Genet.* **48**, 1279–1283 (2016).

17. Abecasis, G. R. *et al.* A map of human genome variation from population-scale sequencing. *Nature* **467**, 1061–1073 (2010).

18. Willer, C. J., Li, Y. & Abecasis, G. R. METAL: fast and efficient meta-analysis of genomewide association scans. *Bioinformatics* **26**, 2190–2191 (2010).

19. Yang, J. *et al.* Genomic inflation factors under polygenic inheritance. *Eur. J. Hum. Genet.* **19**, 807–812 (2011).

20. Bulik-Sullivan, B. K. *et al.* LD Score regression distinguishes confounding from polygenicity in genome-wide association studies. *Nat. Genet.* **47**, 291–295 (2015).

21. The 1000 Genomes Project Consortium *et al.* An integrated map of genetic variation from 1,092 human genomes. *Nature* **491**, 56–65 (2012).

22. Chang, C. C. *et al.* Second-generation PLINK: rising to the challenge of larger and richer datasets. *Gigascience* **4**, 1–16 (2015).

23. Yang, J., Lee, S. H., Goddard, M. E. & Visscher, P. M. GCTA: A tool for genome-wide complex trait analysis. *Am. J. Hum. Genet.* **88**, 76–82 (2011).

24. Rietveld, C. A. *et al.* GWAS of 126,559 individuals identifies genetic variants associated with educational attainment. *Science* **340**, 1467–1471 (2013).

25. Bulik-Sullivan, B. K. *et al.* An atlas of genetic correlations across human diseases and traits. *Nat. Genet.* **47**, 1236–1241 (2015).

26. Yang, J. *et al.* Conditional and joint multiple-SNP analysis of GWAS summary statistics identifies additional variants influencing complex traits. *Nat. Genet.* **44**, 369–375 (2012).

27. Purcell, S. & Chang, C. PLINK 2.0.

28. Karlsson Linnér, R. *et al.* Genome-wide association analyses of risk tolerance and risky behaviors in over 1 million individuals identify hundreds of loci and shared genetic influences. *Nat. Genet.* **51**, 245–257 (2019).

29. Loh, P.-R. *et al.* Efficient Bayesian mixed-model analysis increases association power in large cohorts. *Nat. Genet.* **47**, 284–290 (2015).

30. Sidorenko, J. *et al.* The effect of X-linked dosage compensation on complex trait variation. *Nat. Commun.* **10**, 1–11 (2019).

31. Hill, W. G., Goddard, M. E. & Visscher, P. M. Data and theory point to mainly additive genetic variance for complex traits. *PLoS Genet.* **4**, e1000008 (2008).

32. Clark, D. W. *et al.* Associations of autozygosity with a broad range of human phenotypes. *Nat. Commun.* **10**, 1–17 (2019).

33. Robertson, A. & Hill, W. G. Population and quantitative genetics of many linked loci in finite populations. *Proc. R. Soc. London - Biol. Sci.* **219**, 253–264 (1983).

34. Hivert, V. *et al.* Estimation of non-additive genetic variance in human complex traits from a large sample of unrelated individuals. *Am. J. Hum. Genet.* **108**, 786–798 (2021).

35. Pazokitoroudi, A., Chiu, A. M., Burch, K. S., Pasaniuc, B. & Sankararaman, S. *Quantifying the contribution of dominance effects to complex trait variation in biobank-scale data*. *bioRxiv* (2020) doi:10.1101/2020.11.10.376897.

36. Risch, N. J. Searching for genetic determinants in the new millennium. *Nature* **405**, 847–856 (2000).

37. Visscher, P. M. & Goddard, M. E. From R.A. Fisher's 1918 Paper to GWAS a century later. *Genetics* **211**, 1125–1130 (2019).

38. Falconer, D. S. & Mackay, T. F. C. *Introduction to Quantitative Genetics*. (Longman, 1996).

39. Lynch, M. & Walsh, B. *Genetics and Analysis of Quantitative Traits*. *Genetics and Analysis of Quantitative Traits* (Sinauer Associates, Inc., 1998).

40. Fisher, R. A. The Correlation between Relatives on the Supposition of Mendelian Inheritance. *Trans. R. Soc. Edinburgh* **52**, 399–433 (1918).

41. Yengo, L. *et al.* Genomic partitioning of inbreeding depression in humans. *Am. J. Hum. Genet.* **108**, 1488–1501 (2021).

42. Bashi, J. Effects of inbreeding on cognitive performance. *Nature* **266**, 440–2 (1977).

43. Joshi, P. K. *et al.* Directional dominance on stature and cognition in diverse human populations. *Nature* **523**, 459–462 (2015).

44. Charlesworth, D. & Willis, J. H. The genetics of inbreeding depression. *Nat. Rev. Genet.* **10**, 783–96 (2009).

45. Ceballos, F. C., Joshi, P. K., Clark, D. W., Ramsay, M. & Wilson, J. F. Runs of homozygosity: windows into population history and trait architecture. *Nat. Rev. Genet.* **19**, 220–234 (2018).

46. Yengo, L. *et al.* Detection and quantification of inbreeding depression for complex traits from SNP data. *Proc. Natl. Acad. Sci. U. S. A.* **114**, 8602–8607 (2017).

47. Vilhjálmsson, B. J. *et al.* Modeling Linkage Disequilibrium Increases Accuracy of Polygenic Risk Scores. *Am. J. Hum. Genet.* **97**, 576–592 (2015).

48. Wray, N. R., Goddard, M. E. & Visscher, P. M. Prediction of individual genetic risk to disease from genome-wide association studies. *Genome Res.* **17**, 1520–1528 (2007).

49. UNESCO Institute for Statistics. *International Standard Classification of Education*. (2006).

50. Dunn, L. M. & Dunn, D. M. Peabody Picture Vocabulary Test,. *Summ. Shute. Inst.* **30**, 1–8 (2007).

51. Wechsler, D. *Manual for the Wechsler adult intelligence scale - Revised*. (Pyschological Corporation, 1981).

52. Henmon, V. A. C. Henmon-Nelson Tests of Mental Ability, High School Examination-Grades 7 to 12-Forms A, B, and C. Teacher's Manual. (1946).

53. de Vlaming, R. *et al.* Meta-GWAS Accuracy and Power (MetaGAP) Calculator Shows that Hiding Heritability Is Partially Due to Imperfect Genetic Correlations across Studies. *PLOS Genet.* **13**, e1006495 (2017).

54. Becker, J. *et al.* Resource profile and user guide of the Polygenic Index Repository. *Nat. Hum. Behav.* (2021) doi:10.1038/s41562-021-01119-3.

55. Duncan, L. *et al.* Analysis of polygenic risk score usage and performance in diverse human populations. *Nat. Commun. 2019 101* **10**, 1–9 (2019).

56. Martin, A. R. *et al.* Clinical use of current polygenic risk scores may exacerbate health disparities. *Nat. Genet.* **51**, 584–591 (2019).

57. Wang, Y. *et al.* Theoretical and empirical quantification of the accuracy of polygenic scores in ancestry divergent populations. *Nat. Commun.* **11**, 1–9 (2020).

58. Lloyd-Jones, L. R. *et al.* Improved polygenic prediction by Bayesian multiple regression on summary statistics. *Nat. Commun.* **10**, 5086 (2019).

59. Zeng, J. *et al.* Signatures of negative selection in the genetic architecture of human complex traits. *Nat. Genet. 2018 505* **50**, 746–753 (2018).

60. Morris, A. P. *et al.* Large-scale association analysis provides insights into the genetic architecture and pathophysiology of type 2 diabetes. *Nat Genet* **44**, 981–990 (2012).

61. Willer, C. J. *et al.* Discovery and refinement of loci associated with lipid levels. *Nat Genet* **45**, 1274–1283 (2013).

62. Wain, L. V *et al.* Novel Blood Pressure Locus and Gene Discovery Using Genome-Wide Association Study and Expression Data Sets From Blood and the Kidney. *Hypertension* **70**, e4–e19 (2017).

63. Okada, Y. *et al.* Genetics of rheumatoid arthritis contributes to biology and drug discovery. *Nature* **506**, 376–381 (2014).

64. Zheng, H. F. *et al.* Whole-genome sequencing identifies EN1 as a determinant of bone density and fracture. *Nature* **526**, 112–117 (2015).

65. Wray, N. R. *et al.* Genome-wide association analyses identify 44 risk variants and refine the genetic architecture of major depression. *Nat. Genet.* **50**, 668–681 (2018).

66. Demenais, F. *et al.* Multiancestry association study identifies new asthma risk loci that colocalize with immune-cell enhancer marks. *Nat Genet* **50**, 42–53 (2018).

67. Nikpay, M. *et al.* A comprehensive 1000 Genomes-based genome-wide association meta-analysis of coronary artery disease. *Nat. Genet.* **47**, 1121–1130 (2015).

68. Gormley, P. *et al.* Meta-analysis of 375,000 individuals identifies 38 susceptibility loci for migraine. *Nat. Genet.* **48**, 856–866 (2016).

69. Kong, A. *et al.* The nature of nurture: Effects of parental genotypes. *Science* **359**, 424–428 (2018).

70. Walsh, B. & Lynch, M. *Evolution and selection of quantitative traits*. (Oxford University Press, 2018).

71. Young, A. I., Benonisdottir, S., Przeworski, M. & Kong, A. Deconstructing the sources of genotype-phenotype associations in humans. *Science* **365**, 1396–1400 (2019).

72. Słoczyński, T. Interpreting OLS Estimands When Treatment Effects Are Heterogeneous: Smaller Groups Get Larger Weights. *Rev. Econ. Stat.* 1–27 (2020) doi:10.1162/rest_a_00953.

73. Young, A. I. *et al.* Relatedness disequilibrium regression estimates heritability without environmental bias. *Nat. Genet.* **50**, 1304–1310 (2018).

74. Bycroft, C. *et al.* The UK Biobank resource with deep phenotyping and genomic data. *Nature* **562**, 203–209 (2018).

75. Smith, B. H. *et al.* Cohort Profile: Generation Scotland: Scottish Family Health Study (GS:SFHS). The study, its participants and their potential for genetic research on health and illness. *Int. J. Epidemiol.* **42**, 689–700 (2013).

76. Zagai, U., Lichtenstein, P., Pedersen, N. L. & Magnusson, P. K. E. The Swedish Twin Registry: Content and Management as a Research Infrastructure. *Twin Res. Hum. Genet.* **22**, 672–680 (2019).

77. Manichaikul, A. *et al.* Robust relationship inference in genome-wide association studies. *Bioinformatics* **26**, 2867–2873 (2010).

78. Trampush, J. W. *et al.* GWAS meta-analysis reveals novel loci and genetic correlates for general cognitive function: a report from the COGENT consortium. *Mol. Psychiatry* **22**, 336–345 (2017).

79. Wood, A. R. *et al.* Defining the role of common variation in the genomic and biological architecture of adult human height. *Nat. Genet.* **46**, 1173–1186 (2014).

80. Locke, A. E. A. *et al.* Genetic studies of body mass index yield new insights for obesity biology. *Nature* **518**, 197–206 (2015).

81. Young, A. I. *et al. Mendelian imputation of parental genotypes for genome-wide estimation of direct and indirect genetic effects*. *BioRxiv* (2020) doi:10.1101/2020.07.02.185199.

82. Kong, A., Benonisdottir, S. & Young, A. I. *Family Analysis with Mendelian Imputations*. *BioRxiv* (2020) doi:10.1101/2020.07.02.185181.

83. Zaitlen, N. *et al.* Using Extended Genealogy to Estimate Components of Heritability for 23 Quantitative and Dichotomous Traits. *PLoS Genet.* **9**, e1003520 (2013).

84. Northern Ireland Statistics and Research Agency. Births 1887-2019. *Birth Statistics* https://www.nisra.gov.uk/sites/nisra.gov.uk/files/publications/live_births_1887_2019.xls (2020).

85. National Records of Scotland. Births, by sex, Scotland, 1855 to 2019. *Births Time Series Data* https://www.nrscotland.gov.uk/files//statistics/time-

series/birth-19/births-time-series-19-bt.1.xlsx (2020).

86. Office for National Statistics. 2019 Summary Tables. *Births in England and Wales: summary tables* https://www.ons.gov.uk/file?uri=%2Fpeoplepopulationandcommunity%2Fbirthsdeathsandmarriages%2Flivebirths%2Fdatasets%2Fbirthsummarytables%2F2019/birthsummarytables2019.xlsx (2020).

87. Office for National Statistics. Child mortality (death cohort) tables in England and Wales. *Child and infant mortality in England and Wales* https://www.ons.gov.uk/file?uri=%2Fpeoplepopulationandcommunity%2Fbirthsdeathsandmarriages%2Fdeaths%2Fdatasets%2Fchildmortalitystatisticschildhoodinfantandperinatalchildhoodinfantandperinatalmortalityinenglandandwales%2F2018/cms2018workbookf.xls (2018).

88. Office for National Statistics. Vital Statistics in the UK: births, deaths and marriages. *Population estimates* https://www.ons.gov.uk/file?uri=%2Fpeoplepopulationandcommunity%2Fpopulationandmigration%2Fpopulationestimates%2Fdatasets%2Fvitalstatisticspopulationandhealthreferencetables%2Fcurrent/annualreferencetable2019v2.xlsx (2019).

Supplementary Figures

Supplementary Figure 1. Stratified LD score regression (SLDSC). The figures display the results of running SLDSC on baseline annotations from Gazel et al. (2017) and gene set annotations from Cahoy et al. (2008). Estimates of baseline annotations are from running SLDSC on all the baseline annotations jointly. Estimates of Cahoy et al. (2008) annotations are from running SLDSC on the baseline annotations jointly with one Cahoy et al. annotation added at a time. All figures compare EA4 (this paper, N = 3,037,499) results against the same analysis using EA3 (Lee et al., 2018, N = 1,131,881) results. Error bars are 95% confidence intervals calculated from block jackknife standard errors. Panels: **(a)**: Enrichment estimates; **(b)**: Proportion of heritability estimates; **(c)**: Enrichment standard errors of EA4 against EA3; **(d)**: Proportion of heritability standard errors of EA4 against EA3. Error bars in panels **(a)** and **(b)** are 95% confidence intervals. See **Supplementary Table 30** for additional details.

a. Enrichment estimates

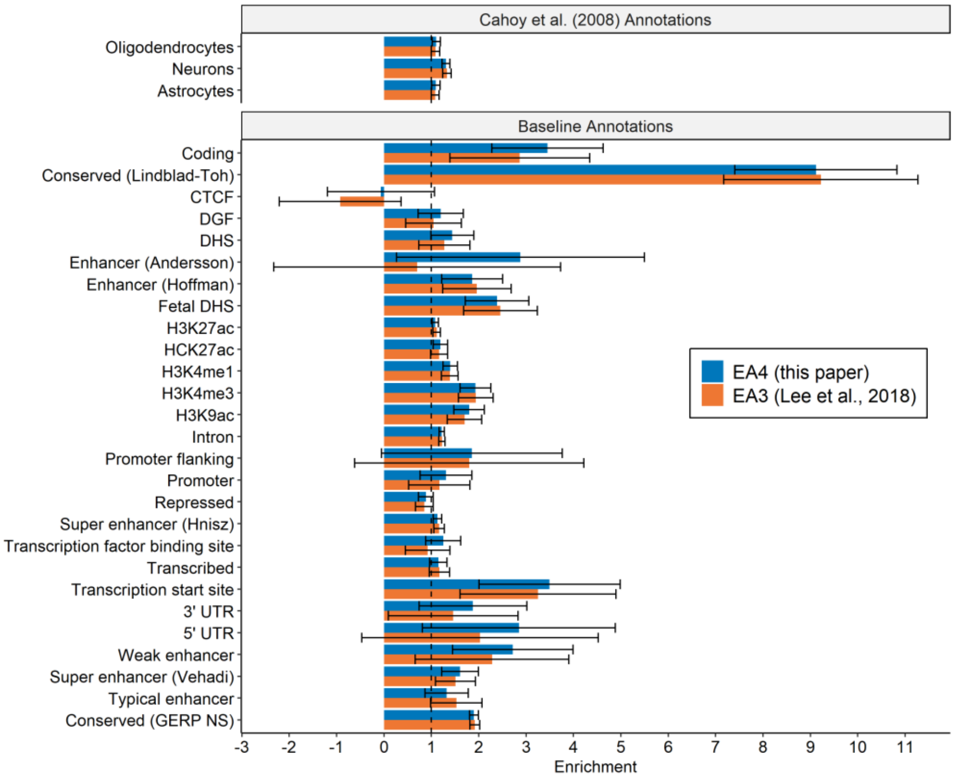

b. Proportion of h^2 estimates

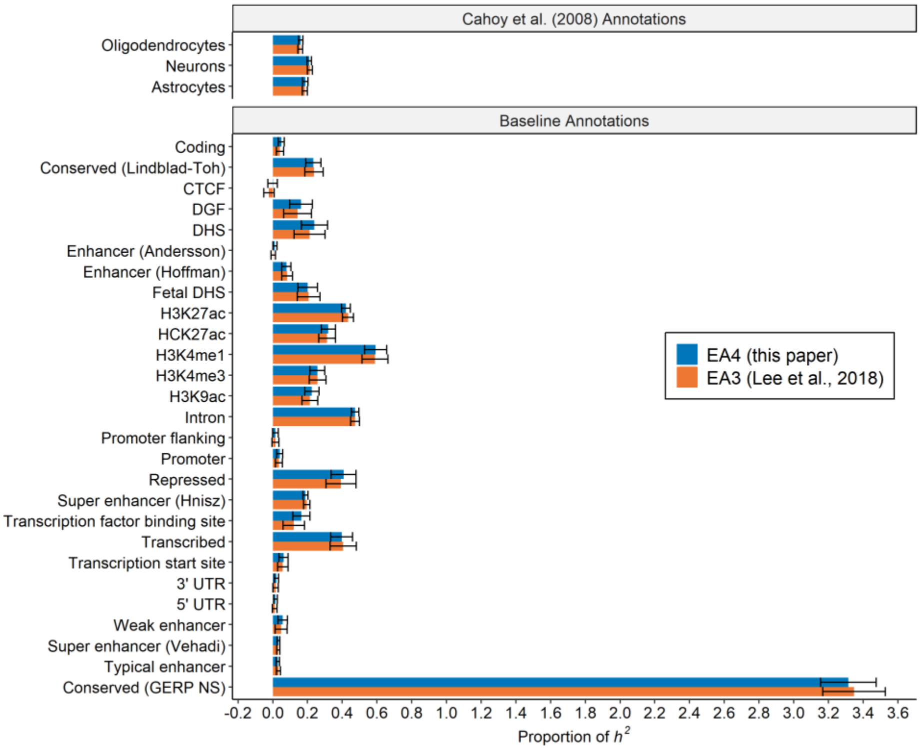

c. Enrichment standard errors

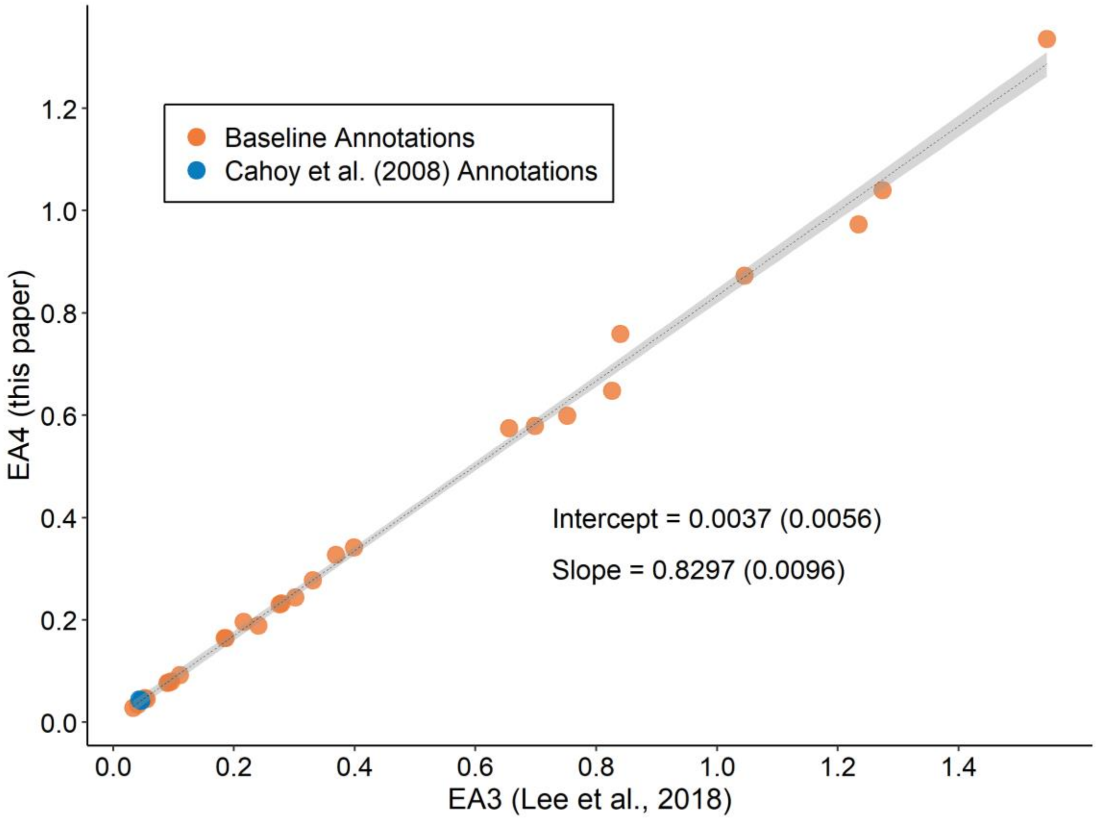

d. Proportion of *h*^{2} standard errors

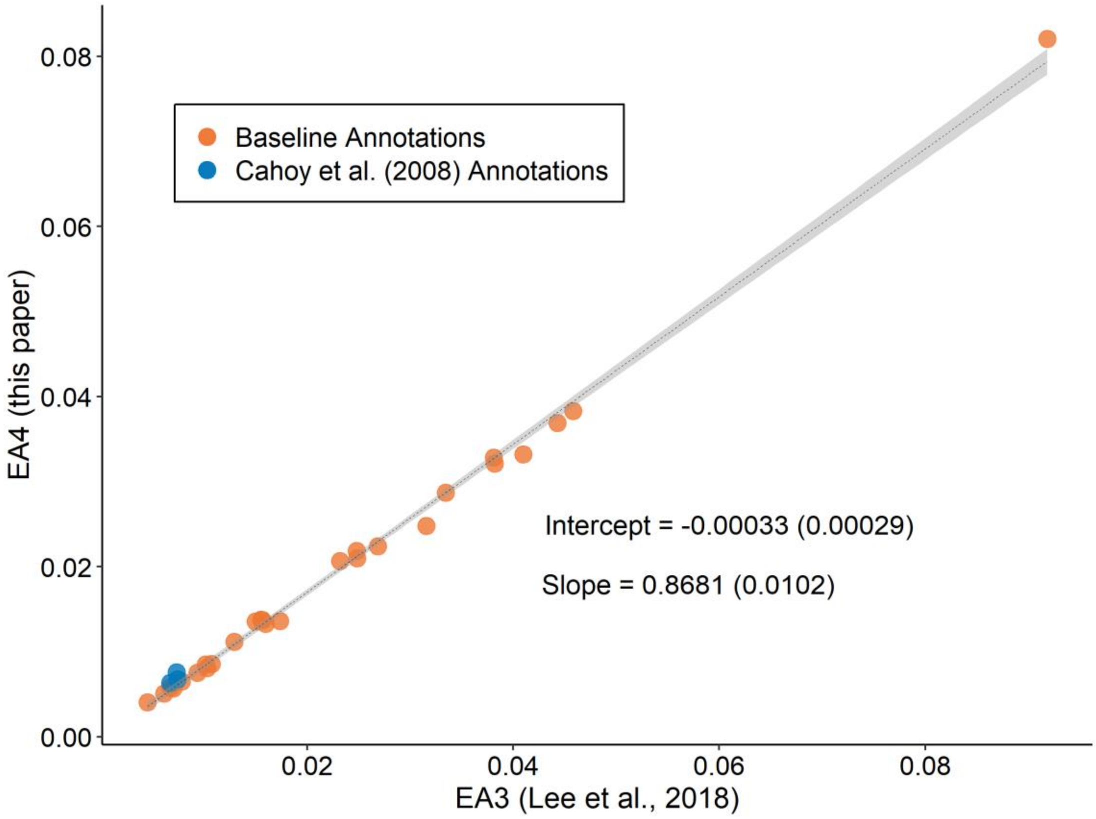

Supplementary Figure 2. Meta-analysis estimates of direct and population effects of PGIs. Plots the standardized effects on 23 phenotypes of PGIs for **(a)** height, **(b)** BMI, and **(c)** cognitive performance. The effects of the PGI on its own phenotype are highlighted in gray. Bars are shaded lighter when the population and direct effects are statistically indistinguishable (two-sided *Z*-test $P > 0.05/23$, where 23 is the number of phenotypes under study). All estimates are from meta-analyses of UKB, GS, and STR samples of siblings and trios. Phenotypes and the PGIs are scaled to have variance one in the combined sample, so effect sizes are partial correlation coefficients. Error bars are 95% confidence intervals. See **Supplementary Table 9** for details on phenotypes and **Supplementary Tables 10-13** for numerical values underlying this figure.

a. Height PGI

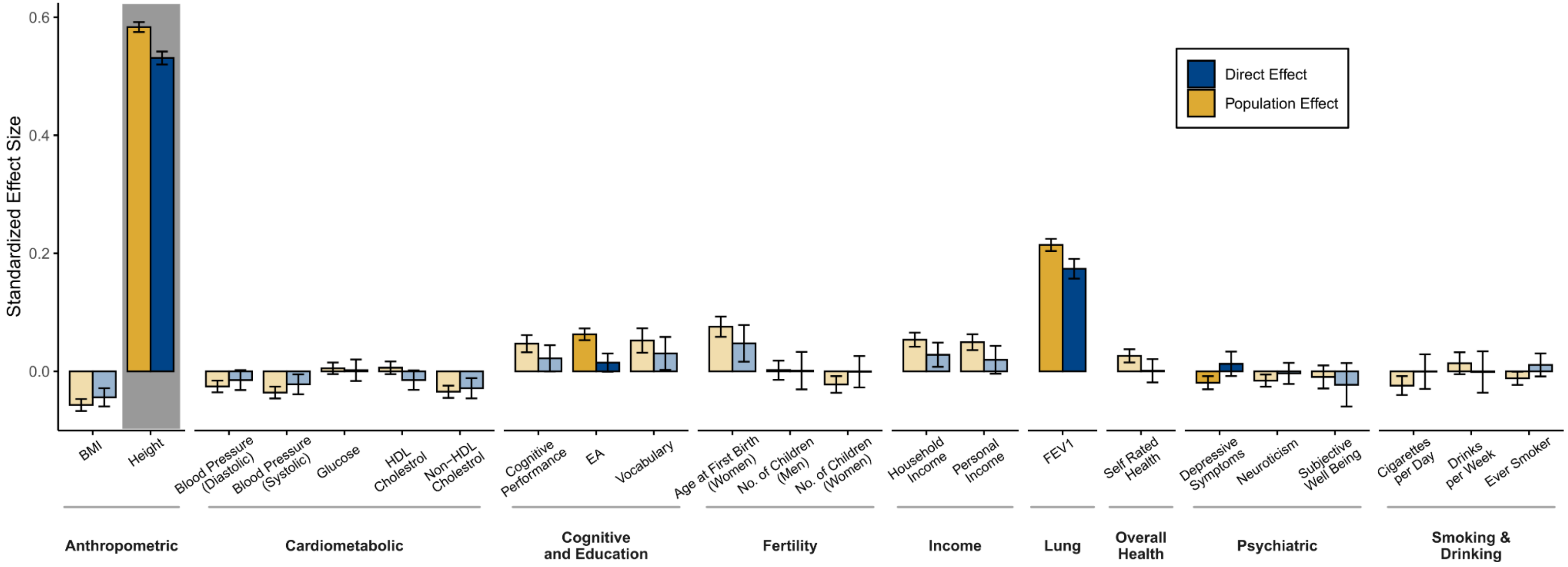

b. BMI PGI

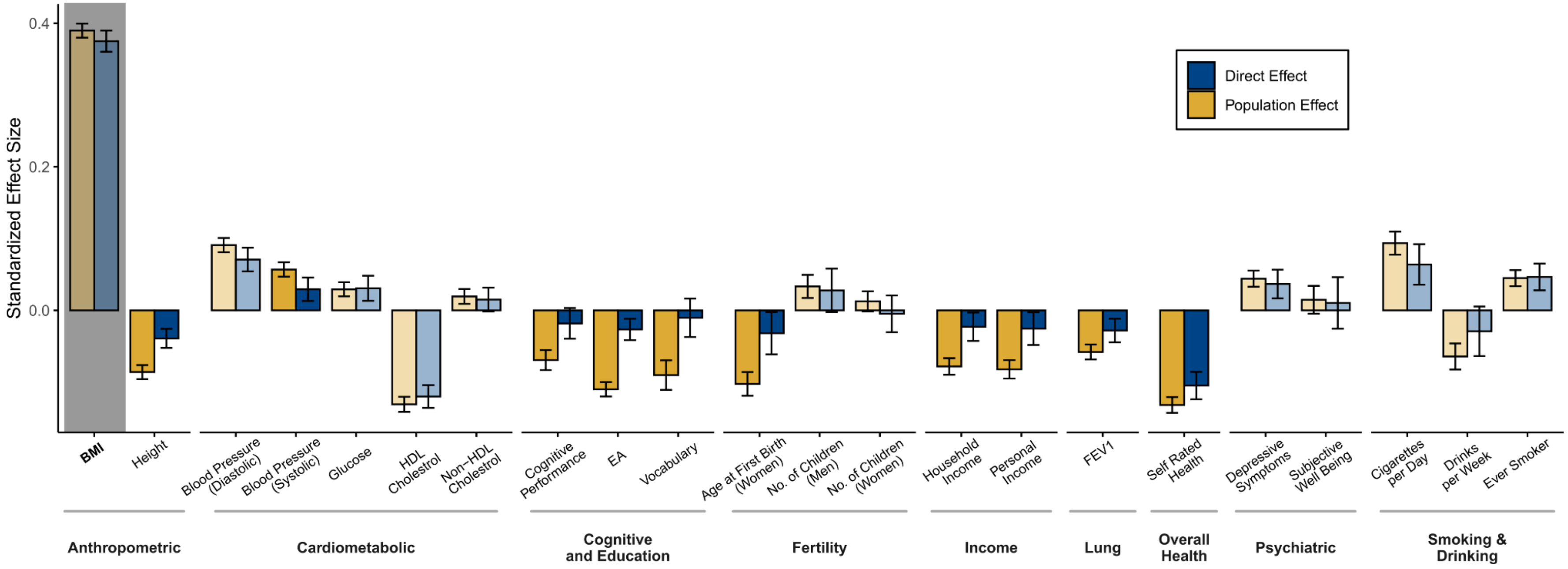

Standardized Effect Size
0.4
0.2
0.0
Direct Effect
Population Effect
BMI
Height
Blood Pressure (Diastolic)
Blood Pressure (Systolic)
Glucose
HDL Cholestrol
Non−HDL Cholestrol
Cognitive Performance
EA
Vocabulary
Age at First Birth (Women)
No. of Children (Men)
No. of Children (Women)
Household Income
Personal Income
FEV1
Self Rated Health
Depressive Symptoms
Subjective Well Being
Cigarettes per Day
Drinks per Week
Ever Smoker
Anthropometric
Cardiometabolic
Cognitive and Education
Fertility
Income
Lung
Overall Health
Psychiatric
Smoking & Drinking

c. Cognitive Performance PGI

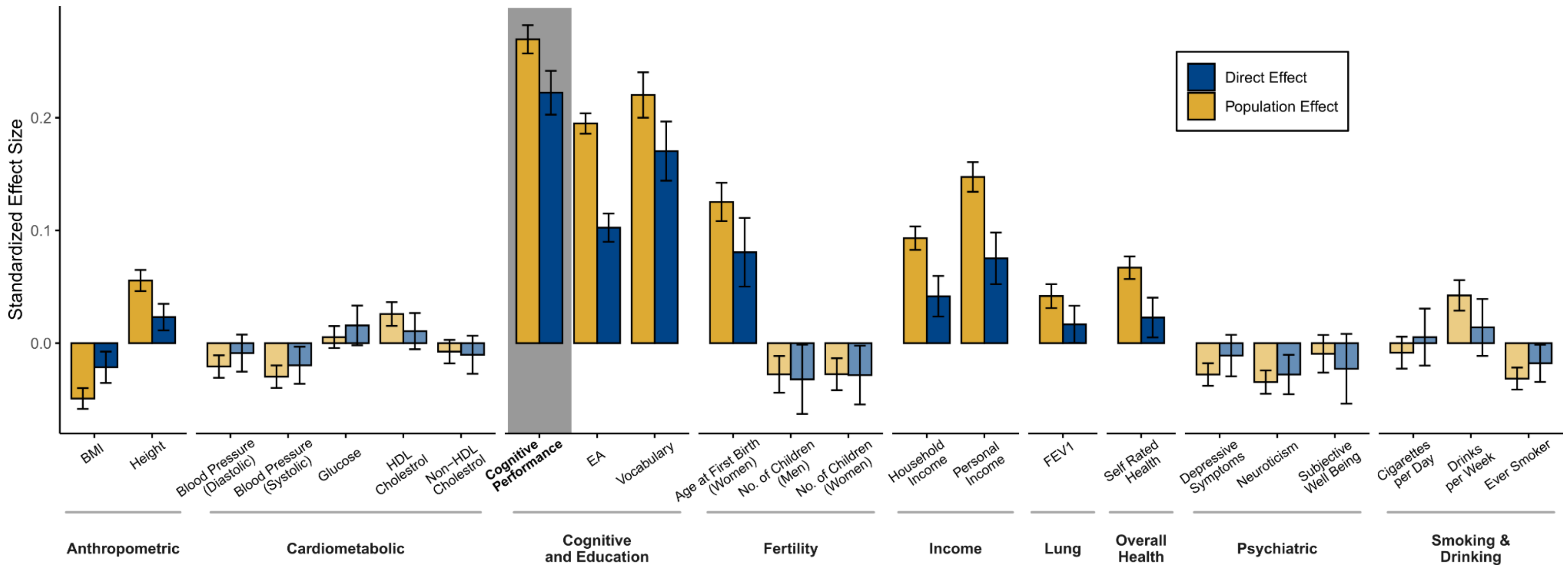

Supplementary Figure 3. Recoding of EA in the UK Biobank. Panels **(a)**: Distribution of NVQs awarded by UK qualification level; **(b)**: distribution of age left full-time education (FTE) for individuals whose highest qualification is "NVQ or HND or HNC or equivalent"; **(c)**: average age left FTE by EA (EA2/EA3 coding); **(d)**: proportion of households earning £52,000 or more per year by EA (EA2/EA3 coding); **(e)**: average age left FTE by EA (EA4 coding); **(f)**: proportion of households earning £52,000 or more per year by EA (EA4 coding). In **(c)** and **(e)**, age left FTE was set to 22 for all college graduates. In **(e)** and **(f)**, values of EA (EA4 coding) for which there were fewer than 1,500 observations were excluded.

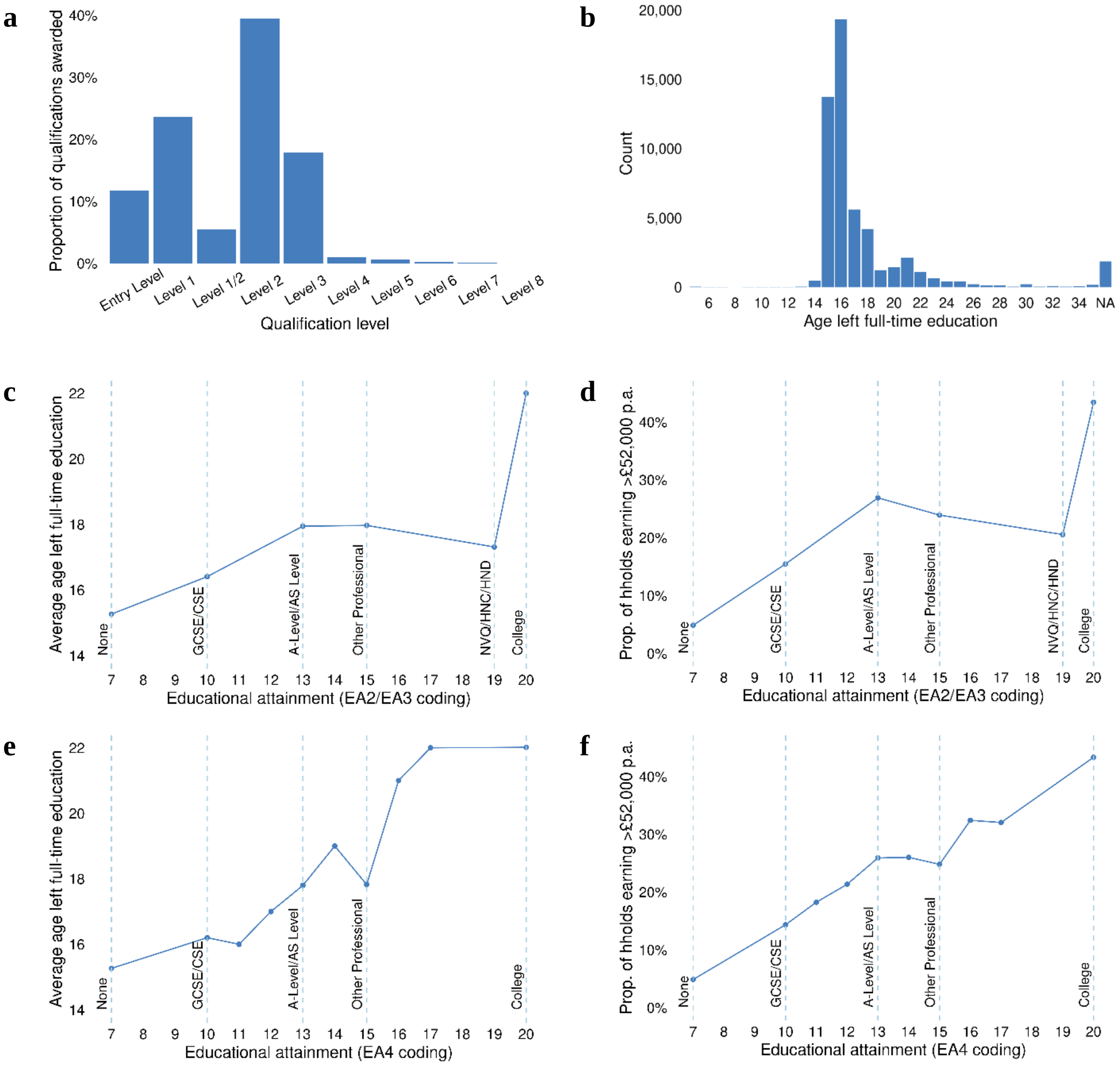

Supplementary Figure 4. Comparison of autosomal and X-chromosomal association results. “Chromosome length” is calculated as the difference between the minimum and maximum base-pair position of SNPs on the chromosome. “Effective number of loci (M_{eff})” is calculated from the M SNPs using data from the UK Biobank (N = 329,358 individuals). For each chromosome, “by-chromosome SNP heritability” is calculated as $h^2 = \frac{(\overline{\chi^2}-1)M_{eff}}{\bar{N}}$ where $\overline{\chi^2}$ is the mean χ^2 test statistic for that chromosome and $\bar{N}$ is the average GWAS sample size. “Number of lead SNPs” is calculated by applying our clumping algorithm (see **Supplementary Note** section 2.2.6) to the set of genome-wide-significant SNPs. The dashed line is the best fit from a regression of the points in the plot with the intercept constrained to zero. The value r^2 is the squared correlation coefficient of the points in each plot. Panels **(a)**: Chromosome length vs. heritability; **(b)**: Effective number of loci vs. heritability; **(c)**: Chromosome length vs. number of lead SNPs; **(d)**: Effective number of loci vs. number of lead SNPs.

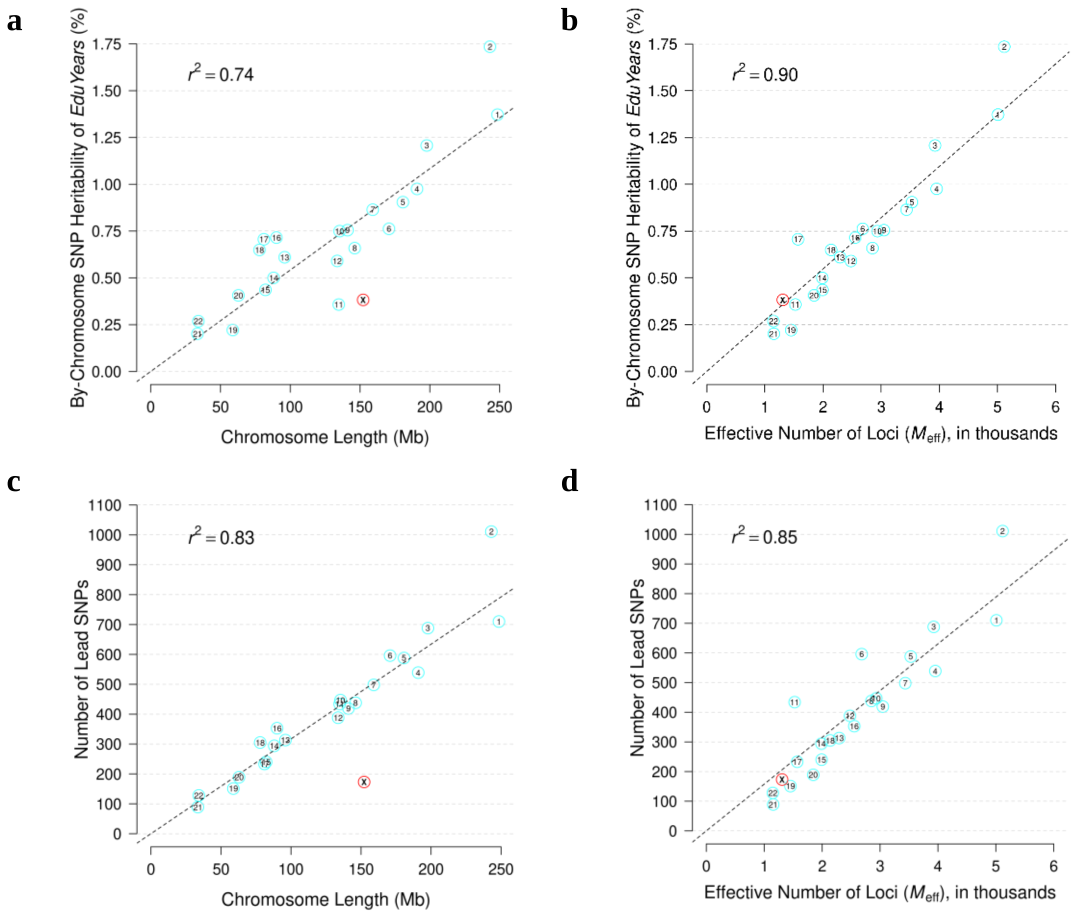

Supplementary Figure 5. Quantile-quantile plots for the overall dominance GWAS meta-analysis. The panels display Q-Q plots, which show the -log$_{10}$(P-values) based on a two-sided Z-test, for **(a)** all SNPs and **(b)** SNPs grouped by minor allele frequency (MAF): low frequency (1-5%) and common (>5%). The plots and λ_{GC} numbers are based on the unadjusted GWAS summary statistics (i.e., with standard errors that were *not* inflated by the square root of the estimated LD score intercept). The dotted line represents the expected -log$_{10}$(P-values) under the null hypothesis. The gray shaded areas in the Q-Q plots represent the 95% confidence intervals under the null hypothesis.

a

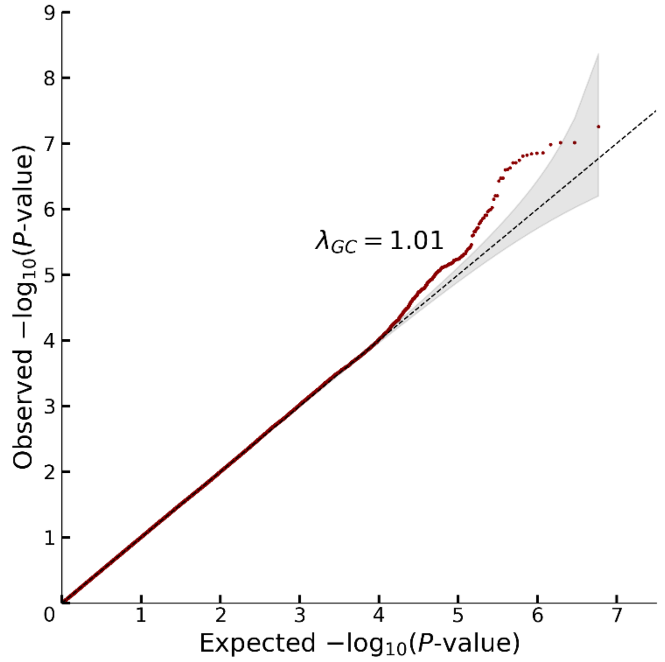

b

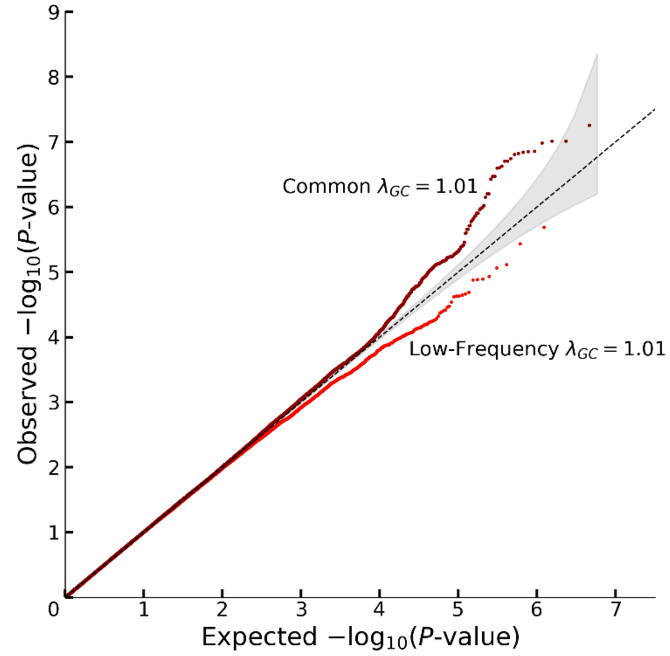

Supplement: Supplementary file 1 — Supplementary Note and Supplementary Figures 1–5. [file 41588_2022_1016_MOESM1_ESM.pdf]
