# Supplementary material for: Polygenic prediction of educational attainment within and between families from genome-wide association analyses in 3 million individuals
Source: Nat Genet. 2022 Mar 31;54(4):437–49. doi: 10.1038/s41588-022-01016-z (PMC9005349; doi:10.1038/s41588-022-01016-z)
Supplement: Supplementary file 2 — Reporting Summary [file 41588_2022_1016_MOESM2_ESM.pdf]

nature research

Corresponding author(s): Daniel J. Benjamin

Last updated by author(s): Jan 10, 2022

Reporting Summary

Nature Research wishes to improve the reproducibility of the work that we publish. This form provides structure for consistency and transparency in reporting. For further information on Nature Research policies, see our Editorial Policies and the Editorial Policy Checklist.

Statistics

For all statistical analyses, confirm that the following items are present in the figure legend, table legend, main text, or Methods section.

n/a	Confirmed	
☐	☒	The exact sample size (*n*) for each experimental group/condition, given as a discrete number and unit of measurement
☐	☒	A statement on whether measurements were taken from distinct samples or whether the same sample was measured repeatedly
☐	☒	The statistical test(s) used AND whether they are one- or two-sided<br>*Only common tests should be described solely by name; describe more complex techniques in the Methods section.*
☐	☒	A description of all covariates tested
☐	☒	A description of any assumptions or corrections, such as tests of normality and adjustment for multiple comparisons
☐	☒	A full description of the statistical parameters including central tendency (e.g. means) or other basic estimates (e.g. regression coefficient) AND variation (e.g. standard deviation) or associated estimates of uncertainty (e.g. confidence intervals)
☐	☒	For null hypothesis testing, the test statistic (e.g. *F*, *t*, *r*) with confidence intervals, effect sizes, degrees of freedom and *P* value noted<br>*Give P values as exact values whenever suitable.*
☐	☒	For Bayesian analysis, information on the choice of priors and Markov chain Monte Carlo settings
☒	☐	For hierarchical and complex designs, identification of the appropriate level for tests and full reporting of outcomes
☐	☒	Estimates of effect sizes (e.g. Cohen's *d*, Pearson's *r*), indicating how they were calculated

Our web collection on statistics for biologists contains articles on many of the points above.

Software and code

Policy information about availability of computer code

Data collection	No software was used for data collection.
Data analysis	The following software packages were used for data analysis: Python version 3.7.4 with packages pandas 0.25.1, scipy 1.3.1, numpy 1.17.2, matplotlib 3.1.1 and argparse 1.1 (https://anaconda.org); R version 4.0.3 with packages EasyQC 9.2, plotrix 3.7.8, tidyr 1.1.3 and readstata13 0.9.2, R version 3.6 (https://www.r-project.org); GCTA 1.93.2beta (https://yanglab.westlake.edu.cn/software/gcta/#Overview); GCTB 2.03 (https://cnsgenomics.com/software/gctb/#Overview); Stata 16.1 (https://www.stata.com); PLINK 1.9 (https://www.cog-genomics.org/plink/1.9); PLINK 2 (https://www.cog-genomics.org/plink/2.0); LDpred 1.0.11 (https://github.com/bvilhjal/ldpred); METAL release 2011-03-25 (https://genome.sph.umich.edu/wiki/METAL_Documentation); BOLT-LMM 2.3 (https://alkesgroup.broadinstitute.org/BOLT-LMM/BOLT-LMM_manual.html); LDSC 1.0.1 (https://github.com/bulik/ldsc); SNIPar (https://github.com/AlexTISYoung/SNIPar/tree/EA4).

For manuscripts utilizing custom algorithms or software that are central to the research but not yet described in published literature, software must be made available to editors and reviewers. We strongly encourage code deposition in a community repository (e.g. GitHub). See the Nature Research guidelines for submitting code & software for further information.

Data

Policy information about availability of data

All manuscripts must include a data availability statement. This statement should provide the following information, where applicable:

- Accession codes, unique identifiers, or web links for publicly available datasets
- A list of figures that have associated raw data
- A description of any restrictions on data availability

GWAS summary statistics can be downloaded from http://www.thessgac.org/data subject to a Terms of Use to ensure responsible use of the data. We provide association results for all SNPs that passed quality-control filters in autosomal, X chromosome, and dominance GWAS meta-analyses that excludes the research

participants from 23andMe. SNP-level summary statistics from analyses based entirely or in part on 23andMe data can only be reported for up to 10,000 SNPs. For the complete dominance GWAS meta-analysis, which includes 23andMe, clumped results for the 1,000 SNPs with the smallest P values are provided. For the complete autosomal and X chromosome GWAS meta-analyses, respectively, clumped results for the 8,617 and 143 SNPs with P < 10-5 are provided; this P value threshold was chosen such that the total number of SNPs across the analyses that include data from 23andMe does not exceed 10,000. The full GWAS summary statistics from 23andMe will be made available through 23andMe to qualified researchers under an agreement with 23andMe that protects the privacy of the 23andMe participants. Please visit https://research.23andme.com/collaborate/#dataset-access/ for more information and to apply to access the data.

Field-specific reporting

Please select the one below that is the best fit for your research. If you are not sure, read the appropriate sections before making your selection.

☐ Life sciences ☒ Behavioural & social sciences ☐ Ecological, evolutionary & environmental sciences

For a reference copy of the document with all sections, see nature.com/documents/nr-reporting-summary-flat.pdf

Behavioural & social sciences study design

All studies must disclose on these points even when the disclosure is negative.

Study description	This is a genome-wide association study (GWAS) meta-analysis of educational attainment (EA) in a sample of ~3 million individuals. All data used in this study (genetic and phenotype data) are quantitative.
Research sample	The research sample consists of ~3 million individuals from 71 research cohorts. We meta-analyzed three sets of summary statistics: publicly available results from Lee et al. (2018) that exclude 23andMe and UKB (N = 324,162), new association results from 23andMe (N = 2,272,216), and new association results from a GWAS we conducted in UKB with an improved coding of the EA measure (N = 441,121). The large study sample was required for us to have sufficient statistical power in detecting single nucleotide polymorphisms (SNPs) with small effect sizes and for our follow-up analyses.
Sampling strategy	We obtained the largest sample we could.
Data collection	Data collection was performed independently by each participating cohort.
Timing	Data was collected from multiple cohorts with variable data collection periods.
Data exclusions	All observations reporting less than seven years of schooling were dropped to exclude outliers (there were fewer than 50 such observations; see Supplementary Note 1.1.4).
Non-participation	No participants dropped out or declined participation.
Randomization	Participants were not allocated into experimental groups.

Reporting for specific materials, systems and methods

We require information from authors about some types of materials, experimental systems and methods used in many studies. Here, indicate whether each material, system or method listed is relevant to your study. If you are not sure if a list item applies to your research, read the appropriate section before selecting a response.

Materials & experimental systems

n/a	Involved in the study	
☒	☐	Antibodies
☒	☐	Eukaryotic cell lines
☒	☐	Palaeontology and archaeology
☒	☐	Animals and other organisms
☐	☒	Human research participants
☒	☐	Clinical data
☒	☐	Dual use research of concern

Methods

n/a	Involved in the study	
☒	☐	ChIP-seq
☒	☐	Flow cytometry
☒	☐	MRI-based neuroimaging

Human research participants

Policy information about studies involving human research participants

Population characteristics	See above.

Recruitment	Recruitment strategies were particular to each cohort.
Ethics oversight	All analyses are on anonymized, secondary data. Nonetheless, the analyses reported in the paper fall under National Bureau of Economic Research IRB protocols 19_434, 19_465, and 20_041.

Note that full information on the approval of the study protocol must also be provided in the manuscript.
