# Supplementary material for: Polygenic prediction of educational attainment within and between families from genome-wide association analyses in 3 million individuals
Source: Nat Genet. 2022 Mar 31;54(4):437–49. doi: 10.1038/s41588-022-01016-z (PMC9005349; doi:10.1038/s41588-022-01016-z)
Supplement: Supplementary file 5 — Frequently asked questions. [file 41588_2022_1016_MOESM5_ESM.pdf]

Frequently Asked Questions (FAQs)

This document provides information about the study:

Okbay *et al.* (2021) "Polygenic prediction within and between families from a 3-million-person GWAS of educational attainment" *Nature Genetics*, submitted.

The document was written by Daniel Benjamin, David Laibson, Michelle N. Meyer, and Patrick Turley. It draws from and builds on the FAQs for earlier SSGAC papers. It has the following sections:

1. **Background**
2. **Study design and results**
3. **Social and ethical implications of the study**
4. **Appendices**

For clarifications or additional questions, please contact Daniel Benjamin (daniel.benjamin@gmail.com).

Table of Contents

1 Background 3

1.1 Who conducted this study? What are the group's overarching goals? 3

1.2 The current study focuses on an outcome called "educational attainment." What is educational attainment? 4

1.3 What is a GWAS? 4

1.4 Are the SNPs identified in a GWAS "causal" (i.e., would a change in the SNPs someone has, if everything else stayed the same, cause a person's life to change)? 5

1.5 In what sense do the SNPs identified in a GWAS "predict" the outcome of interest? What do you mean by "effect size"? 7

1.6 What is a polygenic index? 8

1.7 Why conduct a GWAS of educational attainment? 8

1.8 What was already known about the relationships between genes and educational attainment prior to this study? 10

2 Study design and results 12

2.1 What did you do in this paper? How was the study designed? Why was the study designed in this way? 12

2.2 What are common pitfalls in GWASs? What precautions did you take against them? 13

2.3 What did you find in the main GWAS of educational attainment? 14

2.4 How predictive is the polygenic index developed in this study? 15

2.5 Why is the polygenic index less predictive in samples of African genetic ancestries than in samples of European genetic ancestries? 18

2.6 What did you find in the analysis of disease risk? 19

2.7 What did you find in the family-based analyses? 20

2.8 What did you find in the analysis of assortative mating? 23

2.9 What did you find in the analysis of the X chromosome? 25

2.10 What did you do in the "dominance GWAS" of educational attainment? What did you find? 25

3 Ethical and social implications of the study 27

3.1 Did you find "the gene for" educational attainment? 27

3.2 Well, then, did you find "the genes for" educational attainment? 27

3.3 Does this study show that an individual's level of educational attainment (or any other outcome) is determined, or fixed, at conception? Do genes determine the choices we make and who we become? 29

3.4 Can the polygenic index from this paper be used to accurately predict a particular person's educational attainment? 30

3.5 Can your polygenic index be used for research studies in diverse genetic ancestry populations? 31

3.6 Should practitioners (e.g., in education or other domains) use the results of this study to make decisions? 32

3.7 Could this kind of research lead to discrimination against, or stigmatization of, people with the relevant genetic variants? What has been done to help avert the potential harms of this research? 32

Additional reading and references 35

1 Background

1.1 Who conducted this study? What are the group's overarching goals?

The authors of the study are members of the Social Science Genetic Association Consortium (SSGAC). The SSGAC is a multi-institutional, international research group that aims to identify statistically robust links between genetics and social-science-relevant outcomes. These outcomes include behavior, preferences, and personality. They are traditionally studied by social and behavioral scientists (e.g., economists, psychologists, sociologists) but are often also of interest to health and other researchers.

The SSGAC was formed in 2011 to overcome a specific set of scientific challenges. Most social-scientific outcomes are associated with thousands of genetic differences called single-nucleotide polymorphisms (SNPs, pronounced "snips"). A SNP is a place in the genome where people differ genetically from each other (see FAQ 1.3). Although when you add up thousands of SNPs, their collective predictive power can be meaningful (see FAQs 1.6 & 2.4), we now know that almost every one of these SNPs has an extremely weak association with a particular social-scientific outcome on its own. To identify specific SNPs with such weak associations, scientists must study at least hundreds of thousands of people (to separate weak signals from noise, and thereby avoid finding false positives). One promising strategy for doing this is for many investigators to pool their data into one large study. This approach has borne considerable fruit when used by medical geneticists interested in a range of medical conditions (Visscher *et al.*, 2017). Most of these advances would not have been possible without large research collaborations between multiple research groups interested in similar questions. The SSGAC was formed in an attempt by social scientists to adopt this research model.

The SSGAC is organized as a working group of the Cohorts for Heart and Aging Research in Genomic Epidemiology (CHARGE), a successful medical consortium. (In genetics research, "cohort" is a term that means "dataset.") The SSGAC was founded by three social scientists—Daniel Benjamin (University of California, Los Angeles), David Cesarini (New York University), and Philipp Koellinger (Vrije Universiteit Amsterdam)—who believe that studying SNPs associated with social-scientific outcomes can have substantial positive impacts across many research fields (see FAQ 1.7).

The Advisory Board for the SSGAC is composed of prominent researchers representing various disciplines: Dalton Conley (Sociology, Princeton University), George Davey Smith (Epidemiology, University of Bristol), Tõnu Esko (Molecular Biology and Human Genetics, University of Tartu and Estonian Genome Center), Albert Hofman (Epidemiology, Harvard University), Robert Krueger (Psychology, University of Minnesota), David Laibson (Economics, Harvard University), James Lee (Psychology, University of Minnesota), Sarah Medland (Genetic Epidemiology, QIMR Berghofer Medical Research Institute), Michelle Meyer (Bioethics and Law, Geisinger Health System), and Peter Visscher (Statistical Genetics, University of Queensland).

The SSGAC is committed to the principles of reproducibility and transparency. Prior to conducting studies, power calculations are carried out to determine the necessary sample size for the analysis (assuming realistically small effect sizes associated with individual genetic variants). Whenever possible, we pre-

register our analyses at OSF (formerly Open Science Framework). Major SSGAC publications are usually accompanied by a FAQ document (such as this one). The FAQ document is written to communicate what was found less tersely and technically than in the paper, as well as to emphasize what can and cannot be concluded from the research findings more broadly and how they should and shouldn't be used. FAQ documents produced for SSGAC publications are available on the SSGAC website.

In addition to educational attainment, SSGAC-affiliated papers have studied subjective well-being, reproductive behavior, risk tolerance, and dietary intake. The SSGAC website contains a list of our research publications, including papers in *Science, Nature, Nature Genetics, Nature Human Behaviour, Proceedings of the National Academy of Sciences, Psychological Science,* and *Molecular Psychiatry*.

1.2 The current study focuses on an outcome called "educational attainment." What is educational attainment?

Educational attainment is the number of years of formal education a person has completed, starting with kindergarten or its equivalent. The vast majority of people in our sample are at least age 30; almost all of the people that we study have completed their formal education. Although educational attainment is most strongly influenced by social and other environmental factors (see FAQ 1.8), it is also influenced by thousands of genes. People vary considerably in how much education they complete. Education is recognized throughout the social and biomedical sciences as an important "predictor" (see FAQ 1.5) of many other life outcomes, such as income, occupation, health, and longevity (Ross and Wu, 1995; Cutler and Lleras-Muney, 2010). Educational attainment has also been among the relatively few social-scientific outcomes for which it is feasible to conduct a large-scale genome-wide study, because educational attainment is frequently measured in cohorts, including medical cohorts, due to its robust association with health. The current study is also based on a large sample of research participants of the personal genomics company 23andMe, which asks participants a survey question about educational attainment. A large-scale study is necessary (but not sufficient) to generate scientific findings that are reproducible.

1.3 What is a GWAS?

In a genome-wide association study (GWAS, pronounced JEE-wahs), scientists look across the entire human genome at genetic differences among people to see whether any of these differences are, *on average*, associated statistically with higher or lower levels of some outcome—for instance, more or less cancer, height, or risk tolerance. Typically, and in our studies, such analyses focus on places in the human genome where people commonly differ: so-called single-nucleotide polymorphisms (SNPs). At a given SNP located on a particular copy of a chromosome, each of us has one of the four genetic base pairs (A-T, T-A, C-G, or G-C), which is called an "allele." We inherit one of each chromosome from our biological father and one from our biological mother, so at each SNP, we inherit one allele from each biological parent and hence have two alleles in total. In some cases, we inherit the same allele from each parent, and in other cases, we inherit one allele from one parent and a different allele from the other parent. In a GWAS, researchers look to see whether particular alleles are associated statistically with having more or less of some outcome.

Although there are tens of millions of sites where SNPs are located in the human genome, GWASs typically investigate only SNPs that can be measured (or imputed) with a high level of accuracy. These days, such

procedures usually yield millions of SNPs that together capture most common genetic variation across people.

When the SSGAC conducts a GWAS, every participating cohort uploads the (within-cohort) statistical associations between the outcome—for example, educational attainment—and each SNP that was measured in the genomes of the individuals in the cohort. The cohort-level results do not contain individual-level data—just summary statistics about these within-cohort statistical associations. The SSGAC then combines these cohort results to produce the overall GWAS results. By using existing datasets and combining cohort-level results, we can study the genetics of ~3 million people at very low cost. The SSGAC publicly shares overall, aggregated results (subject to some Terms of Service; see FAQ 3.7) so that other scientists can build on this work. These publicly available data have already catalyzed many research projects and analyses across the social and biomedical sciences (see FAQ 1.7 for examples).

GWASs have been a successful research strategy for identifying genetic variants associated with many outcomes and diseases, including body height (Wood *et al.*, 2014) , BMI (Locke *et al.*, 2015), Alzheimer's disease (Lambert *et al.*, 2013), and schizophrenia (Ripke *et al.*, 2014). It has also recently been used to identify genetic variants associated with a variety of health-relevant social-science outcomes, such as the number of children a person has (Barban *et al.*, 2016), happiness (Okbay, Baselmans, *et al.*, 2016; Turley *et al.*, 2018), and educational attainment (Rietveld *et al.*, 2013; Okbay, Beauchamp, *et al.*, 2016; Lee *et al.*, 2018).

1.4 Are the SNPs identified in a GWAS "causal" (i.e., would a change in the SNPs someone has, if everything else stayed the same, cause a person's life to change)?

GWASs identify alleles that are associated with the outcome and cannot distinguish whether the associations are causal or not. While such an association can arise if a SNP causally influences the outcome, it is not necessarily the case that all associations between SNPs and outcomes are causal. Here are several non-causal reasons why a SNP may be associated statistically (i.e., correlated) with an outcome. First, SNPs are often highly correlated with other, nearby SNPs on the same chromosome. As a result, when one or more SNPs in a region causally influence an outcome (in that particular environment), many *non-causal* SNPs in that region may also be identified as statistically associated with the outcome. When GWAS results are analyzed, researchers typically report results for the SNP in a region that shows the strongest evidence of association. Even if there is a causal SNP, GWASs may not identify that particular SNP. In fact, the causal SNP may not have even been included among the SNPs that were originally measured directly for the study. For example, GWASs that focus on common SNPs would not be able to identify rare or structural genetic differences between people (e.g., deletions or insertions of an entire genetic region) that are causal, but GWASs may identify SNPs that are correlated with these unobserved sources of genetic variation.

Second, at a particular SNP, the frequencies of different alleles might vary systematically across environments. If those environmental factors are not accounted for in the association analyses, some of the associations found may be spurious—that is, the result of coincidence or of a third factor. Consider the well-known example of a GWAS of chopstick use (Lander & Schork 1994; Hamer and Sirota, 2000). Because alleles are, *by chance*, more and less common in different populations, some alleles are more common in people with Asian genetic ancestries. At the same time, for cultural reasons, practices like

chopstick use are often more common in some populations than in others. Both alleles and social outcomes like chopstick use, then, are distributed unevenly among people with different genetic ancestries. As a result, a "chopstick GWAS" would almost certainly find some alleles that are associated statistically with chopstick use, but these associations would be coincidental, and the alleles would not *cause* chopstick use. This is the problem of "population stratification bias" discussed in FAQ 2.2. GWAS researchers have a number of strategies for addressing the challenges posed by population stratification bias (see FAQs 2.7 & 3.5).

Even in studies such as ours that attempt to account for diversity in genetic ancestry, allele frequencies may nonetheless vary systematically with social practices and other environmental factors even *within* a group of people of similar genetic ancestry. For example, an allele that is associated with improved educational outcomes in the parental generation may have downstream effects on parental income and other factors known to influence children's educational opportunities and outcomes (such as neighborhood characteristics). This same allele is likely to be inherited by the children of these parents, creating a correlation between the presence of the allele in a child's genome and the extent to which the child was reared in a specific kind of environment. A recent study of Icelandic families showed that the parental allele that is *not* passed on to the child is still associated with the child's educational attainment, suggesting that GWAS results for educational attainment partly represent these intergenerational pathways (Kong *et al.*, 2018). Our family-based analyses yield results that are consistent with this conclusion (see FAQ 2.7).

There are also cases where a SNP *may* indeed be causal, but not in the way that some people may think when they hear that genes "cause" an outcome. In these cases, SNPs' effects on an outcome may be indirect, so a SNP that may be "causal" in one environment may have a diminished effect or no effect at all in other environments. For example, the nicotinic acetylcholine receptor gene cluster on chromosome 15 is associated with lung cancer (Amos *et al.*, 2008; Hung *et al.*, 2008; Thorgeirsson *et al.*, 2008). From this observation alone we cannot conclude that these genetic variants cause lung cancer through some direct *biological* mechanism. In fact, it is likely that one version of this gene, which is part of the nicotinic acetylcholine receptor gene cluster that affects nicotine metabolism, increases lung cancer risk through effects on smoking behavior. In a tobacco-free environment, it is plausible that many of the associations would be substantially weaker and perhaps disappear altogether. Thus, even *if* we have credible evidence that a specific association is not spurious, it is entirely possible that the SNP in question influences the outcome through channels that most people would call environmental (e.g., smoking). Nearly forty years ago, the sociologist Christopher Jencks criticized the widespread tendency to mistakenly treat environmental and genetic sources of variation as mutually exclusive (see also Turkheimer, 2000). As the example of smoking illustrates, and as Jencks (1980) explains, it is often overly simplistic to assume that "genetic explanations of behavior are likely to be exclusively physical explanations while environmental explanations are likely to be social" (p.723).

In general, a GWAS is just one step in a longer, often complex process of identifying causal pathways, but the results of a large-scale GWAS are a useful tool for that purpose and often lead to novel and important insights (Visscher *et al.*, 2017). In other words, GWAS results provide important signals as to where scientists should invest future in-depth research to understand why the association exists (see also FAQ 3.6).

1.5 In what sense do the SNPs identified in a GWAS "predict" the outcome of interest? What do you mean by "effect size"?

When we and other scientists say that SNPs—and other variables, such as demographics or environmental factors—"predict" certain outcomes, we mean that people with particular alleles will tend—with *some* degree of likelihood, and only *on average*—to complete in the future or to have already completed more formal education, while people who carry other alleles will tend—again with *some* degree of likelihood, and only *on average*—to complete less formal education (see FAQ 1.8).

Our use of "predict" in this sense differs in several important ways from how "predict" is sometimes used in standard language (e.g., outside of social science research papers). First, we do not mean that the presence of an allele guarantees an outcome with certainty, or even with a high degree of likelihood. Rather, we mean that the SNP is, on average across people, associated statistically with an outcome. In other words, on average, people with one allele at that SNP have a higher likelihood of the outcome compared to people with the other allele. Scientists describe a SNP as statistically "predictive" of an outcome even if it has only a *weak* association with the outcome—as is the case, for instance, with every SNP that we identify that is associated with educational attainment.

Second, in standard language, "prediction" usually refers to the future. In contrast, when scientists say that SNPs "predict" an outcome, they mean that they expect to see the association in *other data*. "Other data" means data that aren't part of the current study—regardless of whether those data will be collected in the future or have already been collected. In other words, in social science, it makes perfect sense to ask how well a SNP predicts an outcome that has already occurred, like how many years of education were attained by older adults.

Finally, in standard language, a "prediction" is often an unconditional guess about what will happen. Instead of meaning it unconditionally, scientists mean that they expect to see an association in other data collected—but only if those data will be or were collected in an environment that is approximately the same as the environment in which the original data were collected. In the example given in FAQ 1.4, in which a SNP is associated with lung cancer due to its effect on smoking, we might *not* expect the SNP to be predictive of lung cancer in an environment where cigarettes and other smoked tobacco products are absent.

"Effect size" is a scientific term that refers to the magnitude of the predicted difference in the outcome resulting from having one allele of a SNP as opposed to the other possible allele (see FAQ 2.3). For example, the average SNP identified in the current study is associated with only 1.4 more weeks of school *on average*. (Note that the association might average out to 1.4 weeks of school if, for example, one of the alleles is associated with an additional year of school for 3.5% of people and no additional school for 96.5% of people.) The use of the word "effect" is *not* intended to imply that the strength of the *association* between a SNP and educational attainment is necessarily a measure of the SNP's *causal* effect on educational attainment (see FAQ 1.4).

1.6 What is a polygenic index?

The results of a GWAS can be used to create a "polygenic index," which sums up the net "effects" (see FAQ 1.5) of many SNPs from across an individual's genome on the GWAS outcome. (We prefer the term "polygenic index" over the more common terms "polygenic score" and "polygenic risk score," because the words "score" and "risk" can convey a value judgment where none is intended.) Because a polygenic index aggregates the information from many SNPs, it can "predict" (see FAQ 1.5) far more of the variation among individuals for the GWAS outcome than any single SNP. (Note, however, that even polygenic indexes are not good predictors of outcomes for one person; see FAQ 2.4.) Often, the polygenic indexes with the most predictive power are those created using *all* the (millions of) SNPs studied in a GWAS. The larger the GWAS sample size, the greater the predictive power (in other, independent samples) of a polygenic index constructed from the GWAS results. More precisely, the GWAS results are used to create a *formula* for constructing a polygenic index based on the effects on the outcome of having each allele. Using this formula, a polygenic index can then be constructed for any individual for whom there is genetic data that includes the SNPs that were used to construct the index. Indeed, some of the value of a GWAS is that the polygenic index it produces can be used in subsequent studies conducted in other samples.

We do not refer to the association between a polygenic index and an outcome as "causal." That is because the polygenic index is composed of many SNPs, and while some of these may be causal, some (or, in principle, all) may not be causal (see FAQ 1.4). Some of the analyses in our paper are designed to quantify *how much* of the predictive power of the polygenic index for educational attainment is due to causal effects of SNPs (see FAQ 2.1).

1.7 Why conduct a GWAS of educational attainment?

We are motivated to conduct this research because we believe it can be fruitful for the social sciences and health research. In addition to the specific findings of our paper, which are discussed in Section 2 of these FAQs, the results of a GWAS of educational attainment also provide inputs for other research. In our view, some of the most valuable uses of the results will be to improve our ability to study the effects of *environments*. Because this may be counterintuitive, we will give a few examples of what we mean

One example is using a polygenic index to *control for*, or hold constant, genetic influences when studying the effect of an environment. Doing so can be important when the study is correlational, but it can be valuable even in a study where the environmental variable is randomly assigned. Suppose researchers are studying the effect of an educational intervention, such as providing free preschool to economically disadvantaged children, on subsequent school achievement. Because a year of preschool is expensive, the sample sizes of such studies have been small (e.g., Weikart and Perry Preschool Project, 1967). In a study where a year of free preschool is randomly given to half of the children participating in the study, control variables are not needed in order to get an estimate of the effect of preschool on average—but control variables, such as gender, age, and parental socioeconomic status, are typically included in order to make the estimate more precise (by removing some of the background "noise" that makes it harder to detect the effect of the intervention). In effect, using the polygenic index as an additional control variable can allow the researchers to learn more from the same-size sample. The value of using the polygenic index as a control depends on how much predictive power it has over the set of other control variables used. In our first GWAS

of educational attainment (Rietveld *et al.*, 2013, Supplementary Materials section 8), we conducted calculations to quantify these gains. For the purposes of illustration, suppose control variables other than the polygenic index capture 10% of the variation in the outcome, and the polygenic index captures an *additional* 12% of the variation. Then to attain any given level of precision for the estimate of the effect of preschool, including the polygenic index as a control variable reduces the required sample size for the study by 13%. Relative to the cost of providing preschool to additional research participants—for instance, estimated to be $19,208.61 (in 2010 dollars) per child for the two-year Perry preschool study (Heckman *et al.*, 2010)—genotyping participants can be highly cost effective. (Genotyping currently costs roughly $30/person, with this cost falling quickly over time.) There are currently only a few examples of polygenic indexes used in this way (e.g., Davies et al. 2018), because polygenic indexes have only recently attained enough predictive power to usefully serve as control variables. We anticipate that this type of application will become widespread in future social-scientific studies.

Another example is using the results of a GWAS of educational attainment to study how parenting and other features of a child's rearing environment influence his or her developmental outcomes. This idea was pioneered in a paper by Kong *et al.* (2018), who studied SNPs identified in one of our earlier GWASs of educational attainment (Okbay, Beauchamp, *et al.*, 2016). Kong et al. showed that the alleles of the mother and father that are *not* transmitted to a child are nevertheless related to the child's outcomes, including the child's educational attainment. Because the child did not inherit these alleles, their association with the child's outcomes cannot be due to genetic influences on the child. Instead, their association with the child's outcomes must be due to their effects on the parents, which in turn affects the environment in which the child is reared. While Kong et al. did not pin down the specific pathways that account for these associations, there are many interesting possibilities that can be explored in future work; for instance, parents with these alleles are more likely to attend school longer and earn higher incomes, which may enable them to provide educational advantages to their children. Kong et al.'s methodology can also be used to address other questions, such as whether the non-transmitted alleles of the mother or father are more strongly with the child's outcomes.

Much more briefly, here are some other examples of how results from our earlier GWASs of educational attainment (Rietveld *et al.*, 2013; Okbay, Beauchamp, *et al.*, 2016; Lee *et al.*, 2018) conducted in much smaller sample sizes (see also FAQ 1.8) have been used:

- examine the genetic overlap between educational attainment and ADHD, schizophrenia, Alzheimer's disease, intellectual disability, cognitive decline in the elderly, brain morphology, and longevity (Pickrell *et al.*, 2016; Riccardo E. Marioni *et al.*, 2016; Warrier *et al.*, 2016; Anderson *et al.*, 2017);
- help us better identify possible genetic subtypes of schizophrenia (Bansal *et al.*, 2017);
- provide insights into the genetics of brain development and function (Lee *et al.*, 2018);
- explore why educational attainment appears to be protective against coronary artery disease (Tillmann *et al.*, 2017) and obesity (van Kippersluis and Rietveld, 2018);
- study why specific SNPs predict educational attainment. For example, it appears that some genetic effects on educational attainment operate through associations with cognitive performance and outcomes such as self-control (Belsky *et al.*, 2016), which in turn affect educational attainment;

- study how the effects of genes on education differ across environmental contexts (Schmitz and Conley, 2017; Barcellos, Carvalho and Turley, 2018; Cheesman *et al.*, 2020); and
- determine the limits of genetic influences and debunk cultural myths about group differences (e.g., between men and women, see FAQs 2.9 & 3.7) (Houmark, Ronda and Rosholm, 2020).

By making the results of our analyses publicly available at https://www.thessgac.org/data, we hope to facilitate this and other valuable work by other researchers.

1.8 What was already known about the relationships between genes and educational attainment prior to this study?

Educational attainment is strongly influenced by social and other environmental factors. For example, holding all other influences equal, those who live in communities where education (at least beyond a certain level) is relatively expensive are less likely to obtain a high level of educational attainment. Even when education is free or heavily subsidized, full-time education implies an opportunity cost that not everyone is equally able to bear: some individuals, due to a variety of family or economic circumstances, will face more pressure than others to leave school and enter the work force. More generally, educational outcomes are strongly influenced by environmental factors such as social norms, early-life educational experiences, economic opportunity, and many forms of bias and discrimination that make it harder for some people to succeed or stay in school.

A variety of findings—from twin, family, and GWASs—suggest that genetic factors predict some of the differences across people in their educational attainment (Heath *et al.*, 1985; Silventoinen *et al.*, 2004; Branigan *et al.*, 2013). Studies have found repeatedly that identical twins raised in the same home are substantially more similar to each other in their educational attainment than fraternal twins (or other full siblings) reared together. Full siblings reared together are, in turn, more similar than half siblings reared together who, in turn, are more similar than genetically unrelated siblings reared together (e.g., siblings who are conventionally unrelated, typically because at least one of them is adopted) (Sacerdote, 2007, 2011; Cesarini and Visscher, 2017). The studies have also provided strong evidence that the so-called "common environment" (the environmental factors shared by siblings raised in the same household) can have long-lasting effects on educational outcomes. In Sweden, the educational outcomes of adopted (i.e., genetically unrelated) brothers reared in the same households are about as similar as the educational outcomes of full siblings reared in separate homes (Cesarini and Visscher, 2017). A study of Korean-American adoptees finds that adoptees assigned to households where both parents had college degrees were 16 percentage points more likely to attend college than children assigned to families in which neither parent completed college (Sacerdote, 2007).

Research (like the current study) using molecular genetic data—data that measures each person's DNA and can be used to identify differences among people at the molecular level—has similarly estimated that SNPs may jointly predict up to 20% of the variation in educational attainment across individuals (Rietveld *et al.*, 2013). Prior GWASs have begun to identify some of those SNPs. In the SSGAC's first major publication (Rietveld *et al.*, 2013), we conducted a GWAS in a sample of roughly 100,000 people and identified three SNPs that were statistically associated with educational attainment. In 2016, the SSGAC published another GWAS of educational attainment, this time in a sample of around 300,000 people (Okbay, Beauchamp, *et*

al., 2016). We found that 74 SNPs were associated with educational attainment. These included the three SNPs identified in our earlier study (Rietveld *et al.*, 2013). In 2018, the SSGAC published its most recent GWAS of educational attainment in a sample of roughly 1.1 million people (Lee *et al.*, 2018). We found 1,271 SNPs associated with educational attainment, and earlier findings continued to replicate well. All three of these studies involved, at the time they were conducted, the largest sample sizes ever studied for genetic associations with a social-science outcome.

Researchers don't yet know *why* these SNPs are associated with differences in educational attainment. Their predictive power may derive from many different types of mechanisms, some of which would be quite indirect. For example, genetic variation may affect neural functions such as memory. Genetic variation may improve sleep quality (making it easier to subsequently stay awake in boring lectures). Genetic variation can affect personality traits, such as the willingness to listen politely to and follow the instructions of teachers (who aren't always right but nevertheless dictate grades and other outcomes). There may also be even more convoluted pathways. For example, genetic variation can affect one's sociability, which might draw someone into or drive someone out of the particular social environments that exist in higher education.

There were three key takeaways from the SSGAC's prior work:

(1) A GWAS approach can identify specific SNPs statistically associated with socio-behavioral outcomes if the study is conducted in large enough samples (at least 100,000 people).

(2) SNPs that are associated with a socio-behavioral outcome such as educational attainment are each likely to have less predictive power than are SNPs that are associated with a biomedical or other physical outcome (Chabris *et al.*, 2015). For example, of the hundreds of SNPs found to be associated with height to date (Wood *et al.*, 2014; Yengo, Sidorenko, *et al.*, 2018), the SNP with the strongest association predicts 0.4% of the variation across individuals in height, whereas the SNP with the strongest association with educational attainment identified to date predicts less than one tenth (<0.04%) as much of the variation in educational attainment (Lee *et al.*, 2018). (The SNPs that have not yet been identified will very likely explain even *less* variance than those that are currently known, since statistical power is greatest for those that explain the most variance; in other words, the largest effect-size SNPs are likely to have been the first ones to have been identified in earlier GWASs.)

(3) In the samples studied, at least 20% of the variation in educational attainment can in principle be predicted by genetic differences (Rietveld *et al.*, 2013), implying that the genetic associations with educational attainment result from the cumulative effects of at least thousands (and probably millions) of SNPs, not just a few.

These findings from twin, family, and GWASs imply that individuals who carry an allele associated with greater educational attainment will on average complete slightly more formal education than other (similarly environmentally situated) individuals who carry a different allele of the same SNP. Put in population terms, these findings imply that people with particular alleles will tend *on average* to complete more formal education, while people who carry other alleles will tend *on average* to complete less formal education. It is important to emphasize that these associations represent *average tendencies* in a population. Women are, on average, shorter than men. But you likely know many tall women and many short men.

Similarly, many individuals with high polygenic indexes for educational attainment will not get a college degree, and vice-versa (see FAQ 3.4). It is also important to recall (from FAQ 1.5) that these average tendencies of alleles on educational outcomes may reflect indirect genetic influences on education that operate through environmental channels, such that a polygenic index that is moderately predictive in one environment may become less predictive or not at all predictive in a very different environment. Polygenic indexes for educational attainment are poor predictors of individual outcomes and sensitive to environments, but increasingly useful tools in social science research (see FAQ 2.4).

2 Study design and results

2.1 What did you do in this paper? How was the study designed? Why was the study designed in this way?

We conducted a GWAS (see FAQ 1.3) of educational attainment (see FAQ 1.2) in a sample of over 3 million people. The sample size we used in the current study is much larger than that used in previous GWAS of educational attainment (see FAQ 1.8). By constructing a sample of over 3 million, we expected to estimate genetic effects with much greater accuracy than previous studies (with smaller samples). As a result, we expected to identify many more specific SNPs that are associated with educational attainment and to build a more accurate polygenic index.

To construct such a large sample, we started with the data analyzed in our most recent paper (Lee *et al.*, 2018): a GWAS of roughly 300,000 research participants from 69 datasets; a GWAS of roughly 440,000 research participants from the UK Biobank, a large-scale biomedical database and research resource; and a GWAS of roughly 365,000 research participants from the personal genomics company 23andMe. We then replaced the earlier 23andMe sample with an updated GWAS based on roughly 2.3 million 23andMe research participants. This new data increased the combined sample size from about 1.1 million participants to about 3 million participants. All of these datasets have surveyed and genotyped their research participants.

Our study was limited to only the most common type of genetic difference: SNPs (see FAQ 1.3). Like our most recent previous study (Lee *et al.*, 2018) but unlike most other studies, which have analyzed only the autosomes (the non-sex chromosomes), our study also included SNPs on the X chromosome (see FAQ 2.9). Also unlike most other studies (including our own previous work), which have studied only the additive (i.e., linear) effects of SNPs, our study also studied their dominance (i.e., non-linear) effects (see FAQ 2.10). In total, our analyses included approximately 10 million SNPs.

As in other GWASs, our analyses included only individuals of primarily European genetic ancestries. (We say *genetic* ancestries because we are not talking about who someone identifies as their ancestors but, rather, the similarity between someone's genome and the genome of a "reference sample" for a population from prior genetic studies. And throughout these FAQs we refer to European and African genetic *ancestries*, plural, because there is tremendous genetic diversity within each continent, especially Africa.) Such individuals are identified in different ways in different cohorts that participated in our study (depending on, for example, the demographic composition of the country where the individuals live). In all cohorts, though,

statistical summaries of the allele frequencies and allele correlation patterns in people's genomes (see FAQ 2.2), called principal components, are used as part of the procedure. In particular, individuals are only identified as having European genetic ancestries if their principal components are sufficiently similar to those of reference individuals recruited in prior genetic studies, whose ancestors over several generations were all born in a European country. The restriction to European genetic ancestries is needed in order to reduce statistical confounds that otherwise arise from studying populations that include people with different genetic ancestries (see the discussion of population stratification bias in FAQ 2.2; see also FAQs 1.4, 2.7 & 3.5).

In the remainder of the paper, we used the findings from the GWAS for a range of additional analyses that explored (among other things):

- the predictive power of the polygenic index for educational attainment (FAQ 2.4), as well as cognitive performance and high school academic achievement;
- why the polygenic index is less predictive in individuals of African genetic ancestries than in individuals of European genetic ancestries (FAQ 2.5);
- the predictive power of the polygenic index for the risk of various diseases (FAQ 2.6);
- the extent to which the polygenic index's predictive power for educational attainment and other outcomes is due to its correlation with environmental factors rather than to genes, per se (see FAQ 2.7);
- assortative mating based on educational attainment (see FAQ 2.8);
- the effects of SNPs on the X chromosome on educational attainment (FAQ 2.9); and
- the magnitude of "dominance effects" for the effects of SNPs on educational attainment (see FAQ 2.10).

2.2 What are common pitfalls in GWASs? What precautions did you take against them?

There are many potential pitfalls that can lead to spurious results in genome-wide association studies (GWASs). We took many precautions to guard against these pitfalls.

One potential source of spurious results is incomplete "quality control" (QC) of the genetic data. To avoid this problem, we use QC protocols from medical genetics research (Winkler *et al.*, 2014). We supplement these protocols by developing and applying additional, more stringent QC filters.

Another potential source of spurious results is a confound known as "population stratification bias." (We discuss a well-known illustration of this confound—a hypothetical GWAS of chopstick use—in FAQ 1.3.) In our study we correct for population stratification bias as much as possible. At the outset, we restrict the study to individuals of European genetic ancestries. As is standard in GWASs, we also control for "principal components" of the genetic data in the analysis; these principal components capture the small genetic differences across genetic ancestry groups within European populations, so controlling for them largely removes the spurious associations arising solely from these small differences.

After taking these steps to minimize bias stemming from population stratification, we conduct a standard analysis to assess how much population stratification bias still remains in our data after our efforts to minimize it, called LD Score regression (Bulik-Sullivan *et al.*, 2015). The results of this analysis indicate that the biases in our results due to population stratification are small.

The "direct effect" of the polygenic index from our within-family analysis, described in FAQ 2.7, is immune to any remaining population stratification bias. Population stratification bias can only arise when individuals are from different families with different genetic ancestries. By controlling for the polygenic indexes of an individual's parents, we are also controlling for any differences in genetic ancestry across individuals.

2.3 What did you find in the main GWAS of educational attainment?

In our sample of roughly 3 million people, we found 3,952 SNPs that were associated with educational attainment (using the standard statistical threshold in GWAS, which adjusts for multiple hypothesis testing). This is a substantial increase from the 1,271 SNPs identified in our last GWAS of around 1 million individuals (Lee *et al.*, 2018), further confirming the importance of large sample size for identifying SNPs associated with socio-behavioral outcomes.

The current study further confirmed the finding from our earlier work that the effects of *individual* SNPs on educational attainment are each extremely small. The median effect size across the 3,952 SNPs was just 1.4 weeks of schooling per allele; even the SNPs with the strongest associations only predicted around 3.5 weeks of additional schooling per allele. Taken together, these 3,952 SNPs accounted for roughly 8% of the variation across individuals in years of education completed.

Here is another way to think about this result. We could use the results for these 3,952 SNPs (not the ~1 million SNPs across entire genome that we discuss in FAQ 2.4) to predict the educational attainment for a new group of people (separate from our discovery sample) whose educational attainment is unknown to us. We could then compare each individual's *predicted* educational attainment to their *actual* educational attainment. If we did so, our results would show that if someone were predicted to complete an above average number of years of schooling (i.e., to be in the top half of educational attainment), that person would have about a 59% chance of actually being in the top half of educational attainment. 59% is better than the 50% odds of making a correct prediction that you would have if you used a coin flip to predict whether someone is in the top or bottom half of educational attainment—but only a bit better. By contrast, a prediction based on a polygenic index that combines the complete set of ~1 million SNPs that we studied (see FAQs 1.6 & 2.4) has more predictive power: about 13% of the variation across individuals. (Even this amount of predictive power still corresponds to having only a 62% chance of correctly guessing whether someone is in the top or bottom half of educational attainment.)

The contrast between the 8% of the variation predicted by the 3,952 SNPs and the 20% estimated to be explained by common SNPs (see FAQ 1.8) implies that there are many other SNPs that have not yet been identified. Even larger sample sizes will be needed to identify them.

It is also important to keep in mind that educational attainment is a complex outcome, and our study focuses on only a tiny piece of the bigger picture. In this paper, we only examine one type of genetic difference (SNPs). Other genetic effects, environmental effects, and their interactions are important topics of active research and of future work by the SSGAC. Such work includes studies of associations between educational attainment and epigenetic marks, i.e., other molecules that attach to a person's DNA over the course of their lifetime and tell their genes to switch "on" or "off" (Linnér *et al.*, 2017).

2.4 How predictive is the polygenic index developed in this study?

As discussed in FAQ 1.6, we can create a polygenic index using the GWAS results from around ~1 million SNPs. The polygenic index we construct "predicts" (see FAQ 1.5) around 13% of the variation in education across individuals of European genetic ancestries (when tested in independent data that was not included in the GWAS). This ~1 million SNP polygenic index predicts much more of the variation than does the genetic predictor described in FAQ 2.2, which was based on only 3,952 SNPs. Including all ~1 million SNPs tends to add predictive power because the threshold for significance/inclusion that is used to identify the 3,952 SNPs is very conservative (i.e., many of the other ~1 million SNPs are also associated with educational attainment but are not identified by our study, and on net, it turns out empirically that more signal than noise is added by including them). This study's polygenic index has much more predictive power than polygenic indexes constructed from our earlier three GWASs of educational attainment, because all of those studies had much smaller sample sizes (~100,000, ~300,000, and ~1.1 million individuals, respectively, compared with ~3 million individuals in the current study).

Individuals with high polygenic indexes have, *on average*, higher levels of education than those with lower polygenic indexes. In the present study, we found that among the individuals of European genetic ancestries from a U.S. sample of young adults (the National Longitudinal Study of Adolescent to Adult Health), 7% of those with the lowest 10% of polygenic indexes graduated from college, compared with 71% of those with the highest 10% of polygenic indexes. These results show both that polygenic indexes have some predictive power but also that polygenic indexes do not at all pin down individual outcomes: even when polygenic indexes are based on a GWAS of many more people and therefore have even greater predictive power than ours, there will always be many people whose polygenic indexes "predict" lower educational attainment who in fact attain relatively high amounts of education and vice-versa.

As we discuss further in FAQ 3.4, an individual's polygenic index for education (even a polygenic index based on ~1 million SNPs) is still *not* a very accurate prediction of that individual's actual level of education attained. We emphasize that point using Figure 2c in the paper, reproduced here:

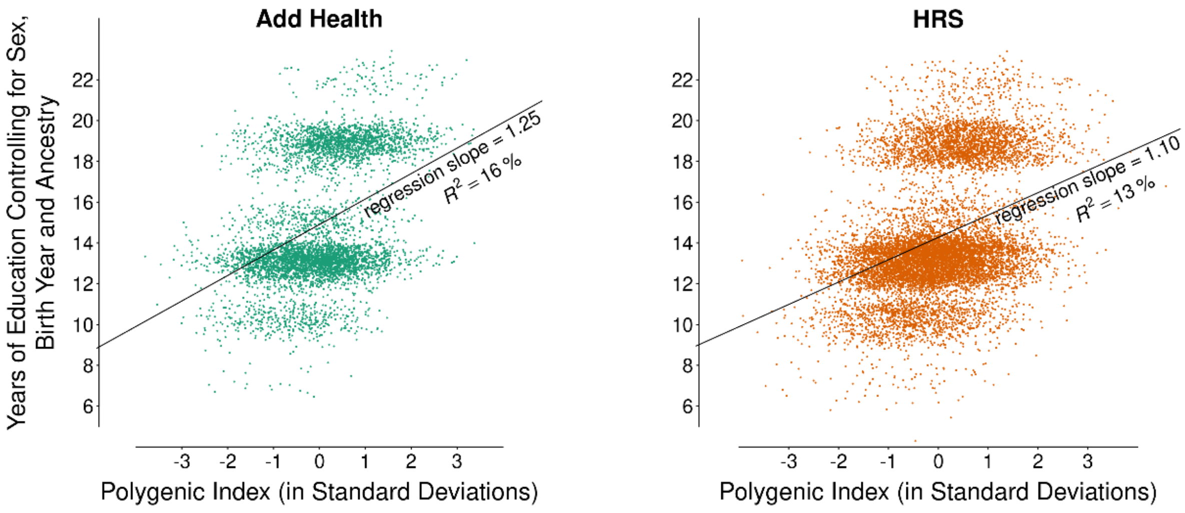

In the figure on the left, each point is an individual of European genetic ancestries from the U.S. sample of younger adults mentioned above (the National Longitudinal Study of Adolescent to Adult Health). In the figure on the right, each point is an individual of European genetic ancestries from a U.S. sample of older adults (the Health and Retirement Study). In both figures, the *x*-axis is the individual's polygenic index, and the *y*-axis is the individual's actual number of years of formal schooling (after converting the level of education to an internationally standardized scale and adjusting for age and sex). The points are jittered slightly from their actual values in order to ensure that points do not lie directly on top of each other. While the figures show that there is a relationship between the polygenic index and the actual amount of an individual's education, they also show that people with the same polygenic index value—points with the same *x*-axis value—vary a great deal in how much education they have. For instance, in both samples, among those with a polygenic index that is one standard deviation below average, individuals range in their actual educational attainment from about 7 years of formal education to about 22 years.

Despite the fact that polygenic indexes are not useful for predicting a particular individual's educational attainment, they are useful for *scientific studies* (including social science, health research, etc.). Such studies are concerned with aggregate population trends and averages rather than with individual outcomes. In particular, because the polygenic index predicts about 13% of the variation across individuals, studies of its association with other variables can be well powered in sample sizes as small as 61 individuals (but not as small as 1 individual!).

Through this lens, the fact that the current study's polygenic index for educational attainment predicts 13% of the variation across individuals in education attained is quite meaningful and rivals the predictive power of other variables commonly used in research—none of which, taken alone, predicts a large amount of variation in a socio-behavioral outcome. For example, in our prior work (Lee *et al.*, 2018) we estimated that household income predicts ~7% of variation in educational attainment and mother's education predicts ~15%. Thus, our index has approached the predictive power of important demographic variables and can

be used in similar ways (e.g., to control for genetics as an additional confound when evaluating the effects of environmental differences or interventions).

With a relatively high level of population-level predictive power, the polygenic index we constructed enables other research that is of value to social scientists and health researchers. Such studies are already being conducted with the (less powerful) polygenic indexes from our earlier GWASs of educational attainment (see FAQ 1.7). Our new results will enable many additional applications, such as studies that use the polygenic index in relatively small samples that contain rich health and socio-behavioral data that is expensive to collect (e.g., a randomized controlled trial that studies the effects of subsidizing higher education and uses the polygenic index as a control variable).

A major caveat to all of this is that polygenic indexes developed from GWASs of particular genetic ancestry populations are known to be less predictive when applied to people of any other genetic ancestry (for reasons we discuss in FAQ 2.5). (As noted above in FAQ 2.1, we say *genetic* ancestries because we are not talking about who someone identifies as their ancestors but, rather, the similarity between someone's genome and the genome of a "reference sample" for a population from prior genetic studies. And throughout these FAQs we refer to European and African genetic *ancestries*, plural, because there is tremendous genetic diversity within each continent, especially Africa.) For example, studying polygenic indexes for various health outcomes derived from GWAS participants of European genetic ancestries, Martin *et al.*, (2017) and Duncan *et al.* (2019) found that, on average across the polygenic indexes, the predictive power for the outcomes was roughly 20-30% as large in samples of African genetic ancestries as it was in samples of European genetic ancestries.

Our educational attainment polygenic index, like most other polygenic indexes, was developed with participants of European genetic ancestries (because currently most genotyped people are of European genetic ancestries, and very large numbers of people are needed to create meaningful polygenic indexes). We illustrate and quantify the attenuation in predictive power for individuals of African genetic ancestries, populations for which previous work has found that the attenuation is especially large. Specifically, we examined the predictive power of our educational attainment polygenic index in the samples of African genetic ancestries of our two prediction datasets, HRS and Add Health. The polygenic score explained 12.0% and 15.8% of the variance among the participants of European genetic ancestries in the HRS and in Add Health, respectively. By contrast, the polygenic index explained far less in the participants of African genetic ancestries: 1.3% and 2.3%, respectively. Thus, when applied to those of African genetic ancestries, the polygenic index has only 10-15% of the predictive power in has with those of European genetic ancestries. (In our previous GWAS of educational attainment, we also tested the predictive power of the polygenic index in a sample of HRS participants with African genetic ancestries (who may or may not have additional genetic ancestries). We similarly found that the earlier polygenic index had predictive power only 11% as large as the predictive power in the European genetic ancestries sample.) Thus, our results suggest that the drop-off in predictive power for the polygenic index for educational attainment is especially large, relative to polygenic indexes for other outcomes.

Unfortunately, this attenuation of predictive power means that for most populations, many of the benefits of a polygenic index will be postponed until large GWAS studies are conducted using samples from these populations. Currently, most large, genotyped samples are of European genetic ancestries. We prioritize

GWASs of samples of other genetic ancestries, but cannot implement this analysis until large enough samples of these populations have been genotyped and are made available to the research community.

2.5 Why is the polygenic index less predictive in samples of African genetic ancestries than in samples of European genetic ancestries?

As noted above in FAQ 2.4, we expect attenuated predictive power when applying a polygenic index developed with participants from *any* particular genetic ancestry populations to people of any other genetic ancestry, and we illustrated this attenuation in samples of participants of African genetic ancestries. We conducted additional analysis in order to shed some light on *why* the polygenic index is less predictive in participants of African genetic ancestries than in samples of European genetic ancestries. We study the main potential reasons, which fall into two categories.

The first category is primarily about environmental factors. In this category, there are two main explanations of the reduced predictive power in samples of African genetic ancestries. First, genetic factors as a whole might simply matter relatively less for predicting educational attainment in samples of African genetic ancestries because environmental factors matter relatively more. In that case, the polygenic index—which captures some of these genetic factors—would similarly predict less well in the samples of African genetic ancestries. Second, there are gene-environment interactions (see FAQ 3.2). Since the samples of African genetic ancestries face different environments on average than do the samples of European genetic ancestries—for instance, racist expectations for classroom performance, poorer access to educational resources, and other average socio-economic circumstances that can affect the ability to succeed or remain in school—the associations between SNPs and educational attainment could be different in those of African genetic ancestries than in those of European genetic ancestries. If so, the SNP weights that produce a predictive polygenic index in populations of European genetic ancestries will turn out to be suboptimal weights for prediction in African-genetic-ancestry populations.

The second category can be thought of as purely genetic reasons. In this category, there are also two main explanations of the reduced predictive power in samples of African genetic ancestries (to use our example). First, purely by chance, particular alleles are more or less common in populations with different genetic ancestries. Much of the predictive power of the polygenic index comes from the (positive or negative) weights it puts on alleles that are relatively common in populations of European genetic ancestries. Because many of these alleles are not as common in populations of African genetic ancestries, the polygenic index will have less predictive power in those populations. Second, populations also differ from each other in their linkage disequilibrium (LD) patterns, i.e., their correlation structure across SNPs (see FAQs 2.2 & 3.5). A given SNP may be associated with educational attainment because the SNP is in LD (i.e., correlated) with a SNP elsewhere in the genome that causally affects education (see FAQ 1.6). If the strength of the correlation is greater in one genetic ancestry group than in another, then the size of the association will be larger in that genetic ancestry group. The fact that there are differences across genetic ancestry groups in the set of associated SNPs and their effect sizes means that the weights for constructing polygenic indexes in individuals of European genetic ancestries (FAQ 1.4) would be the "wrong" weights for individuals of other genetic ancestries.

The first category of explanations is difficult for us to directly assess given the data we have, but in our paper, we directly evaluated the second category of explanations and used those results to indirectly assess the first category. Geneticists have conducted in-depth studies of the genetic differences across genetic ancestry groups in allele frequencies and LD. This makes it possible for us to assess how much the polygenic index's predictive power would be expected to be reduced in samples of African genetic ancestries based on these factors alone. The paper that developed the methodology for this analysis applied it in the UK Biobank dataset and studied height, BMI, HDL and LDL cholesterol, triglycerides, asthma, type 2 diabetes, and hypertension (Wang *et al.*, 2020). We also used the UK Biobank in order to enable us to compare educational attainment to these other phenotypes.

We find that, based on the second category of explanations, we would expect the predictive power for educational attainment to be 35% as large in samples of African genetic ancestries than in samples of European genetic ancestries. This is *much* larger than the 10-15% we actually find in our U.S.-based samples (see FAQ 2.4). We conclude that the first category of explanations—the environmental factors—is therefore likely to be important. Moreover, the discrepancy between the actual predictive power in samples of African genetic ancestries and the predictive power expected based on the second category of explanations is larger for educational attainment than for the phenotypes studied by Wang *et al.* (2020), suggesting that the environmental factors are more important for educational attainment.

For a number of reasons, this analysis of ours is only suggestive. One reason is that the UK Biobank sample of African genetic ancestries likely includes a higher fraction of immigrants to the UK than does the sample of European genetic ancestries, and individuals who completed some or all of their schooling outside the UK education system are less comparable. However, we believe our analysis points toward one direction for future work to understand why the polygenic index is less predictive in people of different genetic ancestries.

2.6 What did you find in the analysis of disease risk?

In addition to studying how accurately the polygenic index predicts educational attainment, we also examined how accurately it could predict some common diseases. Prior work, including our own, has found that the SNPs that predict educational attainment overlap with those that predict health outcomes, including Alzheimer's disease, bipolar disorder, ADHD, schizophrenia, coronary artery disease, and longevity (Okbay, Beauchamp, *et al.*, 2016; Pickrell *et al.*, 2016; Riccardo E Marioni *et al.*, 2016; Warrier *et al.*, 2016; Anderson *et al.*, 2017; Tillmann *et al.*, 2017). However, the polygenic index for educational attainment has not been used as a predictor of such outcomes.

We studied ten common diseases, including asthma, arthritis, migraine, depression, and several related to heart disease (such as Type 2 diabetes and heart attack). We chose diseases that themselves have been the focus of large-scale GWASs. Thus, we could compare the predictive accuracy of our polygenic index for educational attainment with disease-specific polygenic indexes from those GWASs. For these analyses, we used a sample of roughly 440,000 individuals from the UK Biobank (the number of individuals with each disease varied depending on the disease).

The main result from these analyses is that, on average across the diseases, predicting disease risk using *both* the polygenic index for educational attainment *and* the disease-specific polygenic index increases predictive accuracy by roughly 50%, relative to using only the disease-specific polygenic index. On average, a disease-specific polygenic index predicts roughly 1.2% of the variation across individuals, whereas a disease-specific polygenic index together with the polygenic index for educational attainment jointly predict roughly 1.8% of the variation. This finding points to the potential value of the polygenic index for educational attainment for medical and epidemiological research. However, we highlight that the actual amounts of predictive power are small, much smaller than the roughly 13% for predicting educational attainment itself (see FAQ 2.4). We also note that genes are estimated to have a stronger influence (relative to environmental influences) on many complex diseases than they do on educational attainment; the primary reason that the educational attainment polygenic index has much greater predictive power than these disease polygenic indexes is that the GWASs that created the disease polygenic indexes are to date much smaller than our educational attainment GWAS.

2.7 What did you find in the family-based analyses?

Our family-based analyses involve looking at how predictive the polygenic index for educational attainment is once we control for the educational attainment polygenic indexes of the individual's parents. Doing this allows us to better understand some of the sources of the predictive power of the polygenic index. There are three categories of sources of predictive power, listed here along with conventional names for these sources used in the literature:

- Direct genetic effects: Some SNPs (that are either included in the polygenic index or correlated with SNPs that are included) may have an effect on characteristics of an individual, such as cognitive skills and personality, that in turn may influence educational attainment. These effects may be mediated by environmental factors (e.g., a child who likes reading will be more likely to pursue that interest in school if the child lives in a society where reading is valued in school).

- Gene-environment correlation: The polygenic index is correlated with environmental factors that affect educational attainment. For example, a person's polygenic index is correlated with the polygenic indexes of that person's biological parents. Rearing parents' polygenic indexes affect the environment in which the person grows up. For example, if the parents are more educated, they are likely to earn higher incomes and live in a neighborhood with well-funded schools (where local funding matters), which may provide educational advantages to their child. (Another source of gene-environment correlation is "population stratification," in which certain genetic variants are more common in certain genetic ancestries, e.g., English versus Scottish. This can generate "population stratification bias" if having those genetic ancestries is also associated with cultural influences that affect educational attainment. However, population stratification bias should be largely reduced by the "quality control" procedures of our GWAS; see FAQ 2.2.)

- Assortative mating: Having a higher polygenic index is correlated with having other SNPs that are also associated with greater educational attainment. Assortative mating on educational attainment refers to the fact that, on average, there is a tendency for people to marry and have children with other people who have a similar amount of education (see FAQ 2.8). Consequently, people who

inherit SNPs associated with higher educational attainment from one of their parents are also more likely than average to inherit SNPs associated with higher educational attainment from their other parent. Thus, the SNPs included in the polygenic index are correlated with SNPs not included in the polygenic index in such a way that magnifies the index's predictive power.

The key idea of the family-based analyses is to study the predictive power of the polygenic index *controlling for the polygenic indexes of an individuals' parents*. This allows us to control the second and third sources of a polygenic index's predictive power from the bullet list above and isolate the first, which are commonly referred to as the "direct genetic effects." (This terminology is used to distinguish effects of SNPs on one's own outcome—the "direct effects" of a SNP—from effects on someone else's outcome, which are called "indirect effects." An example of indirect effects is in the second bullet list above: parents' SNPs affecting someone else's—the child's—educational attainment.) When controlling for the polygenic indexes of a person's parents, the association between the person's polygenic index and that person's outcomes captures only direct genetic effects. We therefore call it the "direct effect" of the polygenic index.

While we have noted that the predictive power of the polygenic index as a whole does not necessarily reflect causal effects (see FAQ 1.6)—and indeed, the second and third categories above generate predictive power that is correlational but not causal—the component of the polygenic index's predictive power that is due to "direct effects" *does* reflect the causal effects of some SNPs. Because of that, when we identify how much of the predictive power is due to direct effects, we interpret it as telling us how much is due to causal effects. However, we cannot infer that it is the SNPs included in the polygenic index that have those causal effects; the "direct effects" might be due to correlation between measured non-causal SNPs and unmeasured causal SNPs.

Controlling for the polygenic indexes of a person's parents requires having genetic information on the person's parents. Such data is not available in most of the samples available to us, but we have it or can construct it in some of the samples. The Generation Scotland sample contains data on ~3,500 trios: individuals and both their parents. Two other samples—the UK Biobank and the Swedish Twin Registry (where we use only the fraternal twins)—contain large numbers of siblings, ~53,000 individuals in total. From the sibling data, we use a recently developed method (Young *et al.*, 2020) to impute (i.e., statistically partially reconstruct) parental genetic data. These are the samples we use for our family-based analyses.

In our analyses, we compare the direct effect of the polygenic index (i.e., controlling for the polygenic indexes of an individual's parents) with the "population effect," which is the term we use for the association between the polygenic index and an outcome when we do *not* control for the polygenic indexes of an individual's parents. In contrast to the direct effect of the polygenic index, which captures only the source of predictive power in the first bullet point above, the population effect captures all three sources of predictive power in the bullet list above. Our analyses estimate the ratio of the direct effect of a polygenic index to its population effect. This ratio tells us what fraction of a polygenic index's association with some outcome is due to direct genetic effects.

When the educational attainment polygenic index is used to predict educational attainment itself, we estimate that this ratio is 0.556. That is, we estimate that 56% of the association between the polygenic

index and educational attainment is due to direct genetic effects, and the remainder—44%—is due to the other sources of predictive power representing the second and third bullets above.

Next, we sought to determine this ratio when using the educational attainment polygenic index to predict other outcomes, such as the diseases we analyzed in the paper, including Alzheimer's disease, bipolar disorder, ADHD, schizophrenia, coronary artery disease, and longevity (see FAQ 2.6). However, we cannot study this same set of diseases in our family-based analyses because our trio and sibling samples either do not contain data on the diseases or (in the case of the UK Biobank siblings) do not contain a sufficient number of individuals that have one of the diseases. Instead, to estimate the fraction of the educational attainment polygenic index's association with complex diseases that is due to direct genetic effects, we study a set of 22 health, cognitive, and socioeconomic outcomes. These include several biomarkers related to disease risk, such as BMI, blood pressure, and cholesterol. The set of outcomes also includes height, cognitive performance, smoking, alcohol use, income, and depression. For each of these 22 outcomes, we estimate both the direct effect and the population effect of the polygenic index for educational attainment.

On average across the 22 outcomes, we estimate that the ratio of direct to population effects is 0.588. This is very similar to the ratio when the outcome is educational attainment, and the conclusion is correspondingly similar: we estimate that 59% of the association between the polygenic index and these other outcomes is due to direct genetic effects, and the remaining roughly 41% is due to the other sources of predictive power.

In summary, our family-based analyses find that a substantial part of the predictive power of the polygenic index is due to direct effects, and a substantial part is not. This is true both when using the educational attainment polygenic index to predict educational attainment itself, and when using it to predict other outcomes.

The finding that much of the predictive power is due to direct effects is important for at least three reasons. First, it shows that biases in the GWAS, such as unaccounted-for "population stratification bias" (see FAQs 1.4 & 2.2), are not entirely responsible for the predictive power that we find. This had been shown previously for predicting educational attainment but not for using the educational attainment polygenic index to predict other outcomes.

Second, the finding is a preliminary step toward unpacking the reasons *why* the genetic influences on educational attainment also matter for other outcomes. One possibility is that SNPs influence an outcome that in turn separately influences both education and health. For example, it could be that genetic influences on conscientiousness partially affect both how much education a person gets and also health-promoting behaviors that reduce disease risk. Another possibility is that SNPs influence educational attainment and there is something about formal schooling that, in turn, causes people to engage in more health-promoting behaviors. While our paper does not distinguish between these and other possibilities, our results are informative about the overall strength of the relationship between genetic influences on educational attainment and on certain other outcomes.

Third, the finding that much of the polygenic index's predictive power is *not* due to direct effects—either for educational attainment or for the disease-related biomarkers and outcomes we investigated—is also important. It reinforces the importance of interpreting genetic associations with caution. Our finding implies that a substantial part of the predictive power of the polygenic index is due to some mix of assortative mating and gene-environment correlation. For this and other reasons, we believe it is misleading to use phrases such as "innate ability" or "genetic endowments" to describe what is measured by polygenic indexes based on our GWAS estimates. These phrases incorrectly imply that the polygenic index is entirely capturing direct effects, and they further ignore the potentially important role that environmental factors play in mediating direct effects.

2.8 What did you find in the analysis of assortative mating?

Assortative mating refers to the idea that people tend to have children with people who are similar to themselves in particular ways. Assortative mating is a research topic in the social sciences and also in the field of genomics.

Much prior research has found that there is assortative mating on educational attainment: i.e., the available data reveals a tendency for people to have children with people who have similar educational attainment as themselves (e.g., Mare, 1991). For example, in the UK Biobank, we estimate that the correlation between the educational attainments of mates (i.e., biological mothers and fathers) is roughly 0.4. That is a moderately sized correlation. In recent decades in Western countries, educational attainment is one of the outcomes with the strongest assortative mating. In our analysis of assortative mating, we use height as an outcome to compare with educational attainment because it is another outcome for which assortative mating is relatively strong. In the UK Biobank, we estimate that the correlation between mates' heights is roughly 0.3.

In our paper, we study assortative mating using polygenic indexes, which has also been done in some prior research (e.g. Conley *et al.*, 2016; Hugh-Jones *et al.*, 2016; Robinson *et al.*, 2017 and Yengo, Robinson, *et al.*, 2018). In each of the datasets we use, we identify mate pairs based on the genetic data: we find pairs of individuals who share a child in common. We identify 894 mate pairs in the UK Biobank and 2,964 mate pairs in Generation Scotland. Averaging across these data sources, we find that the mate correlation in the polygenic index for educational attainment is roughly 0.17. We again compare with height, this time using a polygenic index for height constructed using the largest published height GWAS that was not based on the datasets we study (Wood *et al.*, 2014). We find that the mate correlation in the polygenic index for height is roughly 0.10.

What is new in our paper is that we use these correlations to test a model of assortative mating that is often assumed in the genetics literature, called the "phenotypic assortment model." When applied to educational attainment, this model states that the mate-pair correlation in the polygenic index for educational attainment is entirely due to the mate-pair correlation in educational attainment. Given the predictive power of the polygenic index, which we estimated (see FAQ 2.4), this model makes a precise prediction about how the mate-pair correlation in an outcome should be related to the mate-pair correlation in the polygenic index for that outcome.

For height, that prediction comes close to what we observe. That is, for height, it does appear that the mate-pair correlation in the polygenic index for height is entirely due to the people tending to marry others with similar height. However, for educational attainment, the prediction of the phenotypic assortment model is far off: the mate-pair correlation in the polygenic index for educational attainment is too high. Thus, for educational attainment, our results provide strong evidence against the phenotypic assortment model. Instead, our results imply that people are marrying other people who are similar to them based on some factor or factors *other than* educational attainment (perhaps in addition to educational attainment itself) but which is correlated with the polygenic index for educational attainment.

We conduct additional analyses to shed light on what these other factors might be. One possible factor is genetic ancestry, which in our data might reflect, for example, people being more likely to marry others from the same city or region of the UK. Another possible factor is cognitive performance. However, we find that assortative mating on both of these factors, added to the effect of assortative mating on educational attainment itself, are not together sufficient to fully account for the mate-pair correlation in the polygenic index for educational attainment. While our results raise the question of what else explains the high mate-pair correlation in the polygenic index, we cannot fully answer the question with the data we have.

In addition to helping us better understand assortative mating, our results also relate to a common theme across several of our analyses (see FAQs 2.4, 2.6 & 2.7): helping us better understand the sources of the polygenic index's predictive power. Specifically, we draw two conclusions about the polygenic index's predictive power from our analysis of assortative mating. First, there are factors besides educational attainment on which people assortatively mate that contribute to the mate-pair correlation in the polygenic index for educational attainment—and these factors in turn likely contribute to the predictive power of the polygenic index for a range of outcomes. Suppose one of these factors is the region where a person grew up. In order to be a factor that contributes to the mate-pair correlation, the region where a person grew up must be associated with the polygenic index. Moreover, the region where a person grew up is likely to be associated with many other things that relate to educational, socioeconomic, and health outcomes, such as quality of local schools, local economic opportunities, air quality, and so on. If the region where a person grew up is correlated with *both* the polygenic index for educational attainment *and* these various outcomes, then it is one component of the gene-environment correlation that helps explain the polygenic index's predictive power (see FAQ 2.4). Thus, the results of our assortative mating analysis provide evidence that there is substantial gene-environment correlation that likely contributes to the polygenic index's predictive power.

Second, if people assortatively mate on factors that are correlated with the educational attainment polygenic index—as our results imply that they do—then this increases the variation of the polygenic index in the population and thereby magnifies its predictive power. To continue the example from above, imagine an extreme scenario of exact assortative mating on where a person grew up. That is, everyone has a mother and a father who are from the same region. In this scenario, there will be more people with very high and very low educational attainment polygenic indexes compared to a scenario where people marry at random across regions. That is because people from regions with high average polygenic indexes are marrying other people from the same region and having offspring that are relatively more likely to also have a high polygenic index. Similarly, people from regions with low polygenic indexes are marrying other people from their same region and having offspring that are relatively more likely to also have a low polygenic index.

Thus, relative to the scenario of people marrying at random across regions, there is more variation of the polygenic index across people in the scenario with assortative mating. Consequently, variation in the polygenic index across people will be associated with (and hence "statistically predict") more of the variation in educational attainment across people.

2.9 What did you find in the analysis of the X chromosome?

Like our most recent GWAS of educational attainment (Lee *et al.*, 2018)—but unlike most GWASs—this study also examined genetic variants on the X chromosome. In addition to the 3,952 variants identified on the autosomes (the non-sex chromosomes), we identified 57 variants associated with educational attainment on the X chromosome.

The results of our analysis of the X chromosome in this study are fully consistent with the results from the previous GWAS of educational attainment (but our confidence in these results is even greater in the current study because of our larger sample size). For example, as in the previous study, we found fewer SNPs associated with educational attainment on the X chromosome than on other chromosomes of similar length. Also as in the previous study, in separate GWASs of men and women, we found that, in aggregate, SNPs on the X chromosome predict similar amounts of variation in educational attainment in men and in women. Some researchers had hypothesized that genetic influences on the X chromosome are an important source of differences in the variance in cognitive performance across men and women. While there were compelling scientific reasons to view such claims skeptically even prior to the publication of our earlier study, the results of both of our studies provide further evidence against the hypothesis.

2.10 What did you do in the "dominance GWAS" of educational attainment? What did you find?

In addition to our standard GWAS of educational attainment on the autosomes (i.e., the non-sex chromosomes) described in FAQ 1.3 above, we also conducted a "dominance GWAS" of educational attainment on the autosomes. As in a standard GWAS, in a dominance GWAS we test each SNP for its association with educational attainment. The only difference is that, unlike in a standard GWAS, in a dominance GWAS, we allow for the possibility that each SNP has a non-linear relationship with educational attainment. A linear relationship is one where an increase in one variable is associated with a correspondingly-sized increase or decrease in another variable. Specifically, suppose (as is typical) there are three possible combinations of alleles at a given SNP: let's call them AA, AB, and BB. And let's assume that the B allele is associated with greater educational attainment. A standard GWAS assumes the "additive model," according to which the effect of going from zero to one B allele (i.e., AA to AB) is assumed to be equal to the effect of going from one to two B alleles (i.e., AB to BB). In contrast, a dominance GWAS separately estimates each of these two effects and thereby allows us to test whether or not they are equal.

(In this context, the term "dominance" originally comes from the idea of "dominant" and "recessive" alleles. In the classical usage, often called "complete dominance," an organism has a trait—for example, a pea is smooth rather than wrinkled—if there are *any* B alleles. In that case, AB and BB would both yield the dominant trait of a smooth pea, and only AA would yield the recessive trait of a wrinkled pea. A dominance GWAS allows for this possibility: a large effect of going from AA to AB but no effect of going from AB to BB. It also allows for other, more common possibilities. For example, in "incomplete dominance," the

effect of going from AA to AB is larger than the effect of going from AB to BB, but both effects are non-zero. Another possibility is "overdominance," where an organism with the AB combination of alleles has more of the trait than an organism with AA or BB.)

To many researchers, it seems natural to expect that relationships between SNPs and outcomes like educational attainment would be non-linear—that is, that there might be less of an effect, or no effect at all, of going from one B allele to two B alleles. Indeed, there is a long tradition in behavior genetics research (much of which compares outcomes across identical and fraternal twins) of assuming that non-linear relationships between SNPs and socio-behavioral outcomes account for a non-trivial fraction of the variation in such outcomes across individuals (e.g. Jinks and Eaves, 1974).

Perhaps surprisingly, there is an equally long tradition in a field of research called quantitative genetics showing that, theoretically, for outcomes like educational attainment that are influenced by many SNPs, deviation from linear relationships are likely to be small and account only for a small fraction of the variation in outcomes across individuals (e.g. Hill, Goddard and Visscher, 2008). There are a variety of theoretical arguments, which stem from both biological and statistical considerations. Based on other kinds of studies that are not dominance GWAS, for many outcomes (but not educational attainment), there is also evidence that dominance deviations account for only a small fraction of the variation across individuals (e.g. Pazokitoroudi *et al.*, 2020; Hivert *et al.*, 2021).

Thus, researchers are divided about whether the relationship between SNPs and socio-behavioral outcomes are likely to be largely linear or to have significant dominance effects. Partly because a dominance GWAS needed to answer that question is more complex than a standard GWAS, ours is one of the first large-scale dominance GWAS conducted for any outcome. We conducted our dominance GWAS in a sample of individuals from 23andMe and the UK Biobank. It was a slightly smaller sample than our standard GWAS, but still a very large sample: ~2.6 million individuals.

Our results strongly support the view of those researchers who believe that with respect to educational attainment, deviations from linear relationships are likely to be small and account only for a small fraction of the variation in outcomes across individuals. We cannot identify *any* SNPs with such a non-linear relationship to educational attainment, despite our very large sample size. While some non-linear relationships between SNPs and educational attainment probably exist, our results indicate that such SNPs must be at least an order of magnitude smaller than the (already very small) linear effects of SNPs on educational attainment.

Even though we cannot identify any dominance effects of specific SNPs, we can use the aggregate results of our dominance GWAS to estimate how much of the variation in educational attainment is explained by the dominance effects that do exist among SNPs in our analysis. Our results suggest that, taken altogether, dominance effects of the SNPs included in our GWAS account for only roughly 0.02% (two hundredths of one percent) of the variation in educational attainment across individuals.

We note two important qualifications about how our findings should be interpreted. First, our results leave open the possibility that there are rare SNPs that have large non-linear relationships with educational

attainment. The data included in our GWAS, as in almost all GWASs, are common SNPs (see FAQ 2.1). These common SNPs capture most of the information about common ways in which people vary genetically (e.g., at least 1% of the population has a different genotype than the remainder of the population). However, these common SNPs do not capture information about rare SNPs (e.g., over 99% of the population has the same genotype, but a small percentage of people differ). Our results are therefore silent about whether these rare SNPs may have substantial non-linear relationships with educational attainment.

Second, although the dominance effects of SNPs included in our GWAS account for only a tiny fraction of the variation across individuals, the combined effect of dominance across many SNPs on a particular individual can nonetheless be substantial. In particular, when two close relatives have offspring, the offspring will have an unusually large number of AA and BB SNPs and an unusually small number of AB SNPs (because the parents are more likely than unrelated individuals to both have the same allele, either A or B). While there is a lot of variation across SNPs and outcomes, on average, having the same two alleles at a SNP, i.e., having AA or BB rather than AB, is known to be harmful to an organism. When an individual's recent genetic ancestors are closely genetically related, there can be a noticeably harmful effect on certain outcomes due to the unusually large number of AA and BB genetic variants. Using our dominance GWAS results, we estimate that the offspring of first cousins will have, on average, roughly 1 fewer month of formal schooling than the offspring of unrelated individuals.

3 Ethical and social implications of the study

3.1 Did you find "the gene for" educational attainment?

No.

We did not find "the gene for" educational attainment or anything else. We identified many SNPs that are associated with educational attainment. Although it was once believed that scientists would discover a few strong associations between genes and outcomes, we have known for a number of years that the vast majority of human outcomes are complex and influenced by many thousands of genes, each of which alone tends to have a small influence on the relevant outcome.

Furthermore, many complex outcomes are also influenced by parts of the genome that are not genes at all but instead serve to regulate genes (e.g., sequences of DNA that influence when a gene is turned on or off). Genes typically contain many SNPs (often dozens or hundreds, and in some cases thousands), and there are even more SNPs outside of genes than inside genes. Complex outcomes are often influenced by millions of SNPs.

3.2 Well, then, did you find "the genes for" educational attainment?

Although we did find many SNPs that are associated with educational attainment, we believe that characterizing these as "genes for educational attainment" is still likely to mislead, for many reasons.

First, most of the variation in people's educational attainment is accounted for by social and other environmental factors, not by additive genetic effects (see FAQ 1.8). "Genes for educational attainment" might be read to imply, incorrectly, that genes are the strongest predictor of variation in educational attainment.

Second, the SNPs that are associated with educational attainment are also associated with many other things (only some of which we identify in this study; see FAQs 2.6. & 2.7). These SNPs are no more "for" educational attainment than for the other outcomes with which they are associated.

Third, the "predictive" power (see FAQ 1.5) of each individual SNP that we identify is very small. Our results show that genetic associations with educational attainment are comprised of thousands, or even millions, of SNPs, each of which has a tiny effect size. Each SNP is therefore weakly associated with, rather than a strong influence on, educational attainment. "Genes for educational attainment" might misleadingly imply the latter.

Fourth, environmental factors can increase or decrease the impact of specific SNPs. Put differently, even if a SNP is associated with higher or lower levels of educational attainment *on average*, it may have a much larger or smaller effect depending on environmental conditions. Indeed, in our most recent previous large-scale GWAS of educational attainment (Lee *et al.*, 2018), we report exploratory analyses that provide evidence of such gene-environment interactions. Educational attainment couldn't even exist as a meaningful object of measurement if we didn't have schools, and having schools introduces societal mechanisms that influence who spends the most years attending them. Accordingly, genetic associations with educational attainment necessarily will be mediated by societal systems and therefore genetic variation should often be expected to interact with environmental factors when it influences social phenomena, such as educational attainment. "Genes for educational attainment" suggests a stability in the relationship between these genes and the outcome of educational attainment that does not exist.

Finally, genes do not affect educational attainment directly (see FAQ 1.4), although we don't know exactly why the SNPs we identify are associated with educational attainment. We found in our most recent previous large-scale GWAS of educational attainment (Lee *et al.*, 2018) that the genes identified as associated with educational attainment tend to be especially active in the brain and involved in neural development and neuron-to-neuron communication. The "predictive" power (see FAQ 1.5) of genes on educational attainment might therefore partly depend on a long process starting with brain development, followed by the emergence of particular psychological outcomes (e.g., cognitive performance and personality). These outcomes might then lead to behavioral tendencies as well as experiences and treatment by parents, peers, and teachers. All of these factors may additionally interact with the environment in which a person lives. Eventually these outcomes, behaviors, and experiences might influence (but not completely determine) educational attainment. Much more research is needed to explore these and other possible explanations for the relationship between SNPs and educational attainment.

3.3 Does this study show that an individual's level of educational attainment (or any other outcome) is determined, or fixed, at conception? Do genes determine the choices we make and who we become?

No and no.

Genes and genetic variation do not determine our choices or who we become. If they did, identical twins would make all of the same decisions, have the same interests, etc. Years of twin studies have shown that, while identical twins tend to be more similar than fraternal twins—including with respect to the years of formal schooling they complete—they are nevertheless different (see FAQ 1.8). This implies that environmental factors also play a large role in our outcomes. In the case of educational attainment, social and other environmental factors account for most variation among people.

But even if it were true that genetic factors accounted for *all* of the differences among individuals in educational attainment, it would *still* not follow that an individual's number of years of formal schooling is "determined" at conception. There are at least three reasons for this.

First, some genetic effects operate through environmental channels (Jencks, 1980). When this is the case, SNPs that are associated with an outcome in one setting might not be associated with it in another setting. As an illustrative example, suppose—hypothetically—that the SNPs we identified are associated with educational attainment because they help students to memorize and, as a result, to become better at taking tests that rely on memorization (in fact, we do not know why the SNPs we identified are associated with educational attainment; see FAQ 3.2). In this example, changes to the intermediate environmental channels—the type of tests administered in schools—could have drastic effects on individuals' educational attainment, even though individuals' DNA would not have changed. A genetic association with educational attainment might not be found *at all* if schools did not use tests that rely on memorization. More generally, the genetic associations that we found might not apply as strongly if the education system were organized differently than it is at present (see also FAQ 1.4).

Second, even if the genetic associations with educational attainment operated entirely through non-environmental mechanisms that are difficult to modify (such as direct influences on the formation of neurons in the brain and the biochemical interactions among them), there could still exist powerful environmental interventions that could cancel out what would have been the effect of SNPs. Consider a famous example suggested by the economist Arthur Goldberger. Genes influence eyesight at least partly through biological mechanisms that themselves are hard to change. Yet even if *all* variation in unaided eyesight were due to genes, there would still be enormous benefits from introducing eyeglasses, which can erase the contribution of genes to that outcome (Goldberger, 1979). Conversely, someone genetically predisposed towards being taller than average might end up being shorter than average if they lacked adequate nutrition during childhood. In the context of educational attainment, policies such as a required minimum number of years of education and dedicated resources for individuals with learning disabilities can increase educational attainment in the entire population and/or reduce differences among individuals—all without, of course, changing anyone's DNA.

Third, even if the genetic effects on educational attainment were not influenced by changes in the environment, those environmental changes themselves could still have a major impact on the educational attainment of the population as a whole. For example, if young children were given more nutritious diets, then everyone's school performance might improve, and college graduation rates might increase. By analogy, 80%-90% of the variation across individuals in height is due to genetic factors. Yet the current generation of people is much taller than past generations due to changes in the environment such as improved nutrition.

3.4 Can the polygenic index from this paper be used to accurately predict a particular person's educational attainment?

No. While the "predictive" power (see FAQ 1.5) of our polygenic index is substantial—it predicts 13% of variation in educational attainment across individuals with European genetic ancestries—and useful for some purposes (see FAQ 1.7), it is important to keep in mind that the score *fails to predict* the vast majority (87%) of variation in years of education across individuals. Many of those with low polygenic indexes go on to achieve high levels of education, and a large proportion of those with high polygenic indexes do not complete college.

Thus, an important message of this paper and our earlier papers is that DNA does *not* "determine" an individual's level of education, for multiple reasons: First, it is estimated that, at least in the environments in which we have been measuring it, the additive effects of common SNPs will only ever predict about 20% of the variance in educational attainment across individuals. Second, *today's* polygenic index is only able to predict about two-thirds of that 20% (i.e., 13 percentage points). Third, since SNPs matter more or less depending on environmental context (see FAQ 2.7), a polygenic index might be less (or more) predictive for individuals in some environments than for individuals in others. Fourth, polygenic indexes are most predictive when the individuals used to make the index have the same genetic ancestries as the people whose outcomes you would like to predict. For example, because the research in this paper is almost entirely based on individuals with European genetic ancestries, the polygenic index predicts only about 2% of the variance in individuals with African genetic ancestries (see FAQ 3.5). Finally, polygenic predictions only hold for as long as the environment in which they were developed remains substantially the same: if the laws or pedagogy underlying a population's educational system changes substantially, then so, too, might the optimized polygenic index. Just as eyeglasses allow those genetically predisposed to poor vision to have nearly perfect vision, innovations in education (say, an innovation that makes education irresistibly engaging, thus mitigating the risk to those with SNPs associated with lower propensity to pay attention or avoid distraction) might result in those with lower polygenic indexes to actually achieve just as much education, on average, as those with higher polygenic indexes (see also FAQs 3.2 and 3.3).

As sample sizes for GWASs continue to grow, it will likely be possible to construct a polygenic index for educational attainment whose predictive power comes closer to 20% of the variance in educational attainment across individuals (Rietveld *et al.*, 2013). Even this level of predictive power would pale in comparison to some other scientific predictors. For example, professional weather forecasts correctly predict about 95% of the variation in day-to-day temperatures.

The results of SSGAC studies have sometimes been used by online platforms, including some companies, to predict individual outcomes. We recognize that returning individual genomic "results" can be a fun way to engage people in research and other projects and to feed or stoke their interest in genomics. But it is important that participants/users understand that these individual results are not *meaningful* predictions and should be regarded essentially as entertainment. Failure to make this point clear risks sowing confusion and undermining trust in genetics research.

3.5 Can your polygenic index be used for research studies in diverse genetic ancestry populations?

Only in a limited way. As a practical matter, it is possible to calculate a polygenic index for any individual for whom genome-wide data is available, but the polygenic index will be most "predictive" (see FAQ 1.5) in populations of European genetic ancestries.

Our study was conducted only using samples of individuals of European genetic ancestries (see Appendix 1). The set of SNPs that are associated with educational attainment in people of European genetic ancestries is unlikely to overlap perfectly with the set of SNPs associated with educational attainment in people of other genetic ancestries. And even if a given SNP is associated in both genetic ancestry groups, the effect size—in other words, the strength of the association—will likely differ. This is partly because linkage disequilibrium (LD) patterns (i.e., the correlation structure of the genome) vary by genetic ancestry. This means that some variant may be associated with educational attainment because the variant is in LD (i.e., correlated) with a variant elsewhere in the genome that causally affects education (see FAQ 1.4). If the strength of the correlation is greater in one genetic ancestry group than in another, then the size of the association will be larger in that genetic ancestry group. Moreover, even if LD patterns were similar in each genetic ancestry group, the association may differ in different groups because environmental conditions differ (see FAQ 1.4, 3.2 & 3.3). The fact that there are differences across genetic ancestry groups in the set of associated SNPs and their effect sizes has two important implications.

First, it means that *polygenic indexes of individuals from different genetic ancestry groups* cannot *be meaningfully compared*. A recent paper (Martin *et al.*, 2017) illustrated this point in the context of polygenic indexes for predicting height; in the sample analyzed in that paper, polygenic indexes for height predict that individuals of European genetic ancestries would be taller than those of South Asian genetic ancestries, who in turn would be taller than those of African genetic ancestries. In actuality, however, populations of African genetic ancestries represented by the sample have similar height to populations of European genetic ancestries, and populations of both African and European genetic ancestries tend to be taller than populations of South Asian genetic ancestries.

Second, while polygenic indexes can be used to predict differences across individuals *within* a sample of people of non-European genetic ancestries, *the amount of predictive power will be much smaller than in a sample of people of European genetic ancestries*. Such an attenuation of predictive power has been repeatedly found in prior work (Belsky *et al.*, 2013; Domingue *et al.*, 2015, 2017; Vassos *et al.*, 2017). Unfortunately, this attenuation means that for non-European genetic-ancestry populations, many of the benefits of having a polygenic index available will have to wait until large GWASs are conducted using

samples from these populations. (Currently, most large genotyped samples are of European genetic ancestries.)

For a more extensive, excellent discussion of these and related issues, see Graham Coop's blog post "Polygenic scores and tea drinking": https://gcbias.org/2018/03/14/polygenic-scores-and-tea-drinking/.

For more on population stratification bias, see FAQs 1.4 & 2.2.

3.6 Should practitioners (e.g., in education or other domains) use the results of this study to make decisions?

No. Doing so would be extremely premature and unsupported by the science. As explained in FAQ 3.4, our polygenic index is only *weakly* "predictive" (see FAQ 1.5) of educational and health outcomes for individuals. Guessing whether a person has above- or below-average years of education using their polygenic index would only be slightly better than a coin flip: that will be unacceptable in most practice contexts. Nor can our results immediately be used to develop an intervention (say, to improve graduation rates by changing pedagogy) because we don't know *why* the SNPs we identified are associated with educational attainment; much more research is needed to investigate that before any such interventions would be evidence-based.

In this respect, our study is no different from GWASs of complex medical outcomes. There, as here, GWAS associations alone are not actionable for decisions being made by practitioners. They are only an important first step in basic science research that might someday be useful in helping practitioners make decisions. GWAS can help identify SNPs associated with an outcome of interest. Subsequent studies of those SNPs would then be needed to confirm their relationship to the outcome.

When the outcome in question is socio-behavioral rather than clinical, there are additional questions about whether polygenic indexes might stigmatize, whether there are sufficient legal and other protections to prevent discrimination on the basis of polygenic indexes, and whether the expected benefits of using polygenic indexes in a particular practice setting would justify these risks. Addressing these questions would involve a great deal of multidisciplinary empirical and normative research.

Although the results of our study are not immediately useful in practice, they are useful to social scientists (e.g., by allowing them to construct polygenic indexes that can be used as control variables in randomized controlled trials or in studies of gene-by-environment interactions, see FAQ 1.7).

3.7 Could this kind of research lead to discrimination against, or stigmatization of, people with the relevant genetic variants? What has been done to help avert the potential harms of this research?

Unfortunately, like much research, the results of our study and of research that builds on it could be misunderstood, misapplied in ways that are inconsistent with the science, and applied in ways that are unethical.

Genetics research in particular has a long history of being used to harm people, especially on the basis of racist and classist inferences. Indeed, the term "eugenics" was coined in the late 1800s by one of the most prominent early researchers of heredity, Francis Galton. In the first half of the 20th century, many prominent scientists, politicians, clergymen, and other influential individuals across the political spectrum were active proponents of the belief that socioeconomic disparities in society were primarily or exclusively caused by genetic factors and that existing social disparities simply reflected the natural order and were both inevitable and justified. These ideas, and their active development and endorsement by many in the scientific community, laid the groundwork for 20th century forced sterilizations, anti-miscegenation laws, eugenics-based immigration restrictions, and genocide. Today, racist individuals and groups continue to misinterpret and misuse the results of genetics research to give unjustified support for their agenda.

Acknowledging the harm done to certain groups in the name of science reminds us of the importance of careful communication of the implications of scientific research and the need for intense vigilance to ensure that disadvantaged groups are not further harmed by this and related work. Nevertheless, for a variety of reasons, in this instance, we do not think that the best response to the possibility that useful knowledge might be misused is to refrain from producing the knowledge. Here, we briefly discuss some of the broad potential benefits of this research. We then describe what we take to be our ethical obligation as researchers conducting this work.

First, one benefit of conducting social-science genetics research in ever larger samples is that doing so allows us to correct the scientific record. An important theme in our earlier work has been to point out that most existing studies in social-science genetics that report genetic associations with behavioral outcomes have serious methodological limitations, fail to replicate, and are likely to be false-positive findings (Benjamin *et al.*, 2012; Chabris *et al.*, 2012, 2015). This same point was made in an editorial in *Behavior Genetics* (the leading journal for the genetics of behavioral outcomes), which stated that "it now seems likely that many of the published [behavior genetics] findings of the last decade are wrong or misleading and have not contributed to real advances in knowledge" (Hewitt, 2012). One of the most important reasons why earlier work generated unreliable results is that the sample sizes were far too small, given that the true effects of individual genetic variants on behavioral outcomes are tiny. Pre-existing claims of genetic associations with complex social-science outcomes have reported widely varying effect sizes, many of them purporting to "predict" (see FAQ 1.5) ten to one hundred times as much of the variation across individuals as did the genetic variants we found in this study and in our other studies.

Second, behavioral genetics research also has the potential to correct the *social* record and thereby to help *combat* discrimination and stigmatization. For instance, at various times and places throughout human history (unfortunately, including the present day), girls and women have been discouraged or even prevented from pursuing as much education as their male counterparts. There are of course many reasons why that argument has been made and sometimes prevailed, but to the extent that it is rooted in a belief in genetically-based differences between males and females, our current study's (and our previous study's) analysis of the X chromosome finds no such evidence (see FAQ 2.9). Similarly, overestimating the role of genetics can be damaging, and the present work can help debunk this myth, too. Of the 20% of the variance in educational attainment that is related to the additive effects of common SNPs, we (Lee *et al.*, 2018) and others have found that the relationship to educational attainment depends importantly on environmental factors. By clarifying the *limits* of deterministic views of complex outcomes, recent behavioral genetics

research—if communicated responsibly—could make appeals to genetic justifications for discrimination and stigmatization *less* persuasive to the public in the future.

Third, behavioral genetics research has the potential to yield many other benefits, especially as sample sizes continue to increase—as briefly summarized in FAQ 1.7. Foregoing this research necessarily entails foregoing these and any other possible benefits, some of which will likely be the result of serendipity rather than being foreseeable. For instance, because educational attainment is measured in far larger genotyped samples than brain function, large-scale GWASs of educational attainment have provided better insights into brain function than GWASs to date that directly examine brain function, since the latter have necessarily been conducted in much smaller samples.

In sum, we agree with the U.K. Nuffield Council on Bioethics, which concluded in a report (Nuffield Council on Bioethics, 2002, p. 114) that "research in behavioural genetics has the potential to advance our understanding of human behaviour and that the research can therefore be justified," but that "researchers and those who report research have a duty to communicate findings in a responsible manner." In our view, responsible behavioral genetics research includes sound methodology and analysis of data; a commitment to publish all results, including any negative results; and transparent, complete reporting of methodology and findings in publications, presentations, and communications with the media and the public, including particular vigilance regarding what the results do—and do not—show and how they should—and should not—be used (hence, this FAQ document). In addition, we are developing a Terms of Use for researchers who would like to use our results in their own research. Researchers will agree to "have read and understand the principles articulated by the American Society of Human Genetics (ASHG) position statement: 'ASHG Denounces Attempts to Link Genetics and Racial Supremacy.' (See also International Genetic Epidemiological Society Statement on Racism and Genetic Epidemiology.)." Data-users also will acknowledge "I understand that comparisons of genetically predicted phenotype levels across ancestral groups are usually scientifically confounded due to the effects of linkage disequilibrium, gene-environment correlation, gene-environment interactions, and other methodological problems" (see FAQ 3.5).

Additional reading and references

Amos, C. I. *et al.* (2008) 'Genome-wide association scan of tag SNPs identifies a susceptibility locus for lung cancer at 15q25.1', *Nature Genetics*, 40, pp. 616–622. doi: 10.1038/ng.109.

Anderson, E. L. *et al.* (2017) 'The causal effect of educational attainment on Alzheimer's disease: A two-sample Mendelian randomization study', *bioRxiv [https://doi.org/10.1101/127993]*.

Bansal, V. *et al.* (2017) 'Genetics of educational attainment aid in identifying biological subcategories of schizophrenia', *bioRxiv [https://doi.org/10.1101/114405]*.

Barban, N. *et al.* (2016) 'Genome-wide analysis identifies 12 loci influencing human reproductive behavior', *Nature Genetics*, 48(12), pp. 1462–1472. doi: 10.1038/ng.3698.

Barcellos, S. H., Carvalho, L. S. and Turley, P. (2018) 'Education can Reduce Health Disparities Related to Genetic Risk of Obesity: Evidence from a British Reform', *bioRxiv [https://doi.org/10.1101/260463]*. doi: 10.1101/260463.

Belsky, D. W. *et al.* (2013) 'Development and evaluation of a genetic risk score for obesity', *Biodemography and Social Biology*, 59(1), pp. 85–100. doi: 10.1080/19485565.2013.774628.

Belsky, D. W. *et al.* (2016) 'The Genetics of Success', *Psychological Science*, 27(7), pp. 957–972. doi: 10.1177/0956797616643070.

Benjamin, D. J. *et al.* (2012) 'The Promises and Pitfalls of Genoeconomics', *Annual Review of Economics*, 4(1), pp. 627–662. doi: 10.1146/annurev-economics-080511-110939.

Branigan, A. R. *et al.* (2013) 'Variation in the Heritability of Educational Attainment: An International Meta-Analysis', *Social Forces*, 92(1), pp. 109–140. doi: 10.1093/sf/sot076.

Bulik-Sullivan, B. K. *et al.* (2015) 'LD Score regression distinguishes confounding from polygenicity in genome-wide association studies.', *Nature Genetics*, 47(3), pp. 291–295. doi: 10.1038/ng.3211.

Cesarini, D. and Visscher, P. M. (2017) 'Genetics and educational attainment', *npj Science of Learning*, 2(1), p. 4. doi: 10.1038/s41539-017-0005-6.

Chabris, C. F. *et al.* (2012) 'Most reported genetic associations with general intelligence are probably false positives', *Psychological Science*, 23(11), pp. 1314–1323. doi: 10.1177/0956797611435528.

Chabris, C. F. *et al.* (2015) 'The Fourth Law of Behavior Genetics', *Current Directions in Psychological Science*, 24(4), pp. 304–312. doi: 10.1177/0963721415580430.

Cheesman, R. *et al.* (2020) 'Comparison of Adopted and Nonadopted Individuals Reveals Gene–Environment Interplay for Education in the UK Biobank', *Psychological Science*, 31(5), pp. 582–591. doi: 10.1177/0956797620904450.

Conley, D. *et al.* (2016) 'Assortative mating and differential fertility by phenotype and genotype across the 20th century', *Proceedings of the National Academy of Sciences*, 113(24), pp. 6647–6652.

Cutler, D. M. and Lleras-Muney, A. (2010) 'Education and Health: Evaluating Theories and Evidence', in House, J. et al. (eds) *Making Americans Healthier: Social and Economic Policy as Health Policy*. New York: New York: Russell Sage Foundation, pp. 29–60.

Davies, N. M. *et al.* (2018) 'The causal effects of education on health outcomes in the UK Biobank', *Nature Human Behaviour*. doi: 10.1038/s41562-017-0279-y.

Domingue, B. W. *et al.* (2015) 'Polygenic Influence on Educational Attainment: New evidence from The National Longitudinal Study of Adolescent to Adult Health', *AERA Open*, 1(3), pp. 1–13. doi: 10.1177/2332858415599972.

Domingue, B. W. *et al.* (2017) 'Mortality selection in a genetic sample and implications for association studies', *International Journal of Epidemiology*, 46(4), pp. 1285–1294. doi: 10.1093/ije/dyx041.

Duncan, L. *et al.* (2019) 'Analysis of polygenic risk score usage and performance in diverse human populations', *Nature Communications*, 10(1), pp. 1–9. doi: 10.1038/s41467-019-11112-0.

Goldberger, A. S. A. (1979) 'Heritability', *Economica*, 46(184), pp. 327–347. Available at: http://www.jstor.org/stable/2553675.

Heath, A. C. *et al.* (1985) 'Education policy and the heritability of educational attainment', *Nature*, 314(6013), pp. 734–736. doi: doi:10.1038/314734a0.

Heckman, J. J. *et al.* (2010) 'The rate of return to the HighScope Perry Preschool Program', *Journal of Public Economics*, 94(1–2), pp. 114–128. doi: 10.1016/j.jpubeco.2009.11.001.

Hewitt, J. K. (2012) 'Editorial policy on candidate gene association and candidate gene-by-environment interaction studies of complex traits.', *Behavior Genetics*, 42(1), pp. 1–2. doi: 10.1007/s10519-011-9504-z.

Hill, W. G., Goddard, M. E. and Visscher, P. M. (2008) 'Data and theory point to mainly additive genetic variance for complex traits.', *PLoS Genetics*. Edited by T. F. C. Mackay, 4(2), p. e1000008. doi: 10.1371/journal.pgen.1000008.

Hivert, V. *et al.* (2021) 'Estimation of non-additive genetic variance in human complex traits from a large sample of unrelated individuals', *American Journal of Human Genetics*, 108(5), pp. 786–798. doi: 10.1016/J.AJHG.2021.02.014.

Houmark, M. A., Ronda, V. and Rosholm, M. (2020) *The Nurture of Nature and the Nature of Nurture: How Genes and Investments Interact in the Formation of Skills*. Bonn: Institute of Labor Economics (IZA). Available at: http://hdl.handle.net/10419/227307.

Hugh-Jones, D. *et al.* (2016) 'Assortative mating on educational attainment leads to genetic spousal resemblance for polygenic scores', *Intelligence*, 59, pp. 103–108. doi: 10.1016/j.intell.2016.08.005.

Hung, R. J. *et al.* (2008) 'A susceptibility locus for lung cancer maps to nicotinic acetylcholine receptor subunit genes on 15q25', *Nature*. doi: 10.1038/nature06885.

Jencks, C. (1980) 'Heredity, environment, and public policy reconsidered', *American Sociological Review*, 45(5), pp. 723–736. Available at: http://www.jstor.org/stable/2094892.

Jinks, J. and Eaves, L. J. (1974) 'IQ and Inequality', *Nature*, 248(5446), pp. 287–289. Available at: https://doi.org/10.1038/248287a0.

van Kippersluis, H. and Rietveld, C. A. (2018) 'Pleiotropy-robust Mendelian randomization', *International Journal of Epidemiology*, 47(4), pp. 1279–1288. doi: 10.1093/ije/dyx002.

Kong, A. *et al.* (2018) 'The nature of nurture: Effects of parental genotypes', *Science*, 359(6374), pp. 424–428. doi: 10.1126/science.aan6877.

Lambert, J.-C. *et al.* (2013) 'Meta-analysis of 74,046 individuals identifies 11 new susceptibility loci for Alzheimer's disease', *Nature Genetics*, 45(12), pp. 1452–1458. doi: 10.1038/ng.2802.

Lander, E. S. and Schork, N. J. (1994) 'Genetic dissection of complex traits', *Science*, 265, pp. 2037–48.

Lee, J. J. *et al.* (2018) 'Gene discovery and polygenic prediction from a genome-wide association study of educational attainment in 1.1 million individuals', *Nature Genetics*, 50(8), pp. 1112–1121. doi: 10.1038/s41588-018-0147-3.

Linnér, R. K. *et al.* (2017) 'An epigenome-wide association study meta-analysis of educational attainment', *Nature Publishing Group*. doi: 10.1038/mp.2017.210.

Locke, A. E. A. *et al.* (2015) 'Genetic studies of body mass index yield new insights for obesity biology', *Nature*, 518(7538), pp. 197–206. doi: 10.1038/nature14177.

Mare, R. D. (1991) 'Five decades of educational assortative mating', *American sociological review*, pp. 15–32.

Marioni, Riccardo E. *et al.* (2016) 'Genetic variants linked to education predict longevity', *Proceedings of the National Academy of Sciences*, 113(47), pp. 13366–13371. doi: 10.1073/pnas.1605334113.

Marioni, Riccardo E *et al.* (2016) 'The epigenetic clock and telomere length are independently associated with chronological age and mortality', *International journal of epidemiology*, pp. 1–9. doi: 10.1093/ije/dyw041.

Martin, A. R. *et al.* (2017) 'Human Demographic History Impacts Genetic Risk Prediction across Diverse Populations', *American Journal of Human Genetics*, 100(4), pp. 635–649. doi: 10.1016/j.ajhg.2017.03.004.

Nature Editors (2013) 'Dangerous work', *Nature*, 502(7469), pp. 5–6. doi: 10.1038/502005b.

Nuffield Council on Bioethics (2002) *Genetics and human behaviour: the ethical context*. London: Nuffield Council on Bioethics [http://nuffieldbioethics.org/wp-content/uploads/2014/07/Genetics-and-human-behaviour.pdf].

Okbay, A., Baselmans, B. M. L., *et al.* (2016) 'Genetic variants associated with subjective well-being, depressive symptoms, and neuroticism identified through genome-wide analyses', *Nature Genetics*, 48(6), pp. 624–633. doi: 10.1038/ng.3552.

Okbay, A., Beauchamp, J. P., *et al.* (2016) 'Genome-wide association study identifies 74 loci associated with educational attainment', *Nature*, 533(7604), pp. 539–542. doi: 10.1038/nature17671.

Parens, E. and Appelbaum, P. S. (2015) 'An introduction to thinking about trustworthy research into the genetics of intelligence', *Hastings Center Report*, 45(S1), pp. S2–S8. doi: 10.1002/hast.491.

Pazokitoroudi, A. *et al.* (2020) *Quantifying the contribution of dominance effects to complex trait variation in biobank-scale data*, *bioRxiv*. doi: 10.1101/2020.11.10.376897.

Pickrell, J. K. *et al.* (2016) 'Detection and interpretation of shared genetic influences on 42 human traits', *Nature Genetics*, 48(7), pp. 709–717. doi: 10.1038/ng.3570.

Rietveld, C. A. *et al.* (2013) 'GWAS of 126,559 individuals identifies genetic variants associated with educational attainment', *Science*, 340(6139), pp. 1467–1471. doi: 10.1126/science.1235488.

Ripke, S. *et al.* (2014) 'Biological insights from 108 schizophrenia-associated genetic loci', *Nature*, 511(7510), pp. 421–427. doi: 10.1038/nature13595.

Robinson, M. R. *et al.* (2017) 'Genetic evidence of assortative mating in humans', *Nature Human Behaviour*. doi: 10.1038/s41562-016-0016.

Ross, C. E. and Wu, C. (1995) 'The links between education and health', *American Sociological Review*, 60(5), pp. 719–745.

Sacerdote, B. (2007) 'How Large are the Effects from Changes in Family Environment? A Study of Korean American Adoptees', *The Quarterly Journal of Economics*, 122(1), pp. 119–157. doi: 10.1162/qjec.122.1.119.

Sacerdote, B. (2011) 'Nature and Nurture Effects On Children's Outcomes: What Have We Learned From Studies of Twins And Adoptees?', in Benhabib, J., Bisin, A., and Jackson, M. O. (eds) *Handbook of Social Economics*. Elsevier/North-Holland, pp. 1–29.

Schmitz, L. L. and Conley, D. (2017) 'The effect of Vietnam-era conscription and genetic potential for educational attainment on schooling outcomes', *Economics of Education Review*, 61, pp. 85–97. doi: https://doi.org/10.1016/j.econedurev.2017.10.001.

Silventoinen, K. *et al.* (2004) 'Heritability of body height and educational attainment in an international context: comparison of adult twins in Minnesota and Finland', *American Journal of Human Biology*, 16(5), pp. 544–555.

Thorgeirsson, T. E. *et al.* (2008) 'A variant associated with nicotine dependence, lung cancer and peripheral arterial disease', *Nature*, 452(7187), pp. 638–642. doi: 10.1038/nature06846.

Tillmann, T. *et al.* (2017) 'Education and coronary heart disease: Mendelian randomisation study', *BMJ (Online)*, 358, p. j3542. doi: 10.1136/bmj.j3542.

Turkheimer, E. (2000) 'Three laws of behavior genetics and what they mean', *Current Directions in Psychological Science*, 9(5), pp. 160–164.

Turley, P. *et al.* (2018) 'Multi-trait analysis of genome-wide association summary statistics using MTAG', *Nature Genetics*, 50(2), pp. 229–237. doi: 10.1101/118810.

Vassos, E. *et al.* (2017) 'An Examination of Polygenic Score Risk Prediction in Individuals With First-Episode Psychosis', *Biological Psychiatry*, 81(6), pp. 470–477. doi: 10.1016/j.biopsych.2016.06.028.

Visscher, P. M. *et al.* (2017) '10 Years of GWAS Discovery: Biology, Function, and Translation', *The American Journal of Human Genetics*, 101(1), pp. 5–22. doi: 10.1016/j.ajhg.2017.06.005.

Wang, Y. *et al.* (2020) 'Theoretical and empirical quantification of the accuracy of polygenic scores in ancestry divergent populations', *Nature Communications*, 11(1), pp. 1–9. doi: 10.1038/s41467-020-17719-y.

Warrier, V. *et al.* (2016) 'Genetic overlap between educational attainment, schizophrenia and autism', *bioRxiv [https://doi.org/10.1101/093575]*. doi: 10.1101/093575.

Weikart, D. P. and Perry Preschool Project (1967) *Preschool intervention; a preliminary report of the Perry Preschool Project.* Ann Arbor: Campus Publishers.

Winkler, T. W. *et al.* (2014) ‘Quality control and conduct of genome-wide association meta-analyses.’, *Nature Protocols*, 9(5), pp. 1192–1212.

Wood, A. R. *et al.* (2014) ‘Defining the role of common variation in the genomic and biological architecture of adult human height’, *Nature Genetics*, 46(11), pp. 1173–1186. doi: 10.1038/ng.3097.

Yengo, L., Robinson, M. R., *et al.* (2018) ‘Imprint of Assortative Mating on the Human Genome’, *Nature Human Behaviour*, 2(12), pp. 2, 948–954. doi: 10.1038/s41562-018-0476-3.

Yengo, L., Sidorenko, J., *et al.* (2018) ‘Meta-analysis of genome-wide association studies for height and body mass index in ∼700000 individuals of European ancestry.’, *Human molecular genetics*, 27(20), pp. 3641–3649. doi: 10.1093/hmg/ddy271.

Young, A. I. *et al.* (2020) ‘Mendelian imputation of parental genotypes for genome-wide estimation of direct and indirect genetic effects’, *bioRxiv*, p. 2020.07.02.185199. doi: 10.1101/2020.07.02.185199.
